# Divergent Access to Fluorinated Pharmacons From Ethyl Trifluoropyruvate

**DOI:** 10.1002/adsc.70384

**Published:** 2026-03-24

**Authors:** Rifat N. Nabi, Ryan C. Kashatus, Kaden M. Joseph, Salina Zhang, Daniel K. Kim

**Affiliations:** Department of Chemistry, Temple University, Philadelphia, Pennsylvania, USA

**Keywords:** difluoromethyl ketone, fluorine, Krapcho decarboxylation, monofluoromethyl ketone, trifluoroethanol, trifluoromethyl ketone, trifluoropyruvate

## Abstract

Fluoro(oxo) functional groups, such as trifluoromethyl ketones, trifluoroethanols, and difluoromethyl ketones, have emerged as desirable motifs in medicinal chemistry. Traditionally, the installation of these functional groups is accomplished through the use of either designer reagents or nucleophilic trifluoromethylation. In contrast, commercially available fluorinated building blocks, such as ethyl trifluoropyruvate, can be used in a divergent manner to access a wide variety of fluoro(oxo) functional groups. Herein, we demonstrate facile activation of trifluoromethyl(hydroxy) esters for the divergent synthesis of fluorinated pharmacons.

## 1 | Introduction

The development of sustainable and selective methods for the incorporation of fluorine-based functional groups remains a cornerstone of modern organic synthesis. One in five pharmaceuticals and half of all agrochemicals contain at least one fluorine atom [1, 2]. As such, fluorinated pharmacons are an attractive choice in drug discovery for their inherent tunability in both pharmacokinetic and pharmacodynamic properties [3–7]. As a subclass of bioactive fluorinated compounds, fluoro(oxo) containing motifs, such as trifluoromethyl ketones (TFMKs), difluoromethyl ketones (DFMKs), monofluoromethyl ketones (MFMKs), trifluoroethanols (TFEs), and chlorodifluoroethanols (CDFEs), are of particular interest owing to their versatility as synthetic intermediates, as well as their ability to be potent carboxylic acid bioisosteres [8, 9]. Additionally, fluoro(oxo) pharmacons are employed for attenuation in lipophilicity, binding affinity, hydrogen bonding ability, and are used as reversible covalent inhibitors [8, 10, 11].

**FIGURE 1 |:**
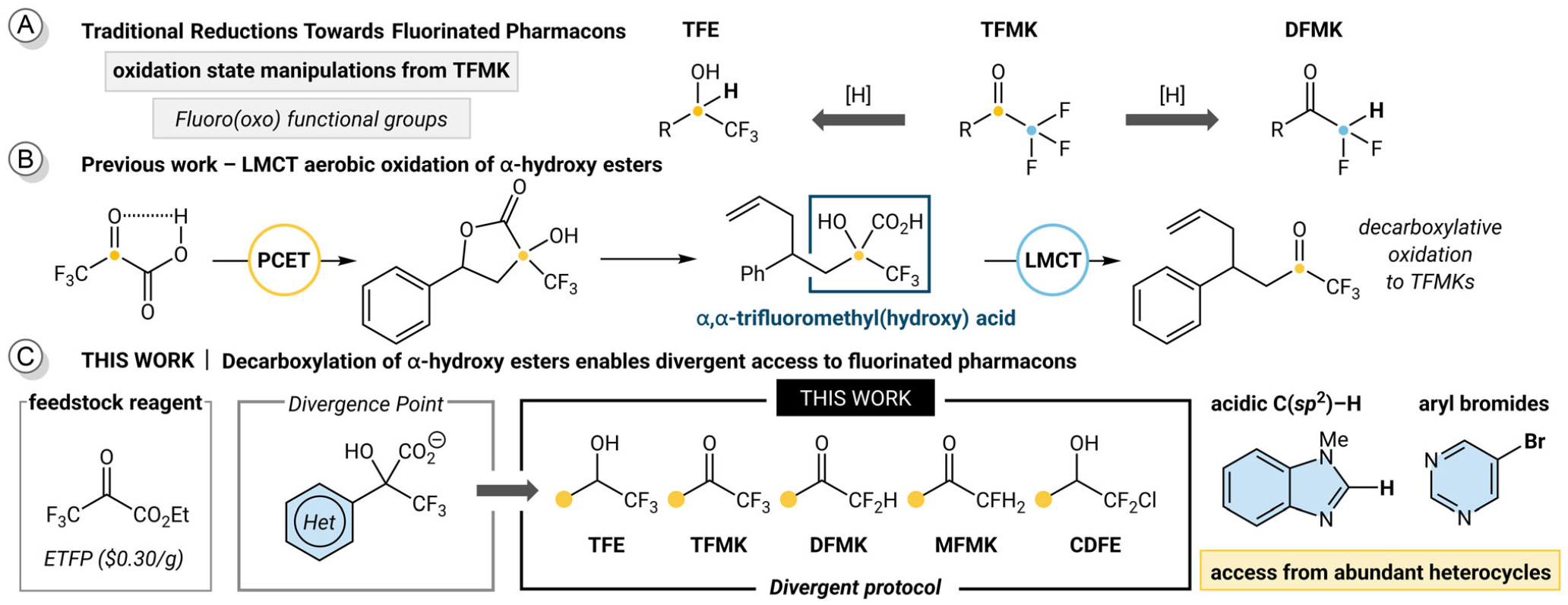
(A) Trifluoromethyl ketones as a common intermediate for fluorinated functional groups. (B) Decarboxylative oxidation of α,α-trifluoromethyl(hydroxy) acids to TFMKs. (C) Divergent functionalization of trifluoromethyl(hydroxy) esters to fluorinated products.

Over the past few decades, fluoro(oxo) functional group diversity has been accessed through oxidation state manipulations from the parent TFMK, either by carbonyl reduction to give the TFE [12] or elimination to yield the DFMK (Figure 1A) [13]. Among the synthetic methods to install these fluorinated motifs, there are many that employ designer reagents derived from fluorinated feedstocks [14–17]. These methods are invaluable for the selective (oxo)fluorination of complex molecules; however, complementary methods that install these motifs in a divergent manner are also highly desirable [18–22].

Our group recently reported an intramolecular proton-coupled electron transfer (PCET) activation of fluorinated α-keto acids. A key insight from our previous work is the identification of an α,α-trifluoromethyl(hydroxy) acid as an intermediate to TFMKs, a desirable fluoro(oxo) functional group (Figure 1B) [23]. In our previous study, we focused our attention on styrenyl substrates and now aim to incorporate pharmaceutically relevant heterocycles. Additionally, we desire broad access to fluorinated chemical space beyond TFMKs. To impart structural diversity and expand the synthetic methods for installing fluorinated carboxylic acid surrogates, we envisioned harnessing the extremely abundant pool of commercially available heterocycles, particularly those containing acidic C(*sp*^2^)-H bonds or halogenated handles [24]. Herein, our design relied on synthesizing a library of α,α-trifluoromethyl(hydroxy) esters, which could be accessed from ethyl trifluoropyruvate (ETFP), an economical and bench stable feedstock. By carefully tuning reaction conditions, our trifluoromethyl(hydroxy) ester intermediate can serve as the synthetic handle to enable a divergent synthesis to TFEs, TFMKs, DFMKs, MFMKs, and CDFEs (Figure 1C).

## 2 | Results and Discussion

A library of α,α-trifluoromethyl(hydroxy) esters was synthesized from either lithium-halogen exchange or direct lithiation of a diverse set of heterocyclic precursors, followed by nucleophilic addition into ETFP (**2**) (see Supporting Information for details). Informed by previously reported aerobic decarboxylation methods [23, 25, 26], we sought to engage ethyl 3,3,3-trifluoro-2-hydroxy-2-(pyridin-3-yl)propanoate (**1**) for the synthesis of TFMKs. Taking into account the necessity for saponification of the starting ester, a modified procedure for the Fe(III)-mediated aerobic decarboxylation of α,α-trifluoromethyl(hydroxy) acids proved effective and gives **1-TFMK** in 73% isolated yield (Table 1, entry 1).

With oxidative decarboxylation conditions in hand, we turned our efforts toward a complementary and chemoselective protocol that would directly generate the desired trifluoroethanol **1-TFE** from a common intermediate. We can take advantage of the strong electron-withdrawing properties of the trifluoromethyl group of **1** in a Krapcho decarboxylation through the stabilization of a key carbanion intermediate [27, 28]. However, a rapid elimination of fluoride provides a potential chemoselectivity challenge [29]. Initial subjection of the model substrate **1** to typical Krapcho conditions [30] (LiCl and H_2_O at 130°C in DMF) results in poor yields and a mixture of both **1-TFE** and **1-DFMK** (entry 2). We hypothesized that under typical Krapcho conditions, the resulting benzylic anion intermediate competes between protonation (to **1-TFE**) or elimination (to **1-DFMK**) [30, 31]. As such, we identified conditions that delivered **1-TFE** with high chemoselectivity using *tert*-butyldimethylsilyl chloride (TBS-Cl) to attenuate the elimination pathway (Table 1, entry 3) [32]. In a one-pot process, **1** undergoes a sequential silylation-decarboxylation, yielding α-trifluoromethyl silyl ether **1-TBS**, and a subsequent workup with tetrabutylammonium fluoride (TBAF) yields the desired trifluoroethanol **1-TFE** in 95% isolated yield. Spectroscopic evidence for the formation of chloroethane suggests that TBS-Cl acts as the chloride source to facilitate the decarboxylation. As an alternative, the decarboxylation is equally successful with trimethylsilyl chloride (see Supporting Information for details). However, we opted to use the former due to the higher stability of its derivatives.

Continuing the objective of accessing fluoro(oxo) functional groups in a divergent manner, we hypothesized that interrupting the one-pot synthesis toward TFEs and using the intermediate α,α-trifluoromethyl((TBS)oxy) arenes could enable a path toward DFMKs that circumvents the previously encountered chemoselectivity challenges. Taking advantage of the acidic benzylic α-protons on the aforementioned α,α-trifluoromethyl((TBS)oxy) arenes, deprotonation with NaHMDS at −78°C results in elimination of fluoride and delivers the corresponding difluoro silyl enol ether. In a telescoped sequence, this difluoro silyl enol ether can be desilylated to the DFMK under acidic conditions (Table 1, entry 4) to yield **1-DFMK** in 90% isolated yield over two steps from **1**.

**FIGURE 2 |:**
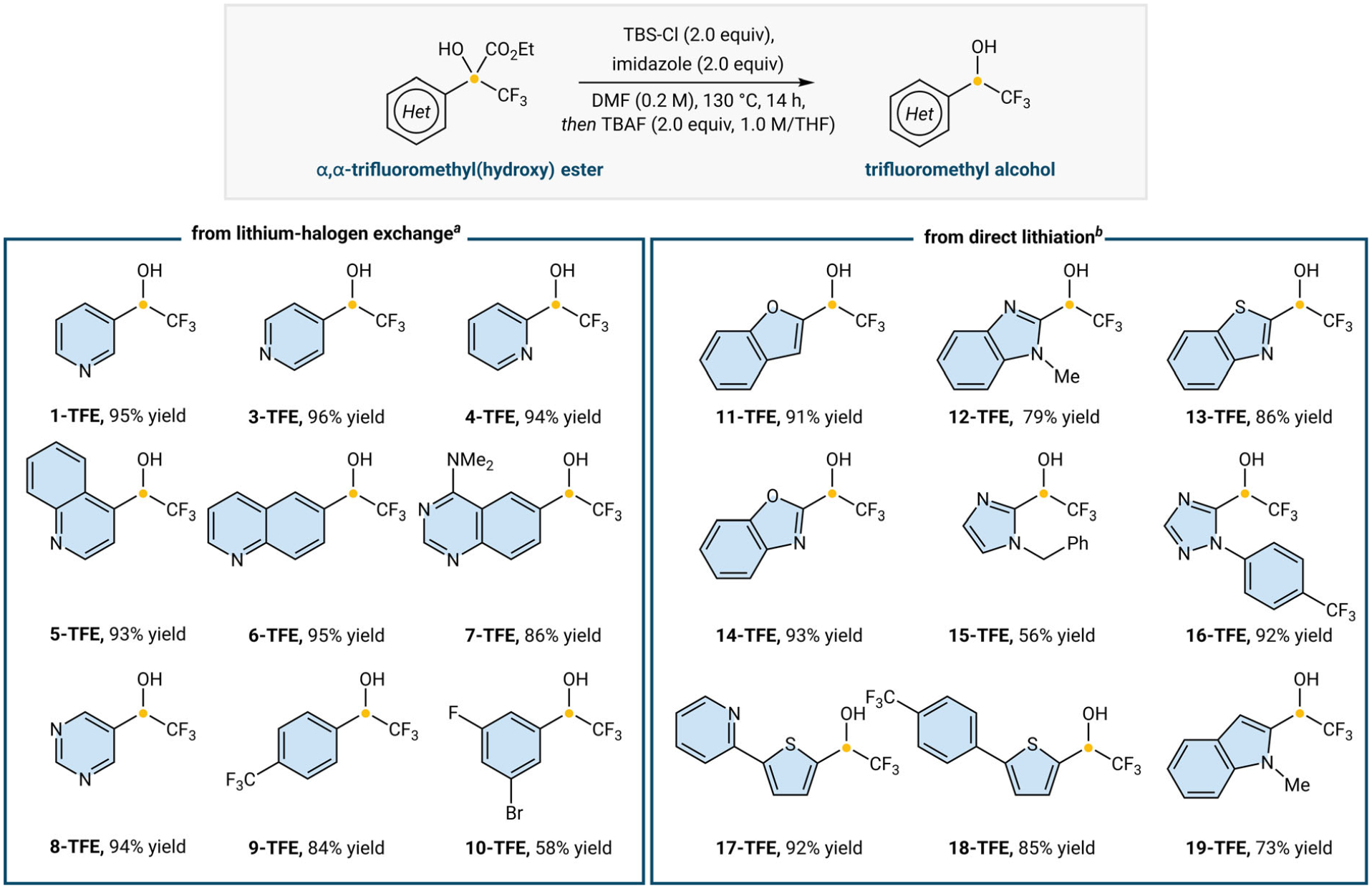
Synthesis of trifluoroethanols. ^a^From the corresponding aryl bromide. ^b^From the corresponding heterocycle.

**FIGURE 3 |:**
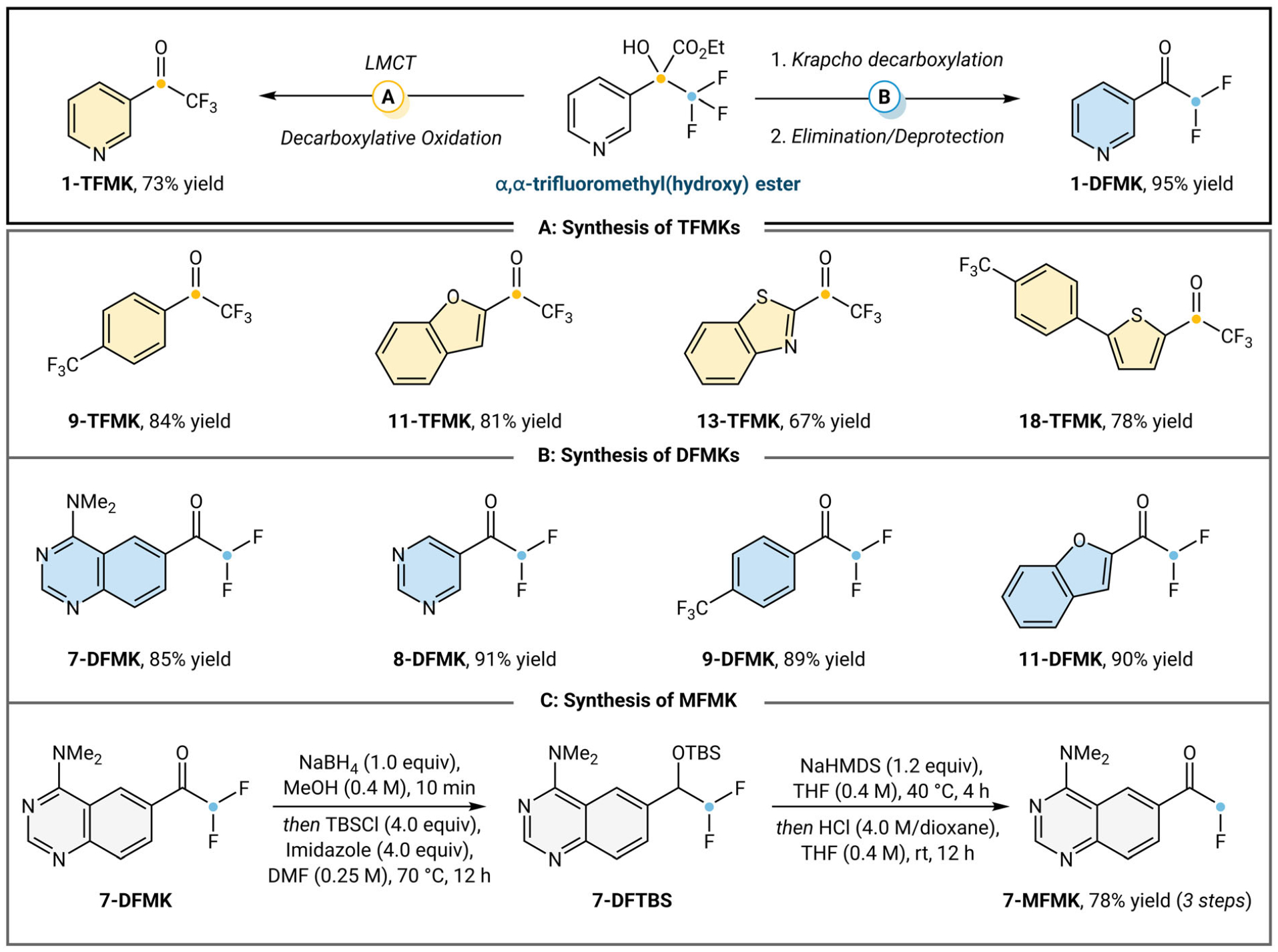
Synthesis of fluorinated ketones. Condition **A**: Fe(acac)_3_ (10 mol%), NaOH (1.0 equiv), 1:1 THF: H_2_O (0.1 M), 390 nm LEDs, O_2_ (1.0 atm), 12 h. Condition **B**, elimination/deprotection: NaHMDS (1.2 equiv), THF (0.4 M), 0°C, 30 min *then* HCl (0.4 M/dioxane), THF (0.4 M), 12 h, rt.

With optimized conditions in hand, we evaluated the scope of α,α-trifluoromethyl(hydroxy) esters as a precursor for the synthesis of TFEs (Figure 2). First, we explored aryl bromides and iodides that could undergo lithium-halogen exchange to give the respective ester. The model substrate ethyl 3,3,3-trifluoro-2-hydroxy-2-(pyridin-3-yl)propanoate (**1**) undergoes decarboxylation to the desired **1-TFE** in 95% isolated yield. To showcase pyridine functionalization diversity, we are able to alter substitution to both the 4- and 2-positions, which gives high yields of the pyridines **3-TFE** and **4-TFE** (96% and 94% yields, respectively). Quinoline-containing α,α-trifluoromethyl(hydroxy) esters are well-tolerated and give the respective alcohols **5-TFE** and **6-TFE** (93% and 95% yields). Other electron-deficient heterocycles, such as diazines, are also amenable to the decarboxylation protocol, providing quinoxaline **7-TFE** in 86% yield and pyrimidine **8-TFE** in 94% yield. An aryl trifluoromethyl(hydroxy) ester also proves successful in the synthesis of **9-TFE** in 84% yield. Notably, 1,3-dibromo-5-fluorobenzene engages in a monolithium-halogen exchange to ultimately give rise to aryl bromide-containing **10-TFE** in a moderate 58% yield, which can be used as a building block for transition metal-mediated cross-couplings. We additionally explored the ability to engage heterocycles through direct lithiation to further broaden our heteroaryl scope (Figure 3). Electron-rich ester **11** can be subjected to the decarboxylation protocol and gives **11-TFE** in 91% yield. Esters derived from azoles, such as *N*-methylbenzimidazole, benzothiazole, benzoxazole, imidazole, and 1,2,4-triazole, are well-tolerated, giving good to excellent yields for the respective fluorinated ethanols **12–16-TFE** (56%–93% yields). Thiophenyl and electron-rich indole esters are also successful in decarboxylation resulting in **17–19-TFE** (73%–92% yield).

In order to further demonstrate α,α-trifluoromethyl(hydroxy) esters as a viable synthetic intermediate toward the synthesis of fluorinated pharmacons, we expanded the diversification scope of DFMKs and TFMKs (Figure 3). The α,α-trifluoromethyl(hydroxy) esters can be saponified and subsequently decarboxylated in one pot toward the synthesis of TFMKs. Electron-deficient trifluoromethyl(hydroxy) ester **1** converts smoothly to **1-TFMK** (73% yield) upon irradiation with 390 nm LEDs and Fe(acac)_3_ under aerobic conditions (Figure 3A). We were pleased to find that our decarboxylative oxidation system enables the synthesis of aryl containing **9-TFMK** in 84% yield and electron-rich **11-TFMK** in 81% yield. Excitingly, electron-rich azole and thiophenyl TFMKs are also synthesized according to our protocol in 67% and 78% yields, respectively (**13-TFMK** and **18-TFMK**). Furthermore, following Krapcho decarboxylation, we can subject **1-TBS** to a benzylic deprotonation-elimination sequence with NaHMDS to yield the respective silyl enol ether. This enol ether is then directly desilylated to give the desired **1-DFMK** in 95% isolated yield. Diazines are also amenable to our methodology, with **7-DFMK** and **8-DFMK** accessed in 85% and 91% yields, respectively (Figure 3B). Compound **9-DFMK** was accessed from simple fluorinated arene in 89% yield. In addition, the electron-rich benzofuran-derived silyl enol ether proceeds to furnish **11-DFMK** in 90% yield. We are able to iterate this elimination-desilylation toward the synthesis of a MFMK (Figure 3C). Reduction and silyl protection of **7-DFMK** affords difluoromethyl silyl ether **7-DFTBS**. Through a similar benzylic deprotonation-elimination sequence, followed by desilylation, **7-MFMK** is synthesized in 78% yield over three steps from **7-DFMK**.

**FIGURE 4 |:**
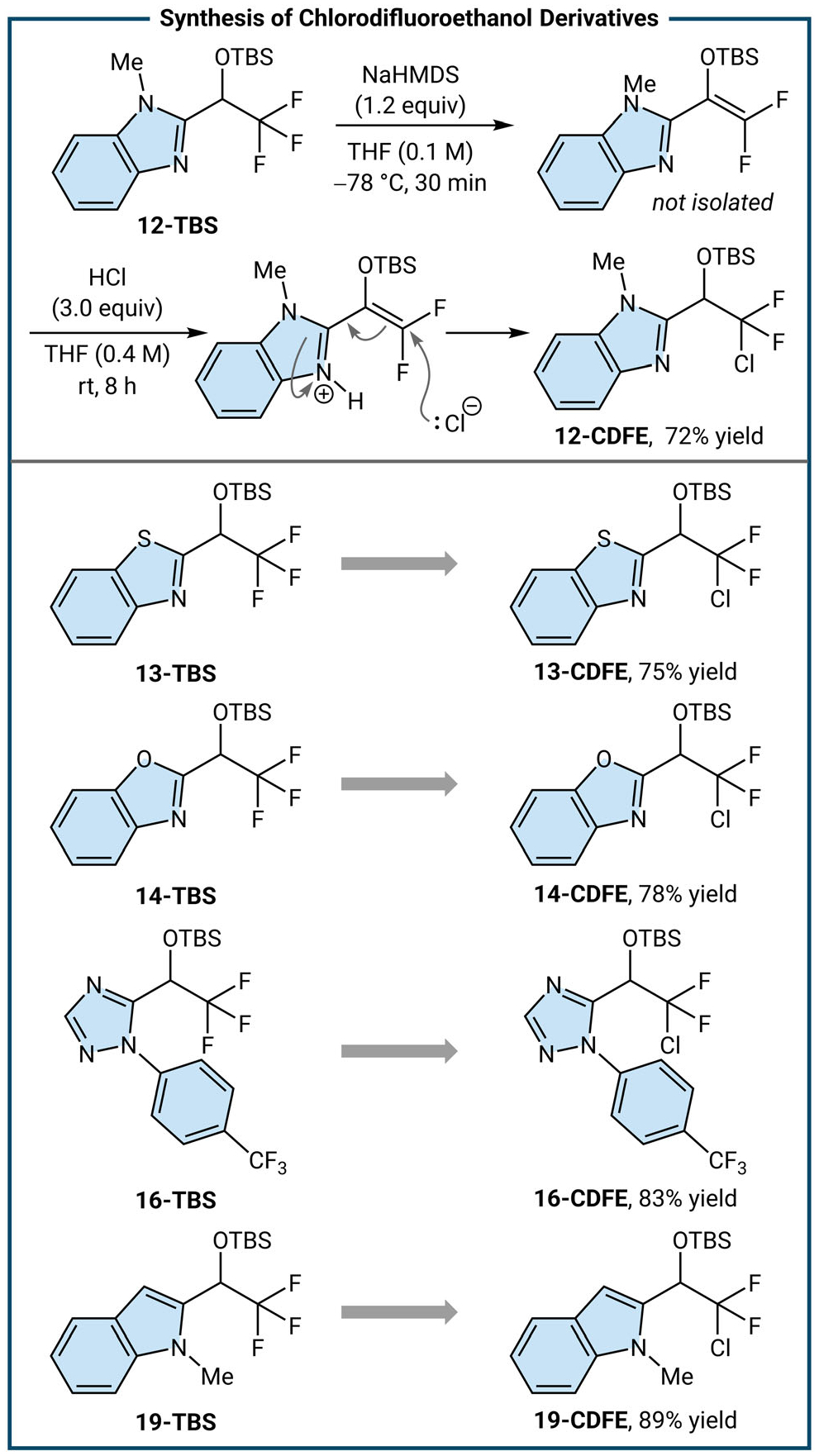
Evaluation of one-pot procedure of chlorodifluoromethyl silyl ethers and proposed mechanism.

During our efforts to convert the trifluoromethyl((TBS)oxy) arene intermediates to DFMKs, we imagined that silyl difluoro enol ethers derived from azole substrates may be activated for conjugate addition under acidic conditions owing to the LUMO lowering effect of iminiums [33, 34]. Indeed, trifluoromethyl((TBS) oxy) benzimidazole (**12-TBS**) converts to protected CDFE **12-CDFE** in 72% yield (Figure 4). Additional chlorinated silyl ethers derived from azole trifluoromethyl silyl ethers **13-CDFE**, **14-CDFE**, and **16-CDFE** are synthesized in good yields (75%–83% yield). This reaction protocol has also been extended to enamine-type trifluoromethyl((TBS)oxy) arenes, such as *N*-methylindole silyl enol ether derived from **19-TBS**, resulting in chlorodifluoromethyl silyl ether **19-CDFE** in 89% yield.

## 3 | Conclusion

In summary, we have demonstrated the activation of α,α-trifluoromethyl(hydroxy) esters through either a Krapcho decarboxylation or LMCT photochemistry for the synthesis of fluorinated pharmacons. This divergent synthesis protocol tolerates a wide range of esters bearing aryl and heteroaryl substituents of varying electronic properties, using ETFP as a cheap and commercially available fluorinated building block. We hope these strategies will enable late-stage diversified syntheses in pursuit of fluoro(oxo) pharmacons for agrochemical and pharmaceutical industries.

## Supplementary Material

si

Additional supporting information can be found online in the Supporting Information section. Supporting information for this article is given via a link at the end of the document. The supporting information is attached. **Supporting Fig. S1**: **Evaluation of solvents and additives**. **Supporting Fig. S2**: Temperature evaluation of thermal decarboxylation. ^a^Isolated yield. **Supporting Fig. S3**: Thermal silylation-decarboxylation control reactions. ^a^Isolated yield. **Supporting Fig. S4**: Aerobic LMCT-mediated decarboxylation control reactions. ^a^Isolated yield.

## Figures and Tables

**TABLE 1 |:** Selected reaction optimization.

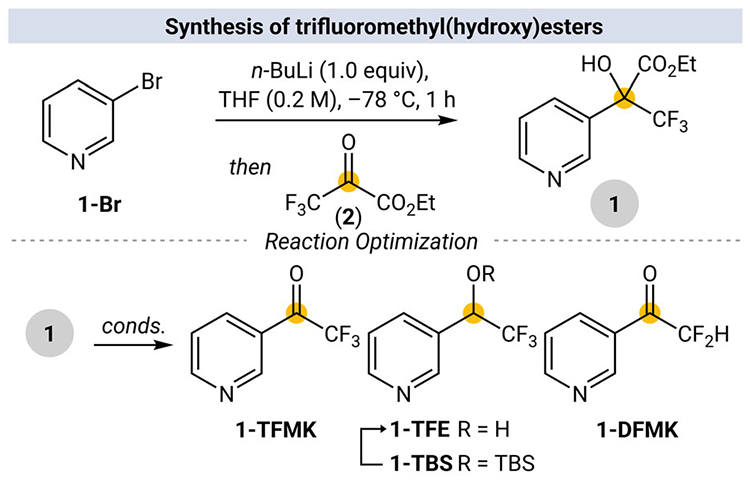				
Entry	Conditions	1-TFMK (%)^a^	1-TFE (%)^a^	1-DFMK (%)^a^
1	Fe(acac)_3_ (10 mol%), NaOH (1.0 equiv), 1:1 THF:H_2_O (0.1 M), O_2_, 14 h	75 **(73)**^b^	0	0
2	LiCl (3.0 equiv), H_2_O (3.0 equiv), DMF (0.2 M), 130°C, 14 h	0	25	12
3	TBS-Cl (2.0 equiv), imidazole (2.0 equiv), DMF (0.2 M), 130°C, 14 h, then TBAF (2.0 equiv) in THF	0	98 **(95)**^b^	0
4	**1.** TBS-Cl (2.0 equiv), imidazole (2.0 equiv), DMF (0.2 M), 130°C, 14 h **2.** NaHMDS (1.2 equiv), THF (0.1 M), −78°C, 30 min, then HCl (3.0 equiv), THF (0.4 M)	0	0	90^b,c^

a Yields determined by ^19^F NMR with benzotrifluoride as internal standard.

b Isolated yield.

c Yield over 2 steps.

## Data Availability

The data that support the findings of this study are available in the Supporting Information of this communication.

## References

[1] Inoue M, Sumii Y, and Shibata N, “Contribution of Organofluorine Compounds to Pharmaceuticals,” ACS Omega 5 (2020): 10633–10640. 32455181 10.1021/acsomega.0c00830 PMC7240833

[2] Zhou Y, Wang J, Gu Z, , “Next Generation of Fluorine-Containing Pharmaceuticals, Compounds Currently in Phase II–III Clinical Trials of Major Pharmaceutical Companies: New Structural Trends and Therapeutic Areas,” Chemical Reviews 116 (2016): 422–518. 26756377 10.1021/acs.chemrev.5b00392

[3] Meanwell NA, “Fluorine and Fluorinated Motifs in the Design and Application of Bioisosteres for Drug Design,” Journal of Medicinal Chemistry 61 (2018): 5822–5880. 29400967 10.1021/acs.jmedchem.7b01788

[4] Meanwell NA, “Applications of Bioisosteres in the Design of Biologically Active Compounds,” Journal of Agricultural and Food Chemistry 71 (2023): 18087–18122. 36961953 10.1021/acs.jafc.3c00765

[5] Gillis EP, Eastman KJ, Hill MD, Donnelly DJ, and Meanwell NA, “Applications of Fluorine in Medicinal Chemistry,” Journal of Medicinal Chemistry 58 (2015): 8315–8359. 26200936 10.1021/acs.jmedchem.5b00258

[6] Chao H, Tuerdi H, Kick E, and Yang W, Benzazepine Derivatives as LXR Modulators and Their Preparation, Pharmaceutical Compositions and Use in the Treatment of Various Diseases, 2006.

[7] Tanaka T, Nishimura Y, Shimada Y, , Preparation of Guaiazulene Derivative as Antitumor Agents, 2016.

[8] Ballatore C, Huryn DM, and Smith AB, “Carboxylic Acid (Bio) Isosteres in Drug Design,” ChemMedChem 8 (2013): 385–395. 23361977 10.1002/cmdc.201200585 PMC3640829

[9] Citarella A and Micale N, “Peptidyl Fluoromethyl Ketones and Their Applications in Medicinal Chemistry,” Molecules (basel, Switzerland) 25 (2020): 4031. 32899354 10.3390/molecules25174031 PMC7504820

[10] Kelly CB, Mercadante MA, and Leadbeater NE, “Trifluoromethyl Ketones: Properties, Preparation, and Application,” Chemical Communications 49 (2013): 11133. 24162741 10.1039/c3cc46266h

[11] Citarella A, Gentile D, Rescifina A, , “Pseudo-Dipeptide Bearing α,α-Difluoromethyl Ketone Moiety as Electrophilic Warhead with Activity against Coronaviruses,” International Journal of Molecular Sciences 22 (2021): 1398. 33573283 10.3390/ijms22031398 PMC7866854

[12] Stewart R and Teo KC, “The Reduction of Aryl Trifluoromethyl Ketones by Sodium Borohydride. The Hydride Transfer Process,” Canadian Journal of Chemistry 58 (1980): 2491–2496.

[13] Surya Prakash GK, Hu J, and Olah GA, “Facile Preparation of Di- and Monofluoromethyl Ketones from Trifluoromethyl Ketones via Fluorinated Enol Silyl Ethers,” Journal of Fluorine Chemistry 112 (2001): 355–360.

[14] Giri R, Zhilin E, and Katayev D, “Divergent Functionalization of Alkenes Enabled by Photoredox Activation of CDFA and α-Halo Carboxylic Acids,” Chemical Science 15 (2024): 10659–10667. 38994427 10.1039/d4sc01084a PMC11234866

[15] Han S, Samony KL, Nabi RN, Bache CA, and Kim DK, “Hydrotrifluoroacetylation of Alkenes via Designer Masked Acyl Reagents,” Journal of the American Chemical Society 145 (2023): 11530–11536. 37192402 10.1021/jacs.3c04294

[16] Lombardi L, Cerveri A, Giovanelli R, , “Direct Synthesis of α-Aryl-α-Trifluoromethyl Alcohols via Nickel Catalyzed Cross-Electrophile Coupling,” Angewandte Chemie International Edition 61 no.47 (2022): e202211732 10.1002/anie.202211732. 36161744 PMC9828748

[17] Gallego-Gamo A, Pleixats R, Gimbert-Suriñach C, Vallribera A, and Granados A, “Hydroxytrifluoroethylation and Trifluoroacetylation Reactions via SET Processes,” Chemistry – A European Journal 30 no.18 (2024): e202303854, 10.1002/chem.202303854. 38183331

[18] Pierce ME, Parsons RL, Radesca LA, , “Practical Asymmetric Synthesis of Efavirenz (DMP. 266), an HIV-1 Reverse Transcriptase Inhibitor,” Journal of Organic Chemistry 63 (1998): 8536–8543.

[19] Cabrera PJ, Allais C, Arcari JT, González-Esguevillas M, McInturff EL, and Reese MR, “Rapid Multikilogram Scale-Up of Di- and Trifluoromethoxy Proline Derivatives,” Organic Process Research and Development 28 (2024): 1119–1128.

[20] Zhang K, Rombach D, Nötel NY, Jeschke G, and Katayev D, “Radical Trifluoroacetylation of Alkenes Triggered by a Visible-Light-Promoted C–O Bond Fragmentation of Trifluoroacetic Anhydride,” Angewandte Chemie International Edition 60 (2021): 22487–22495. 34289531 10.1002/anie.202109235 PMC8518413

[21] Campbell MW, Polites VC, Patel S, Lipson JE, Majhi J, and Molander GA, “Photochemical C–F Activation Enables Defluorinative Alkylation of Trifluoroacetates and -Acetamides,” Journal of the American Chemical Society 143 (2021): 19648–19654. 34793157 10.1021/jacs.1c11059 PMC9172939

[22] Box JR, Atkins AP, and Lennox AJJ, “Direct Electrochemical Hydrodefluorination of Trifluoromethylketones Enabled by Non-Protic Conditions,” Chemical Science 12 (2021): 10252–10258. 34377412 10.1039/d1sc01574e PMC8336478

[23] Nabi RN, Jarquin KA, Karmakar A, Brunner KE, and Kim DK, “Intramolecular PCET of α-Keto Acids: Synthesis of Trifluoromethyl Ketones via Ketyl Radicals,” Chemistry – A European Journal 31 no. 37 (2025): e20250161. 10.1002/chem.202501613. PMC12223336 40402022

[24] Vitaku E, Smith DT, and Njardarson JT, “Analysis of the Structural Diversity, Substitution Patterns, and Frequency of Nitrogen Heterocycles among U.S. FDA Approved Pharmaceuticals,” Journal of Medicinal Chemistry 57 (2014): 10257–10274. 25255204 10.1021/jm501100b

[25] Tu J-L, Gao H, Luo M, , “Iron-Catalyzed Ring-Opening of Cyclic Carboxylic Acids Enabled by Photoinduced Ligand-to-Metal Charge Transfer,” Green Chemistry 24 (2022): 5553–5558.

[26] Blay G, Fernández I, Marco-Aleixandre A, Monje B, Pedro JR, and Ruiz R, “Catalytic Aerobic Oxidative Decarboxylation of α-Trifluoromethyl-α-Hydroxy Acids to Trifluoromethyl Ketones,” Tetrahedron 58 (2002): 8565–8571.

[27] Krapcho AP, “Recent Synthetic Applications of the Dealkoxycarbonylation Reaction. Part 1. Dealkoxycarbonylations of Malonate Esters,” Arkivoc: Free Online Journal of Organic Chemistry 2007 (2008): 1–53.

[28] Krapcho AP, “Recent Synthetic Applications of the Dealkoxycarbonylation Reaction. Part 2. Dealkoxycarbonylations of β-Keto Esters, α-Cyanoesters and Related Analogues,” Arkivoc: Free Online Journal of Organic Chemistry 2008 (2008): 54–120.

[29] Kiyoharu Nishide TKK, Kobori Takeo, and Daiei, “Synthesis of Novel Fluorine-Containing Cephalosporins,” Heterocycles 26 (1987): 633–640.

[30] Krapcho AP, Weimaster JF, Eldridge JM, Jahngen EGE, Lovey AJ, and Stephens WP, “Synthetic Applications and Mechanism Studies of the Decarbalkoxylations of Geminal Diesters and Related Systems Effected in Dimethyl Sulfoxide by Water and/or by Water with Added Salts,” Journal of Organic Chemistry 43 (1978): 138–147.

[31] Duchemin N, Buccafusca R, Daumas M, Ferey V, and Arseniyadis S, “A Unified Strategy for the Synthesis of Difluoromethyl- and Vinylfluoride-Containing Scaffolds,” Organic Letters 21 (2019): 8205–8210. 31566980 10.1021/acs.orglett.9b02887

[32] Dankert F and von Hänisch C, “Siloxane Coordination Revisited: Si−O Bond Character, Reactivity and Magnificent Molecular Shapes,” European Journal of Inorganic Chemistry 2021 (2021): 2907–2927.

[33] Baum JS and Viehe HG, “Synthesis and Cycloaddition Reactions of Acetylenic Iminium Compounds,” Journal of Organic Chemistry 41 (1976): 183–187.

[34] Petronijević FR, Nappi M, and MacMillan DWC, “Direct β-Functionalization of Cyclic Ketones with Aryl Ketones via the Merger of Photoredox and Organocatalysis,” Journal of the American Chemical Society 135 (2013): 18323–18326. 24237366 10.1021/ja410478a PMC3934322

