# Supplementary material for: Divergent Access to Fluorinated Pharmacons From Ethyl Trifluoropyruvate
Source: Adv Synth Catal. Author manuscript; Available in PMC 2026 May 21. (PMC13188445; doi:10.1002/adsc.70384)
Supplement: si [file NIHMS2175321-supplement-si.pdf]

Supporting Information

Divergent Access to Fluorinated Pharmacons from Ethyl Trifluoropyruvate

Rifat N. Nabi, Ryan C. Kashatus, Kaden M. Joseph, Salina Zhang, and Prof. Dr. Daniel K. Kim*

Department of Chemistry, Temple University

1901 North 13th Street,

Philadelphia, Pennsylvania, 19122, United States

*corresponding author. Email: danielkim@temple.edu

Table of Contents

General Information 3

Reaction Optimization 4

Preparation of Starting Materials 8

General Procedure A: Preparation of α,α-trifluoromethyl(hydroxy) Esters 11

General Procedure B: Decarboxylation of α,α-trifluoromethyl(hydroxy) Esters 30

General Procedure C: Silylation-Decarboxylation of α,α-trifluoromethyl(hydroxy) Esters 49

General Procedure D: Preparation of Difluoromethyl Ketones 60

General Procedure E: Preparation of Trifluoromethyl Ketones 67

General Procedure F: Preparation of Chlorodifluoromethyl Silyl Ethers 73

Preparation of Monofluoromethyl Ketone (7-MFMK) 79

Procedure for the Detection of Chloroethane 81

References 82

^{1}H, ^{13}C, and ^{19}F NMR spectra of all compounds 83

General Information

Commercially available reagents were used without additional purification, unless otherwise indicated. Reaction vials (8 mL) were purchased from Fischer Scientific, oven dried overnight and cooled to room temperature prior to use. Solvents were purified using a Pure Process Technology 5-Solvent Purification System (DMF, MeCN, THF, DCM, Et_2O). Unless otherwise noted, reactions were performed with rigorous exclusion of air and moisture. Thin layer chromatography was carried out using Silica XHL TLC Plates w/ UV254 (Sorbent Technologies, PN: 4115126). For flash column chromatography, Silica Gel, Enhanced Grade, 60A, 40-63μm was used (Sorbent Technologies, PN: 40930M-25). Purification was carried out using ACS grade solvents. Melting points were determined using a DigiMelt MPA160 SRS melting point apparatus at a ramp rate of 5 °C/min. Infrared spectra were obtained using a Thermo Nicolet iS5 FTIR spectrometer with an iD5 ATR accessory and are reported as cm^{-1}.

Nuclear magnetic resonance spectra (^{1}H NMR, ^{13}C NMR, ^{19}F NMR) were recorded on Bruker Unity 400 MHz or 500 MHz spectrometers in $CDCl_3$, DMSO-d_6, or MeOD-d_4 solutions. ^{1}H NMR spectra were internally referenced to the residual proteo-solvent signals (7.26 ppm for $CDCl_3$; 2.50 ppm for DMSO-d_6; 3.34 for MeOD-d_4). ^{1}H NMR data are reported in terms of chemical shift (δ, ppm), multiplicity (s = singlet, d = doublet, t = triplet, q = quartet, p = pentet, m = multiplet, br = broad), coupling constant (Hz), and integration. ^{13}C NMR spectra were internally referenced to the residual solvent signal. ^{13}C NMR data are reported in terms of chemical shift (δ, ppm), multiplicity (t = triplet, q = quartet), and coupling constant (Hz) when there is coupling. ^{19}F NMR spectroscopy was performed in the same deuterated solvent as ^{1}H NMR and the resulting spectra are unreferenced. ^{19}F NMR data are reported in terms of chemical shift (δ, ppm), multiplicity (s = singlet, d = doublet, t = triplet, q = quartet, m = multiplet, br = broad), coupling constant (Hz), and integration. All ^{19}F NMR spectra were recorded with ^{1}H coupling.

Accurate masses for derivatized products were conducted on an Agilent 6520 Accurate-Mass Q-TOF LC/MS. Samples were taken up in a suitable solvent (MeCN) for analysis. Accurate mass measurement (AMM) analyses were conducted on either a Waters GCT Premier, time-of-flight, GCMS with electron ionization (EI-TOF), or an LCT Premier XE, time-of-flight, LCMS with electrospray ionization (ESI-TOF). The signals were mass measured against an internal lock mass reference of perfluorotributylamine (PFTBA) for EI-GCMS and leucine enkephalin for ESI-LCMS, positive and negative ion modes. Waters software calibrates the instruments and reports measurements by use of neutral atomic masses. The mass of the electron was not included.

Reaction Optimization

Procedure for Optimization Studies 1

To an oven-dried 8 mL vial and stir bar was added α,α-trifluoromethyl(hydroxy) ester **1** (49.8 mg 0.2 mmol, 1.0 equiv), additives (0.6 mmol, 3.0 equiv), and solvent (1.0 mL, 0.2 M). All reagents were added open to air and no further considerations were made to purge the solution. The vial was tightly sealed with a screw-on PTFE septum cap before being heated to 130 °C while stirring at 1000 RPM. After 14 hours, the reaction mixture was cooled to room temperature before being diluted with 5-mL of H_2O and transferred to a separatory funnel. The aqueous layer was extracted with EtOAc (5 × 15 mL) and the organic extractions were collected and washed with brine. The organics were dried over Na_2SO_4, filtered, and evaporated under reduced pressure to afford the crude reaction mixture. The crude reaction mixture was dissolved in $CDCl_3$ before adding benzotrifluoride (24.6 µL, 0.2 mmol) as an internal standard. The crude reaction mixture was then submitted for ^1H and ^{19}F NMR analysis.

All yields for optimization reactions were determined by ^{19}F NMR with benzotrifluoride as an internal standard.

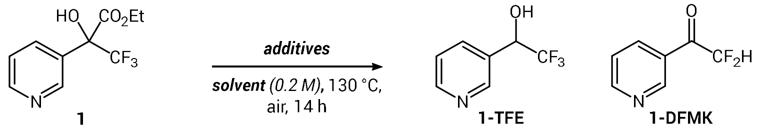

Entry	Additive(s)	Solvent	% Yield 1-TFE	%Yield 1-DFMK
1	H_2O (3.0 equiv), LiCl (3.0 equiv)	DMSO	24	9
2	H_2O (3.0 equiv), LiCl (3.0 equiv)	DMF	25	12
3	*none*	DMF	15	2
4	H_2O (3.0 equiv), NaCl (3.0 equiv)	DMSO	35	12
5	H_2O (3.0 equiv), KCl (3.0 equiv)	DMSO	30	17
6	H_2O (3.0 equiv), KCN (3.0 equiv)	DMSO	14	6
7	AcOH (3.0 equiv), LiCl (3.0 equiv)	DMSO	21	12
8	LiCl (3.0 equiv)	DMSO	20	15

Figure S1. Evaluation of solvents and additives

Procedure for Optimization Studies 2

To an oven-dried 8 mL vial and stir bar was added α,α-trifluoromethyl(hydroxy) ester **1** (49.8 mg 0.2 mmol, 1.0 equiv), *tert*-butyldimethylsilyl chloride (60.3 mg, 0.4 mmol, 2.0 equiv), imidazole (27.2 mg, 0.4 mmol, 2.0 equiv) and DMF (1.0 mL, 0.2 M). All reagents were added open to air and no further considerations were made to purge the solution. The vial was tightly sealed with a screw-on PTFE septum cap before being heated to 130 °C while stirring at 1000 RPM. After 14 hours, the reaction mixture was cooled to room temperature before adding TBAF (0.6 mL, 3.0 equiv, 1.0 M in THF) at room temperature. The reaction mixture was stirred for 30 minutes at room temperature before being diluted with 5 mL of water and transferred to a separatory funnel. The aqueous layer was extracted with EtOAc (5 × 15 mL) and the organic extractions were collected and washed with brine. The organics were dried over Na_2SO_4, filtered, and concentrated under reduced pressure to afford the crude reaction mixture. The crude reaction mixture was dissolved in $CDCl_3$ before adding benzotrifluoride (24.6 μL, 0.2 mmol) as an internal standard. The crude reaction mixture was then submitted for ^{1}H and ^{19}F NMR analysis.

All yields for optimization reactions were determined by ^{19}F NMR with benzotrifluoride as an internal standard.

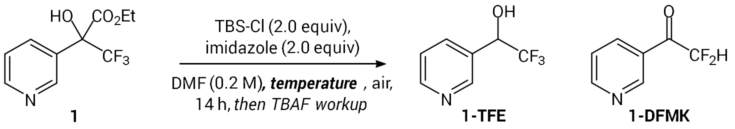

Entry	Temperature	% Yield 1-TFE	%Yield 1-DFMK
1	130 °C	98 **(95)**[a]	0
2	23 °C	0	0
3	40 °C	0	0
4	80 °C	0	0

Figure S2. Temperature evaluation of thermal decarboxylation. [a]Isolated yield.

Control Reactions

Control reactions were set up according to **Procedure for Optimization Studies 2** with the exclusion of the indicated reagents.

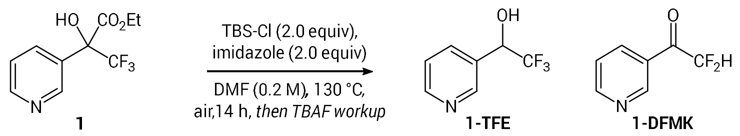

Entry	Conditions	% Yield 1-TFE	%Yield 1-DFMK
1	*no change*	98 **(95)**[a]	0
2	no TBS-Cl	55	7
3	no imidazole	76	3
4	no TBS-Cl, no imidazole	15	2
5	no TBS-Cl, 2 equiv TMS-Cl	99	0

Figure S3. Thermal silylation-decarboxylation control reactions. [a]Isolated yield.

Procedure for Optimization Studies 3

To an 8 mL vial with a stir bar was added α,α-trifluoromethyl(hydroxy) ester **1** (49.8 mg, 0.2 mmol, 1.0 equiv) and THF:H_2O (2.5 mL of THF and 2.5 mL of H_2O: 0.1 M). To this solution was added NaOH (20.0 mg, 0.5 mmol, 1.0 equiv) and iron (III) acetylacetonate (17.6 mg, 0.05 mmol, 10 mol%). The reaction mixture was then sealed with a screw-on PTFE septum cap and then sparged with a balloon of O_2 for 10 minutes, while stirring at room temperature. After removing the O_2 balloon, the reaction vial was then sealed with Parafilm and electrical tape. The vial was then irradiated with two 34W Gen 1 PR160L Kessil 390 nm lamps (1 cm away) for 16 hours without the presence of a cooling fan. After 16 hours, the reaction mixture was allowed to cool to room temperature before being diluted with H_2O (5 mL) and DCM (5 mL). The resulting aqueous layer was extracted with DCM (4 × 10 mL). The collected organics were dried over Na_2SO_4 and filtered, then dry loaded onto celite. The celite was then eluted with DCM and the filtrate evaporated to afford the crude reaction mixture. The crude reaction mixture was dissolved in $CDCl_3$ before adding benzotrifluoride (24.6 µL, 0.2 mmol) as an internal standard. The crude reaction mixture was then submitted for ^{1}H and ^{19}F NMR analysis.

All yields for optimization reactions were determined by ^{19}F NMR with benzotrifluoride as an internal standard.

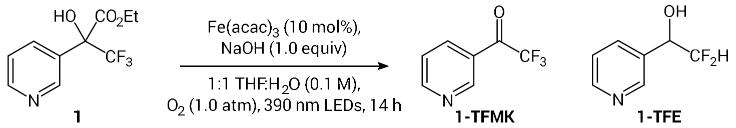

Entry	Conditions	% Yield 1-TFMK	%Yield 1-TFE
1	*no change*	75 **(73)**[a]	0
2	no NaOH,<br>3 equiv LiCl	6	0
3	no NaOH	0	0

Figure S4. Aerobic LMCT-mediated decarboxylation control reactions. [a]Isolated yield.

Preparation of Starting Materials

1-(4-(trifluoromethyl)phenyl)-1*H*-1,2,4-triazole (16-H)

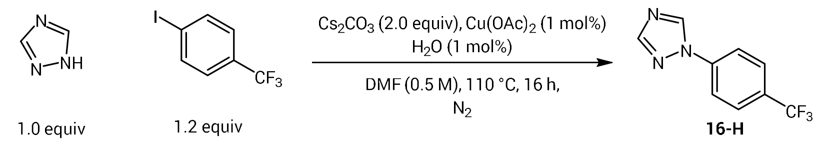

Prepared from a modified literature procedure.[1] To an oven-dried 150 mL Schlenk flask, added 1*H*-1,2,4-triazole (540.0 mg, 7.82 mmol, 1.0 equiv), 1-iodo-4-(trifluoromethyl)benzene (1.38 mL, 9.37 mmol, 2.55 g, 1.2 equiv), Cs_2CO_3 (6.11 g, 18.8 mmol, 2.0 equiv), copper(II) acetate (14.2 mg, 0.078 mmol, 0.01 equiv), H_2O (1.4 µL, 0.078 mmol, 0.01 equiv), and DMF (15.6 mL, 0.5 M). The flask was purged with nitrogen, sealed, and stirred at 110 °C for 16 hours. After cooling to room temperature, the reaction mixture was diluted with H_2O (80 mL) and extracted with EtOAc (4 × 40 mL). The combined organic extractions were washed with 5% aqueous LiCl, washed with brine, dried over Na_2SO_4, filtered, and concentrated under reduced pressure to afford the crude reaction mixture. The crude material was purified with flash column chromatography (40% EtOAc in hexanes) to afford **16-H** (1.38 g in 83% yield) as a white solid. The NMR spectra of the purified product were consistent with literature spectra.[2]

^{1}H NMR (400 MHz, $CDCl_3$) *δ* 8.64 (s, 1H), 8.15 (s, 1H), 7.85 (d, J = 9.3 Hz, 2H), 7.79 (d, J = 8.7 Hz, 2H)

^{13}C NMR (126 MHz, $CDCl_3$) *δ* 153.3, 141.1, 139.6, 130.3 (q, *J* = 33.3 Hz), 127.3 (q, *J* = 3.7 Hz), 123.7 (q, *J* = 272.2 Hz), 120.0

^{19}F NMR (471 MHz, $CDCl_3$) *δ* –62.6 (s, 3F)

2-(thiophen-2-yl)pyridine (17-H)

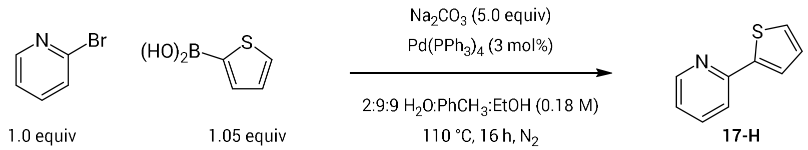

Prepared from a literature procedure.[3] To an oven-dried 150 mL Schlenk flask, was added 2-bromopyridine (500.0 mg, 3.6 mmol, 1.0 equiv), 2-thienylboronic acid (430.0 mg, 3.32 mmol, 1.05 equiv), Na_2CO_3 (1.7g, 16 mmol, 5.0 equiv), and $Pd(PPh_3)_4$ (110.0 mg, 0.095 mmol, 0.03 equiv). The flask was purged with nitrogen for 15 minutes, then charged with 20 mL of a mixture of H_2O:$PhCH_3$:EtOH ([2 mL H_2O, 9 mL $PhCH_3$, 9 mL EtOH], 0.18 M). The flask was sealed with a PTFE screw cap before being heated to 110 °C with stirring. After 16 hours, the reaction mixture was allowed to cool to room temperature before being diluted with saturated NH_4Cl (15 mL) and extracted with EtOAc (3 × 30 mL). The combined organic extractions were washed with brine, dried over Na_2SO_4, filtered, and concentrated under reduced pressure to afford the crude reaction mixture. The crude material was purified with flash column chromatography (gradient of 20 to 45% EtOAc in hexanes) to afford **17-H** (231.8 mg, 40% yield) as a white solid. The NMR spectra of the purified product were consistent with literature spectra.[4]

^{1}H NMR (500 MHz, $CDCl_3$) δ 8.59–8.56 (m, 1H), 7.73–7.63 (m, 2H), 7.60 (d, J = 3.6 Hz, 1H), 7.40 (dd, J = 5.0, 1.1 Hz, 1H), 7.15 (ddd, J = 6.7, 4.9, 1.6 Hz, 1H), 7.12 (dd, J = 5.0, 3.7 Hz, 1H)

2-(4-(trifluoromethyl)phenyl)thiophene (18-H)

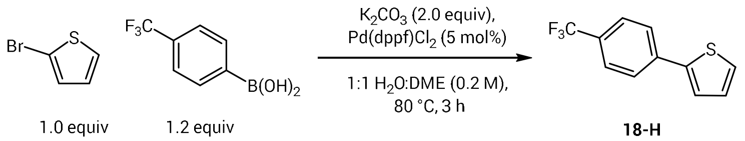

Prepared from a modified literature procedure.[5] To an oven-dried 100 mL round-bottom flask was added 4-trifluoromethylphenylboronic acid (1.39 g, 7.36 mmol, 1.2 equiv), 2-bromothiophene (0.594 mL, 6.13 mmol, 1.0 equiv), [1,1'-Bis(diphenylphosphino)ferrocene]palladium(II) dichloride (250.0 mg, 0.31 mmol, 0.05 equiv), K_2CO_3 (1.6 g, 12.3 mmol, 2.0 equiv), and 1:1 H_2O:dimethoxyethane (15 mL H_2O and 15 mL dimethoxyethane, 0.2 M). The flask was capped with a rubber septum, purged with nitrogen for 15 minutes, and stirred at 80 °C. After 3 hours, the reaction mixture was allowed to cool to room temperature before being diluted with H_2O (15 mL) and extracted with Et_2O (3 × 30 mL). The combined organic extractions were washed with brine, dried over Na_2SO_4, filtered, and concentrated under reduced pressure to afford the crude reaction mixture. The crude material was purified with flash column chromatography (100% hexanes) to afford **18-H** (1.27 g, 91% yield) as a white solid. The NMR spectra of the purified product were consistent with literature spectra.[6]

^{1}H NMR (500 MHz, $CDCl_3$) δ 7.71 (d, J = 8.0 Hz, 1H), 7.63 (d, J = 9.7 Hz, 1H), 7.40 (dd, J = 3.6, 1.1 Hz, 1H), 7.36 (dd, J = 5.1, 1.2 Hz, 1H), 7.12 (dd, J = 5.1, 3.6 Hz, 1H)

^{19}F NMR (471 MHz, $CDCl_3$) δ –62.5 (s, 3F)

IR (ATR-FTIR) υ_{max} (cm^{-1}) 1612, 1427, 1325, 1128, 1067, 706

mp: 109–111 °C (lit.[4] mp 114 °C)

Preparation of Substrates

General Procedure A: Preparation of α,α-trifluoromethyl(hydroxy) Esters

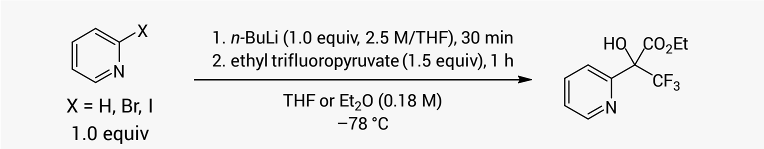

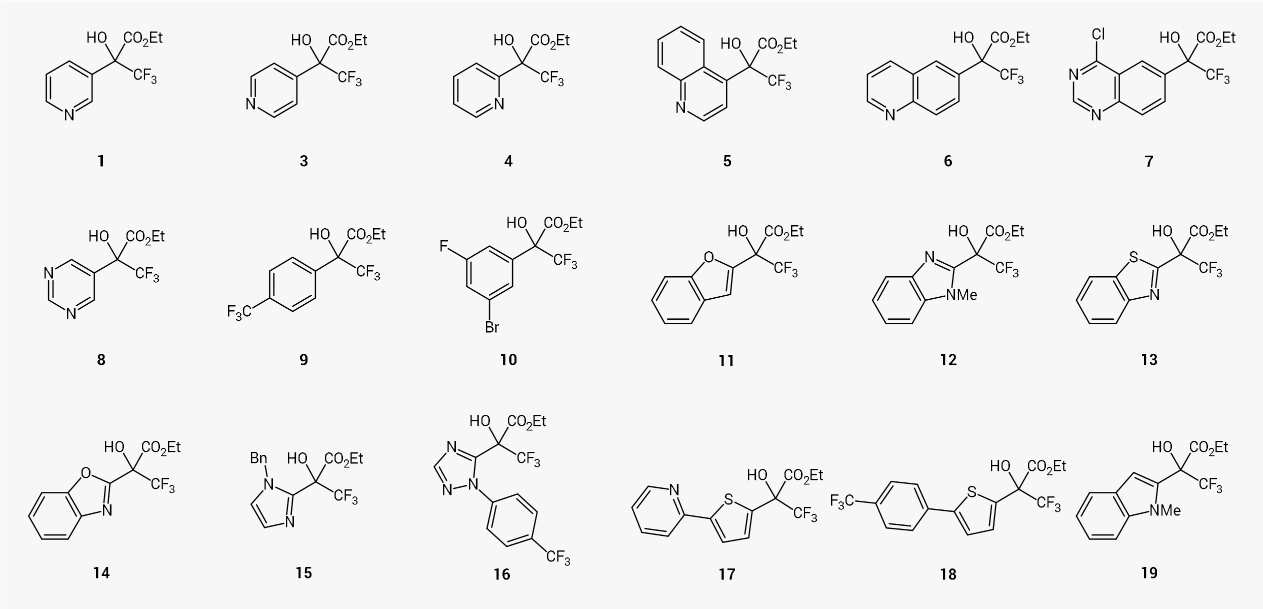

To a flame-dried 100 mL round-bottom flask with a stir bar was added arene (5 mmol, 1.0 equiv) and diethyl ether (28 mL, 0.18 M) or THF (28 mL, 0.18 M). The flask was capped with a rubber septum, cooled to –78 °C (in a dry ice-acetone bath), and purged with a constant flow of nitrogen for 15 minutes. To the flask was added *n*-butyllithium (2.0 mL, 2.5 M solution/hexanes, 5 mmol, 1.0 equiv) dropwise. The resulting solution was allowed to stir at –78 °C under nitrogen atmosphere for 30 minutes. To the flask was then added ethyl trifluoropyruvate (1.27 g was dissolved in 5 mL of THF, 7.5 mmol, 1.5 equiv) in one portion. The reaction was stirred for an additional 1 hour while warming to room temperature. The reaction was quenched with saturated aqueous NH_4Cl (25 mL) and diluted with EtOAc (25 mL). The aqueous layer was extracted with EtOAc (4 × 20 mL) and the collected organics were washed with brine, dried over Na_2SO_4, filtered, and concentrated under reduced pressure to afford the crude reaction mixture. The crude material was then dry loaded onto silica gel for purification by flash column chromatography. *Note: in the event there is residual ethyl trifluoropyruvate after purification, the product can be heated (with a heat gun, at approximately 80 °C) under high vacuum for about 10 minutes.*

ethyl 3,3,3-trifluoro-2-hydroxy-2-(pyridin-3-yl)propanoate (1)

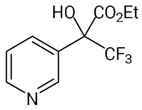

1

Prepared from 3-bromopyridine using a modified version of general procedure A. The addition of *n*-butyllithium was conducted at –50 °C in Et_2O and the reaction was then stirred for 15 minutes. Afterward, the reaction was cooled to –78 °C (dry ice-acetone bath) and anhydrous THF (28 mL) was added. The general procedure was then followed for the rest of the reaction. The crude material was purified by flash column chromatography (gradient of 30 to 50% EtOAc in hexanes) to afford **1** (1.03 g, 83% yield) as an off-white solid.

^{1}H NMR (500 MHz, $CDCl_3$) δ 9.03 (d, J = 2.2 Hz, 1H), 8.64 (dd, J = 4.8, 1.6 Hz, 1H), 8.14 (d, J = 8.2 Hz, 1H), 7.36 (ddd, J = 8.2, 4.8, 0.8 Hz, 1H), 5.29 (brs, 1H), 4.46 (dq, J = 10.7, 7.1 Hz, 1H), 4.38 (dq, J = 10.7, 7.1 Hz, 1H), 1.36 (t, J = 7.1 Hz, 3H)

^{13}C NMR (126 MHz, $CDCl_3$) δ 168.2, 150.5, 148.3 (q, J = 2.2 Hz), 135.13, 129.3, 123.3, 123.0 (q, J = 286.1 Hz), 76.9 (q, J = 30.8 Hz), 64.9, 14.0

^{19}F NMR (471 MHz, $CDCl_3$) δ –76.7 (s, 3F)

HRMS AMM (ESI–TOF) m/z calculated for $C_{10}H_{11}F_3NO_3^+$ $[M+H]^+$ 250.0686, found 250.0681

IR (ATR-FTIR) υ_{max} (cm^{-1}) 2996 br, 1739, 1284, 1184, 1172, 1153, 1015, 807, 724

mp: 72–75 °C

ethyl 3,3,3-trifluoro-2-hydroxy-2-(pyridin-4-yl)propanoate (3)

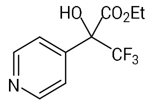

3

Prepared from 4-bromopyridine using general procedure A. The crude material was purified by flash column chromatography (100% DCM) to afford **3** (834.1 mg, 67% yield) as an orange solid.

^{1}H NMR (500 MHz, CDCl$_3$) δ 8.68–8.64 (m, 2H), 7.74 (d, J = 5.5 Hz, 2H), 4.46 (dq, J = 10.8, 7.1 Hz, 1H), 4.38 (dq, J = 10.7, 7.1 Hz, 1H), 1.36 (t, J = 7.1 Hz, 3H)

^{13}C NMR (126 MHz, CDCl$_3$) δ 167.8, 149.8, 142.2, 122.7 (q, J = 286.9 Hz), 121.8, 77.6, 77.5 (q, J = 35.7 Hz), 64.9, 13.9

^{19}F NMR (471 MHz, CDCl$_3$) δ –76.2 (s, 3F)

HRMS AMM (ESI–TOF) m/z calculated for $C_{10}H_{11}F_3NO_3^+$ [M+H]$^+$ 250.0686, found 250.0680

IR (ATR-FTIR) υ_{max} (cm^{-1}) 2987 br, 1744, 1268, 1169, 1134, 1028, 805, 717, 680

mp: 70–75 °C

ethyl 3,3,3-trifluoro-2-hydroxy-2-(pyridin-2-yl)propanoate (4)

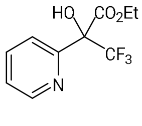

4

Prepared from 2-bromopyridine using general procedure A. The crude material was purified by flash column chromatography (100% DCM) to afford **4** (797.0 mg, 64% yield) as a pale yellow oil.

^{1}H NMR (400 MHz, CDCl$_3$) δ 8.57 (dt, J = 4.9, 1.4 Hz, 1H), 7.86 – 7.77 (m, 2H), 7.41 (ddd, J = 6.2, 4.9, 2.4 Hz, 1H), 6.53 (bs, 1H), 4.28 (qd, J = 7.1, 3.1 Hz, 2H), 1.25 (t, J = 7.1 Hz, 3H)

^{13}C NMR (126 MHz, CDCl$_3$) δ 167.0, 149.2, 147.6, 137.9, 125.1, 123.0 (q, J = 286.1 Hz), 122.5 (q, J = 2.2 Hz), 77.6 (q, J = 29.6 Hz), 63.1, 13.8

^{19}F NMR (376 MHz, CDCl$_3$) δ –76.2 (s, 3F)

HRMS AMM (ESI–TOF) m/z calculated for $C_{10}H_{11}F_3NO_3^+$ [M+H]$^+$ 250.0686, found 250.0694

IR (ATR-FTIR) υ_{max} (cm^{-1}) 3253 br, 2986, 1743, 1246, 1173, 1134, 1027, 756, 718

ethyl 3,3,3-trifluoro-2-hydroxy-2-(quinolin-4-yl)propanoate (5)

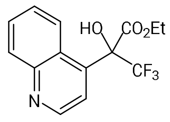

5

Prepared from 4-bromoquinoline using general procedure A. crude material was purified by flash column chromatography (gradient of 20 to 35% EtOAc in hexanes) to afford **5** (1.22 g, 82% yield) as a yellow solid.

^{1}H NMR (400 MHz, $CDCl_3$) δ 8.92 (d, J = 4.8 Hz, 1H), 8.16 (td, J = 8.5, 0.8 Hz, 2H), 7.73 (ddd, J = 8.4, 6.9, 1.3 Hz, 1H), 7.65 (dd, J = 4.8, 1.9 Hz, 1H), 7.56 (ddd, J = 8.4, 6.8, 1.4 Hz, 1H), 4.36 (dq, J = 10.7, 7.1 Hz, 1H), 4.23 (dq, J = 10.7, 7.1 Hz, 1H), 1.11 (t, J = 7.1 Hz, 3H)

^{13}C NMR (101 MHz, $CDCl_3$) δ 169.3, 149.5, 149.1, 138.1, 130.7, 129.6, 127.6, 125.8, 124.4, 120.1 (q, J = 3.2 Hz), 79.5 (q, J = 29.5 Hz), 64.8, 13.8

^{19}F NMR (376 MHz, $CDCl_3$) δ –73.2 (s, 3F)

HRMS AMM (ESI–TOF) m/z calculated for $C_{14}H_{13}F_3NO_3^+$ $[M+H]^+$ 300.0842, found 300.0842

IR (ATR-FTIR) υ_{max} (cm^{-1}) 2982 br, 1738, 1255, 1181, 1159, 1136, 1057, 1022, 759

mp: 127–138 °C

ethyl 3,3,3-trifluoro-2-hydroxy-2-(quinolin-6-yl)propanoate (6)

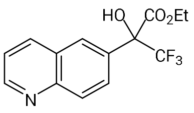

6

Prepared from 6-bromoquinoline using general procedure A. The crude material was purified by flash column chromatography (gradient of 20 to 30% [3:1 EtOAc:EtOH mixture] in hexanes) to afford **6** (1.05 g, 70% yield) as an off-white solid.

^{1}H NMR (400 MHz, CDCl$_3$) δ 8.95 (dd, J = 4.3, 1.7 Hz, 1H), 8.33 (d, J = 2.3 Hz, 1H), 8.22 (dd, J = 8.3, 1.7 Hz, 1H), 8.18–8.10 (m, 2H), 7.45 (dd, J = 8.3, 4.3 Hz, 1H), 5.13 (brs, 1H), 4.48 (dq, J = 9.5, 6.5 Hz, 1H), 4.40 (dq, J = 10.6, 6.5 Hz, 1H), 1.37 (t, J = 7.2 Hz, 3H)

^{13}C NMR (101 MHz, CDCl$_3$) δ 168.7, 151.6, 148.3, 137.1, 131.4, 129.6, 127.62, 127.59, 127.0, 123.1 (q, J = 286.0 Hz), 121.7, 77.9 (q, J = 30.2 Hz), 64.7, 14.1

^{19}F NMR (376 MHz, CDCl$_3$) δ –76.0 (s, 3F)

HRMS AMM (ESI–TOF) m/z calculated for $C_{14}H_{13}F_3NO_3^+$ $[M+H]^+$ 300.0803, found 300.0844

IR (ATR-FTIR) υ_{max} (cm^{-1}) 3020 br, 1748, 1242, 1153, 1129, 1107, 840, 768

mp: 156–161 °C

ethyl 2-(4-chloroquinazolin-6-yl)-3,3,3-trifluoro-2-hydroxypropanoate (7)

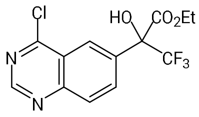

7

Prepared from 6-bromo-4-chloroquinazoline using general procedure A. The crude material was purified by flash column chromatography (gradient of 20 to 30% [3:1 EtOAc:EtOH mixture] in hexanes) to afford **7** (952.0 mg, 57% yield) as a white solid.

^{1}H NMR (500 MHz, $CDCl_3$) δ 9.10 (s, 1H), 8.80 (d, J = 2.0 Hz, 1H), 8.42 (dd, J = 9.0, 1.2 Hz, 1H), 8.13 (d, J = 9.0 Hz, 1H), 4.64 (s, 1H), 4.52 (dq, J = 10.7, 7.1 Hz, 1H), 4.48 (dq, J = 10.7, 7.1 Hz, 1H), 1.44 (t, J = 7.2 Hz, 3H)

^{13}C NMR (126 MHz, $CDCl_3$) δ 168.1, 163.4, 154.8, 151.5, 134.0, 133.3, 129.2, 125.4, 123.9 (q, J = 289.5 Hz), 123.7, 77.6 (q, J = 30.8 Hz), 65.3, 14.1

^{19}F NMR (471 MHz, $CDCl_3$) δ –76.2 (s, 3F)

HRMS AMM (ESI–TOF) m/z calculated for $C_{13}H_{12}F_3N_2O_4^+$ $[M–Cl+H_2O]^+$ 317.0744, found 317.0747

IR (ATR-FTIR) υ_{max} (cm^{-1}) 2960 br, 1748, 1564, 1551, 1236, 1180, 1154, 1126, 1015, 996, 841, 750

mp: 163–166 °C

ethyl 3,3,3-trifluoro-2-hydroxy-2-(pyrimidin-5-yl)propanoate (8)

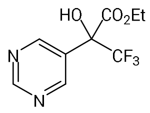

8

Prepared from 5-bromopyrimidine using general procedure A. The crude material was purified by flash column chromatography (30% in EtOAc in hexanes) to afford **8** (412.5 mg, 33% yield) as an orange oil.

^{1}H NMR (500 MHz, $CDCl_3$) δ 9.27 (s, 1H), 9.17 (s, 2H), 4.78 (brs, 1H), 4.52 (dq, J = 10.7, 7.2 Hz, 1H), 4.43 (dq, J = 10.7, 7.2 Hz, 1H), 1.40 (t, J = 7.2 Hz, 3H)

^{13}C NMR (126 MHz, $CDCl_3$) δ 167.5, 159.4, 156.0, 127.1, δ 122.5 (q, J = 286.6 Hz), 75.8 (q, J = 31.6 Hz), 65.6, 14.0

^{19}F NMR (471 MHz, $CDCl_3$) δ –77.2 (s, 3F)

HRMS AMM (ESI–TOF) m/z calculated for $C_9H_{10}F_3N_2O_3^+$ $[M+H]^+$ 251.0638, found 251.0643

IR (ATR-FTIR) υ_{max} (cm^{-1}) 2987 br, 1749, 1559, 1417, 1243, 1178, 1136, 1105, 1015, 948, 737

ethyl 3,3,3-trifluoro-2-hydroxy-2-(4-(trifluoromethyl)phenyl)propanoate (9)

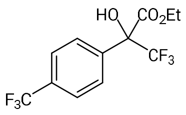

9

Prepared from 1-iodo-4-(trifluoromethyl)benzene using general procedure A. The crude material was purified by flash column chromatography (5% EtOAc in hexanes) to afford **9** (1.12 g, 71% yield) as a colorless oil.

^{1}H NMR (500 MHz, $CDCl_3$) δ 7.97 (d, J = 7.5 Hz, 2H), 7.68 (d, J = 9.8 Hz, 2H), 4.48 (dq, J = 10.7, 7.1 Hz, 1H), 4.48 (bs, 1H), 4.41 (dq, J = 10.7, 7.1 Hz, 1H), 1.39 (t, J = 7.1 Hz, 3H)

^{13}C NMR (126 MHz, $CDCl_3$) δ 168.4, 136.7, 131.6 (q, J = 32.7 Hz), 127.5 (q, J = 1.4 Hz), 125.3 (q, J = 3.8 Hz), 123.8 (q, J = 272.1 Hz), 122.7 (q, J = 285.9 Hz), 77.5 (q, J = 30.6 Hz), 65.1, 14.0

^{19}F NMR (471 MHz, $CDCl_3$) δ –63.0 (s, 3F), –76.4 (s, 3F)

HRMS AMM (ESI–TOF) m/z calculated for $C_{12}H_9F_6O_3^-$ $[M–H]^-$ 315.0461, found 315.0457

IR (ATR-FTIR) υ_{max} (cm^{-1}) 2990 br, 1739, 1324, 1163, 1125, 1102, 1069, 1018, 847, 707

ethyl 2-(3-bromo-5-fluorophenyl)-3,3,3-trifluoro-2-hydroxypropanoate (10)

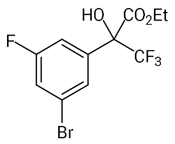

10

Prepared from 1,3-dibromo-5-fluorobenzene using general procedure A. The crude material was purified by flash column chromatography (10% DCM in hexanes) to afford **10** (1.21 g, 70% yield) as a white solid.

^{1}H NMR (500 MHz, $CDCl_3$) δ 7.78 (m, 1H), 7.51 (m, 1H), 7.31 (ddd, J = 7.8, 2.4, 1.7 Hz, 1H), 4.49 (dq, J = 10.7, 7.2 Hz, 1H), 4.43 (s, 1H), 4.42 (dq, J = 10.7, 7.1 Hz, 1H), 4.43 (brs, 1H), 1.40 (t, J = 7.2 Hz, 3H)

^{13}C NMR (126 MHz, $CDCl_3$) δ 168.0, 162.5, 136.8 (d, J = 8.2 Hz), 126.2, 122.8 (d, J = 9.7 Hz), 122.5 (q, J = 286.0 Hz), 120.6, 120.4, 113.8 (q, J = 1.6 Hz), 113.6 (q, J = 1.6 Hz), 65.2, 14.0

^{19}F NMR (coupled, 471 MHz, $CDCl_3$) δ –76.5 (s, 3F), –109.6 (dd, J = 10.3, 7.9 Hz, 1F)

HRMS AMM (ESI–TOF) m/z calculated for $C_{11}H_8BrF_4O_3^-$ [M–H]$^-$ 342.9598, found 342.9580

IR (ATR-FTIR) υ_{max} (cm^{-1}) 3463 br, 3105, 2918, 1732, 1579, 1302, 1179, 1149, 1129, 983, 862, 740

mp: 37–40 °C

ethyl 2-(benzofuran-2-yl)-3,3,3-trifluoro-2-hydroxypropanoate (11)

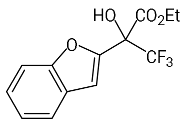

11

Prepared from benzofuran using general procedure A. The crude material was purified by flash column chromatography (15% EtOAc in hexanes) to afford **11** (1.28 g, 89% yield) as a pale yellow oil.

^{1}H NMR (400 MHz, CDCl$_3$) δ 7.61 (ddd, J = 7.7, 1.4, 0.7 Hz, 1H), 7.51 (dq, J = 8.4, 1.0 Hz, 1H), 7.35 (ddd, J = 8.3, 7.2, 1.4 Hz, 1H), 7.27 (td, J = 7.5, 1.0 Hz, 1H), 7.02 (t, J = 0.8 Hz, 1H), 4.54–4.46 (dq, J = 10.7, 7.1 Hz, 1H), 4.52 (brs, 1H), 4.46–4.37 (dq, J = 10.7, 7.1 Hz, 1H), 1.36 (t, J = 7.1 Hz, 3H)

^{13}C NMR (101 MHz, CDCl$_3$) δ 167.2, 155.3, 148.4, 127.3, 125.7, 123.5, 122.4 (q, J = 286.1 Hz), 121.9, 111.8, 107.5, 75.6 (q, J = 32.1 Hz), 65.1, 14.0

^{19}F NMR (376 MHz, CDCl$_3$) δ –75.8 (s, 3F)

HRMS AMM (ESI–TOF) m/z calculated for $C_{13}H_{12}F_3O_4^+$ $[M+H]^+$ 289.0682, found 289.0690

IR (ATR-FTIR) υ_{max} (cm^{-1}) 3465 br, 1741, 1224, 1186, 1153, 1140, 1011, 984, 746

ethyl 3,3,3-trifluoro-2-hydroxy-2-(1-methyl-1*H*-benzo[*d*]imidazol-2-yl)propanoate (12)

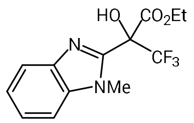

12

Prepared from *N*-methylbenzimidazole using general procedure A. The crude material was purified by flash column chromatography (30% EtOAc in hexanes) to afford **12** (1.35 g, 89% yield) as a pale yellow oil.

^{1}H NMR (400 MHz, $CDCl_3$) δ 7.82 (dt, J = 7.9, 1.1 Hz, 1H), 7.41–7.36 (m, 2H), 7.35–7.29 (m, 1H), 5.21 (brs, 1H), 4.52 (dq, J = 10.7, 7.1 Hz, 1H), 4.43 (dq, J = 10.7, 7.1 Hz, 1H), 3.87 (s, 3H), 1.36 (t, J = 7.1 Hz, 3H)

^{13}C NMR (101 MHz, $CDCl_3$) δ 166.9, 145.2, 141.2, 137.1, 124.2, 123.0, 122.6 (q, J = 286.1 Hz), 120.9, 109.8, 77.5 (q, J = 31.6 Hz), 64.8, 31.5 (q, J = 2.0 Hz), 14.0

^{19}F NMR (376 MHz, $CDCl_3$) δ –74.4 (s, 3F)

HRMS AMM (ESI–TOF) m/z calculated for $C_{13}H_{14}F_3N_2O_3^+$ $[M+H]^+$ 303.0951, found 303.0957

IR (ATR-FTIR) υ_{max} (cm^{-1}) 2984 br, 1748, 1473, 1237, 1199, 1164, 1124, 1016, 966, 744, 734

ethyl 2-(benzo[*d*]thiazol-2-yl)-3,3,3-trifluoro-2-hydroxypropanoate (13)

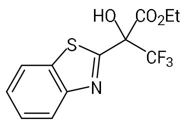

13

Prepared from 1,3-benzothiazole using general procedure A. The crude material was purified by flash column chromatography (10% EtOAc in hexanes) to afford **13** (960 mg, 63% yield) as a yellow oil.

^{1}H NMR (500 MHz, $CDCl_3$) δ 8.13 (dt, J = 8.2, 0.9 Hz, 1H), 7.94 (dt, J = 8.1, 1.0 Hz, 1H), 7.54 (ddd, J = 8.4, 7.2, 1.3 Hz, 1H), 7.48 (ddd, J = 8.3, 7.2, 1.2 Hz, 1H), 5.20 (brs, 1H), 4.51 (dq, J = 10.7, 7.1 Hz, 1H), 4.46 (dq, J = 10.7, 7.1 Hz, 1H), 1.40 (t, J = 7.1 Hz, 3H)

^{13}C NMR (101 MHz, $CDCl_3$) δ 166.2, 163.0, 152.4, 136.0, 126.4, 126.3, 124.2, 122.0 (q, J = 286.5 Hz), 121.8, 78.3 (q, J = 31.5 Hz), 65.1, 14.0

^{19}F NMR (471 MHz, $CDCl_3$) δ –75.9 (s, 3F)

HRMS AMM (ESI–TOF) m/z calculated for $C_{12}H_{11}F_3NO_3S^+$ $[M+H]^+$ 306.0406, found 306.0412

IR (ATR-FTIR) υ_{max} (cm^{-1}) 3365 br, 2966, 1761, 1452, 1288, 1183, 1169, 1148, 1123, 981, 742

ethyl 2-(benzo[*d*]oxazol-2-yl)-3,3,3-trifluoro-2-hydroxypropanoate (14)

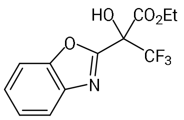

14

Prepared from 1,3-benzoxazole using general procedure A. The crude material was purified by flash column chromatography (20% EtOAc in hexanes) to afford **14** (1.42 g, 98% yield) as an off-white solid.

^{1}H NMR (500 MHz, $CDCl_3$) δ 7.82–7.80 (m, 1H), 7.61–7.55 (m, 1H), 7.44–7.40 (m, 1H), 7.38 (td, J = 7.6, 1.4 Hz, 1H), 5.32 (brs, 1H), 4.50 (dq, J = 10.7, 7.2 Hz, 1H), 4.43 (dq, J = 10.7, 7.1 Hz, 1H), 1.33 (t, J = 7.2 Hz, 1H)

^{13}C NMR (126 MHz, $CDCl_3$) δ 165.4, 157.7, 151.0, 139.9, 126.7, 125.3, 121.8 (q, J = 286.4 Hz), 121.1, 111.3, 75.7 (q, J = 32.4 Hz), 65.3, 13.8

^{19}F NMR (471 MHz, $CDCl_3$) δ –75.0 (s, 3F)

HRMS AMM (ESI–TOF) m/z calculated for $C_{12}H_{11}F_3NO_4^+$ $[M+H]^+$ 290.0635, found 290.0630

IR (ATR-FTIR) υ_{max} (cm^{-1}) 3445 br, 2986, 1751, 1227, 1136, 1017, 748, 668

mp: 124–128 °C

ethyl 2-(1-benzyl-1*H*-imidazol-2-yl)-3,3,3-trifluoro-2-hydroxypropanoate (15)

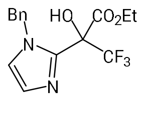

15

Prepared from *N*-benzylimidazole using general procedure A. The crude material was purified by flash column chromatography (1% MeOH in DCM) to afford **15** (1.25 g, 76% yield) as a white solid.

^{1}H NMR (500 MHz, $CDCl_3$) δ 7.36–7.29 (m, 3H), 7.09 (d, J = 1.2 Hz, 1H), 7.08–7.05 (m, 2H), 6.88 (d, J = 1.2 Hz, 1H), 5.34 (d, J = 15.8 Hz, 1H), 5.23 (d, J = 15.8 Hz, 1H), 4.85 (bs, 1H), 4.25 (dq, J = 10.7, 7.1 Hz, 1H), 4.07 (dq, J = 10.7, 7.1 Hz, 1H), 1.26 (t, J = 7.1 Hz, 3H)

^{13}C NMR (126 MHz, $CDCl_3$) δ 167.3, 139.0, 136.2, 129.0, 128.22, 128.15, 127.1, 123.7, 122.6 (q, J = 286.3 Hz), 76.4 (q, J = 31.2 Hz), 64.6, 50.7, 13.9

^{19}F NMR (471 MHz, $CDCl_3$) δ –75.0 (s, 3F)

HRMS AMM (ESI–TOF) m/z calculated for $C_{15}H_{16}F_3N_2O_3^+$ $[M+H]^+$ 329.1108, found 329.1107

IR (ATR-FTIR) υ_{max} (cm^{-1}) 3032 br, 1748, 1254, 1164, 1122, 1028, 711, 693

mp: 73–78 °C

ethyl 3,3,3-trifluoro-2-hydroxy-2-(4-(4-(trifluoromethyl)phenyl)-4*H*-1,2,4-triazol-3-yl)propanoate (16)

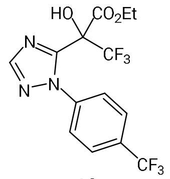

16

Prepared from 1-(4-(trifluoromethyl)phenyl)-1*H*-1,2,4-triazole using general procedure A. The crude material was purified by flash column chromatography (40% EtOAc in hexanes) to afford **16** (1.67 g, 87% yield) as a white solid.

^{1}H NMR (500 MHz, $CDCl_3$) δ 8.11 (s, 1H), 7.75 (d, *J* = 8.3 Hz, 2H), 7.56 (d, *J* = 8.2 Hz, 2H), 4.53 (s, 1H), 4.28 (dq, *J* = 10.7, 7.1 Hz, 1H), 4.08 (dq, *J* = 10.7, 7.1 Hz, 1H), 1.22 (t, *J* = 7.2 Hz, 3H)

^{13}C NMR (126 MHz, $CDCl_3$) δ 166.2, 151.5, 148.2, 140.3, 132.7 (q, *J* = 33.2 Hz), 127.4, 123.5 (q, *J* = 272.6 Hz), 126.6 (q, *J* = 3.7 Hz), 122.0 (q, *J* = 287.5 Hz), 75.3 (q, *J* = 32.0 Hz), 65.5, 13.7

^{19}F NMR (376 MHz, $CDCl_3$) δ –62.8 (s, 3F), –74.9 (s, 3F)

HRMS AMM (ESI–TOF) m/z calculated for $C_{14}H_{12}F_6N_3O_3^+$ $[M+H]^+$ 384.0777, found 384.0782

IR (ATR-FTIR) υ_{max} (cm^{-1}) 3064 br, 1729, 1321, 1210, 1160, 1126, 1070, 837, 709

mp: 129–135 °C

ethyl 3,3,3-trifluoro-2-hydroxy-2-(5-(pyridin-2-yl)thiophen-2-yl)propanoate (17)

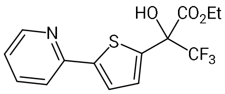

17

Prepared from 2-(thiophen-2-yl)pyridine using general procedure A. The crude material was purified by flash column chromatography (gradient of 10 to 30% EtOAc in hexanes) to afford **17** (850.0 mg, 51% yield) as a yellow solid.

^{1}H NMR (500 MHz, $CDCl_3$) δ 8.57 (ddd, J = 4.9, 1.8, 1.0 Hz, 1H), 7.69 (ddd, J = 8.0, 7.4, 1.8 Hz, 1H), 7.66–7.61 (m, 1H), 7.49 (d, J = 4.0 Hz, 1H), 7.17 (ddd, J = 7.3, 4.9, 1.2 Hz, 1H), 4.49 (dq, J = 10.7, 7.1 Hz, 1H), 4.42 (dq, J = 10.7, 7.1 Hz, 1H), 1.40 (t, J = 7.1 Hz, 3H)

^{13}C NMR (126 MHz, $CDCl_3$) δ 168.0, 152.1, 146.4, 137.6, 137.0, 128.6, 124.5, 122.5, 122.5 (q, J = 286.4 Hz), 118.9, 76.8 (q, J = 31.4 Hz), 65.0, 14.0

^{19}F NMR (471 MHz, $CDCl_3$) δ –77.7 (s, 3F)

HRMS AMM (ESI–TOF) m/z calculated for $C_{14}H_{13}F_3NO_3S^+$ $[M+H]^+$ 332.0563, found 332.0567

IR (ATR-FTIR) υ_{max} (cm^{-1}) 2977, 1745, 1249, 1162, 1138, 1022, 772, 740

mp: 107–112 °C

ethyl 3,3,3-trifluoro-2-hydroxy-2-(5-(4-(trifluoromethyl)phenyl)thiophen-2-yl)propanoate (18)

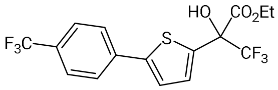

18

Prepared from 2-(4-(trifluoromethyl)phenyl)thiophene using general procedure A. The crude material was purified by flash column chromatography (10% EtOAc in hexanes) to afford **18** (1.31 g, 66% yield) as a yellow oil.

^{1}H NMR (500 MHz, $CDCl_3$) δ 7.68 (d, J = 8.2 Hz, 2H), 7.63 (d, J = 8.7 Hz, 2H), 7.39 (d, J = 3.2 Hz, 1H), 7.31 (d, J = 3.9 Hz, 1H), 4.67 (s, 1H), 4.50 (dq, J = 10.7, 7.2 Hz, 1H), 4.44 (dq, J = 10.7, 7.1 Hz, 1H), 1.42 (t, J = 7.1 Hz, 3H)

^{13}C NMR (126 MHz, $CDCl_3$) δ 167.9, 144.3, 137.2, 136.6, 129.9 (q, J = 32.7 Hz), 128.8, 126.1 (q, J = 3.6 Hz), 126.1, 124.7, 124.2 (q, J = 271.9 Hz), 122.5 (q, J = 286.2 Hz), 76.7 (q, J = 32.2 Hz), 65.2, 14.0

^{19}F NMR (471 MHz, $CDCl_3$) δ –62.6 (s, 3F), –77.9 (s, 3F)

HRMS AMM (ESI–TOF) m/z calculated for $C_{16}H_{11}F_6O_3S^-$ 397.0339 [M–H]$^-$, found 397.0336

IR (ATR-FTIR) υ_{max} (cm^{-1}) 3465 br, 2987, 1738, 1323, 1160, 1108, 1070, 1013, 839, 805

ethyl 3,3,3-trifluoro-2-hydroxy-2-(1-methyl-1*H*-indol-2-yl)propanoate (19)

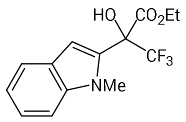

19

Prepared from 1-Methyl-1*H*-indole using a modified version of general procedure A. The addition of *n*-butyllithium was conducted at 0 °C and stirred for 1 hour, warming to room temperature. The reaction was then cooled to –78 °C, ethyl trifluoropyruvate was added, and the general procedure was followed as written. The crude material was purified by flash column chromatography (10% EtOAc in hexanes) to afford **19** (890.1 mg, 59% yield) as an off-white solid.

^{1}H NMR (400 MHz, CDCl$_3$) δ 7.64 (dt, J = 8.1, 1.0 Hz, 1H), 7.34 (dd, J = 8.3, 1.1 Hz, 1H), 7.29 (ddd, J = 8.3, 6.8, 1.2 Hz, 1H), 6.83–6.76 (m, 1H), 4.51 (dq, J = 10.7, 7.2 Hz, 1H), 4.40 (brs, 1H) 4.39 (dq, J = 10.7, 7.1 Hz, 1H), 3.79 (s, 3H), 1.35 (t, J = 7.1 Hz, 3H)

^{13}C NMR (101 MHz, CDCl$_3$) δ 168.8, 138.6, 129.5, 126.6, 123.3, 123.0 (q, J = 286.8 Hz), 121.5, 120.2, 109.5, 104.5 (q, J = 2.9 Hz), 77.6 (q, J = 31.1 Hz), 65.0, 31.4, 14.0

^{19}F NMR (376 MHz, CDCl$_3$) δ –74.8 (s, 3F)

HRMS AMM (ESI–TOF) m/z calculated for $C_{14}H_{15}F_3NO_3^+$ [M+H]$^+$ 302.0999, found 302.1002

IR (ATR-FTIR) υ_{max} (cm^{-1}) 3469, 1726, 1226, 1181, 1161, 1141, 1060, 845, 743

mp: 73–74 °C

General Procedure B: Decarboxylation of α,α-trifluoromethyl(hydroxy) Esters

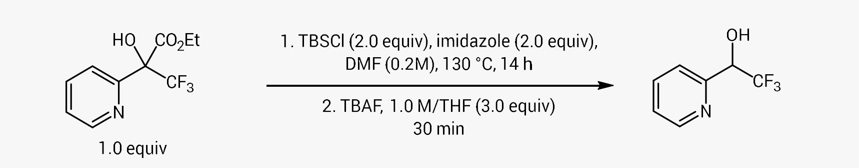

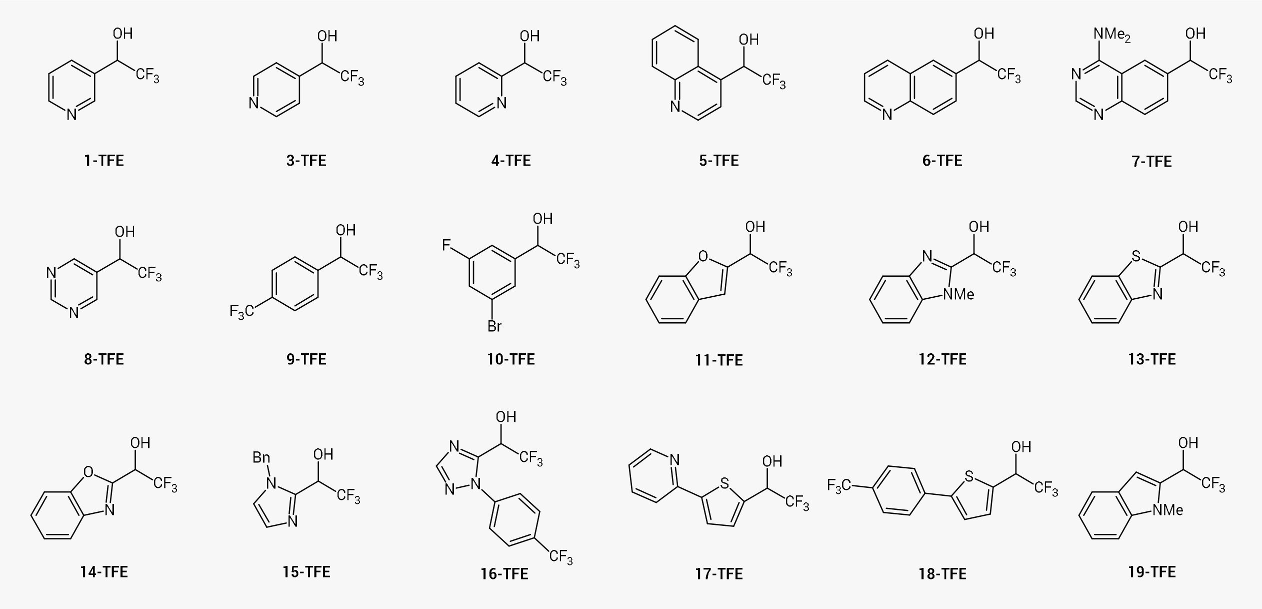

To an oven-dried 8 mL vial with a stir bar was added α,α-trifluoromethyl(hydroxy) ester (0.5 mmol, 1.0 equiv), imidazole (68.1 mg, 1.0 mmol, 2.0 equiv), *tert*-butyldimethylsilyl chloride (150.7 mg, 1.0 mmol, 2.0 equiv), and DMF (2.5 mL, 0.2 M). The vial was tightly sealed with a screw-on PTFE septum cap. All reagents were added open to air and no further considerations were made to purge the solution. The vial was then heated to 130 °C while stirring at 1000 RPM. After 14 hours, reaction mixture was allowed to cool. A solution of TBAF (1.5 mL, 3.0 equiv, 1.0 M/THF) was added to the reaction mixture at room temperature and allowed to stir for an additional 30 minutes, capped. The reaction mixture was diluted with H_2O (2 mL) and EtOAc (2 mL), then transferred to a separatory funnel. The aqueous layer was extracted with EtOAc (5 × 15 mL) and the organic extractions were collected and washed with brine. The organics were dried over Na_2SO_4, filtered, and concentrated under reduced pressure to afford the crude reaction mixture. The crude material was then dry loaded onto silica gel for purification by flash column chromatography.

2,2,2-trifluoro-1-(pyridin-3-yl)ethan-1-ol (1-TFE)

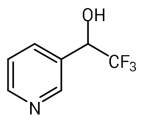

1-TFE

Prepared from **1** using general procedure B. The crude material was purified by flash column chromatography (50% EtOAc in hexanes) to afford **1-TFE** (84.1 mg, 95% yield) as a colorless oil. The NMR spectra of the purified product were consistent with literature spectra.[7]

^{1}H NMR (500 MHz, $CDCl_3$) δ 8.56 (s, 1H), 8.52 (dd, J = 4.9, 1.6 Hz, 1H), 7.94 (dt, J = 8.0, 1.9 Hz, 1H), 7.39 (dd, J = 8.0, 4.9 Hz, 1H), 5.08 (q, J = 6.7 Hz, 1H)

^{13}C NMR (126 MHz, $CDCl_3$) δ 149.8, 148.4, 136.2, 131.6, 124.4 (q, J = 282.5 Hz), 124.0, 70.5 (q, J = 32.3 Hz)

^{19}F NMR (coupled, 471 MHz, $CDCl_3$) δ –78.4 (d, J = 6.5 Hz, 3F)

2,2,2-trifluoro-1-(pyridin-4-yl)ethan-1-ol (3-TFE)

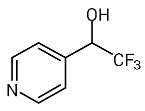

3-TFE

Prepared from **3** using general procedure B. The crude material was purified by flash column chromatography (45% EtOAc in hexanes) to afford of **3-TFE** (85.0 mg, 96% yield) as a yellow oil. The NMR spectra of the purified product were consistent with literature spectra.[8]

^{1}H NMR (500 MHz, $CDCl_3$) δ 8.59 (d, J = 6.2 Hz, 2H), 7.48 (d, J = 5.8 Hz, 2H), 5.07 (q, J = 6.6 Hz, 1H)

^{19}F NMR (coupled, 376 MHz, $CDCl_3$) δ –78.1 (d, J = 6.8 Hz, 3F)

2,2,2-trifluoro-1-(pyridin-2-yl)ethan-1-ol (4-TFE)

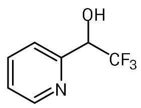

4-TFE

Prepared from **4** using general procedure B. The crude material was purified by flash column chromatography (2% MeOH in DCM) to afford **4-TFE** (83.2 mg, 94% yield) as a colorless oil. The NMR spectra of the purified product were consistent with literature spectra.[9]

^{1}H NMR (400 MHz, $CDCl_3$) δ 8.63 (dt, J = 4.8, 1.4 Hz, 1H), 7.79 (td, J = 7.7, 1.7 Hz, 1H), 7.43 (d, J = 7.9 Hz, 1H), 7.39 (ddd, J = 7.5, 4.8, 1.1 Hz, 1H), 5.30 (brs, 1H), 5.03 (q, J = 6.7 Hz, 1H)

^{13}C NMR (101 MHz, $CDCl_3$) δ 151.2 (q, J = 2.2 Hz), 148.4, 137.4, 124.6, 124.2 (q, J = 283.8 Hz), 122.8, 70.8 (q, J = 31.8 Hz)

^{19}F NMR (coupled, 376 MHz, $CDCl_3$) δ –78.1 (d, J = 6.8 Hz, 3F)

2,2,2-trifluoro-1-(quinolin-4-yl)ethan-1-ol (5-TFE)

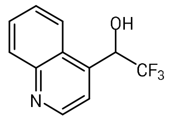

5-TFE

Prepared from **5** using general procedure B. The crude material was purified by flash column chromatography (5% MeOH in DCM) to afford 5-**TFE** (128.8 mg, 93% yield) as a white solid. The NMR spectra of the purified product were consistent with literature spectra.[14]

^{1}H NMR (500 MHz, DMSO-d$_6$) δ 9.00 (d, J = 4.5 Hz, 1H), 8.39 (d, J = 8.5 Hz, 1H), 8.10 (dd, J = 8.6, 1.3 Hz, 1H), 7.83–7.77 (m, 2H), 7.67 (ddd, J = 8.3, 6.8, 1.3 Hz, 1H), 7.30 (d, J = 5.6 Hz, 1H), 6.14 (p, J = 6.7 Hz, 1H)

^{13}C NMR (126 MHz, DMSO-d$_6$) δ 150.2, 147.9, 141.4, 129.7, 129.5, 127.0, 125.8, 125.0 (q, J = 283.9 Hz) 124.4, 120.7, 66.7 (q, J = 31.1 Hz)

^{19}F NMR (coupled, 471 MHz, DMSO-d$_6$) δ –75.5 (d, J = 7.0 Hz, 3F)

HRMS AMM (ESI–TOF) m/z calculated for $C_{11}H_9F_3NO^+$ $[M+H]^+$ 228.0631, found 228.0627

IR (ATR-FTIR) υ_{max} (cm^{-1}) 3020 br, 2844, 2708, 1124, 1043, 827, 768, 631

mp: 145–152 °C (lit.[14] mp 58–59 °C)

2,2,2-trifluoro-1-(quinolin-6-yl)ethan-1-ol (6-TFE)

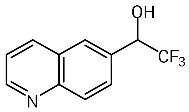

6-TFE

Prepared from **6** using general procedure B. The crude material was purified by flash column chromatography (5% MeOH in DCM) to afford **6-TFE** (107.9 mg, 95% yield) as an off-white solid.

^{1}H NMR (400 MHz, MeOD-d$_4$) δ 8.88 (dd, J = 4.3, 1.7 Hz, 1H), 8.40 (dd, J = 8.3, 1.7 Hz, 1H), 8.12 (d, J = 1.9 Hz, 1H), 8.08 (d, J = 8.8 Hz, 1H), 7.94 (d, J = 9.0 Hz, 1H), 7.57 (dd, J = 8.3, 4.3 Hz, 1H), 5.32 (q, J = 7.0 Hz, 1H)

^{13}C NMR (101 MHz, MeOD-d$_4$) δ 151.9, 148.8, 138.6, 135.9, 130.2, 129.4, 129.2, 128.7, 126.2 (q, J = 281.8 Hz), 123.0, 72.7 (q, J = 31.5 Hz)

^{19}F NMR (coupled, 376 MHz, MeOD-d$_4$) δ –79.4 (d, J = 7.0 Hz, 3F)

HRMS AMM (ESI–TOF) m/z calculated for $C_{11}H_9F_3NO^+$ $[M+H]^+$ 228.0631, found 228.0627

IR (ATR-FTIR) υ_{max} (cm^{-1}) 2847 br, 1256, 1149, 1091, 825, 762

mp: 186–188 °C

1-(4-(dimethylamino)quinazolin-6-yl)-2,2,2-trifluoroethan-1-ol (7-TFE)

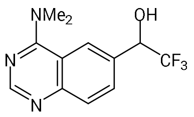

7-TFE

Prepared from **7** using general procedure B. The crude material was purified by flash column chromatography (gradient of 5 to 10% MeOH in DCM) to afford **7-TFE** (116.5 mg, 86% yield) as a colorless oil.

^{1}H NMR (500 MHz, CDCl$_3$) δ 8.46 (s, 1H), 8.16 (s, 1H), 7.77–7.62 (m, 2H), 5.16 (q, J = 6.7 Hz, 1H), 3.36 (s, 6H)

^{13}C NMR (126 MHz, CDCl$_3$) δ 163.4, 154.1, 151.2, 131.6, 127.4, 125.2, 124.7 (q, J = 297.7 Hz), 115.3, 72.0 (q, J =31.6 Hz), 42.0

^{19}F NMR (coupled, 376 MHz, CDCl$_3$) δ –78.0 (d, J = 6.5 Hz)

HRMS AMM (ESI–TOF) m/z calculated for $C_{12}H_{13}F_3N_3O^+$ $[M+H]^+$ 272.1005, found 272.1008

IR (ATR-FTIR) υ_{max} (cm^{-1}) 2922 br, 1576, 1540, 1360, 1262, 1121, 826, 688

2,2,2-trifluoro-1-(pyrimidin-5-yl)ethan-1-ol (8-TFE)

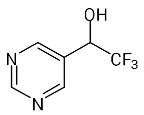

8-TFE

Prepared from **8** using general procedure B. The crude material was purified by flash column chromatography (gradient of 30 to 35% EtOAc in hexanes) to afford **8-TFE** (83.6 mg, 94% yield) as an orange oil. The NMR spectra of the purified product were consistent with literature spectra.[10]

^{1}H NMR (500 MHz, CDCl$_3$) δ 9.18 (s, 1H), 8.88 (s, 2H), 5.16 (q, J = 6.5 Hz, 1H)

^{19}F NMR (coupled, 471 MHz, CDCl$_3$) δ –78.5 (d, J = 6.7 Hz, 3F)

HRMS AMM (ESI–TOF) m/z calculated for $C_6H_4F_3N_2O^-$ [M–H]$^-$ 177.0281, found 177.0276

IR (ATR-FTIR) υ_{max} (cm^{-1}) 2959 br, 2872, 1170, 1132, 762, 668

2,2,2-trifluoro-1-(4-(trifluoromethyl)phenyl)ethan-1-ol (9-TFE)

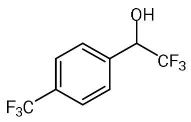

9-TFE

Prepared from **9** using general procedure B. The crude material was purified by flash column chromatography (5% EtOAc in hexanes) to afford **9-TFE** (102.5 mg, 84% yield) as a colorless oil. The NMR spectra of the purified product were consistent with literature spectra.[11]

^{1}H NMR (500 MHz, $CDCl_3$) δ 7.68 (d, J = 8.3 Hz, 2H), 7.62 (d, J = 8.1 Hz, 2H), 5.11 (q, J = 6.6 Hz, 1H), 2.85 (brs, 1H)

^{13}C NMR (126 MHz, $CDCl_3$) δ 137.7, 131.8 (q, J = 32.7 Hz), 125.7 (q, J = 3.8 Hz), 125.1 (d, J = 17.6 Hz), 122.9 (d, J = 7.4 Hz), 120.7 (d, J = 2.4 Hz), 72.3 (q, J = 32.3 Hz)

^{19}F NMR (coupled, 471 MHz, $CDCl_3$) δ –62.9 (s, 3F), –78.4 (d, J = 3.2 Hz, 3F)

1-(3-bromo-5-fluorophenyl)-2,2,2-trifluoroethan-1-ol (10-TFE)

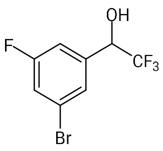

10-TFE

Prepared from **10** using general procedure B. The crude material was purified by flash column chromatography (gradient of 0 to 5% EtOAc in hexanes) to afford **10-TFE** (79.2 mg, 58% yield) as a colorless oil. The NMR spectra of the purified product were consistent with literature spectra.[12]

^{1}H NMR (400 MHz, $CDCl_3$) δ 7.44 (s, 1H), 7.30 (dt, J = 7.9, 2.1 Hz, 1H), 7.18 (dt, J = 8.9, 1.8 Hz, 1H), 5.01 (qd, J = 6.4, 4.1 Hz, 1H), 2.77 (d, J = 4.5 Hz, 1H)

^{13}C NMR (101 MHz, $CDCl_3$) δ 162.7 (d, J = 251.7 Hz), 137.6 (d, J = 8.1 Hz), 126.6 (d, J = 3.2 Hz), 123.9 (q, J = 282.1 Hz), 122.9 (d, J = 9.9 Hz), 120.4 (d, J = 24.2 Hz), 113.8 (d, J = 22.9 Hz), 71.7 (qd, J = 32.5, 2.1 Hz)

^{19}F NMR (coupled, 376 MHz, $CDCl_3$) δ –78.3 (d, J = 6.4 Hz, 3F), –109.7 (t, J = 8.5 Hz, 1F)

1-(benzofuran-2-yl)-2,2,2-trifluoroethan-1-ol (11-TFE)

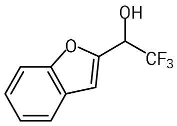

11-TFE

Prepared from **11** using general procedure B. The crude material was purified by flash column chromatography (15% EtOAc in hexanes) to afford **11-TFE** (98.4 mg, 91% yield) as a pale yellow oil. The NMR spectra of the purified product were consistent with literature spectra.[13]

^{1}H NMR (500 MHz, CDCl$_3$) δ 7.61 (dt, J = 7.7, 1.0 Hz, 1H), 7.52 (dq, J = 8.3, 0.9 Hz, 1H), 7.36 (ddd, J = 8.5, 7.3, 1.4 Hz, 1H), 7.28 (td, J = 7.5, 1.0 Hz, 1H), 6.91 (s, 1H), 5.20 (q, J = 6.4 Hz, 1H), 2.95 (brs, 1H)

^{13}C NMR (126 MHz, CDCl$_3$) δ 155.2, 149.4 (q, J = 1.6 Hz), 127.5, 125.6, 123.5, 123.5 (q, J = 282.3 Hz), 121.8, 111.7, 107.1, 67.9 (q, J = 34.3 Hz)

^{19}F NMR (coupled, 471 MHz, CDCl$_3$) δ –76.9 (d, J = 6.2 Hz, 3F)

2,2,2-trifluoro-1-(1-methyl-1*H*-benzo[*d*]imidazol-2-yl)ethan-1-ol (12-TFE)

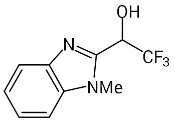

12-TFE

Prepared from **12** using a modified version of general procedure B. A total of 3.0 equiv each of TBS-Cl and imidazole were used instead of 2.0 equiv. The crude material was purified by flash column chromatography (10% EtOAc in DCM) to afford **12-TFE** (90.9 mg, 79% yield) as a white solid.

^{1}H NMR (400 MHz, MeOD-d$_4$) δ 7.70 (dt, J = 7.9, 1.1 Hz, 1H), 7.57 (dt, J = 8.2, 1.0 Hz, 1H), 7.39 (ddd, J = 8.3, 7.2, 1.2 Hz, 1H), 7.33 (ddd, J = 8.3, 7.1, 1.2 Hz, 1H), 5.57 (q, J = 7.3 Hz, 1H), 4.02 (s, 3H)

^{13}C NMR (101 MHz, MeOD-d$_4$) δ 148.8 (q, J = 1.7 Hz), 142.5, 137.6, 125.5 (q, J = 282.3 Hz), 124.9, 124.0, 120.1, 111.3, 69.1 (q, J = 33.4 Hz), 31.4 (q, J = 1.8 Hz)

^{19}F NMR (coupled, 376 MHz, MeOD-d$_4$) δ –77.7 (d, J = 7.0 Hz, 3F)

HRMS AMM (ESI–TOF) m/z calculated for $C_{10}H_{10}F_3N_2O^+$ 231.0740 $[M+H]^+$, found 231.0739

IR (ATR-FTIR) υ_{max} (cm^{-1}) 3115 br, 2922, 1181, 1133, 1122, 1106, 1005, 840, 745

mp: 119–120 °C (lit.[7] mp 144–146 °C)

1-(benzo[*d*]thiazol-2-yl)-2,2,2-trifluoroethan-1-ol (13-TFE)

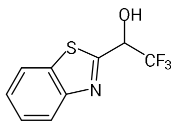

13-TFE

Prepared from **13** using general procedure B. The crude material was purified by flash column chromatography (20% EtOAc in hexanes) to afford **13-TFE** (90.9 mg, 86% yield) as an off-white solid. The NMR spectra of the purified product were consistent with literature spectra.[14]

^{1}H NMR (400 MHz, $CDCl_3$) δ 8.08 (dd, J = 8.2, 1.2 Hz, 1H), 7.93 (dd, J = 8.2, 1.3 Hz, 1H), 7.54 (ddd, J = 8.3, 7.2, 1.4 Hz, 1H), 7.47 (td, J = 7.7, 1.2 Hz, 1H), 5.44 (q, J = 6.3 Hz, 1H), 4.87 (s, 1H)

^{13}C NMR (101 MHz, $CDCl_3$) δ 163.7, 151.6, 135.5, 126.9, 126.4, 123.7, 123.2 (q, J = 283.2 Hz), 122.0, 71.0 (q, J = 33.5 Hz)

^{19}F NMR (coupled, 376 MHz, $CDCl_3$) δ –77.5 (d, J = 6.7 Hz, 3F)

IR (ATR-FTIR) υ_{max} (cm^{-1}) 3100 br, 2846, 1260, 1168, 1135, 1017, 842, 762, 753, 729

1-(benzo[*d*]oxazol-2-yl)-2,2,2-trifluoroethan-1-ol (14-TFE)

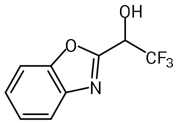

14-TFE

Prepared from **14** using general procedure B. The crude material was purified by flash column chromatography (10% EtOAc in hexanes) to afford **14-TFE** (101.0 mg, 93% yield) as a colorless oil.

^{1}H NMR (400 MHz, $CDCl_3$) δ 7.84–7.75 (m, 1H), 7.65–7.54 (m, 1H), 7.50–7.37 (m, 2H), 5.48 (brs, 1H), 5.38 (q, J = 6.3 Hz, 1H)

^{13}C NMR (101 MHz, $CDCl_3$) δ 159.7 (q, J = 1.9 Hz), 151.3, 139.7, 126.6, 125.5, 122.9 (q, J = 283.6 Hz), 120.6, 111.4, 67.9 (q, J = 34.7 Hz)

^{19}F NMR (coupled, 376 MHz, $CDCl_3$) δ –77.0 (d, J = 6.1 Hz, 3F)

HRMS AMM (ESI–TOF) m/z calculated for $C_9H_7F_3NO_2^+$ $[M+H]^+$ 218.0423, found 218.0419

IR (ATR-FTIR) υ_{max} (cm^{-1}) 3115 br, 2884, 1184, 1122, 1004, 840, 760

1-(1-benzyl-1*H*-imidazol-2-yl)-2,2,2-trifluoroethan-1-ol (15-TFE)

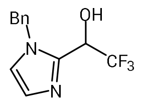

15-TFE

Prepared from **15** using general procedure B. The crude material was purified by flash column chromatography (4% MeOH in DCM) to afford **15-TFE** (71.7 mg, 56% yield) as a white solid.

^{1}H NMR (500 MHz, CDCl$_3$) δ 7.37–7.30 (m, 3H), 7.21–7.16 (m, 2H), 6.84 (d, J = 1.4 Hz, 1H), 6.79 (d, J = 1.3 Hz, 1H), 5.48 (d, J = 15.3 Hz, 1H), 5.23 (d, J = 15.2 Hz, 1H), 5.21 (q, J = 7.23 Hz, 1H)

^{13}C NMR (126 MHz, CDCl$_3$) δ 142.0, 135.9, 129.0, 128.3, 127.7, 127.4, 124.1 (q, J = 283.5 Hz), 122.0, 67.2 (q, J = 33.5 Hz), 50.5

^{19}F NMR (coupled, 471 MHz, CDCl$_3$) δ –76.6 (d, J = 7.4 Hz, 3F)

HRMS AMM (ESI–TOF) m/z calculated for $C_{12}H_{12}F_3N_2O^+$ $[M+H]^+$ 257.0896, found 257.0892

IR (ATR-FTIR) υ_{max} (cm^{-1}) 2718 br, 1267, 1165, 1145, 1116, 1091, 760, 695, 685

mp: 117–121 °C (lit.[15] mp 114–115 °C)

2,2,2-trifluoro-1-(4-(4-(trifluoromethyl)phenyl)-4*H*-1,2,4-triazol-3-yl)ethan-1-ol (16-TFE)

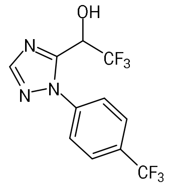

16-TFE

Prepared from **16** using general procedure B. The crude material was purified by flash column chromatography (25% [3:1 EtOAc:EtOH mixture] in hexanes) to afford of **16-TFE** (143.1 mg, 92% yield) as a white solid.

^{1}H NMR (400 MHz, MeOD-d4) δ 8.23 (s, 1H), 7.96 (d, J = 8.6 Hz, 2H), 7.84 (d, J = 8.2 Hz, 2H), 5.40 (q, J = 6.5 Hz, 1H)

^{13}C NMR (101 MHz, MeOD-d4) δ 152.6, 151.6, 141.4, 132.7 (q, J = 33.1 Hz), 127.9 (q, J = 3.7 Hz), 127.4, 125.1 (q, J = 271.5 Hz), 125.0 (q, J = 281.9 Hz), 66.0 (q, J = 33.9 Hz)

^{19}F NMR (coupled, 376 MHz, MeOD-d4) δ –64.2 (s, 3F), –77.6 (d, J = 6.7 Hz, 3F)

HRMS AMM (ESI–TOF) m/z calculated for $C_{11}H_8F_6N_3O^+$ $[M+H]^+$ 312.0566, found 312.0566

IR (ATR-FTIR) υ_{max} (cm^{-1}) 3091 br, 2921, 1319, 1270, 1124, 1063, 1035, 845, 780

mp: 131–134 °C

2,2,2-trifluoro-1-(5-(pyridin-2-yl)thiophen-2-yl)ethan-1-ol (17-TFE)

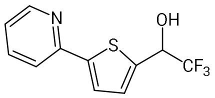

17-TFE

Prepared from **17** using general procedure B. The crude material was purified by flash column chromatography (30% EtOAc in hexanes) to afford **17-TFE** (119.1 mg, 92% yield) as a white solid.

^{1}H NMR (500 MHz, CDCl$_3$) δ 8.51 (d, J = 5.0 Hz, 1H), 7.71 (td, J = 7.8, 1.8 Hz, 1H), 7.64–7.59 (m, 1H), 7.41 (d, J = 3.8 Hz, 1H), 7.23–7.17 (m, 1H), 7.12 (d, J = 3.8 Hz, 1H), 5.22 (q, J = 6.5 Hz, 1H)

^{13}C NMR (126 MHz, CDCl$_3$) δ 152.0, 149.4, 145.1, 139.4, 137.4, 127.9, 124.9, 123.9 (q, J = 282.2 Hz), 122.7, 119.6, 69.1 (q, J = 33.6 Hz)

^{19}F NMR (coupled, 471 MHz, CDCl$_3$) δ –78.2 (d, J = 6.4 Hz, 3F)

HRMS AMM (ESI–TOF) m/z calculated for $C_{11}H_9F_3NOS^+$ $[M+H]^+$ 260.0351, found 260.0348

IR (ATR-FTIR) υ_{max} (cm^{-1}) 3102 br, 1262, 1159, 1114, 1001, 772

mp: 110–115 °C

2,2,2-trifluoro-1-(5-(4-(trifluoromethyl)phenyl)thiophen-2-yl)ethan-1-ol (18-TFE)

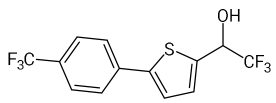

18-TFE

Prepared from **18** using general procedure B. The crude material was purified by flash column chromatography (20% EtOAc in hexanes) to afford **18-TFE** (138.7 mg, 85% yield) as a light yellow solid.

^{1}H NMR (400 MHz, $CDCl_3$) δ 7.68 (d, J = 8.3 Hz, 2H), 7.63 (d, J = 8.4 Hz, 2H), 7.30 (d, J = 3.8 Hz, 1H), 7.20 (d, J = 3.8 Hz, 1H), 5.30 (dq, J = 9.8, 5.8 Hz, 1H), 2.90 (d, J = 4.3 Hz, 1H)

^{13}C NMR (101 MHz, $CDCl_3$) δ 144.4, 137.2, 136.8, 130.0 (q, J = 32.6 Hz), 128.7, 128.4, 128.0, 126.1 (q, J = 3.6 Hz), 124.2 (q, J = 272.2 Hz), 123.8 (q, J = 282.1 Hz), 69.6 (q, J = 33.8 Hz)

^{19}F NMR (coupled, 376 MHz, $CDCl_3$) δ –62.6 (s, 3F), –78.6 (d, J = 6.3 Hz, 3F)

HRMS AMM (ESI–TOF) m/z calculated for $C_{13}H_7F_6S$ $[M–OH]^+$ 309.0173, found 309.0165

IR (ATR-FTIR) υ_{max} (cm^{-1}) 2921 br, 1327, 1165, 1108, 1072, 1053, 841, 801, 695

mp: 88–89 °C

2,2,2-trifluoro-1-(1-methyl-1*H*-indol-2-yl)ethan-1-ol (19-TFE)

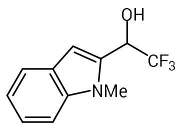

19-TFE

Prepared from **19** using a modified version of general procedure B. A total of 3.0 equiv each of TBS-Cl and imidazole were used instead of 2.0 equiv. The crude material was purified by flash column chromatography (10% EtOAc in hexanes) to afford **19-TFE** (83.7 mg, 73% yield) as a white solid. The NMR spectra of the purified product were consistent with literature spectra.[13]

^{1}H NMR (500 MHz, $CDCl_3$) δ 7.64 (dt, J = 7.9, 1.0 Hz, 1H), 7.40–7.34 (m, 1H), 7.30 (ddd, J = 8.3, 6.9, 1.2 Hz, 1H), 7.15 (ddd, J = 7.9, 6.9, 1.1 Hz, 1H), 5.26 (p, J = 6.6 Hz, 1H), 3.81 (s, 3H), 2.49 (d, J = 7.0 Hz, 1H)

^{13}C NMR (126 MHz, $CDCl_3$) δ 138.1, 131.9, 126.9, 124.2 (q, J = 281.9 Hz), 123.1, 121.5, 120.3, 109.6, 102.4 (q, J = 2.2 Hz), 67.1 (q, J = 33.7 Hz), 30.4

^{19}F NMR (coupled, 471 MHz, $CDCl_3$) δ –76.5 (d, J = 6.7 Hz, 3F)

General Procedure C: Silylation-Decarboxylation of α,α-trifluoromethyl(hydroxy) Esters

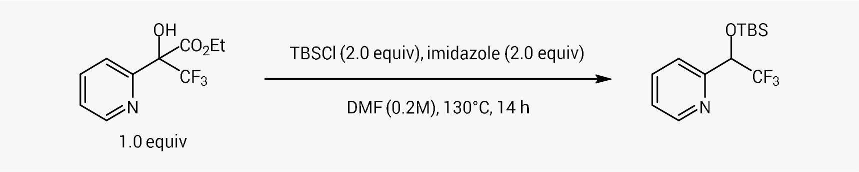

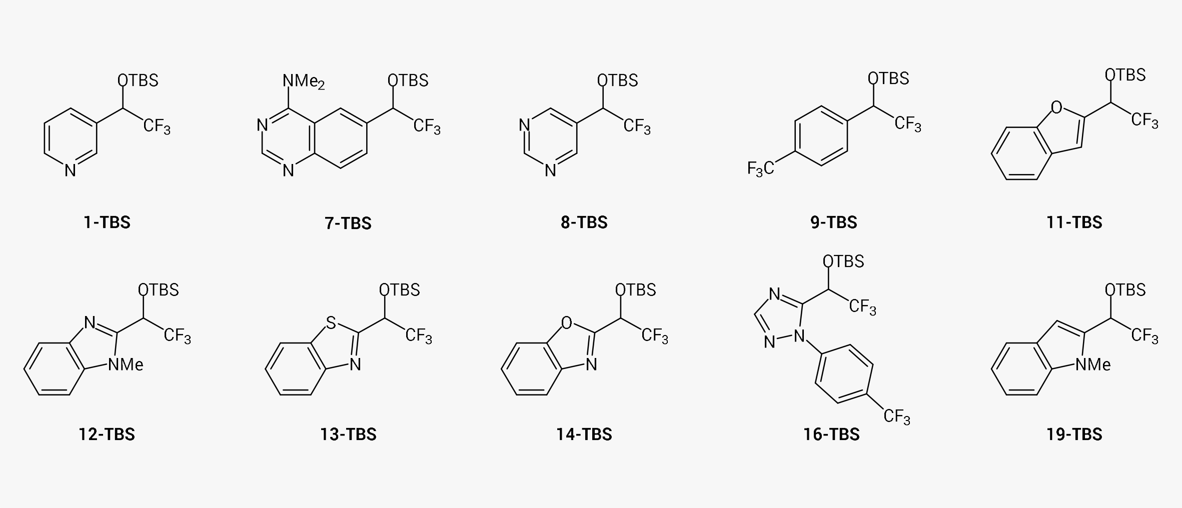

To an oven-dried 8 mL vial and stir bar was added α,α-trifluoromethyl(hydroxy) ester (0.5 mmol, 1.0 equiv), imidazole (68.1 mg, 1.0 mmol, 2.0 equiv), *tert*-butyldimethylsilyl chloride (150.7 mg, 1.0 mmol, 2.0 equiv), and DMF (2.5 mL, 0.2 M). All reagents were added open to air and no further considerations were made to purge the solution. The vial was tightly sealed with a screw-on PTFE septum cap. The vial was then heated to 130 °C while stirring at 1000 RPM. After 14 hours, the reaction mixture was allowed too cool to room temperature, before diluting with H_2O (2 mL) and Et_2O (2 mL), then transferred to a separatory funnel. The aqueous layer was extracted with Et_2O (5 × 10mL) and the organic extractions were collected and washed with brine. The organics were dried over Na_2SO_4, filtered, and concentrated under reduced pressure to afford the crude reaction mixture. The crude material was then dry loaded onto silica gel for purification by flash column chromatography.

3-(1-((*tert*-butyldimethylsilyl)oxy)-2,2,2-trifluoroethyl)pyridine (1-TBS)

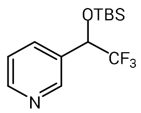

1-TBS

Prepared from **1** using general procedure C. The crude material was purified by flash column chromatography (gradient of 20 to 25% EtOAc in hexanes) to afford **1-TBS** (138.4 mg, 95% yield) as a colorless oil.

^{1}H NMR (500 MHz, CDCl$_3$) δ 8.65 (d, J = 2.2 Hz, 1H), 8.62 (dd, J = 4.8, 1.7 Hz, 1H), 7.80 (dt, J = 7.8, 2.0 Hz, 1H), 7.34–7.29 (m, 1H), 4.95 (q, J = 6.3 Hz, 1H), 0.88 (s, 9H), 0.12 (s, 3H), –0.02 (s, 3H)

^{13}C NMR (126 MHz, CDCl$_3$) δ 150.7, 149.2, 135.3, 131.4, 124.0 (q, J = 282.5 Hz), 123.5, 71.9 (q, J = 32.8 Hz), 25.6, 18.2, –5.0, –5.2

^{19}F NMR (coupled, 471 MHz, CDCl$_3$) δ –78.6 (d, J = 6.2 Hz, 3F)

HRMS AMM (ESI–TOF) m/z calculated for $C_{13}H_{21}F_3NOSi^+$ $[M+H]^+$ 292.1339, found 292.1337

IR (ATR-FTIR) υ_{max} (cm^{-1}) 1271, 1169, 1130, 1025, 853, 836, 779

6-(1-((*tert*-butyldimethylsilyl)oxy)-2,2,2-trifluoroethyl)-*N*,*N*-dimethylquinazolin-4-amine (7-TBS)

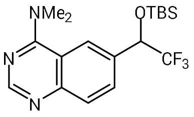

7-TBS

Prepared from **7** using general procedure C. The crude material was purified by flash column chromatography (35% EtOAc in hexanes) to afford **7-TBS** (175.3 mg, 89% yield) as a colorless oil.

^{1}H NMR (500 MHz, $CDCl_3$) δ 8.63 (s, 1H), 8.09 (d, J = 2.1 Hz, 1H), 7.84 (d, J = 8.6 Hz, 1H), 7.74 (dd, J = 8.6, 1.8 Hz, 1H), 5.02 (q, J = 6.3 Hz, 1H), 3.35 (s, 6H), 0.88 (s, 9H), 0.12 (s, 3H), –0.04 (s, 3H)

^{13}C NMR (126 MHz, $CDCl_3$) δ 163.6, 154.6, 152.3, 131.6, 131.2, 128.5, 125.3 (q, J = 283.3 Hz), 124.9, 115.7, 73.5 (q, J = 32.2 Hz), 41.9, 25.5, –4.9, –5.1

^{19}F NMR (coupled, 471 MHz, $CDCl_3$) δ –78.4 (d, J = 6.1 Hz, 3F)

HRMS AMM (ESI–TOF) m/z calculated for $C_{18}H_{27}F_3N_3OSi^+$ $[M+H]^+$ 386.1870, found 386.1877

IR (ATR-FTIR) υ_{max} (cm^{-1}) 1567, 1534, 1352, 1166, 1127, 1070, 833, 779

5-(1-((*tert*-butyldimethylsilyl)oxy)-2,2,2-trifluoroethyl)pyrimidine (8-TBS)

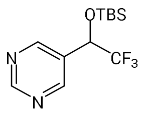

8-TBS

Prepared from **8** using general procedure C. The crude material was purified by flash column chromatography (15% EtOAc in hexanes) to afford **8-TBS** (138.8 mg, 95% yield) as a colorless oil.

^{1}H NMR (500 MHz, CDCl$_3$) δ 9.25 (s, 1H), 8.83 (s, 2H), 4.98 (q, J = 6.2 Hz, 1H), 0.90 (s, 9H), 0.17 (s, 3H), 0.03 (s, 3H)

^{13}C NMR (126 MHz, CDCl$_3$) δ 159.5, 156.3, 129.5, 123.7 (q, J =282.9 Hz), 70.5 (q, J = 33.6 Hz), 25.6, –5.0, –5.2

^{19}F NMR (coupled, 471 MHz, CDCl$_3$) δ –78.6 (d, J = 6.1 Hz, 3F)

HRMS AMM (ESI–TOF) m/z calculated for $C_{12}H_{20}F_3N_2OSi^+$ [M+H]$^+$ 293.1292, found 293.1295

IR (ATR-FTIR) υ_{max} (cm^{-1}) 2962, 1667, 1256, 1165, 1122, 870, 749

2,2,2-trifluoro-1-(4-(trifluoromethyl)phenyl)ethan-1-ol (9-TBS)

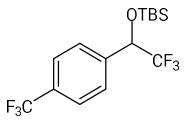

9-TBS

Prepared from **9** using general procedure C. The crude material was purified by flash column chromatography (100% hexanes) to afford **9-TBS** (150.4 mg, 84% yield) as a colorless oil.

^{1}H NMR (500 MHz, $CDCl_3$) δ 7.65 (d, J = 8.7 Hz, 2H), 7.59 (d, J = 1.4 Hz, 2H), 4.98 (q, J = 6.4 Hz, 1H), 0.90 (s, 9H), 0.13 (s, 1H), –0.01 (s, 1H)

^{13}C NMR (126 MHz, $CDCl_3$) δ 139.5, 131.5 (q, J = 32.4 Hz), 128.1, 125.5 (q, J = 3.7 Hz), 124.1 (q, J = 272.3 Hz), 124.0 (q, J = 282.9 Hz), 73.2 (q, J = 32.4 Hz), 25.6, 18.3, –5.0, –5.2

^{19}F NMR (coupled, 471 MHz, $CDCl_3$) δ –62.8 (s, 3F), –78.4 (d, J = 6.1 Hz, 3F)

LR GCMS (EI–TQD) m/z calculated $C_{11}H_{11}F_6OSi^{\bullet+}$ $[M–C_4H_9^{\bullet}]^+$ 301.0483, found 301

IR (ATR-FTIR) υ_{max} (cm^{-1}) 2928, 1663, 1385, 1089, 658

(1-(benzofuran-2-yl)-2,2,2-trifluoroethoxy)(*tert*-butyl)dimethylsilane (11-TBS)

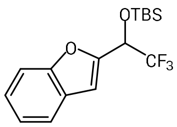

11-TBS

Prepared from **11** using general procedure C. The crude material was purified by flash column chromatography (gradient of 0 to 10% DCM in hexanes) to afford **11-TBS** (152.0 mg, 92% yield) as a colorless oil.

^{1}H NMR (400 MHz, $CDCl_3$) δ 7.61 (ddd, J = 7.7, 1.4, 0.7 Hz, 1H), 7.53 (dq, J = 8.3, 1.0 Hz, 1H), 7.34 (ddd, J = 8.3, 7.3, 1.4 Hz, 1H), 7.27 (td, J = 7.5, 1.1 Hz, 1H), 6.89 (s, 1H), 5.16 (q, J = 6.1 Hz, 1H), 0.94 (s, 9H), 0.18 (s, 3H), 0.08 (s, 3H)

^{13}C NMR (101 MHz, $CDCl_3$) δ 155.2, 151.6, 127.8, 125.1, 123.6 (q, J = 282.7 Hz), 123.3, 121.6, 111.7, 106.5, 68.8 (q, J = 34.2 Hz), 25.6, 18.3, –5.0, –5.3

^{19}F NMR (coupled, 376 MHz, $CDCl_3$) δ –77.7 (d, J = 6.0 Hz, 3F)

HRMS AMM (APPI–TOF) m/z calculated for $C_{10}H_6F_3O^+$ $[M–C_6H_{15}OSi]^+$ 199.0365, found 199.0366

IR (ATR-FTIR) υ_{max} (cm^{-1}) 1253, 1175, 1155, 1049, 833, 773

2-(1-((*tert*-butyldimethylsilyl)oxy)-2,2,2-trifluoroethyl)-1-methyl-1*H*-benzo[*d*]imidazole (12-TBS)

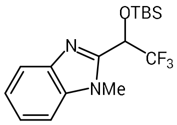

12-TBS

Prepared from **12** using a modified version of general procedure C. A total of 3.0 equiv each of TBS-Cl and imidazole were used instead of 2.0 equiv. The crude material was purified by flash column chromatography (15% EtOAc in hexanes) to afford **12-TBS** (141.20 mg, 82% yield) as a colorless oil.

^{1}H NMR (400 MHz, $CDCl_3$) δ 7.81–7.74 (m, 1H), 7.45–7.39 (m, 1H), 7.36 (td, J = 7.5, 1.3 Hz, 1H), 7.31 (ddd, J = 8.4, 7.1, 1.5 Hz, 1H), 5.54 (q, J = 7.3 Hz, 1H), 3.99 (s, 3H), 0.90 (s, 9H), 0.19 (s, 3H), 0.01 (s, 3H)

^{13}C NMR (101 MHz, $CDCl_3$) δ 146.7 (q, J = 1.8 Hz), 142.2, 137.0, 123.8 (q, J = 282.6 Hz), 123.8, 122.7, 120.5, 109.8, 71.8 (q, J = 34.0 Hz), 31.3 (q, J = 3.1 Hz), 25.6, 18.3, –5.3, –5.4

^{19}F NMR (coupled, 376 MHz, $CDCl_3$) δ –75.9 (d, J = 7.0 Hz, 3F)

HRMS AMM (ESI–TOF) m/z calculated for $C_{16}H_{24}F_3N_2OSi^+$ $[M+H]^+$ 345.1605, found 345.1608

IR (ATR-FTIR) υ_{max} (cm^{-1}) 1263, 1177, 1135, 850, 836, 742

2-(1-((*tert*-butyldimethylsilyl)oxy)-2,2,2-trifluoroethyl)benzo[*d*]thiazole (13-TBS)

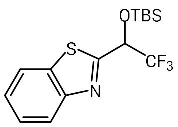

13-TBS

Prepared from **13** using general procedure C. The crude material was purified by flash column chromatography (gradient of 20 to 25% EtOAc in hexanes) to afford **13-TBS** (152.9 mg, 88% yield) as a pale yellow oil.

^{1}H NMR (500 MHz, $CDCl_3$) δ 8.08 (dt, J = 8.2, 0.9 Hz, 1H), 7.94 (dt, J = 8.0, 0.9 Hz, 1H), 7.52 (ddd, J = 8.2, 7.2, 1.2 Hz, 1H), 7.45 (ddd, J = 8.3, 7.3, 1.2 Hz, 1H), 5.44 (q, J = 6.0 Hz, 1H), 0.96 (s, 9H), 0.20 (s, 3H), 0.10 (s, 3H)

^{13}C NMR (126 MHz, $CDCl_3$) δ 167.1, 153.0, 135.3, 126.5, 125.9, 123.8, 123.1 (q, J = 283.7 Hz), 122.0, 72.6 (q, J = 33.3 Hz), 25.6, 18.3, -5.1, -5.2

^{19}F NMR (coupled, 471 MHz, $CDCl_3$) δ –77.3 (d, J = 6.1 Hz, 3F)

HRMS AMM (ESI–TOF) m/z calculated for $C_{15}H_{21}F_3NOSSi^+$ $[M+H]^+$ 348.1060, found 348.1055

IR (ATR-FTIR) υ_{max} (cm^{-1}) 1517, 1471, 1179, 1134, 836, 781, 759

2-(1-((*tert*-butyldimethylsilyl)oxy)-2,2,2-trifluoroethyl)benzo[*d*]oxazole (14-TBS)

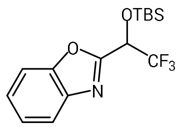

14-TBS

Prepared from **14** using general procedure C. The crude material was purified by flash column chromatography (gradient of 15 to 30% DCM in hexanes) to afford **14-TBS** (157.4 mg, 95% yield) as a colorless oil.

^{1}H NMR (500 MHz, $CDCl_3$) δ 7.82–7.77 (m, 1H), 7.69–7.58 (m, 1H), 7.47–7.31 (m, 2H), 5.33 (q, J = 6.1 Hz, 1H), 0.90 (s, 9H), 0.18 (s, 3H), 0.07 (s, 3H)

^{13}C NMR (101 MHz, $CDCl_3$) δ 159.2 (q, J = 1.9 Hz), 151.1, 140.6, 126.3, 125.1, 122.9 (q, J = 283.2 Hz), 69.1 (q, J = 34.9 Hz), 121.0, 111.3, 25.5, 18.3, –5.2, –5.3

^{19}F NMR (coupled, 471 MHz, $CDCl_3$) δ –76.9 (d, J = 6.4 Hz, 3F)

HRMS AMM (ESI–TOF) m/z calculated for $C_{15}H_{21}F_3NO_2Si^+$ $[M+H]^+$ 332.1288, found 332.1286

IR (ATR-FTIR) υ_{max} (cm^{-1}) 1180, 1143, 1027, 745

5-(1-((*tert*-butyldimethylsilyl)oxy)-2,2,2-trifluoroethyl)-1-(4-(trifluoromethyl)phenyl)-1*H*-1,2,4-triazole (16-TBS)

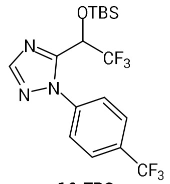

16-TBS

Prepared from **16** using general procedure C. The crude material was purified by flash column chromatography (15% EtOAc in hexanes) to afford **16-TBS** (197.8 mg, 93% yield) as a white solid.

^{1}H NMR (400 MHz, $CDCl_3$) δ 8.11 (s, 1H), 7.79 (d, J = 8.6 Hz, 2H), 7.64 (d, J = 8.3 Hz, 2H), 5.38 (q, J = 6.3 Hz, 1H), 0.76 (s, 9H), 0.01 (s, 3H), –0.11 (s, 3H)

^{13}C NMR (101 MHz, $CDCl_3$) δ 152.2, 149.8, 140.4, 132.0 (q, J = 33.1 Hz), 126.6 (q, J = 3.6 Hz), 126.4, 123.6 (q, J = 273.0 Hz), 123.1 (q, J = 283.3 Hz), 68.1 (q, J = 35.0 Hz), 25.4, 18.1, –5.1, –5.5

^{19}F NMR (coupled, 376 MHz, $CDCl_3$) δ –62.8 (s, 3F), –75.5 (d, J = 6.3 Hz, 3F)

HRMS AMM (ESI–TOF) m/z calculated for $C_{17}H_{22}F_6N_3OSi^+$ $[M+H]^+$ 426.1431, found 426.1435

IR (ATR-FTIR) υ_{max} (cm^{-1}) 1667, 1257, 1165, 1116, 772, 652

mp: 65–72 °C

2-(1-((*tert*-butyldimethylsilyl)oxy)-2,2,2-trifluoroethyl)-1-methyl-1*H*-indole (19-TBS)

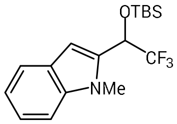

19-TBS

Prepared from **19** using a modified version of general procedure C. A total of 3.0 equiv each of TBS-Cl and imidazole were used instead of 2.0 equiv. The crude material was purified by flash column chromatography (gradient of 0 to 10% DCM in hexanes) to afford **19-TBS** (127.1 mg, 74% yield) as a colorless oil.

^{1}H NMR (500 MHz, $CDCl_3$) δ 7.65 (ddq, J = 7.9, 3.3, 1.0 Hz, 1H), 7.39 (dt, J = 8.4, 1.0 Hz, 1H), 7.31 (ddd, J = 8.3, 7.0, 1.2 Hz, 1H), 7.17 (ddt, J = 8.1, 7.0, 1.1 Hz, 1H), 5.26 (q, J = 7.0 Hz, 1H), 3.90 (s, 3H), 0.94 (s, 9H), 0.18 (s, 3H), 0.02 (s, 3H)

^{13}C NMR (126 MHz, $CDCl_3$) δ 138.8, 132.7, 127.1, 124.1 (q, J = 282.6 Hz), 122.6, 121.2, 120.0, 109.6, 104.4, 69.7 (q, J = 34.1 Hz), 31.3 (q, J = 2.4 Hz), 25.6, 18.3, –5.1, –5.4

^{19}F NMR (coupled, 471 MHz, $CDCl_3$) δ –76.7 (d, J = 6.8 Hz, 3F)

HRMS AMM (ESI–TOF) m/z calculated for $C_{17}H_{25}F_3NOSi^+$ $[M+H]^+$ 344.1652, found 344.1649

IR (ATR-FTIR) υ_{max} (cm^{-1}) 1124, 1101, 1006, 779

General Procedure D: Preparation of Difluoromethyl Ketones

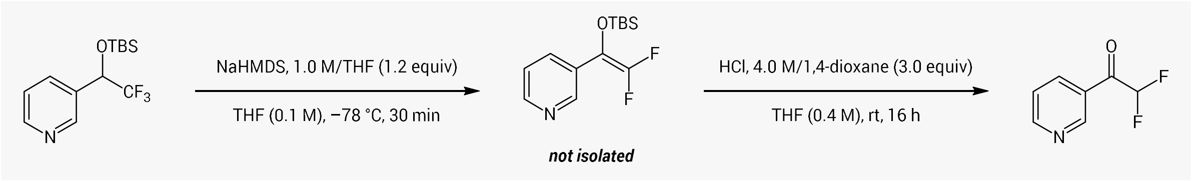

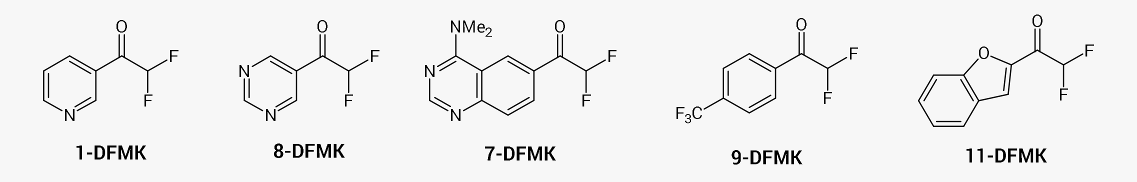

To a flame-dried 20 mL vial and a stir bar was added silyl ether (0.5 mmol, 1.0 equiv) and THF (5.0 mL, 0.1 M). The vial was tightly sealed with a screw-on PTFE septum cap, cooled to –78 °C, and purged with a constant flow of nitrogen for 15 minutes. To the vial was added a solution of sodium bis(trimethylsilyl)amide (0.6 mL, 0.6 mmol, 1.2 equiv, 1.0 M/THF) dropwise. The reaction was then stirred for 30 minutes under nitrogen atmosphere, maintaining a temperature of –78 °C. The reaction mixture was diluted with H_2O (5 mL) and Et_2O (5 mL), then transferred to a separatory funnel. The aqueous layer was extracted with Et_2O (4 × 5 mL) and the organic extractions were collected and washed with brine. The organics were dried over Na_2SO_4, filtered, and evaporated to afford the crude reaction mixture. The crude material was then dry loaded onto silica gel and eluted through a short plug of silica gel with a mixture of 1:1 hexanes:DCM. After concentrating under reduced pressure, the material was used without any further purification and subjected to the next step.

Note: The tert-butyldimethylsilyl difluoro enol ether products were observed to decompose, primarily to the self-aldol product, at room temperature but could be stored neat at –4 °C for at least one month without decomposition.

To an oven-dried 8 mL vial and a stir bar was added the silyl difluoro enol ether product and THF (1.25 mL, 0.4 M). To the reaction vial was added HCl in 1,4-dioxane (375 µL, 1.5 mmol, 3.0 equiv, 4.0 M/dioxane). The vial was tightly sealed with a screw-on PTFE septum cap. All reagents were added open to air and no further considerations were made to purge the solution. The reaction was stirred at room temperature and monitored for starting material consumption by TLC. Upon completion, the reaction was neutralized with saturated $NaHCO_3$ (2 mL), diluted with H_2O (2 mL) and EtOAc (2 mL), then transferred to a separatory funnel. The aqueous layer was extracted with EtOAc (5 × 5 mL) and the organic extractions were collected and washed with brine. The organics were dried over Na_2SO_4, filtered, and evaporated to afford the crude reaction mixture. The crude material was then dry loaded onto silica gel for purification by flash column chromatography.

If the reaction conditions led to the formation of a precipitate, the mixture could be triturated with Et_2O without neutralization, filtered, and washed with cold Et_2O (3 × 3 mL) to obtain the pure product as its hydrochloride salt.

2,2-difluoro-1-(pyridin-3-yl)ethan-1-one (1-DFMK)

Note: isolated as HCl salt in hydrate form (>20:1 hydrate:ketone)

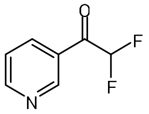

1-DFMK

Prepared from **1-TBS** using general procedure D. The reaction mixture was triturated with cold Et_2O and filtered to afford **1-DFMK** (100.2 mg, 95% yield) as a white solid. The NMR spectra of the purified product were consistent with literature spectra.[16]

Hydrate:

^{1}H NMR (400 MHz, DMSO-d$_6$) δ 8.97 (d, J = 5.5 Hz, 1H), 8.86–8.81 (m, 1H), 8.62 (d, J = 8.1 Hz, 1H), 8.11 (dd, J = 8.1, 5.5 Hz, 1H), 6.03 (t, J = 55.5 Hz, 1H)

^{13}C NMR (126 MHz, DMSO-d$_6$) δ 144.6, 142.3, 140.7, 139.2, 126.7, 113.9 (t, J = 249.2 Hz), 91.8 (t, J = 24.1 Hz)

^{19}F NMR (coupled, 376 MHz, DMSO-d$_6$) δ –125.7 (d, J = 55.7 Hz, 2F)

2,2-difluoro-1-(pyrimidin-5-yl)ethan-1-one (8-DFMK)

Note: isolated as a mixture of ketone:hydrate (1.2:1)

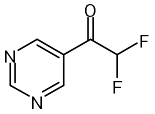

8-DFMK

Prepared from **8-TBS** using general procedure D. The crude material was purified by flash column chromatography (gradient of 20 to 25% EtOAc in hexanes) to afford **8-DFMK** (76.9 mg, 91% yield) as a white solid.

Ketone:

^{1}H NMR (500 MHz, DMSO-d$_6$) δ 8.61 (s, 1H), 8.48 (s, 1H), 7.98 (s, 1H), 6.31 (t, J = 52.3 Hz)

^{13}C NMR (126 MHz, DMSO-d$_6$) δ 185.9 (t, J = 25.0 Hz), 161.9, 157.5, 126.4, 108.9 (t, J = 245.9 Hz)

^{19}F NMR (coupled, 471 MHz, DMSO-d$_6$) δ –130.5 (d, J = 55.7 Hz, 2F)

HRMS AMM (ESI–TOF) m/z calculated for $C_6H_5F_2N_2O^+$ [M+H]$^+$ 159.0364, found 159.0367

IR (ATR-FTIR) υ_{max} (cm^{-1}) 1701, 1613, 1568, 1538, 1516, 1352, 1069, 975, 848

Hydrate:

^{1}H NMR (500 MHz, DMSO-d$_6$) δ 8.48 (s, 1H), 8.33 (s, 1H), 7.98 (s, 1H), 6.62 (s, 2H), 5.06 (t, J = 55.65 Hz)

^{13}C NMR (126 MHz, DMSO-d$_6$) δ 158.5, 156.0, 132.0, 114.2 (t, J = 248.6 Hz), 91.5 (t, J = 24.3 Hz)

^{19}F NMR (coupled, 471 MHz, DMSO-d$_6$) δ –128.5 (d, J = 52.8 Hz, 2F)

HRMS AMM (ESI–TOF) m/z calculated for $C_6H_7F_2N_2O_2^+$ [M+H]$^+$ 177.0470, found 177.0474

IR (ATR-FTIR) υ_{max} (cm^{-1}) 2922 br

mp: 146–152 °C

1-(4-(dimethylamino)quinazolin-6-yl)-2,2-difluoroethan-1-one (7-DFMK)

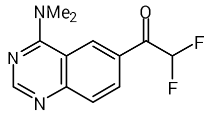

7-DFMK

Prepared from **7-TBS** using general procedure D. The crude material was purified by flash column chromatography (gradient of 20 to 30% EtOAc in hexanes) to afford **7-DFMK** (106.7 mg, 85% yield) as a white solid.

^{1}H NMR (500 MHz, CDCl$_3$) δ 8.89 (s, 1H), 8.68 (s, 1H), 8.29 (dd, J = 8.8, 2.0 Hz, 1H), 7.89 (d, J = 8.9 Hz, 1H), 6.29 (t, J = 53.5 Hz, 1H), 3.47 (s, 6H)

^{13}C NMR (126 MHz, CDCl$_3$) δ 186.4 (t, J = 25.9 Hz), 163.6, 156.8, 155.8, 131.2, 130.5 (t, J = 3.5 Hz), 129.2, 126.6 (t, J = 2.5 Hz), 115.6, 114.3, 112.3 (t, J = 255.0 Hz), 42.2

^{19}F NMR (coupled, 471 MHz, CDCl$_3$) δ –120.0 (d, J = 53.4 Hz, 2F)

HRMS AMM (ESI–TOF) m/z calculated for $C_{12}H_{12}F_2N_3O^+$ [M+H]$^+$ 252.0943, found 252.0943

IR (ATR-FTIR) υ_{max} (cm^{-1}) 1695, 1558, 1539, 1293, 1123, 1033, 728

mp: 119–120 °C

2,2-difluoro-1-(4-(trifluoromethyl)phenyl)ethan-1-one (9-DFMK)

Note: isolated as a mixture of ketone:hydrate (4:1)

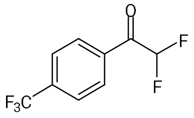

9-DFMK

Prepared from **9-TBS** using general procedure D. The crude material was purified by flash column chromatography (gradient of 0 to 5% EtOAc in hexanes) to afford **9-DFMK** (99.7 mg, 89% yield) as a white solid. The NMR spectra of the purified product were consistent with literature spectra.[16]

Ketone:

^{1}H NMR (500 MHz, $CDCl_3$) δ 8.23–8.15 (m, 2H), 7.80 (d, J = 8.3 Hz, 2H), 6.27 (t, J = 53.3 Hz, 1H)

^{13}C NMR (126 MHz, $CDCl_3$) δ 187.0 (t, J = 26.4 Hz), 136.1 (q, J = 33.0 Hz), 134.1, 130.2 (t, J = 2.5 Hz), 126.1 (q, J = 3.7 Hz), 122.3, 111.4 (t, J = 254.2 Hz)

^{19}F NMR (coupled, 471 MHz, $CDCl_3$) δ –121.7 (d, J = 53.0 Hz, 2F)

Hydrate:

^{1}H NMR (500 MHz, $CDCl_3$) δ 7.79 (d, J = 9.1 Hz, 2H), 7.69 (d, J = 8.3 Hz, 2H), 5.72 (t, J = 55.3 Hz, 1H)

^{19}F NMR (coupled, 471 MHz, $CDCl_3$) δ –133.5 (d, J = 55.4 Hz, 2F)

1-(benzofuran-2-yl)-2,2-difluoroethan-1-one (11-DFMK)

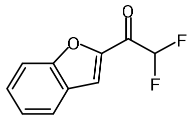

11-DFMK

Prepared from **11-TBS** using general procedure D. The crude material was purified by flash column chromatography (gradient of 1 to 3% EtOAc in hexanes) to afford **11-DFMK** (88.3 mg, 90% yield) as an off-white solid. The NMR spectra of the purified product were consistent with literature spectra.[17]

^{1}H NMR (400 MHz, CDCl$_3$) δ 7.85 (q, J = 1.3 Hz, 1H), 7.78 (dt, J = 8.0, 1.1 Hz, 1H), 7.64 (dq, J = 8.6, 1.0 Hz, 1H), 7.57 (ddd, J = 8.4, 7.1, 1.3 Hz, 1H), 7.38 (ddd, J = 8.0, 7.0, 1.0 Hz, 1H), 6.29 (t, J = 53.4 Hz, 1H)

^{13}C NMR (101 MHz, CDCl$_3$) δ 178.2 (t, J = 26.6 Hz), 156.5, 147.9, 130.1, 126.7, 124.72, 124.2, 118.8 (t, J = 4.0 Hz), 112.9, 110.2 (t, J = 252.9 Hz)

^{19}F NMR (coupled, 376 MHz, CDCl$_3$) δ –123.6 (d, J = 53.4 Hz, 2F)

IR (ATR-FTIR) υ_{max} (cm^{-1}) 1683, 1547, 1120, 1077, 1008, 854, 761

General Procedure E: Preparation of Trifluoromethyl Ketones

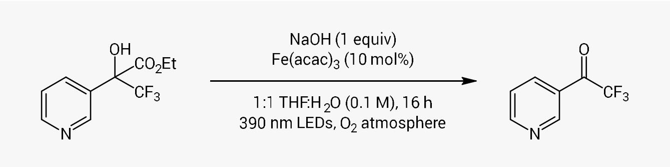

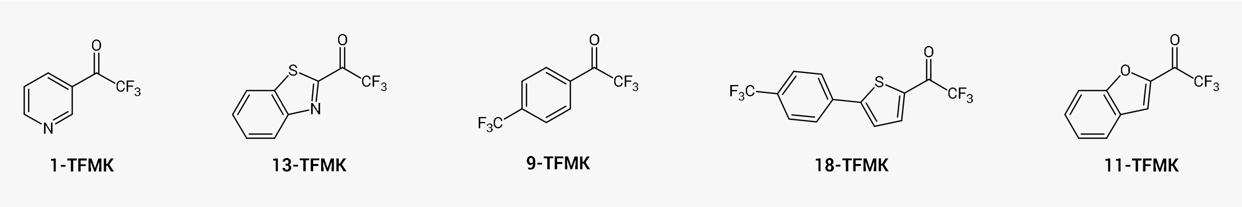

To a 20 mL vial with a stir bar was added α,α-trifluoromethyl(hydroxy) ester (0.5 mmol, 1.0 equiv) and THF:H_2O (2.5 mL of THF and 2.5 mL H_2O, 0.1 M). To this solution was added NaOH (20.0 mg, 0.5 mmol, 1.0 equiv) and iron (III) acetylacetonate (17.6 mg, 0.05 mmol, 10 mol%). The reaction mixture was then sealed with a screw-on PTFE septum cap and then sparged with a balloon of O_2 for 10 minutes, while stirring at room temperature. After removing the O_2 balloon, the reaction vial was then tightly sealed with Parafilm and electrical tape. The vial was then irradiated with two 34W Gen 1 PR160L Kessil 390 nm lamps (1 cm away) for 16 hours without the presence of a cooling fan. After 16 hours, the reaction mixture was allowed to cool to room temperature before being diluted with H_2O (5 mL) and DCM (5 mL). The resulting aqueous layer was extracted with DCM (4 × 10 mL). The collected organics were dried over Na_2SO_4 and filtered, then dry loaded onto celite. The celite was then eluted with DCM and the filtrate evaporated to afford the crude reaction mixture. The crude reaction mixture was then dry loaded onto silica gel for purification by flash column chromatography.

2,2,2-trifluoro-1-(pyridin-3-yl)ethan-1-one (1-TFMK)

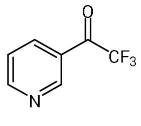

1-TFMK

Prepared from **1** using general procedure E. The crude material was purified by flash column chromatography (gradient of 40 to 50% Et_2O in chloroform) to afford **1-TFMK** (63.9 mg, 75% yield) as a pale yellow oil. The NMR spectra of the purified product were consistent with literature spectra.[18]

^{1}H NMR (400 MHz, $CDCl_3$) δ 9.30–9.24 (m, 1H), 8.91 (dd, J = 4.8, 1.7 Hz, 1H), 8.38–8.30 (m, 1H), 7.53 (ddd, J = 8.1, 4.8, 0.9 Hz, 1H)

^{13}C NMR (101 MHz, $CDCl_3$) δ 180.0 (q, J = 36.7 Hz), 155.6, 151.3 (q, J = 2.8 Hz), 137.4 (q, J = 1.9 Hz), 126.0, 124.1, 116.4 (q, J = 290.8 Hz)

^{19}F NMR (376 MHz, $CDCl_3$) δ –72.3 (s, 3F)

HRMS AMM (ESI–TOF) m/z calculated for $C_7H_5F_3NO^+$ $[M+H]^+$ 176.0318, found 176.0318

1-(benzo[*d*]thiazol-2-yl)-2,2,2-trifluoroethan-1-one (13-TFMK)

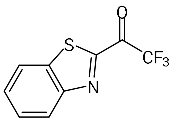

13-TFMK

Prepared from **13** using general procedure E. The crude material was purified by flash column chromatography (gradient of 30 to 50% EtOAc in hexanes) to afford **13-TFMK** (77.1 mg, 67% yield) as a white solid. *Note: In using MeOD-d_4, the compound is reported as the hydrate.*

Ketone:

HRMS AMM (ESI–TOF) m/z calculated for $C_9H_5F_3NOS^+$ $[M+H]^+$ 232.0038, found 232.0041

IR (ATR-FTIR) υ_{max} (cm^{-1}) 1673, 1456, 1175, 1048, 758, 729

Hydrate:

^{1}H NMR (500 MHz, MeOD-d$_4$) δ 8.10–7.98 (m, 1H), 7.59–7.43 (m, 1H), 4.91 (brs, 2H)

^{19}F NMR (471 MHz, MeOD-d$_4$) δ –83.4 (s, 3F)

^{13}C NMR (126 MHz, MeOD-d$_4$) δ 168.0, 154.0, 137.1, 127.8, 127.4, 124.5, 123.5 (q, J = 288.1 Hz), 123.2, 97.0 (q, J = 32.8 Hz)

HRMS AMM (ESI–TOF) m/z calculated for $C_9H_7F_3NO_2S^+$ $[M+H]^+$ 250.0144, found 250.0152

IR (ATR-FTIR) υ_{max} (cm^{-1}) 3062 br

mp: 81–83 °C

2,2,2-trifluoro-1-(4-(trifluoromethyl)phenyl)ethan-1-one (9-TFMK)

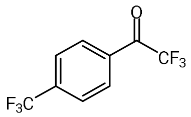

9-TFMK

Prepared from **9** using general procedure E. The crude material was purified by flash column chromatography (gradient of 5 to 15% EtOAc in hexanes) to afford **9-TFMK** (101.2 mg, 84% yield) as a yellow oil. The NMR spectra of the purified product were consistent with literature spectra.[19]

^{1}H NMR (500 MHz, $CDCl_3$) δ 8.20 (d, J = 8.6 Hz, 2H), 7.83 (d, J = 8.3 Hz, 2H)

^{19}F NMR (471 MHz, $CDCl_3$) δ –63.6 (s, 3F), –71.8 (s, 3F)

IR (ATR-FTIR) υ_{max} (cm^{-1}) 1699, 1324, 1130, 1067, 784, 668

2,2,2-trifluoro-1-(5-(4-(trifluoromethyl)phenyl)thiophen-2-yl)ethan-1-one (18-TFMK)

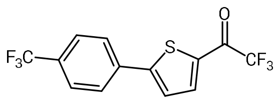

18-TFMK

Prepared from **18** using general procedure E. The crude material was purified by flash column chromatography (gradient of 10 to 15% EtOAc in hexanes) to afford **18-TFMK** (126.3 mg, 78%) yield as a pale yellow solid.

^{1}H NMR (500 MHz, $CDCl_3$) δ 7.96 (dq, J = 4.3, 1.5 Hz, 1H), 7.79 (d, J = 8.1 Hz, 2H), 7.71 (d, J = 8.0 Hz, 2H), 7.49 (d, J = 4.1 Hz, 1H)

^{13}C NMR (126 MHz, $CDCl_3$) δ 173.6 (q, J = 37.2 Hz), 154.9, 137.6 (q, J = 3.1 Hz), 136.1, 135.8, 131.8 (q, J = 32.9 Hz), 126.9, 126.5 (q, J = 3.7 Hz), 126.2, 123.9 (q, J = 272.4 Hz), 116.5 (q, J = 290.3 Hz)

^{19}F NMR (471 MHz, $CDCl_3$) δ –62.9 (s, 3F), –72.2 (s, 3F)

HRMS AMM (ESI–TOF) m/z calculated for $C13H7F6O2S^-$ $[M–H]^-$ 341.0076, found 341.0066

IR (ATR-FTIR) υ_{max} (cm^{-1}) 1685, 1448, 1323, 1166, 1112, 1064, 1011, 871, 735, 669

mp: 83–86 °C

1-(benzofuran-2-yl)-2,2,2-trifluoroethan-1-one (11-TFMK)

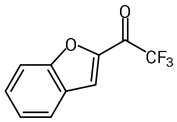

11-TFMK

Prepared from **11** using general procedure E. The crude material was purified by flash column chromatography (10% EtOAc in hexanes) to afford **11-TFMK** (86.7 mg, 81% yield) as a pale yellow solid. The NMR spectra of the purified product were consistent with literature spectra.[19]

^{1}H NMR (400 MHz, $CDCl_3$) δ 7.86 (p, J = 1.3 Hz, 1H), 7.80 (dt, J = 8.0, 1.1 Hz, 1H), 7.65 (dq, J = 8.6, 1.0 Hz, 1H), 7.60 (ddd, J = 8.5, 7.0, 1.3 Hz, 1H), 7.40 (ddd, J = 8.0, 6.9, 1.2 Hz, 1H)

^{13}C NMR (126 MHz, $CDCl_3$) δ 170.8 (q, J = 37.8 Hz), 157.0, 146.6, 130.7, 126.5, 125.0, 124.4, 120.3 (q, J = 2.9 Hz), 116.4 (q, J = 289.3 Hz), 113.0

^{19}F NMR (471 MHz, $CDCl_3$) δ –73.1 (s, 3F)

IR (ATR-FTIR) υ_{max} (cm^{-1}) 1688, 1544, 1255, 1189, 1148, 1126, 1005, 743, 730

mp: 47–50 °C (lit.[19] mp 46–47 °C)

General Procedure F: Preparation of Chlorodifluoromethyl Silyl Ethers

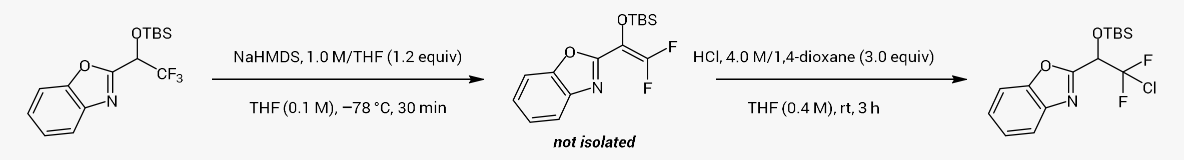

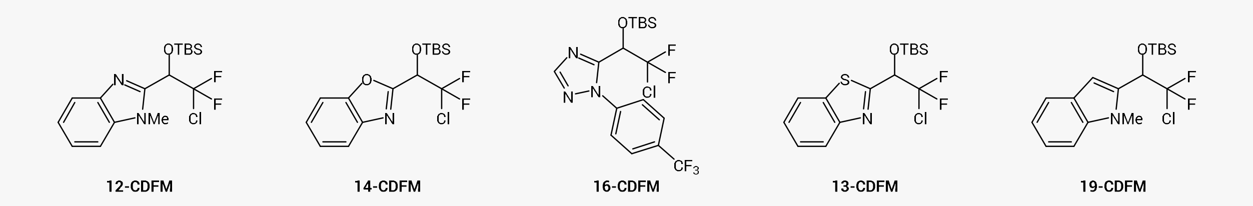

To a flame-dried 20 mL vial with a stir bar was added silyl ether (0.5 mmol, 1.0 equiv) and THF (5.0 mL, 0.1 M). The vial was tightly sealed with a screw-on PTFE septum cap, cooled to –78 °C, and purged with a constant flow of nitrogen for 15 minutes. To the vial was added a solution of sodium bis(trimethylsilyl)amide (0.6 mL, 0.6 mmol, 1.2 equiv, 1.0 M/THF) dropwise. The reaction was then stirred for 30 minutes under nitrogen atmosphere, maintaining a temperature of –78 °C. The reaction mixture was diluted with H_2O (5 mL) and Et_2O (5 mL), then transferred to a separatory funnel. The aqueous layer was extracted with Et_2O (4 × 5 mL) and the organic extractions were collected and washed with brine. The organics were dried over Na_2SO_4, filtered, and evaporated to afford the crude reaction mixture. The crude material was then dry loaded onto silica gel and eluted through a short plug of silica gel with a mixture of 1:1 hexanes:DCM. After concentrating under reduced pressure, the material was subjected to the next step without any further purification.

Note: The tert-butyldimethylsilyl difluoro enol ether products were observed to decompose, primarily to the self-aldol product, at room temperature but could be stored neat at –4 °C for at least one month without decomposition.

To an oven-dried 8 mL vial and a stir bar was added the silyl difluoro enol ether product and THF (1.25 mL, 0.4 M). To the reaction vial was added HCl in 1,4-dioxane (375 µL, 1.5 mmol, 3.0 equiv, 4.0 M/dioxane). The vial was tightly sealed with a screw-on PTFE septum cap. All reagents were added open to air and no further considerations were made to purge the solution. The reaction was stirred at room temperature and monitored for starting material consumption by TLC. Upon completion, the reaction was neutralized with saturated $NaHCO_3$ (2 mL), diluted with H_2O (2 mL) and Et_2O (2 mL), then transferred to a separatory funnel. The aqueous layer was extracted with Et_2O (5 × 5 mL) and the organic extractions were collected and washed with brine. The organics were dried over Na_2SO_4, filtered, and evaporated to afford the crude reaction mixture. The crude material was then dry loaded onto silica gel for purification by flash column chromatography.

2-(1-((*tert*-butyldimethylsilyl)oxy)-2-chloro-2,2-difluoroethyl)-1-methyl-1*H*-benzo[*d*]imidazole (12-CDFE)

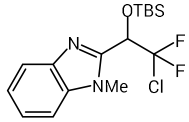

12-CDFE

Prepared from **12-TBS** using general procedure F. The crude material was purified by flash column chromatography (15% EtOAc in hexanes) to afford **12-CDFE** (131.7 mg, 72% yield) as a colorless oil.

^{1}H NMR (400 MHz, CDCl$_3$) δ 7.79 (dt, J = 7.7, 0.9 Hz, 1H), 7.44–7.39 (m, 1H), 7.36 (ddd, J = 8.0, 6.9, 1.4 Hz, 1H), 7.31 (ddd, J = 8.4, 7.0, 1.5 Hz, 1H), 5.59 (t, J = 10.2 Hz, 1H), 3.99 (s, 3H), 0.90 (s, 9H), 0.21 (s, 3H), –0.02 (s, 3H)

^{13}C NMR (101 MHz, CDCl$_3$) δ 147.1, 142.1, 137.0, 127.8 (t, J = 298.2 Hz), 123.8, 122.8, 120.5, 109.8, 31.6 (t, J = 3.5 Hz), 25.6, 18.3, –5.2, –5.4

^{19}F NMR (coupled, 376 MHz, CDCl$_3$) δ –62.2 (dd, J = 165.4, 9.6 Hz, 1F), –62.8 (dd, J = 165.4, 9.6 Hz, 1F)

HRMS AMM (ESI–TOF) m/z calculated for $C_{16}H_{24}ClF_2N_2OSi^+$ $[M+H]^+$ 361.1309, found 361.1307

IR (ATR-FTIR) υ_{max} (cm^{-1}) 1111, 1068, 978, 836, 782, 739

2-(1-((*tert*-butyldimethylsilyl)oxy)-2-chloro-2,2-difluoroethyl)benzo[*d*]oxazole (14-CDFE)

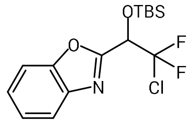

14-CDFE

Prepared from **14-TBS** using general procedure F. The crude material was purified by flash column chromatography (2.5% EtOAc in hexanes) to afford **14-CDFE** (136.7 mg, 78% yield) as a colorless oil.

^{1}H NMR (400 MHz, $CDCl_3$) δ 7.80 (dd, J = 7.0, 2.0 Hz, 1H), 7.61 (dd, J = 7.5, 2.1 Hz, 1H), 7.41 (pd, J = 7.4, 1.5 Hz, 2H), 5.37 (t, J = 7.5 Hz, 1H), 0.91 (s, 9H), 0.18 (s, 3H), 0.04 (s, 3H)

^{13}C NMR (101 MHz, $CDCl_3$) δ 159.7 (t, J = 2.4 Hz), 151.0, 140.6, 126.8 (t, J = 297.9 Hz), 126.3, 125.0, 121.0, 111.3, 73.7 (t, J = 29.9 Hz), 25.5, 18.3, –5.20, –5.24

^{19}F NMR (coupled, 376 MHz, $CDCl_3$) δ –63.4 (t, J = 8.0 Hz, 2F)

HRMS AMM (ESI–TOF) m/z calculated for $C_{15}H_{21}ClF_2NO_2Si^+$ $[M+H]^+$ 348.0993, found 348.0997

IR (ATR-FTIR) υ_{max} (cm^{-1}) 1130, 1040, 979, 835, 781, 744

5-(1-((*tert*-butyldimethylsilyl)oxy)-2-chloro-2,2-difluoroethyl)-1-(4-(trifluoromethyl)phenyl)-1*H*-1,2,4-triazole (16-CDFE)

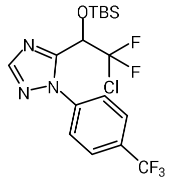

16-CDFE

Prepared from **16-TBS** using general procedure F. The crude material was purified by flash column chromatography (gradient of 10 to 25% EtOAc in hexanes) to afford **16-CDFE** (183.1 mg, 83% yield) as a white solid.

^{1}H NMR (500 MHz, CDCl$_3$) δ 8.13 (s, 1H), 7.81 (d, J = 8.1 Hz, 2H), 7.65 (d, J = 7.8 Hz, 2H), 5.37 (t, J = 8.3 Hz, 1H), 0.80 (s, 9H), 0.02 (s, 3H), –0.11 (s, 3H)

^{13}C NMR (126 MHz, CDCl$_3$) δ 152.2, 150.4, 140.2, 132.1 (q, J = 33.2 Hz), 127.0 (t, J = 345.9 Hz), 126.7 (q, J = 3.7 Hz), 126.4, 122.4, 72.0 (t, J = 29.6 Hz), 25.4, –4.9, –5.3

^{19}F NMR (coupled, 471 MHz, CDCl$_3$) δ –62.0 (dd, J = 8.5, 3.0 Hz, 2F), –62.7 (s, 3F)

HRMS AMM (ESI–TOF) m/z calculated for $C_{17}H_{22}ClF_5N_3OSi^+$ $[M+H]^+$ 442.1135, found 442.1145

IR (ATR-FTIR) υ_{max} (cm^{-1}) 1138, 1120, 1108, 1069, 840, 782

mp: 72–74 °C

2-(1-((*tert*-butyldimethylsilyl)oxy)-2-chloro-2,2-difluoroethyl)benzo[*d*]thiazole (13-CDFE)

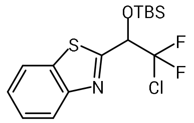

13-CDFE

Prepared from **13-TBS** using general procedure F. The crude material was purified by flash column chromatography (gradient of 20 to 30% DCM in hexanes) to afford **13-CDFE** (136.5 mg, 75% yield) as a pale yellow oil.

^{1}H NMR (400 MHz, CDCl$_3$) δ 8.08 (dq, J = 8.1, 0.6 Hz, 1H), 7.94 (ddd, J = 8.0, 1.2, 0.6 Hz, 1H), 7.52 (ddd, J = 8.3, 7.1, 1.3 Hz, 1H), 7.44 (ddd, J = 8.4, 7.1, 1.3 Hz, 1H), 5.48 (t, J = 6.5 Hz, 1H), 0.97 (s, 9H), 0.21 (s, 3H), 0.08 (s, 3H)

^{13}C NMR (101 MHz, CDCl$_3$) δ 167.6 (d, J = 3.6 Hz), 152.9, 135.4, 127.5 (t, J = 298.3 Hz), 126.4, 125.9, 123.9, 122.0, 25.6, 18.3, –5.0, –5.1

^{19}F NMR (coupled, 376 MHz, CDCl$_3$) δ –61.6 (dd, J = 165.6, 7.3 Hz, 1F), –63.4 (dd, J = 165.5, 6.3 Hz, 1F)

HRMS AMM (ESI–TOF) m/z calculated for $C_{15}H_{21}ClF_2NOSSi^+$ $[M+H]^+$ 364.0764, found 364.0771

IR (ATR-FTIR) υ_{max} (cm^{-1}) 1136, 1029, 971, 837, 758, 729

2-(1-((*tert*-butyldimethylsilyl)oxy)-2-chloro-2,2-difluoroethyl)-1-methyl-1*H*-indole (19-CDFE)

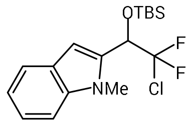

19-CDFE

Prepared from **19-TBS** using a modified version of general procedure F. The addition of NaHMDS was conducted at 0 °C and allowed to warm to room temperature for 1 hour. The general procedure was then followed as written. The crude material was purified by flash column chromatography (15% EtOAc in hexanes) to afford **19-CDFE** (159.8 mg, 89% yield) as a colorless oil.

^{1}H NMR (500 MHz, $CDCl_3$) δ 7.65 (d, J = 7.9 Hz, 1H), 7.41–7.35 (m, 1H), 7.30 (ddd, J = 8.3, 6.9, 1.2 Hz, 1H), 7.16 (ddd, J = 8.0, 7.0, 1.0 Hz, 1H), 6.67 (s, 1H), 5.29 (dd, J = 9.8, 8.3 Hz, 1H), 3.88 (s, 3H), 0.95 (s, 9H), 0.19 (s, 3H), 0.00 (s, 3H)

^{13}C NMR (126 MHz, $CDCl_3$) δ 138.6, 128.8 (t, J = 344.1 Hz), 128.7, 127.0, 122.5, 121.1, 119.9, 109.5, 104.8, 74.1 (t, J = 34.5 Hz), 31.4 (t, J = 3.2 Hz), 25.6, –4.9, –5.3

^{19}F NMR (coupled, 376 MHz, $CDCl_3$) δ –61.9 (dd, J = 162.9, 8.5 Hz), –63.5 (dd, J = 162.6, 9.6 Hz)

HRMS AMM (ESI–TOF) m/z calculated for $C_{17}H_{25}ClF_2NOSi^+$ $[M+H]^+$ 360.1357, found 360.1357

IR (ATR-FTIR) υ_{max} (cm^{-1}) 1100, 1028, 970, 780, 748

Preparation of Monofluoromethyl Ketone (7-MFMK)

6-(1-((*tert*-butyldimethylsilyl)oxy)-2,2-difluoroethyl)-*N*,*N*-dimethylquinazolin-4-amine (7-DFTBS)

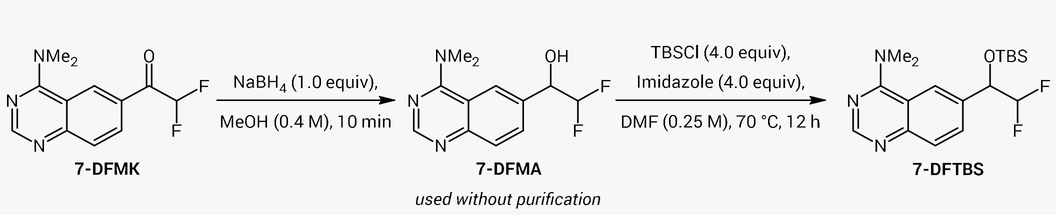

To an 8-mL vial equipped with a stir bar was added **7-DFMK** (125.5 mg, 0.5 mmol, 1.0 equiv) and MeOH (1.25 mL, 0.4 M). The reaction mixture was cooled to 0 °C before adding $NaBH_4$ (18.9 mg, 0.5 mmol, 1.0 equiv) in one portion. The reaction mixture was sealed with a screw-on PTFE septum cap before stirring for 10 minutes at room temperature. The reaction mixture was then diluted with H_2O (2 mL) and EtOAc (2 mL), at room temperature, and then transferred to a separatory funnel. The aqueous layer was extracted with EtOAc (4 × 10 mL) and the organic extractions were collected, dried over Na_2SO_4, filtered, and evaporated. **7-DFMA** was used in the next step without further purification.

7-DFMA was then transferred with DMF (1.8 mL, 0.25 M) to an 8-mL vial equipped with a stir bar. *tert*-butyldimethylsilyl chloride (527.5 mg, 3.5 mmol, 4.0 equiv) and imidazole (238.2 mg, 3.5 mmol, 4.0 equiv) were then added. The reaction mixture was sealed with a screw-on PTFE septum cap before stirring for 12 h at 70 °C. The reaction mixture was then allowed to cool to room temperature before diluting with H_2O (4 mL) and EtOAc (1 mL). The reaction mixture was then transferred to a separatory funnel and the aqueous layer was extracted with EtOAc (4 × 10 mL). The organic extractions were collected, dried over Na_2SO_4, filtered, and evaporated to afford the crude reaction mixture. The crude reaction mixture was then dry loaded onto silica and purified by flash column chromatography (40% EtOAc in hexanes) to afford **7-DFTBS** (174.4 mg, 95% yield) as an orange oil.

^{1}H NMR (500 MHz, $CDCl_3$) δ 8.64 (s, 1H), 8.03 (s, 1H), 7.85 (d, J = 8.6 Hz, 1H), 7.71 (d, J = 8.7 Hz, 1H), 5.62 (td, J = 4.7, 51.3 Hz, 1H), 4.85 (td, J = 9.6, 4.7 Hz, 1H), 3.36 (s, 6H), 0.89 (s, 9H), 0.12 (s, 3H), –0.02 (s, 3H)

^{13}C NMR (126 MHz, $CDCl_3$) δ 163.7, 154.4, 152.0, 133.5 (d, J = 4.6 Hz), 131.0, 128.5, 124.5, 116.8 (t, J = 263.2 Hz), 115.9 (d, J = 3.1 Hz), 74.6 (t, J = 24.1 Hz), 42.0, 25.7, 18.3, –4.7, –4.9

^{19}F NMR (coupled, 471 MHz, $CDCl_3$) δ –124.7 (ddd, J = 280.0, 56.5, 9.9 Hz), –127.5 (ddd, J = 279.8, 55.7, 8.9 Hz)

HRMS AMM (ESI–TOF) m/z calculated for $C_{18}H_{28}F_2N_3OSi^+$ $[M+H]^+$ 368.1964, found 368.1979

IR (ATR-FTIR) υ_{max} (cm^{-1}) 2927, 1567, 1535, 1101, 1060, 835, 778

1-(4-(dimethylamino)quinazolin-6-yl)-2-fluoroethan-1-one (7-MFMK)

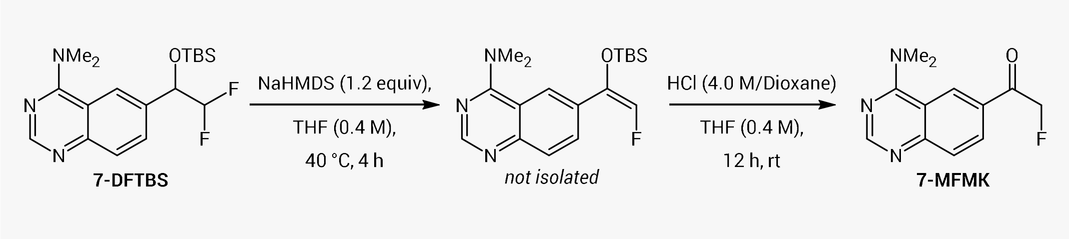

To a flame-dried 8-mL vial with a stir bar was added **7-DFTBS** (174.4 mg, 0.47 mmol, 1.0 equiv) and anhydrous THF (1.2 mL, 0.4 M). The vial was tightly sealed with a screw-on PTFE septum cap and purged with a constant flow of nitrogen for 15 minutes. To the vial was added a solution of sodium bis(trimethylsilyl)amide (0.56 mL, 0.56 mmol, 1.2 equiv, 1.0M/THF) dropwise at room temperature. The reaction was then stirred for 4 hours under nitrogen atmosphere at 40 °C. The reaction mixture was then cooled to room temperature, diluted with H_2O (2 mL) and EtOAc (2 mL), and then transferred to a separatory funnel. The aqueous layer was extracted with EtOAc (4 × 10 mL) and the organic extractions were collected, dried over Na_2SO_4, filtered, and evaporated to afford the crude reaction mixture. The crude reaction mixture was transferred using DCM to an 8-mL vial equipped with a stir bar. The crude reaction mixture was then concentrated to remove any remaining DCM. To this vial was then added THF (1.2 mL, 0.4 M) and HCl (1.2 mL, 4.7 mmol, 10.0 equiv, 4.0 M/dioxane). The reaction mixture was then sealed with a screw-on PTFE septum and allowed to vigorously stir (1000 RPM) for 12 h at room temperature. The reaction mixture was then brought to pH = 7 using a saturated $NaHCO_3$ solution before transferring to a separatory funnel. The aqueous layer was extracted with EtOAc (4 × 10 mL) and the organic extractions were collected, dried over Na_2SO_4, filtered, and evaporated to afford the crude reaction mixture. The crude reaction mixture was then dry loaded onto silica and purified by flash column chromatography (70% EtOAc in hexanes) to afford **7-MFMK** (90.9 mg, 83% yield, 78% overall yield from **7-DFMK**) as a white solid.

^{1}H NMR (500 MHz, $CDCl_3$) δ 8.75 (d, J = 2.0 Hz, 1H), 8.66 (s, 1H), 8.12 (dd, J = 8.8, 1.9 Hz, 1H), 7.88 (d, J = 8.8 Hz, 1H), 5.53 (d, J = 47.1 Hz, 2H), 3.46 (s, 6H)

^{13}C NMR (126 MHz, $CDCl_3$) δ 192.6 (d, J = 16.2 Hz), 163.7, 156.2, 155.2, 130.2 (d, J = 2.6 Hz), 129.5, 128.9, 128.4 (d, J = 5.1 Hz), 115.3, 84.3 (d, J = 184.2 Hz), 42.2

^{19}F NMR (coupled, 471 MHz, $CDCl_3$) δ –227.5 (t, J = 47.1 Hz)

HRMS AMM (ESI–TOF) m/z calculated for $C_{12}H_{13}FN_3O^+$ $[M+H]^+$ 234.1037, found 234.1049

IR (ATR-FTIR) υ_{max} (cm^{-1}) 2920, 1701, 1567, 1348, 979, 866, 795

mp: 164–166 °C

Procedure for the Detection of Chloroethane

To an oven-dried 8 mL vial with a stir bar was added **1** (49.8 mg, 0.2 mmol, 1.0 equiv), imidazole (27.2 mg, 0.4 mmol, 2.0 equiv), *tert*-butyldimethylsilyl chloride (60.3 mg, 0.4 mmol, 2.0 equiv), and DMF (1 mL, 0.2M). The vial was tightly sealed with a screw-on PTFE septum cap. The vial was then heated to 130 °C while stirring at 1000 RPM. After 14 hours, the vial was cooled to –78 °C. The cold reaction mixture was aliquoted into a syringe through the septum cap, charged into an NMR tube containing 700 µL of $CDCl_3$, and the sample was submitted for ^1H NMR analysis. The collected NMR spectrum contained signals consistent with a literature spectrum of chloroethane in $CDCl_3$.[21]

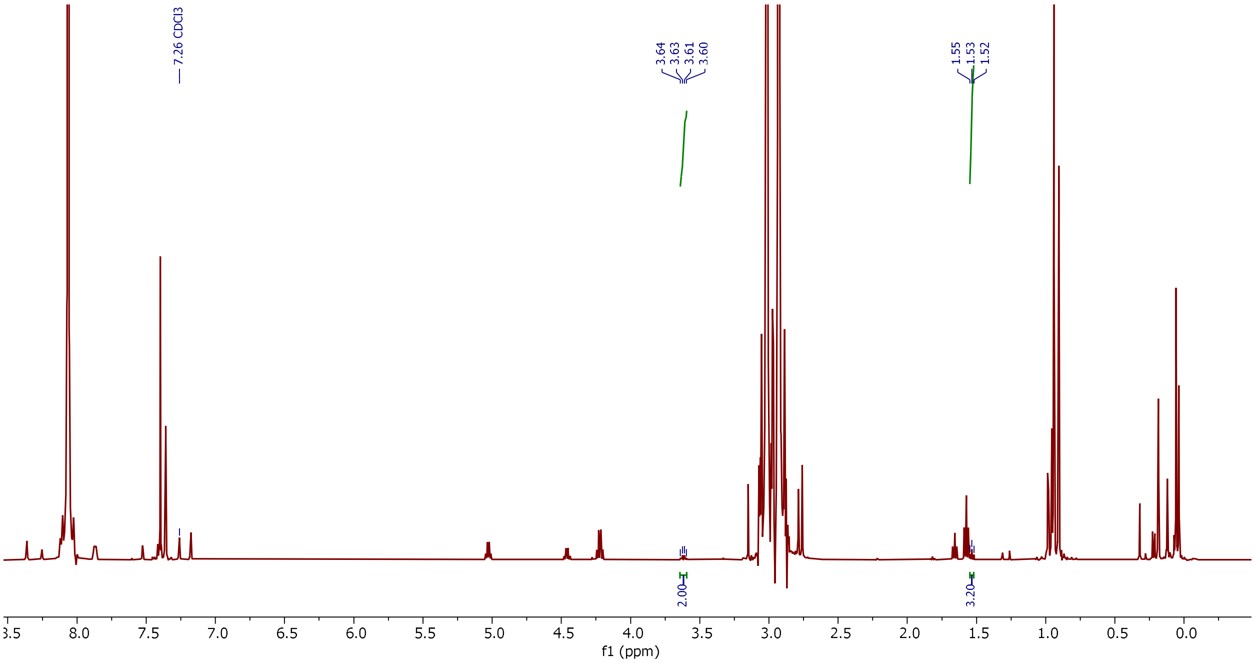

References

1. Xu, Z.; Li, H.; Ren, Z.; Du, W.; Xu, W.; Lang, J. $Cu(OAc)_2$ H_2O-catalyzed N-arylation of nitrogen-containing heterocycles. *Tetrahedron* **2011**, 67, 5282.
2. Ang, W.; Low, C.; Teo, Y. Ligand-Free Copper(I) Chloride Catalyzed N-Arylation of 1,2,4-Triazole with Aryl Bromides. *Synlett* **2023**, vol. 35, #7, p. 821–825.
3. Park, J.; Chang, S. Comparative Catalytic Activity of Group 9 [Cp*MIII] Complexes: Cobalt-Catalyzed C-H Amidation of Arenes with Dioxazolones as Amidating Reagents. *Angewandte Chemie International Edition* **2015,** 54:14103-14107.
4. Bhadra, S.; Matheis, C.; Katayev, D.; Gooβen, L. Copper-Catalyzed Dehydrogenative Coupling of Arenes with Alcohols. *Angewandte Chemie International Edition* **2013,** 52:9279-9283.
5. Monovich, L.G., et al. Discovery of Potent, Selective, and Orally Active Carboxylic Acid Based Inhibitors of Matrix Metalloproteinase-13. *Journal of Medicinal Chemistry* **2009** 52 (11), 3523–3538.
6. Liang, Y.; Steinbock, R.; Yang, L.; Ackermann, L. Continuous Visible-Light Photoflow Approach for a Manganese-Catalyzed (Het)Arene C−H Arylation. *Angewandte Chemie International Edition* **2018,** 57:10625-10629.
7. Kucher, O.; et al. Enzyme-Catalyzed Kinetic Resolution of 2,2,2-Trifluoro-1-(heteroaryl)ethanols: Experimental and Docking Studies. *European Journal of Organic Chemistry* **2014**: 7692-7698.
8. Aït-Mohand, S.; Takechi, N.; Médebielle, M.; Dolbier, W. Nucleophilic Trifluoromethylation Using Trifluoromethyl Iodide. A New and Simple Alternative for the Trifluoromethylation of Aldehydes and Ketones. *Organic Letters* **2001**, 3, 26, 4271–4273.
9. Kelly, C.; Mercadante, M.; Hamlin, T.; Fletcher, M.; Leadbeater, N. Oxidation of α-Trifluoromethyl Alcohols Using a Recyclable Oxoammonium Salt. *Journal of Organic Chemistry* **2012**, 77, 18, 8131–8141.
10. Burnett, Duane. Compounds, Compositions, and Methods of Use. WO**2018**/119395.
11. van der Born, D.; Herscheid, J.; Orru, R.; Vugts, D. Efficient synthesis of [18F]trifluoromethane and its application in the synthesis of PET tracers. *Chemical Communications*, **2013**, 49, 4018-4020.
12. Lo, W.; Hunter, E.; Watson, G.; Patny, A.; Iyer, P.; Boruwa, J. Pesticidal Compositions and Processes Related Thereto. US**2014**.0171312A1.
13. Baumann, M.; Baxendale, I.; Martin, L.; Ley, S. Development of fluorination methods using continuous-flow microreactors. *Tetrahedron* **2009**, 65, 6611.
14. Zheng, G.; Liu, H.; Wang, M. Copper-Catalyzed Aerobic Oxidation of Azinylmethanes for Access to Trifluoromethylazinylols. *Chinese Journal of Chemistry* **2016,** 34, 5, 519–523.
15. Fujii, S.; Maki, Y.; Kimoto, H.; Cohen, L. Thermal condensation of substituted imidazoles with trifluoroacetaldehyde. *Journal of Fluorine Chemistry* **1986**, 32, 3, 329–343
16. Leng, D.; Black, C.; Pattison, G. One-pot synthesis of difluoromethyl ketones by a difluorination/fragmentation process. *Organic and Biomolecular Chemistry* **2016**, vol. 14, # 5, 1531–1535.
17. Zhou, G.; Guo, Z.; Liu, S.; Shen, X. Divergent Synthesis of Fluoroalkyl Ketones through Controlling the Reactivity of Organoboronate Complexes. *Journal of the American Chemical Society* **2024**, 146, 6, 4026–4035.R
18. Sauter, F.; Stanetty, P.; Ramer, W.; Sittenhaler, W. Fungizide Pyridinderivate, 4. Mitt.: α-Trifluormethyl-3-pyridinmethanole. *Monatshefte für Chemie,* **1991**, vol. 122, 879–885.
19. Veth, L.; Windhorst, A. Vugts, D. Synthesis of 18F-labelled aryl trifluoromethyl ketones with improved molar activity. *Chemical Communications*, **2024**, vol. 60, # 53, p. 6801–6804.
20. Liu, X.; et. al. Trifluoromethylation of Benzoic Acids: An Access to Aryl Trifluoromethyl Ketones. *Organic Letters* **2021**, 23, 12, 4930–4934.
21. Xu, L.; Mei, C.; Lu, W. Visible-Light-Driven Oxidative Chlorination of Alkyl sp3 C–H Bonds with HCl/Air at Room Temperature. *Synthesis* **2024**; 56(11): 1793–1798.

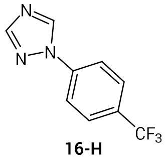

16-H

^1H, ^{13}C, and ^{19}F NMR spectra of all compounds

1-(4-(trifluoromethyl)phenyl)-1*H*-1,2,4-triazole (16-H)

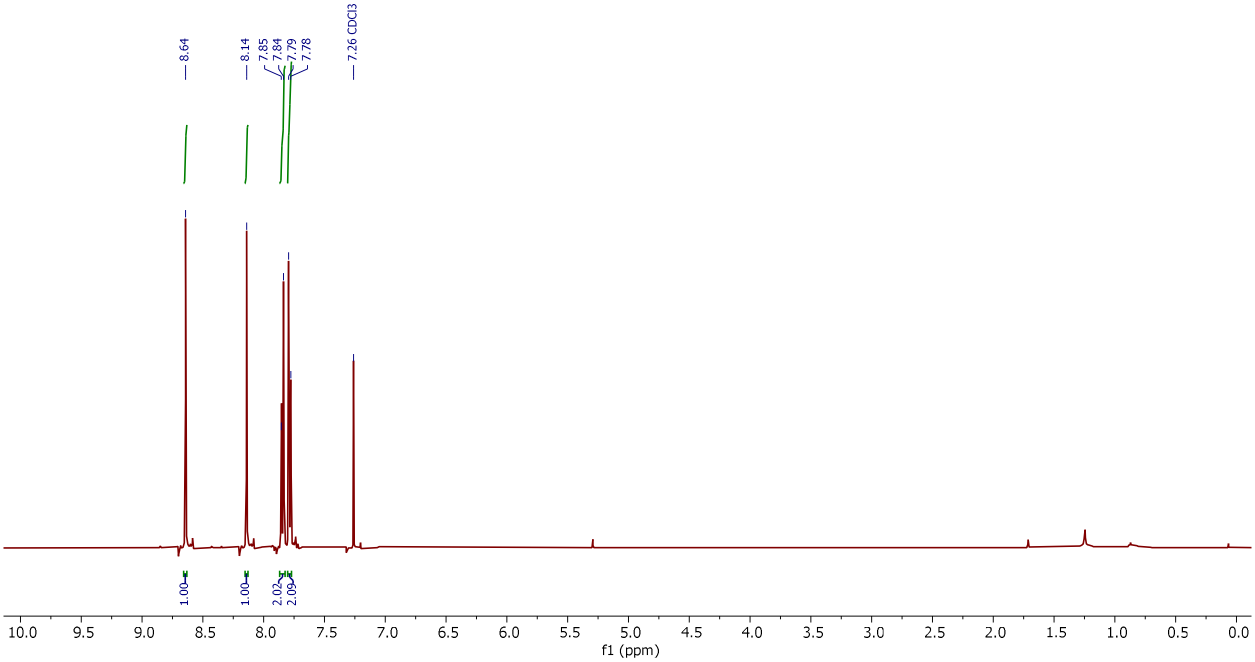

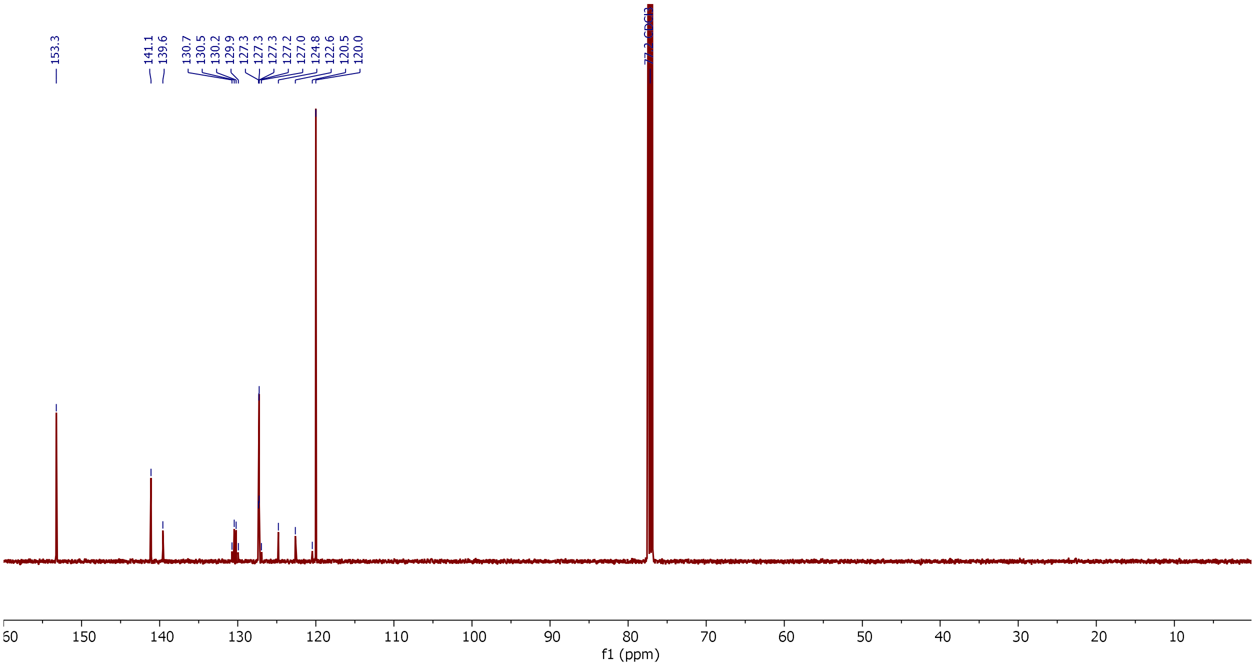

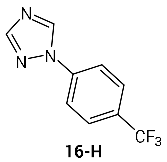
N
N
N
CF3

16-H

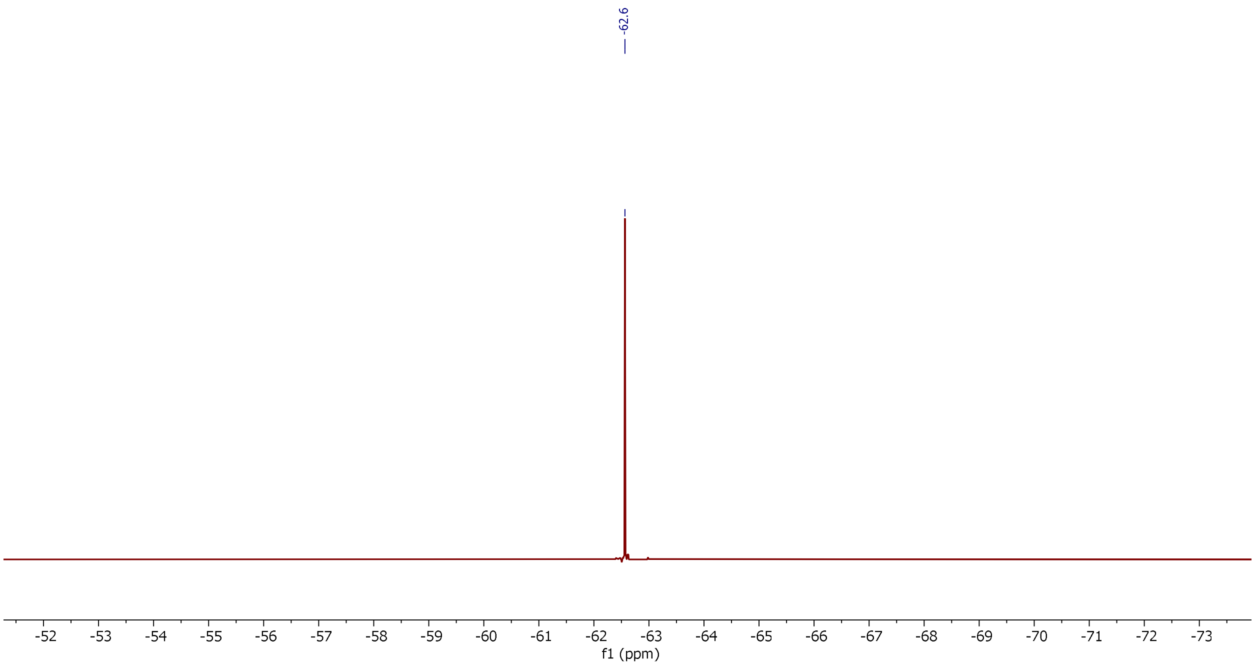
-62.6
-52
-53
-54
-55
-56
-57
-58
-59
-60
-61
-62
-63
-64
-65
-66
-67
-68
-69
-70
-71
-72
-73
f1 (ppm)

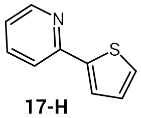

17-H

2-(thiophen-2-yl)pyridine (17-H)

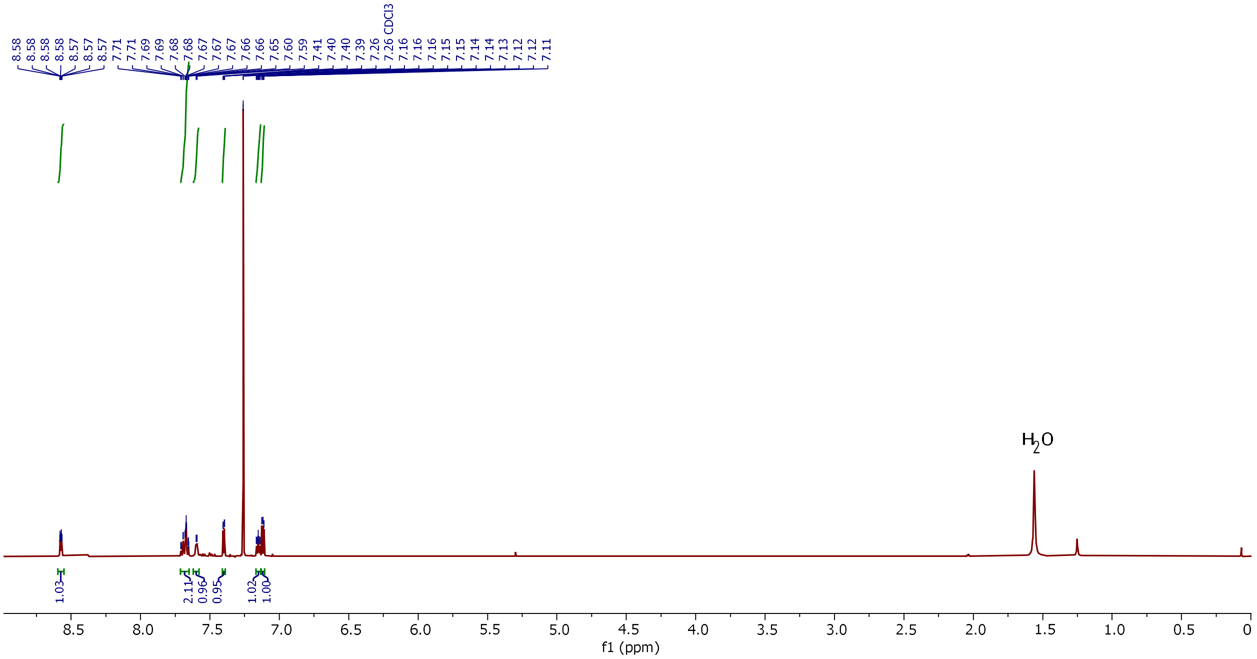

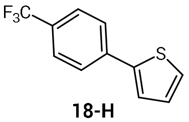

18-H

2-(4-(trifluoromethyl)phenyl)thiophene (18-H)

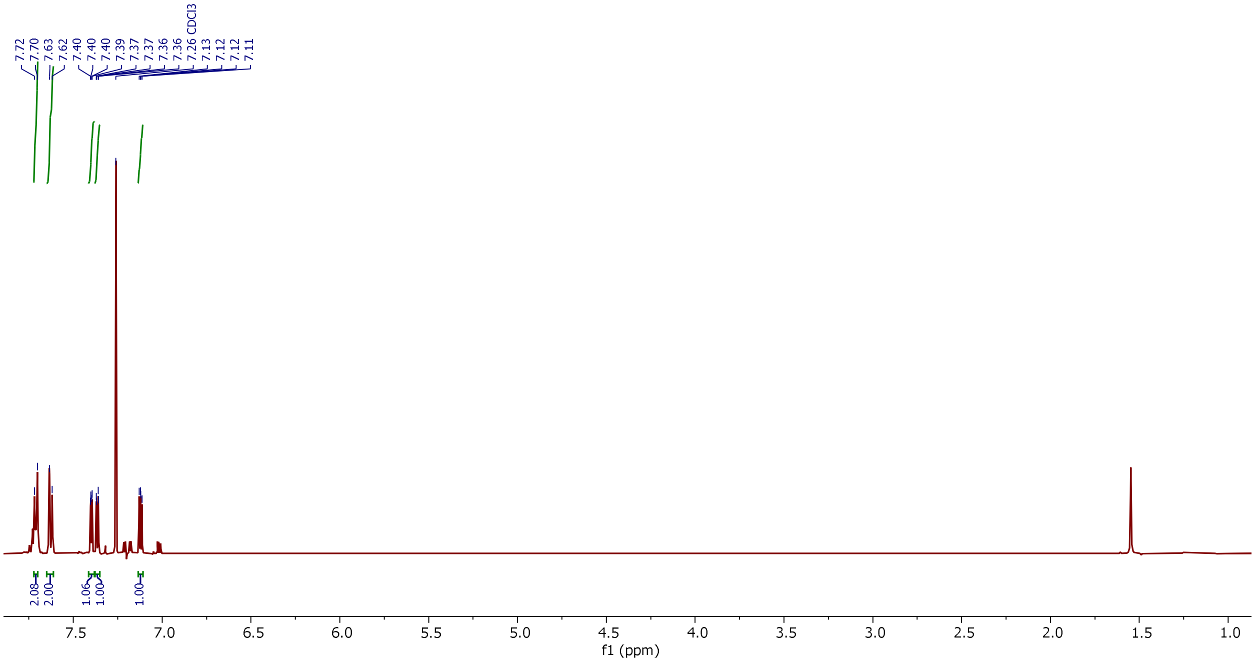

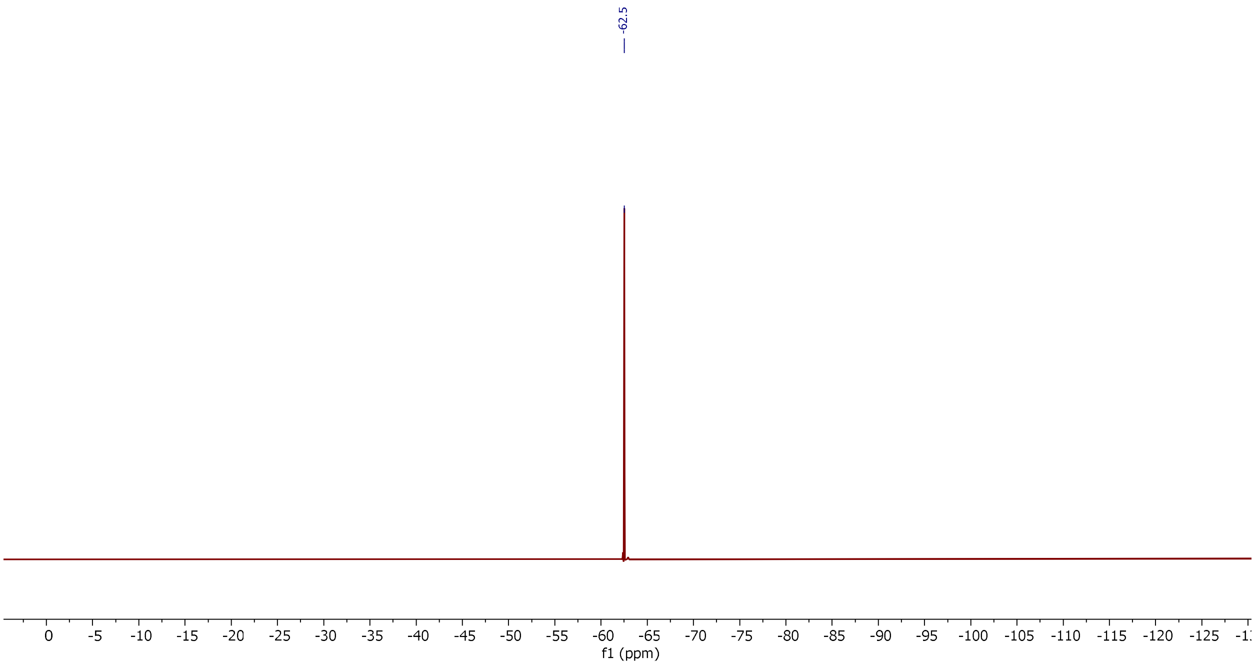

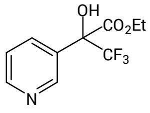

1

ethyl 3,3,3-trifluoro-2-hydroxy-2-(pyridin-3-yl)propanoate (1)

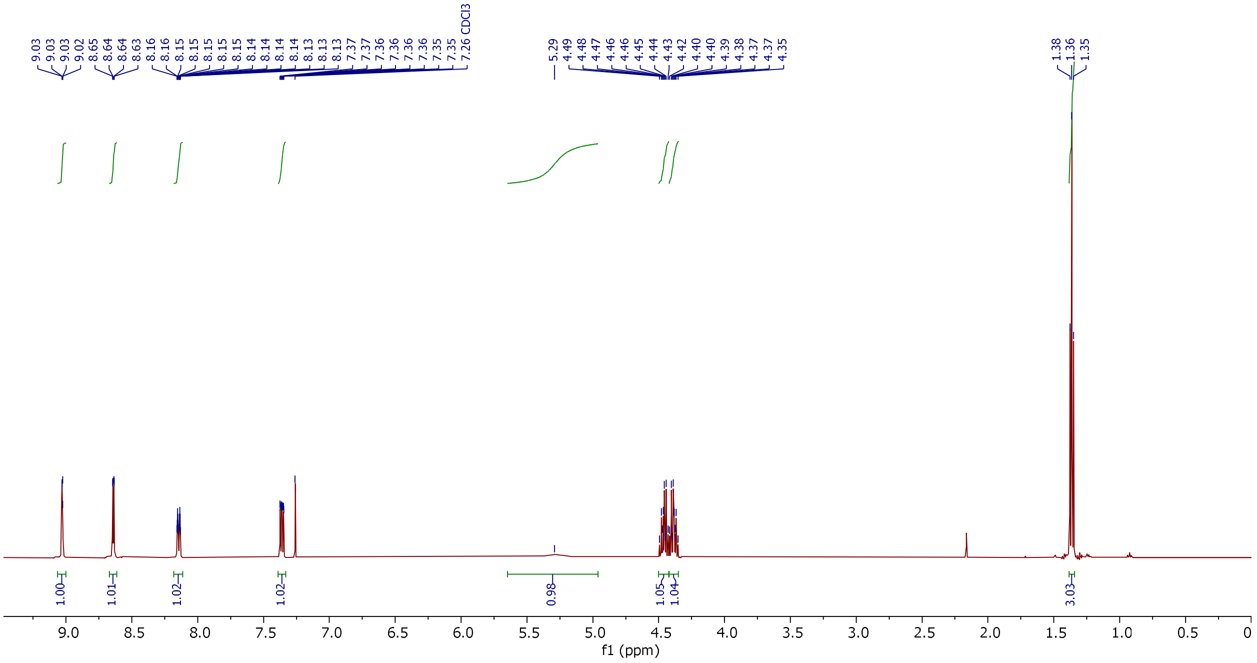

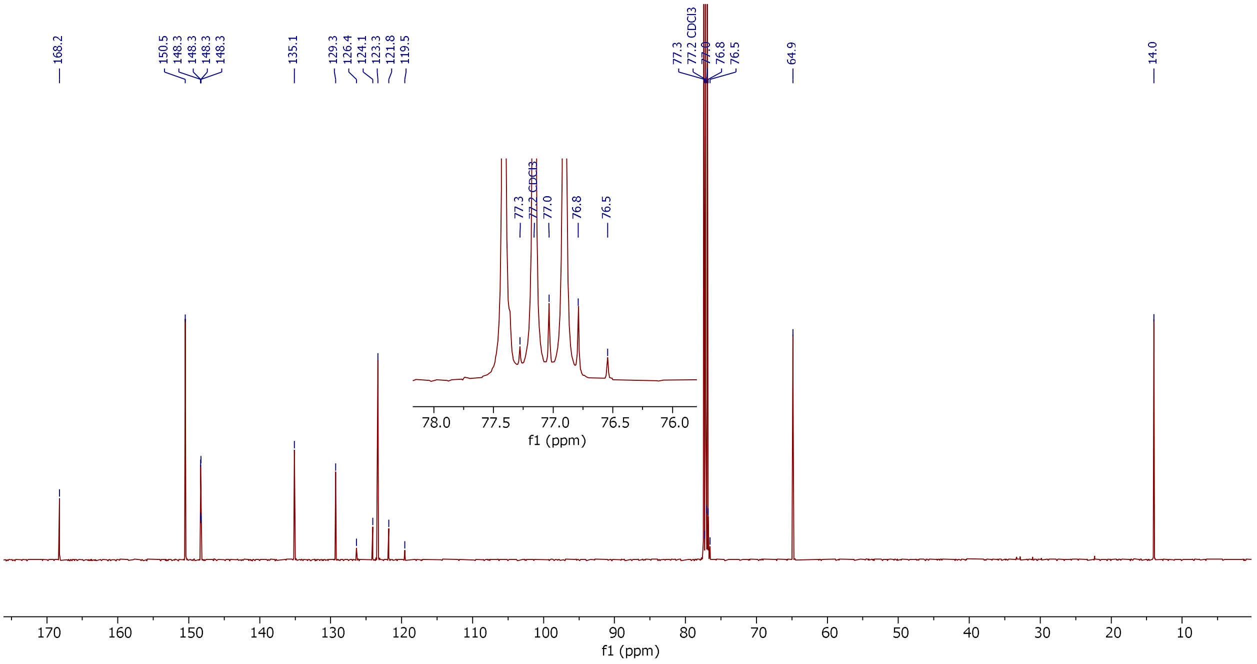

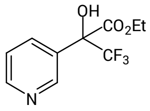
OH
CO2Et
CF3
N

1

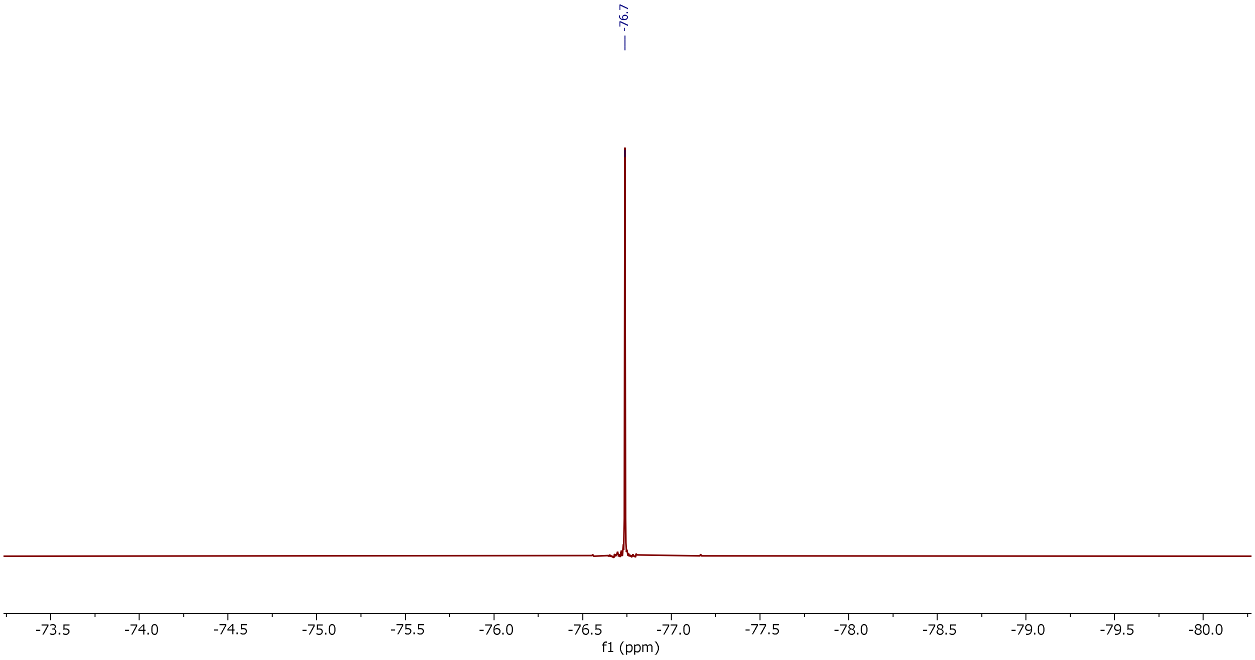
-76.7
-73.5
-74.0
-74.5
-75.0
-75.5
-76.0
-76.5
-77.0
-77.5
-78.0
-78.5
-79.0
-79.5
-80.0
f1 (ppm)

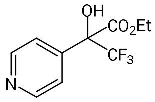

3

ethyl 3,3,3-trifluoro-2-hydroxy-2-(pyridin-4-yl)propanoate (3)

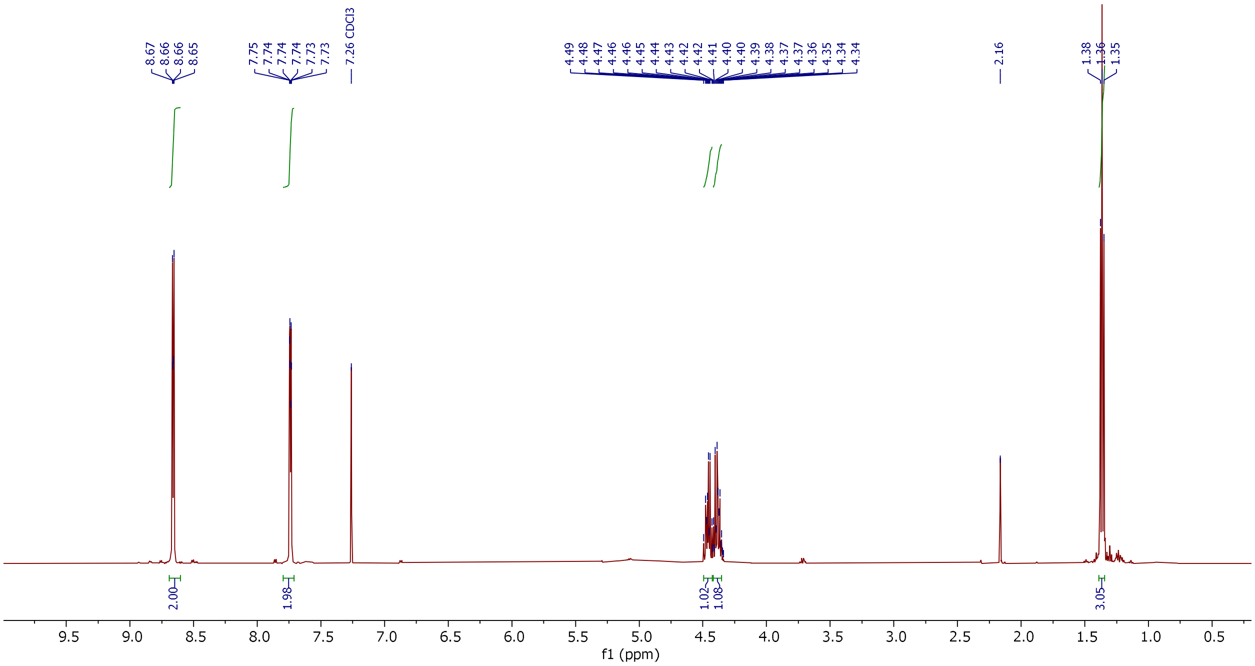

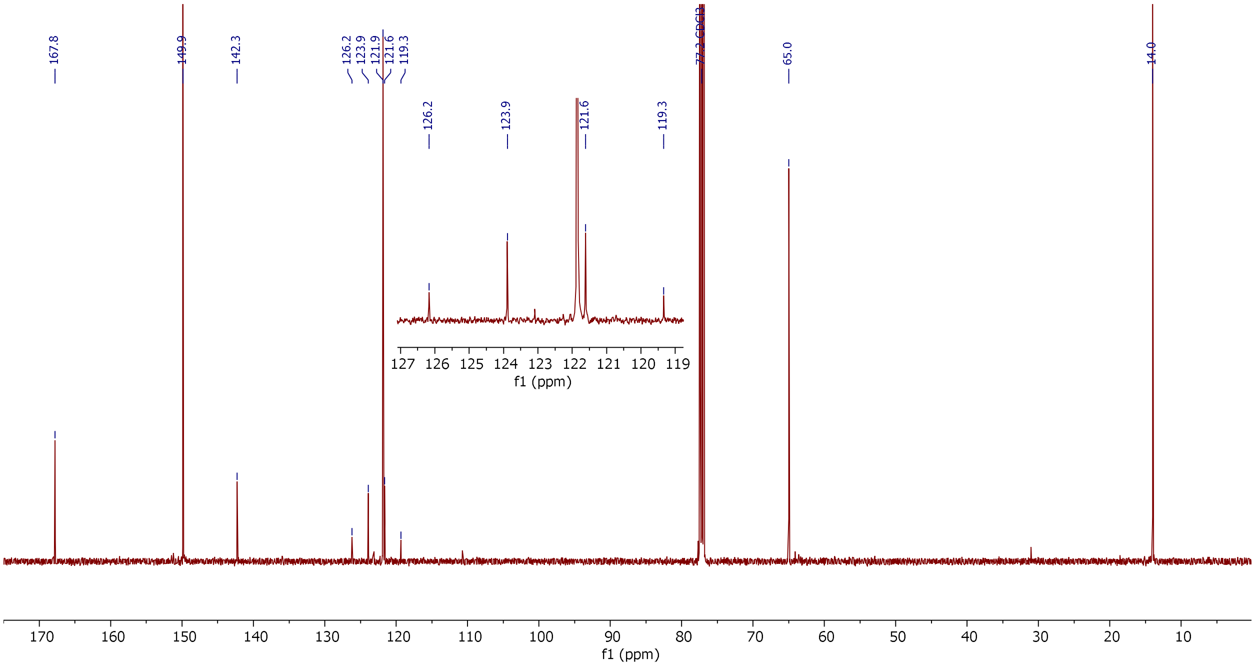

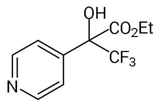

3

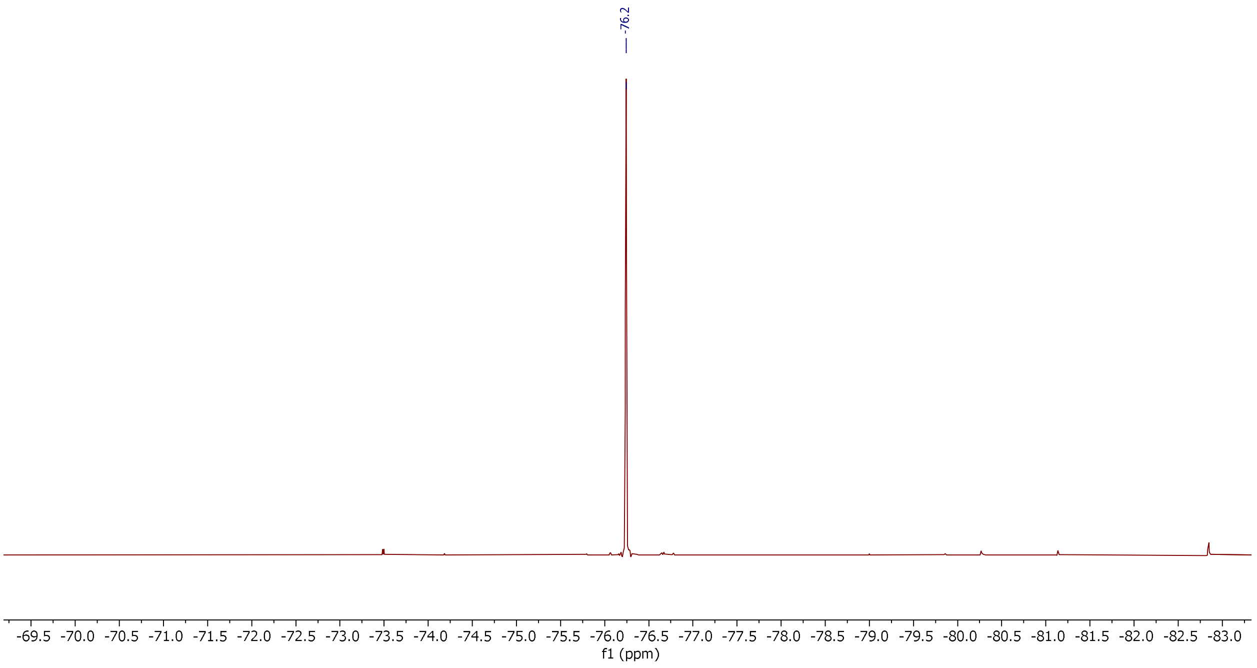
-76.2
-69.5 -70.0 -70.5 -71.0 -71.5 -72.0 -72.5 -73.0 -73.5 -74.0 -74.5 -75.0 -75.5 -76.0 -76.5 -77.0 -77.5 -78.0 -78.5 -79.0 -79.5 -80.0 -80.5 -81.0 -81.5 -82.0 -82.5 -83.0
f1 (ppm)

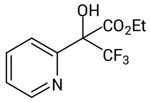

4

ethyl 3,3,3-trifluoro-2-hydroxy-2-(pyridin-2-yl)propanoate (4)

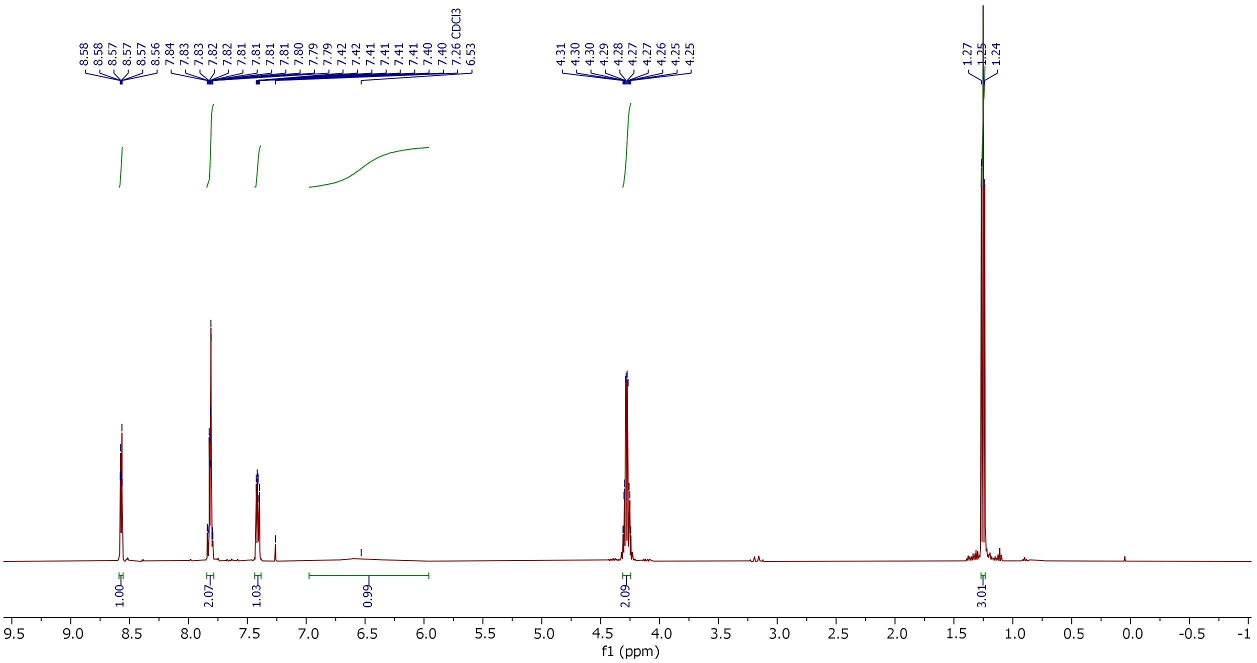

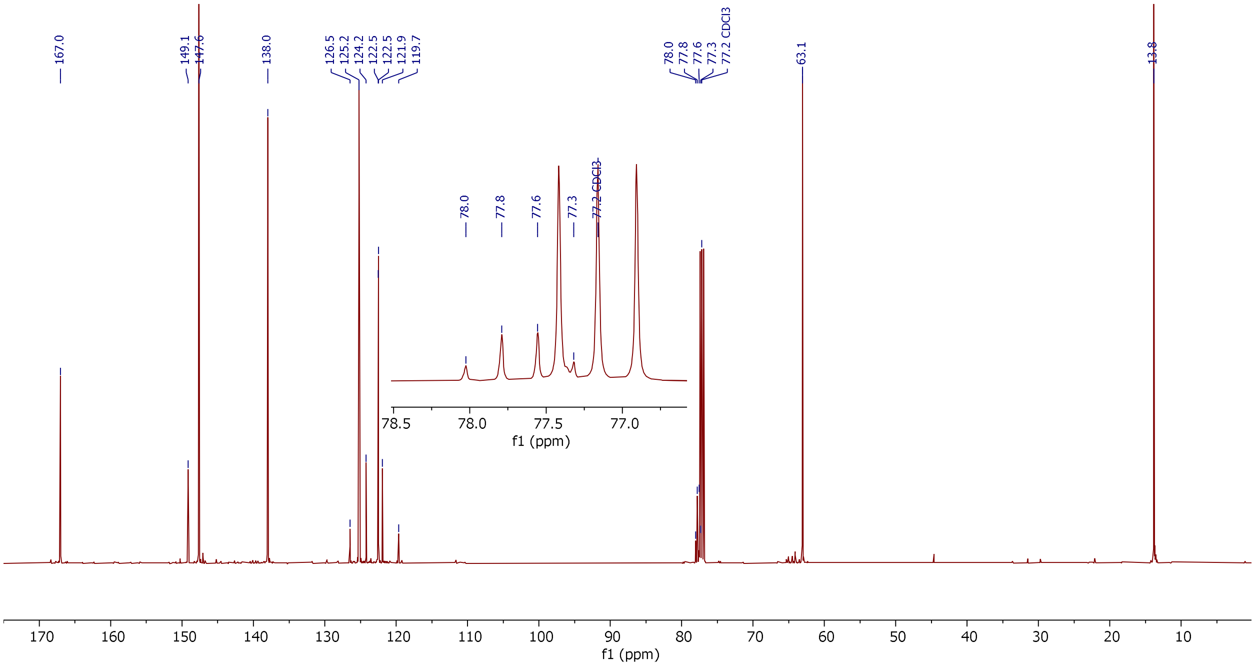

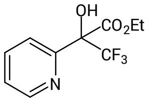
OH
CO2Et
CF3
N

4

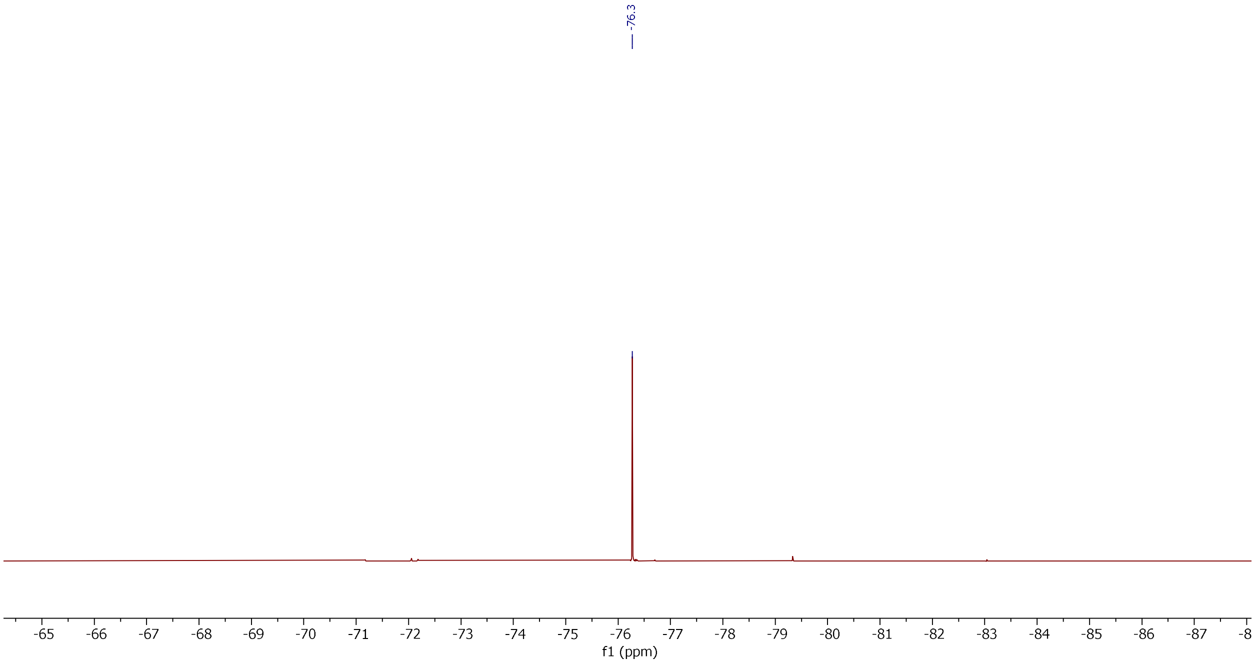
-76.3
-65 -66 -67 -68 -69 -70 -71 -72 -73 -74 -75 -76 -77 -78 -79 -80 -81 -82 -83 -84 -85 -86 -87 -8
f1 (ppm)

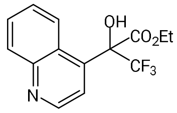

5

ethyl 3,3,3-trifluoro-2-hydroxy-2-(quinolin-4-yl)propanoate (5)

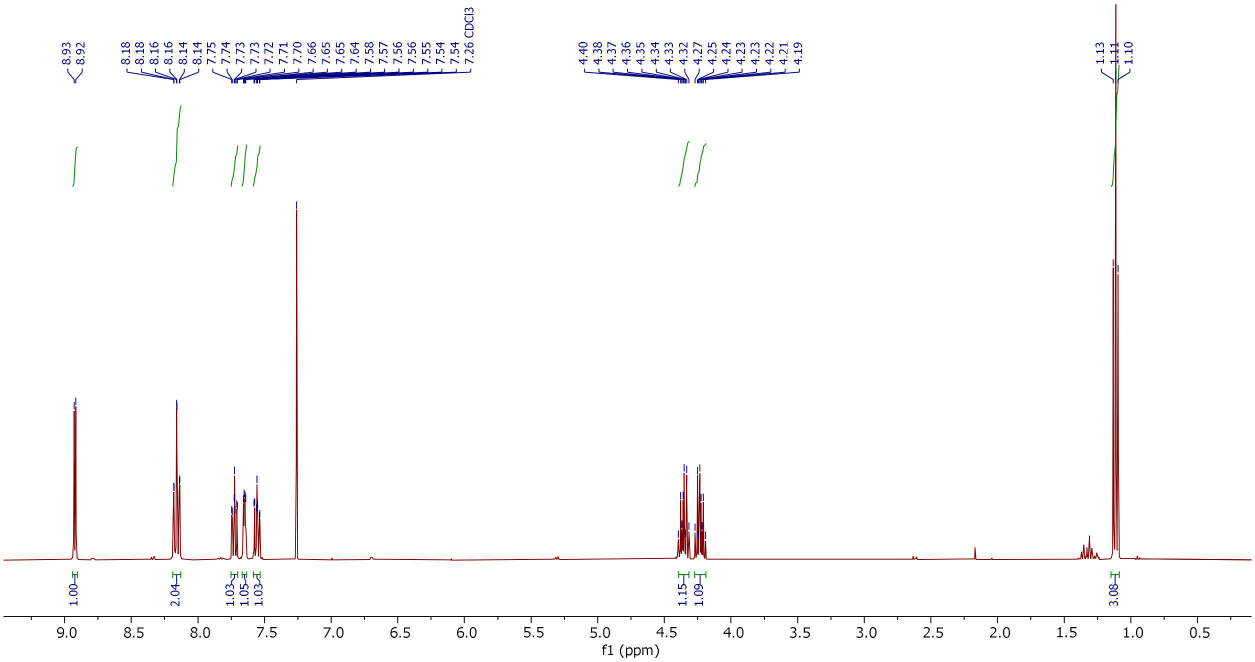

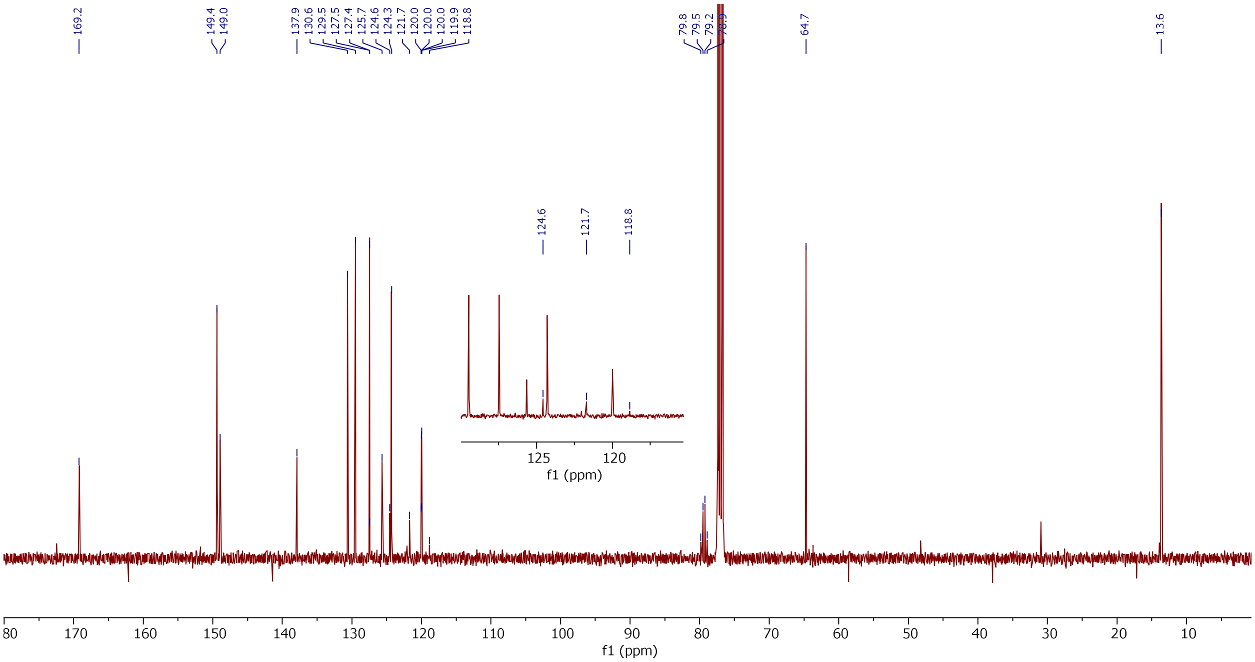

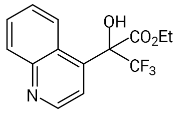
OH
CO2Et
CF3
N

5

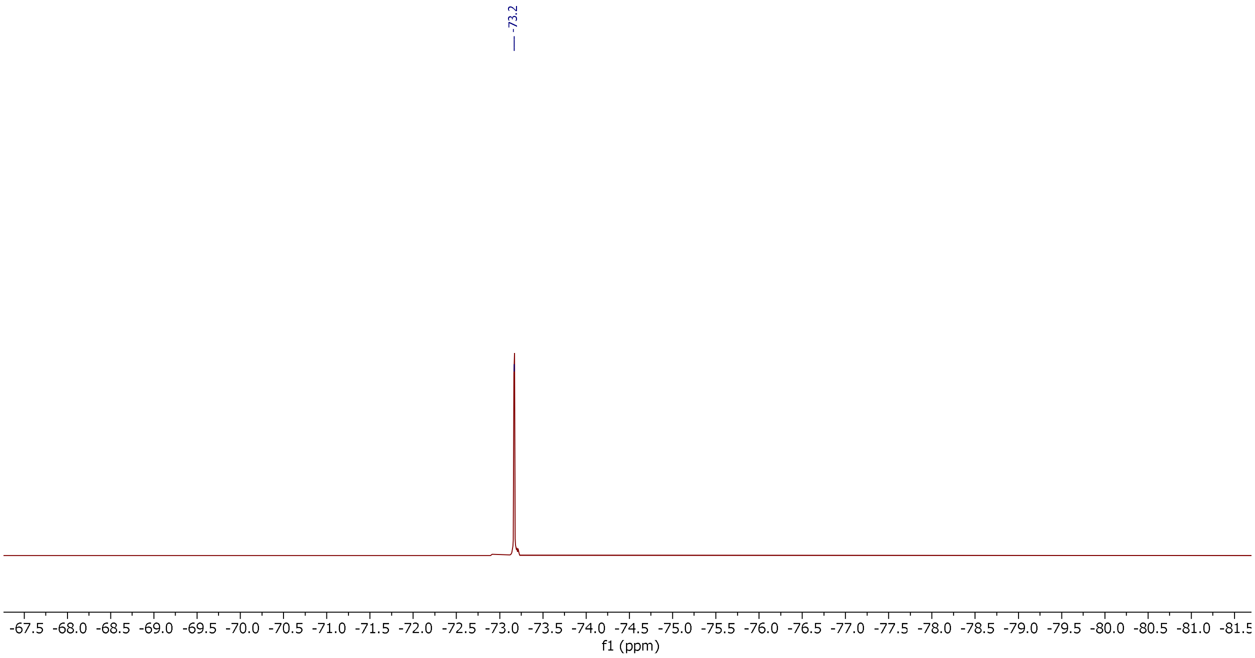
-73.2
-67.5 -68.0 -68.5 -69.0 -69.5 -70.0 -70.5 -71.0 -71.5 -72.0 -72.5 -73.0 -73.5 -74.0 -74.5 -75.0 -75.5 -76.0 -76.5 -77.0 -77.5 -78.0 -78.5 -79.0 -79.5 -80.0 -80.5 -81.0 -81.5
f1 (ppm)

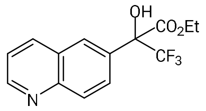

6

ethyl 3,3,3-trifluoro-2-hydroxy-2-(quinolin-6-yl)propanoate (6)

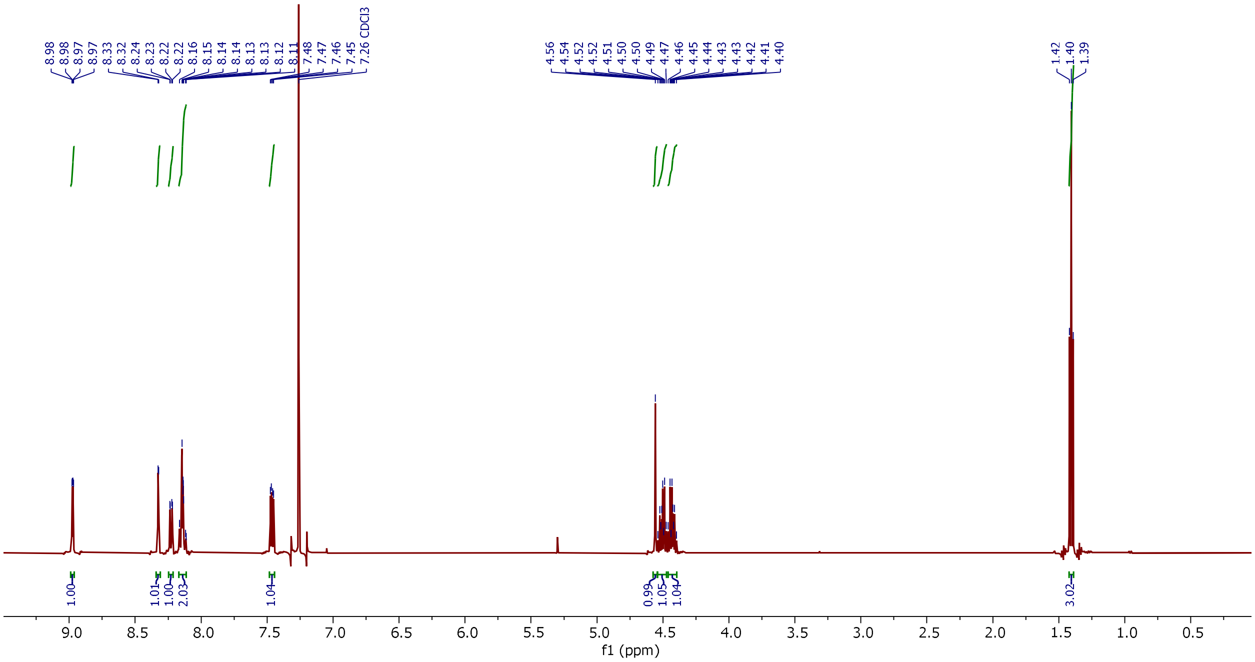

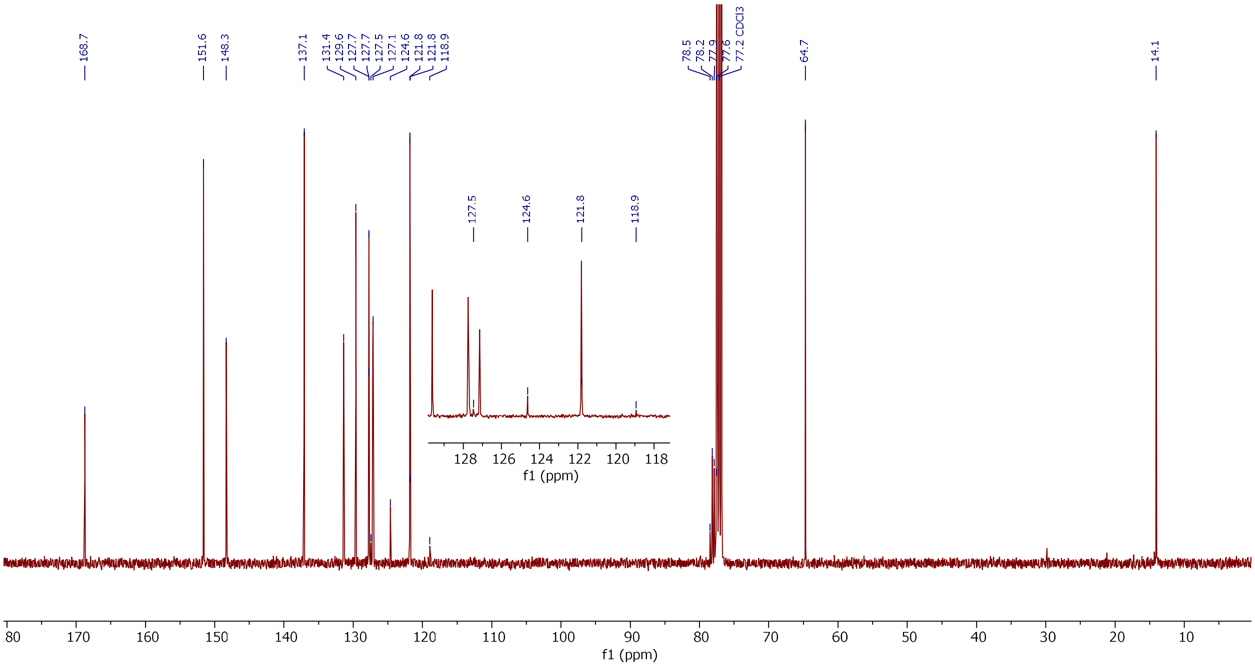

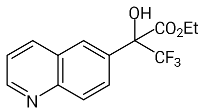

6

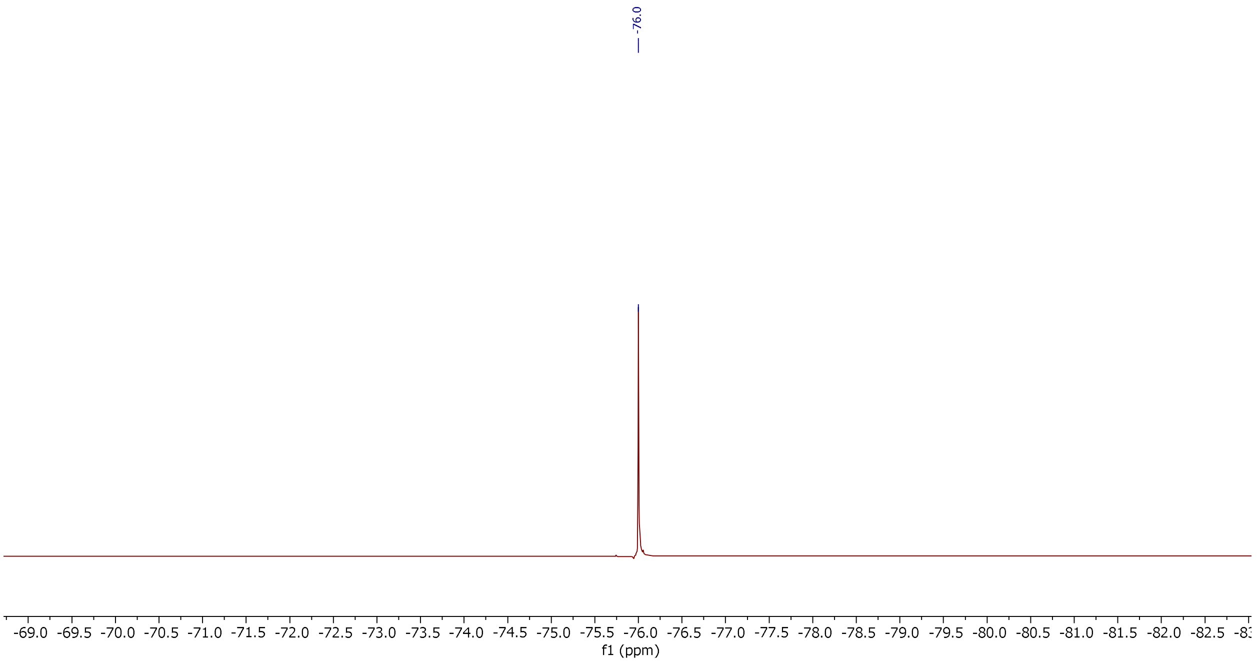
-76.0
-69.0 -69.5 -70.0 -70.5 -71.0 -71.5 -72.0 -72.5 -73.0 -73.5 -74.0 -74.5 -75.0 -75.5 -76.0 -76.5 -77.0 -77.5 -78.0 -78.5 -79.0 -79.5 -80.0 -80.5 -81.0 -81.5 -82.0 -82.5
f1 (ppm)

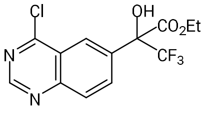

7

ethyl 2-(4-chloroquinazolin-6-yl)-3,3,3-trifluoro-2-hydroxypropanoate (7)

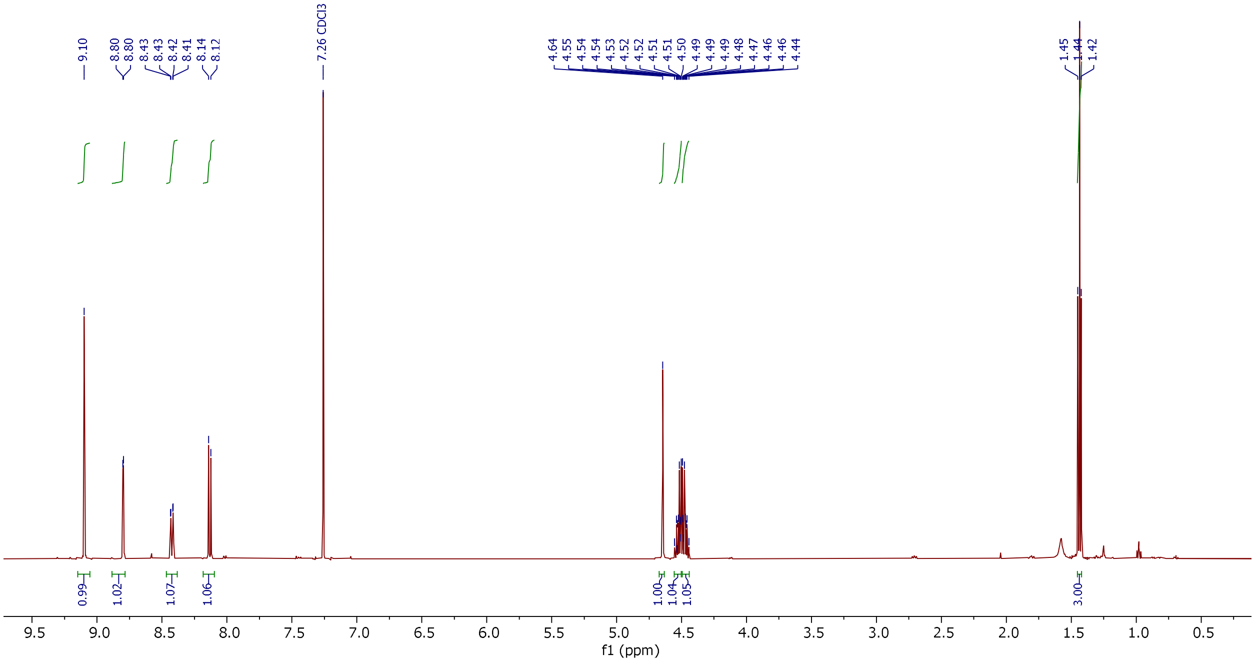

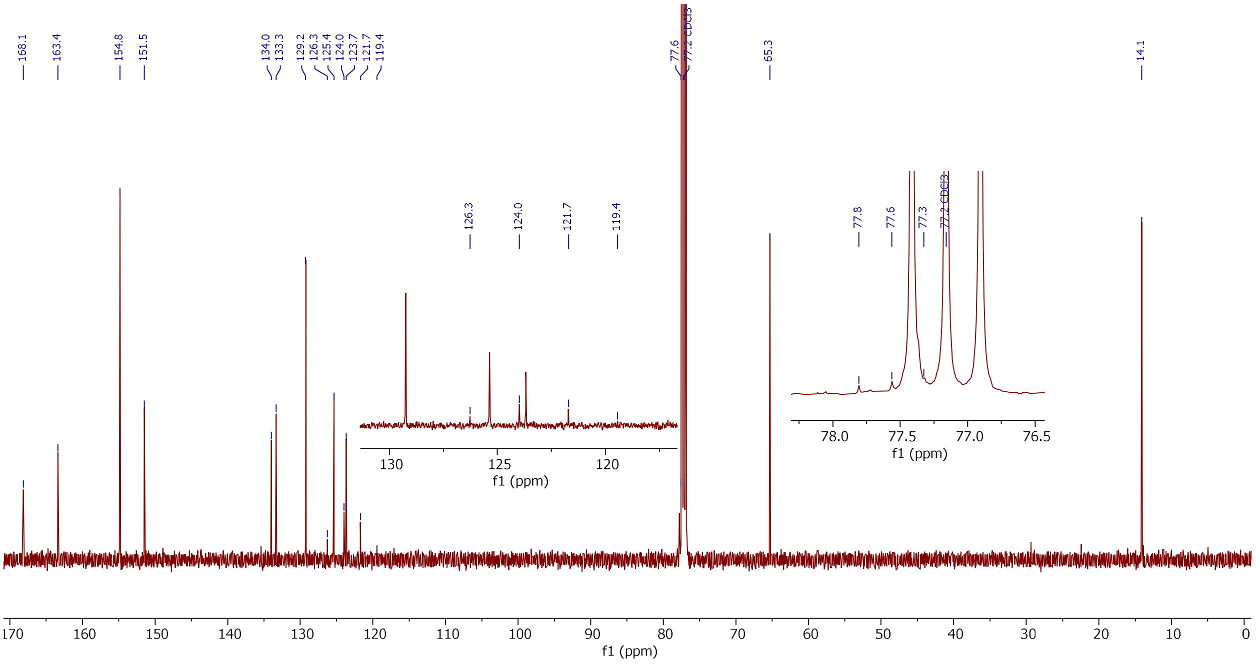

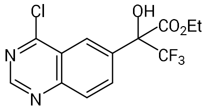
Cl
OH
CO2Et
CF3
N
N

7

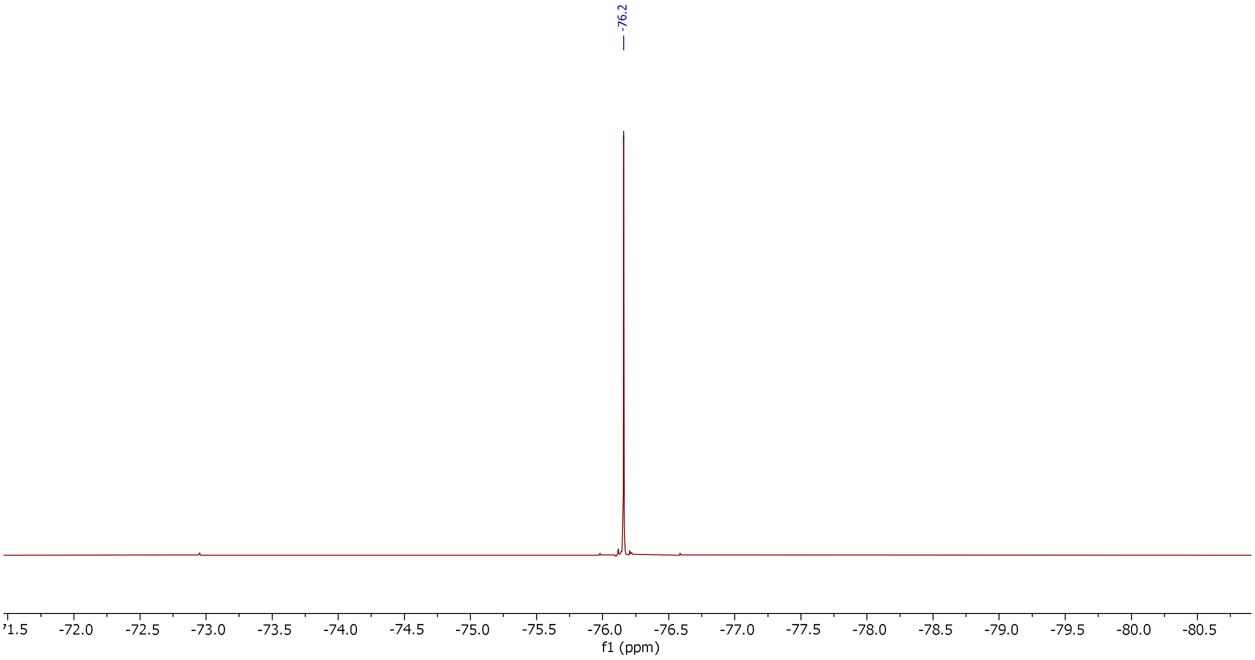
-76.2
-72.0 -72.5 -73.0 -73.5 -74.0 -74.5 -75.0 -75.5 -76.0 -76.5 -77.0 -77.5 -78.0 -78.5 -79.0 -79.5 -80.0 -80.5
f1 (ppm)

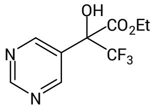

8

ethyl 3,3,3-trifluoro-2-hydroxy-2-(pyrimidin-5-yl)propanoate (8)

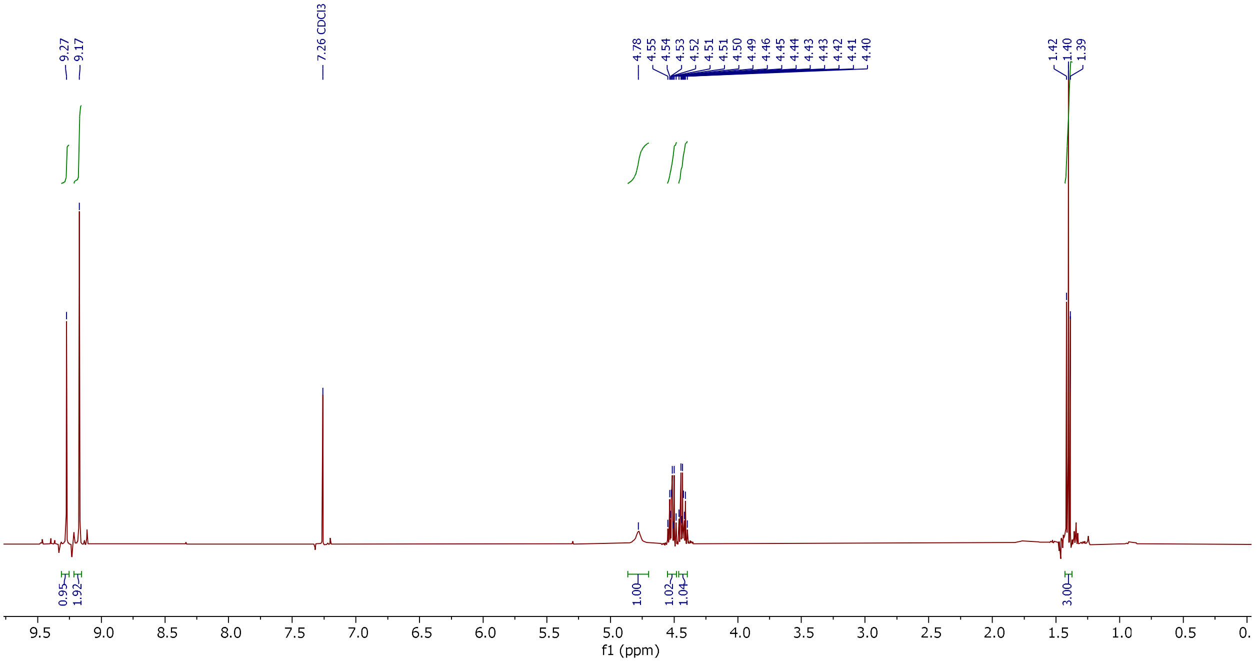

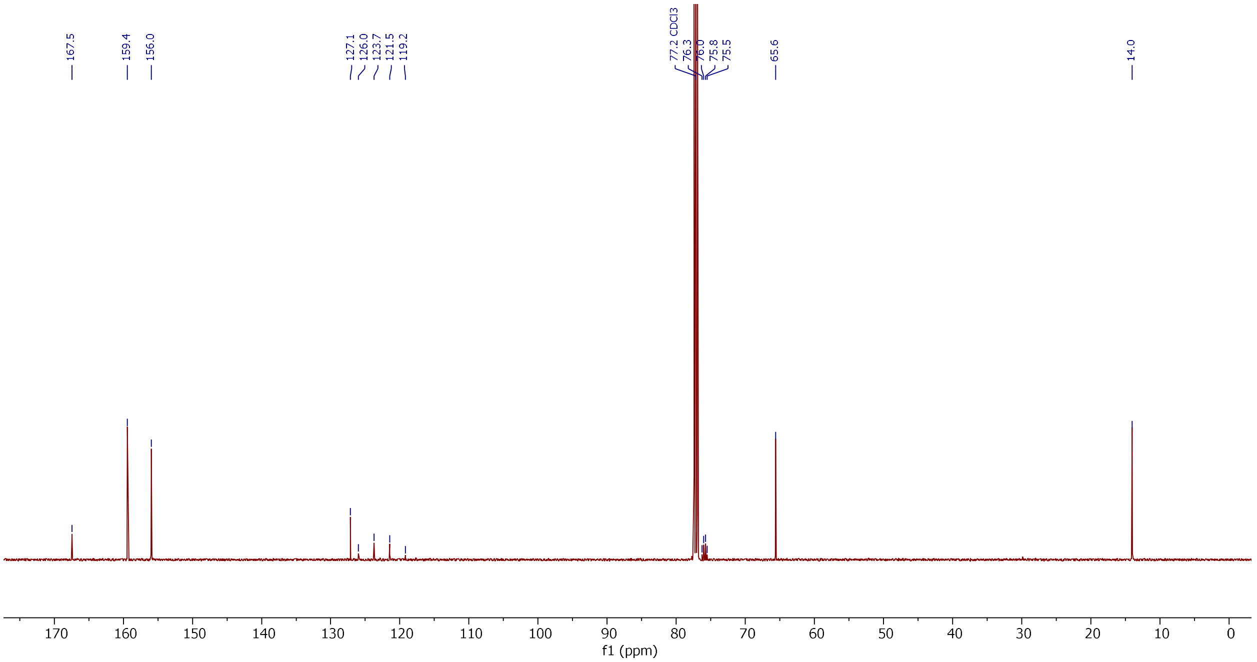

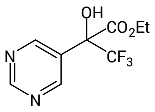

8

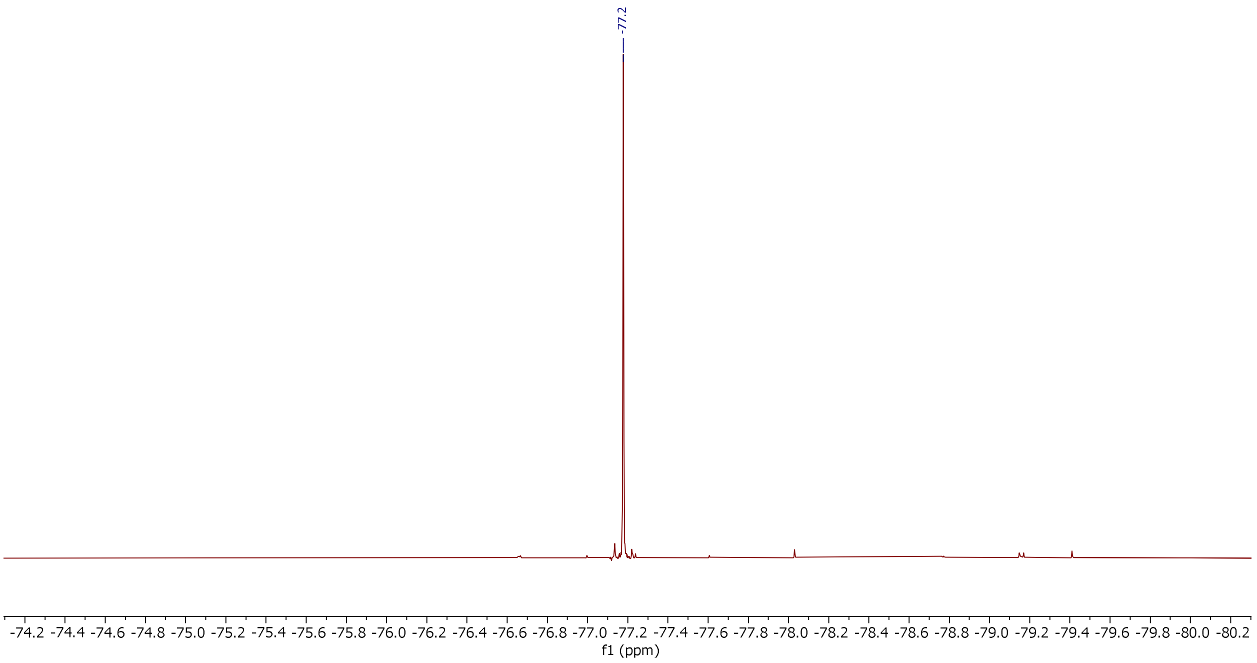

-77.2
-74.2 -74.4 -74.6 -74.8 -75.0 -75.2 -75.4 -75.6 -75.8 -76.0 -76.2 -76.4 -76.6 -76.8 -77.0 -77.2 -77.4 -77.6 -77.8 -78.0 -78.2 -78.4 -78.6 -78.8 -79.0 -79.2 -79.4 -79.6 -79.8 -80.0 -80.2
f1 (ppm)

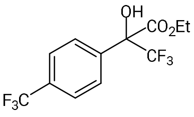

9

ethyl 3,3,3-trifluoro-2-hydroxy-2-(4-(trifluoromethyl)phenyl)propanoate (9)

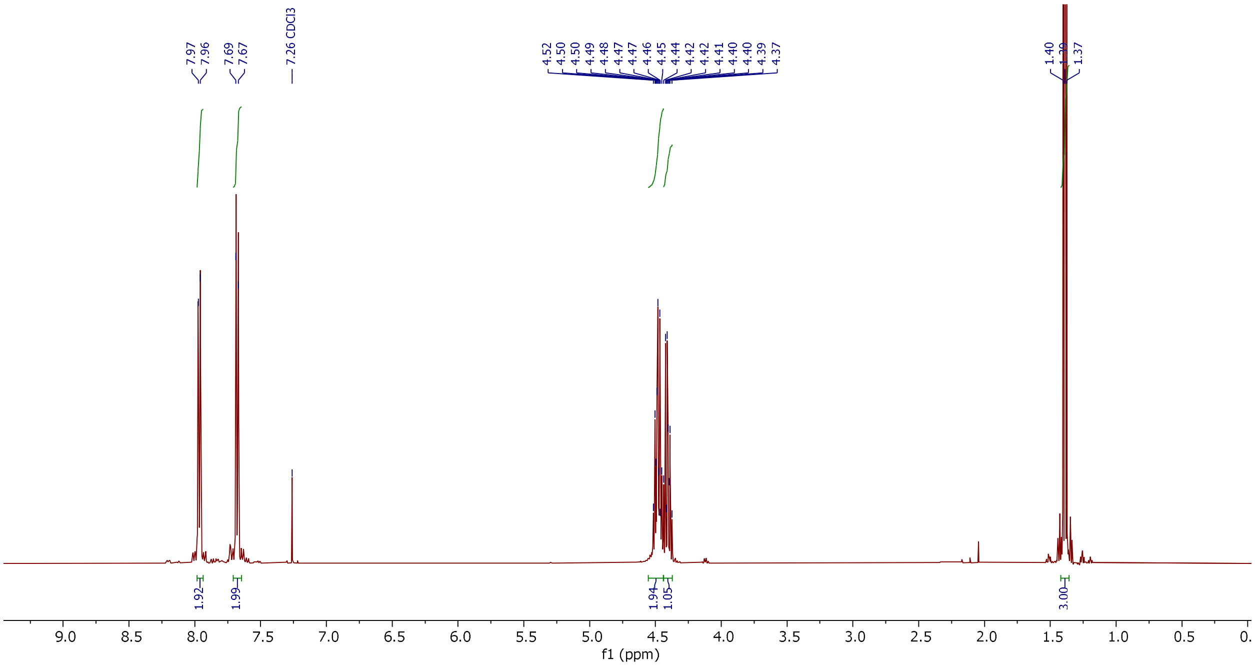

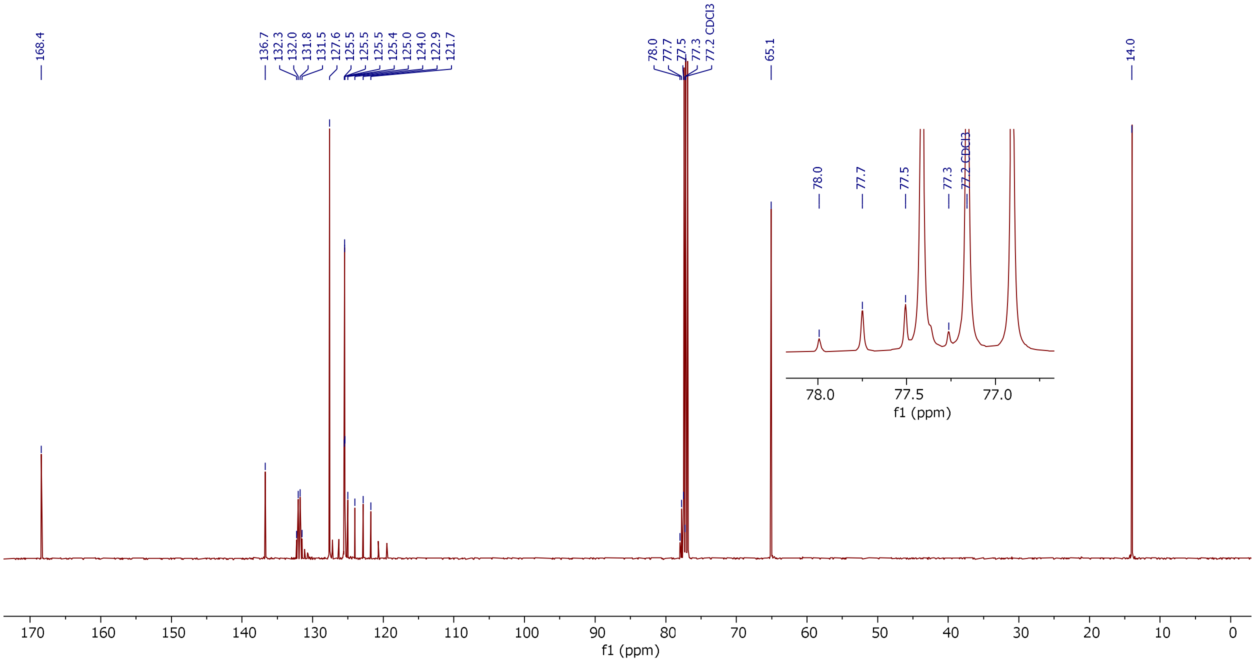

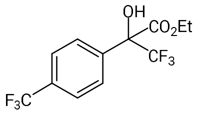

9

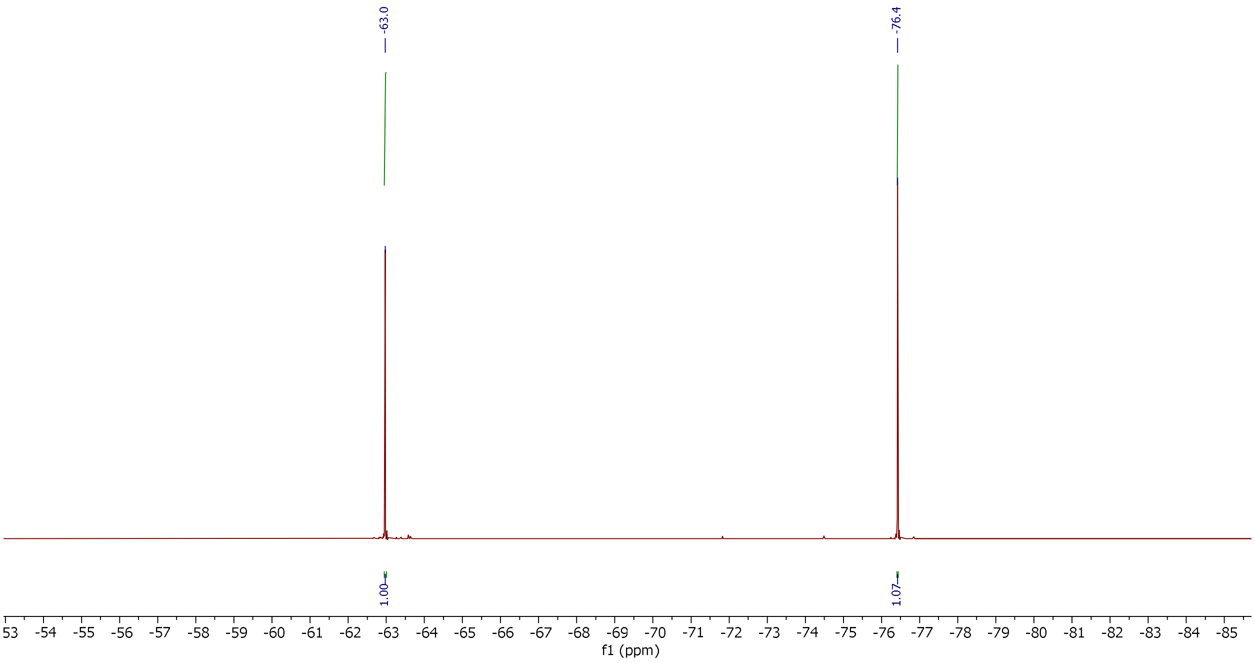

-63.0
-76.4
1.00
1.07
53 -54 -55 -56 -57 -58 -59 -60 -61 -62 -63 -64 -65 -66 -67 -68 -69 -70 -71 -72 -73 -74 -75 -76 -77 -78 -79 -80 -81 -82 -83 -84 -85
f1 (ppm)

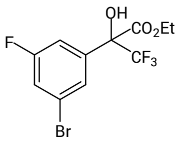

10

ethyl 2-(3-bromo-5-fluorophenyl)-3,3,3-trifluoro-2-hydroxypropanoate (10)

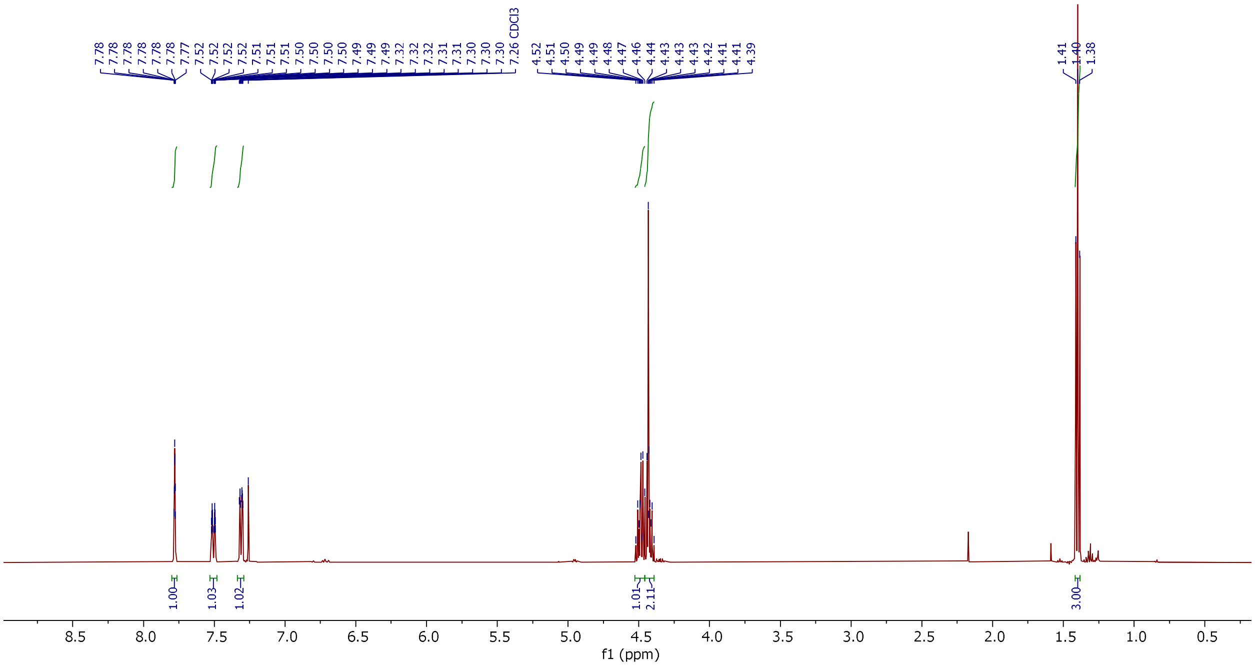

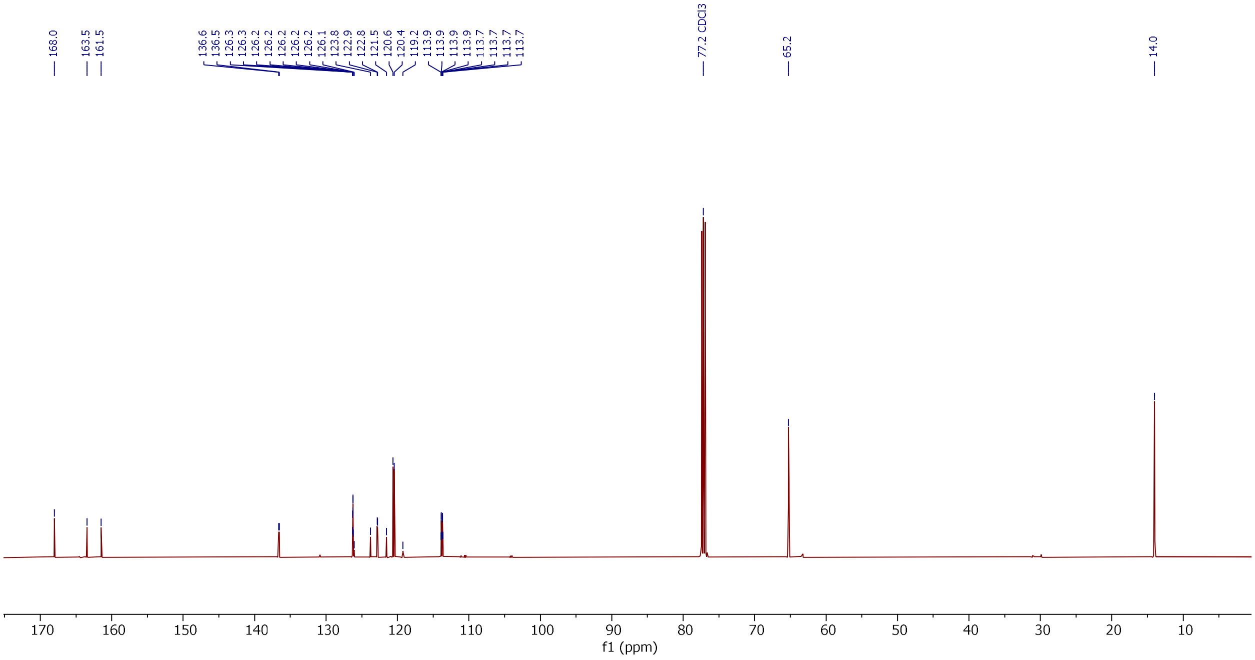

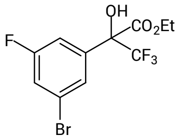

10

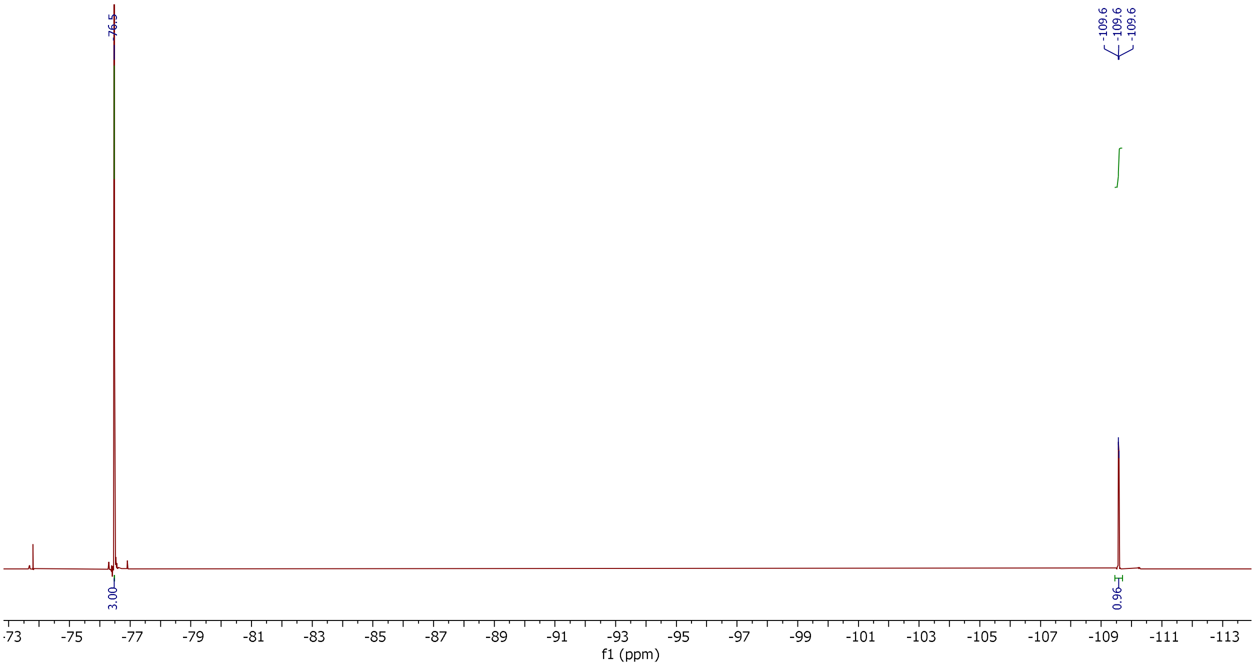

-76.5
-109.6
-109.6
-109.6
3.00
0.96
-73
-75
-77
-79
-81
-83
-85
-87
-89
-91
-93
-95
-97
-99
-101
-103
-105
-107
-109
-111
-113
f1 (ppm)

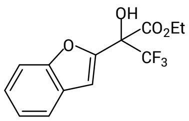

11

ethyl 2-(benzofuran-2-yl)-3,3,3-trifluoro-2-hydroxypropanoate (11)

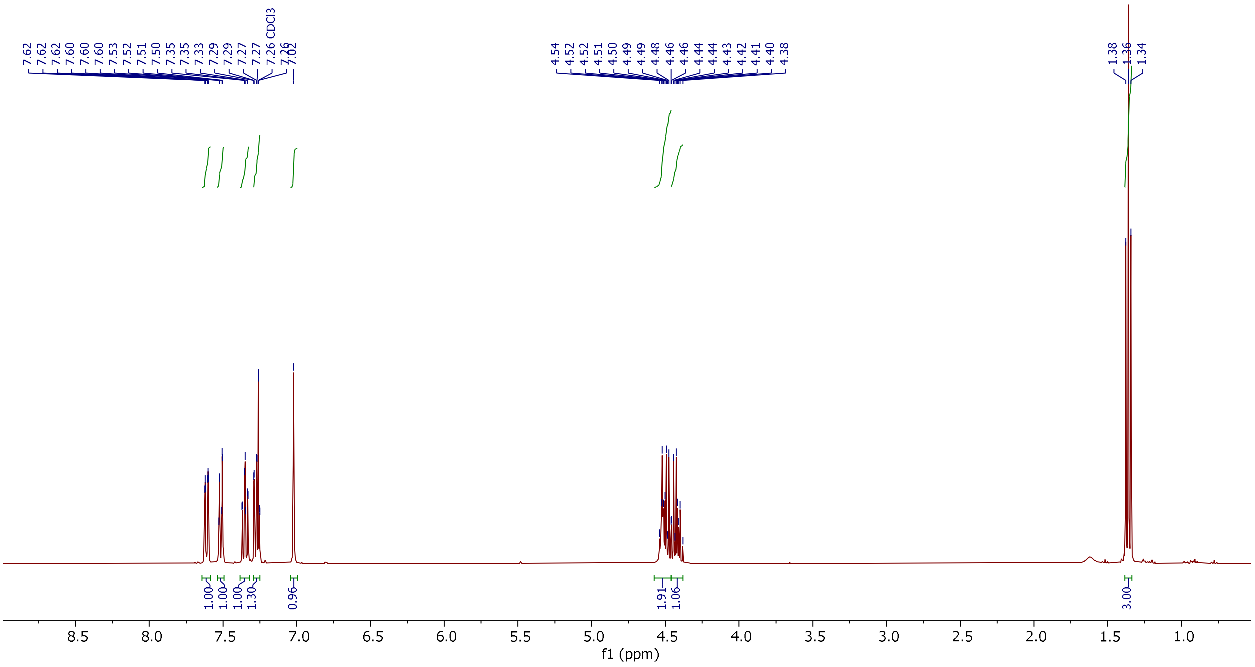

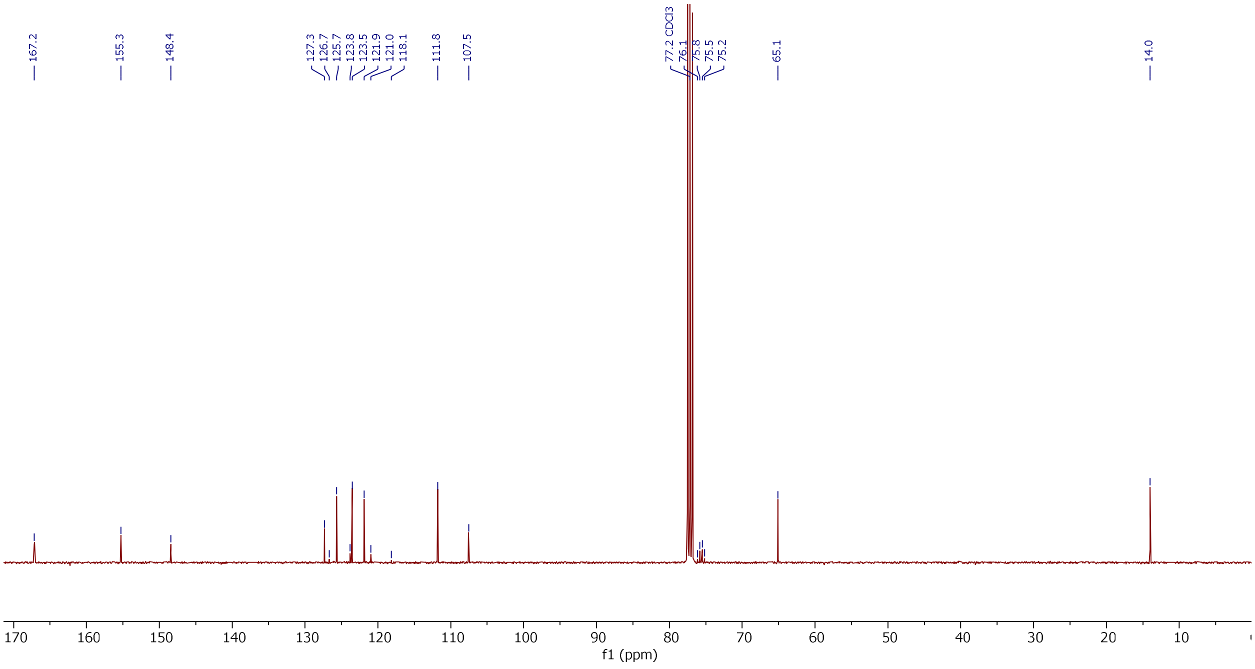

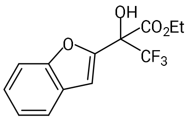
OH
CO2Et
CF3
O

11

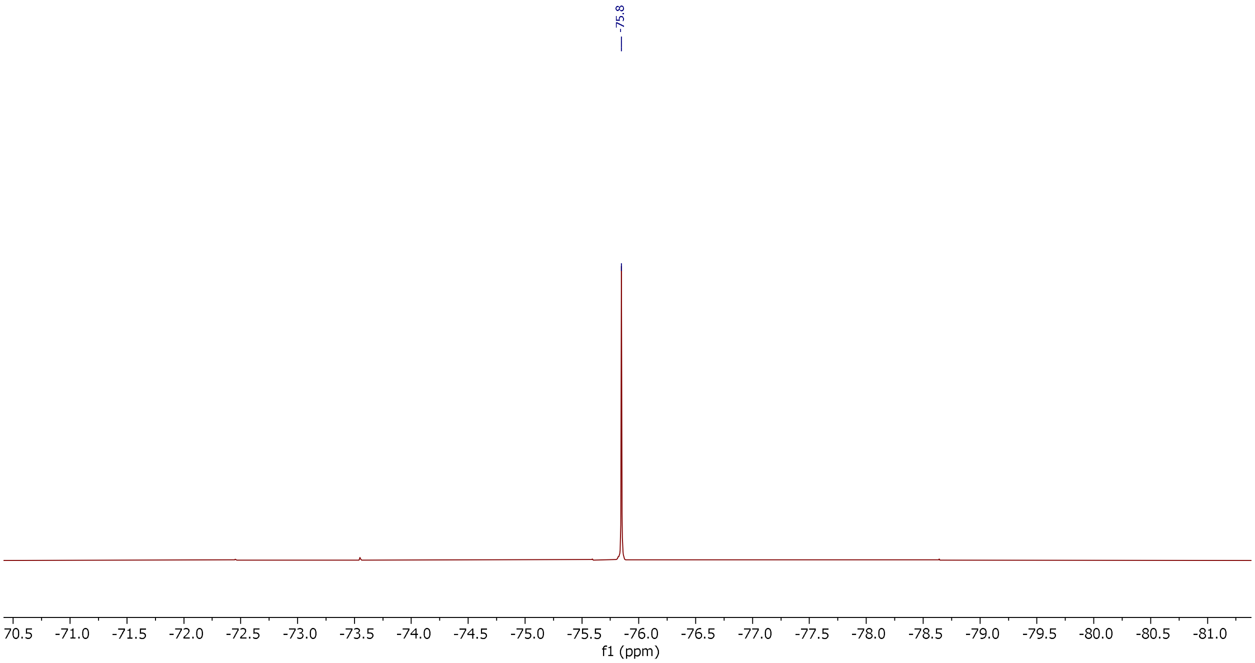
-75.8
70.5 -71.0 -71.5 -72.0 -72.5 -73.0 -73.5 -74.0 -74.5 -75.0 -75.5 -76.0 -76.5 -77.0 -77.5 -78.0 -78.5 -79.0 -79.5 -80.0 -80.5 -81.0
f1 (ppm)

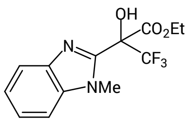

12

ethyl 3,3,3-trifluoro-2-hydroxy-2-(1-methyl-1*H*-benzo[*d*]imidazol-2-yl)propanoate (12)

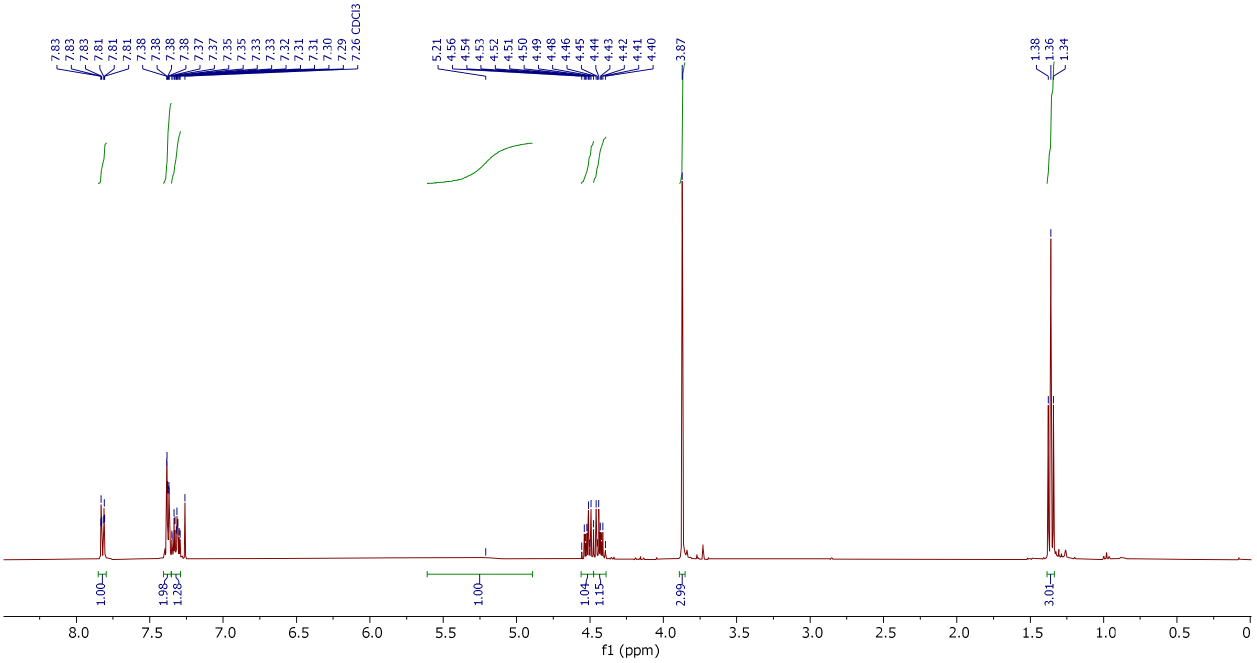

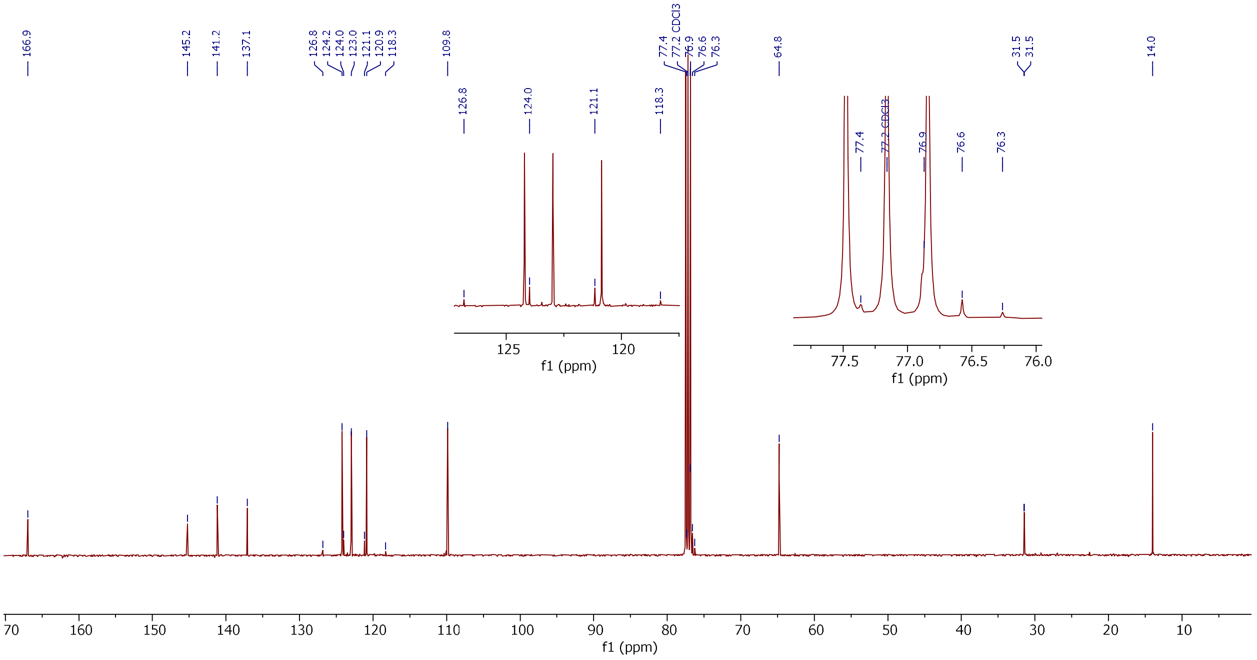

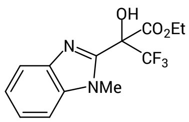
OH
CO2Et
CF3
N
NMe

12

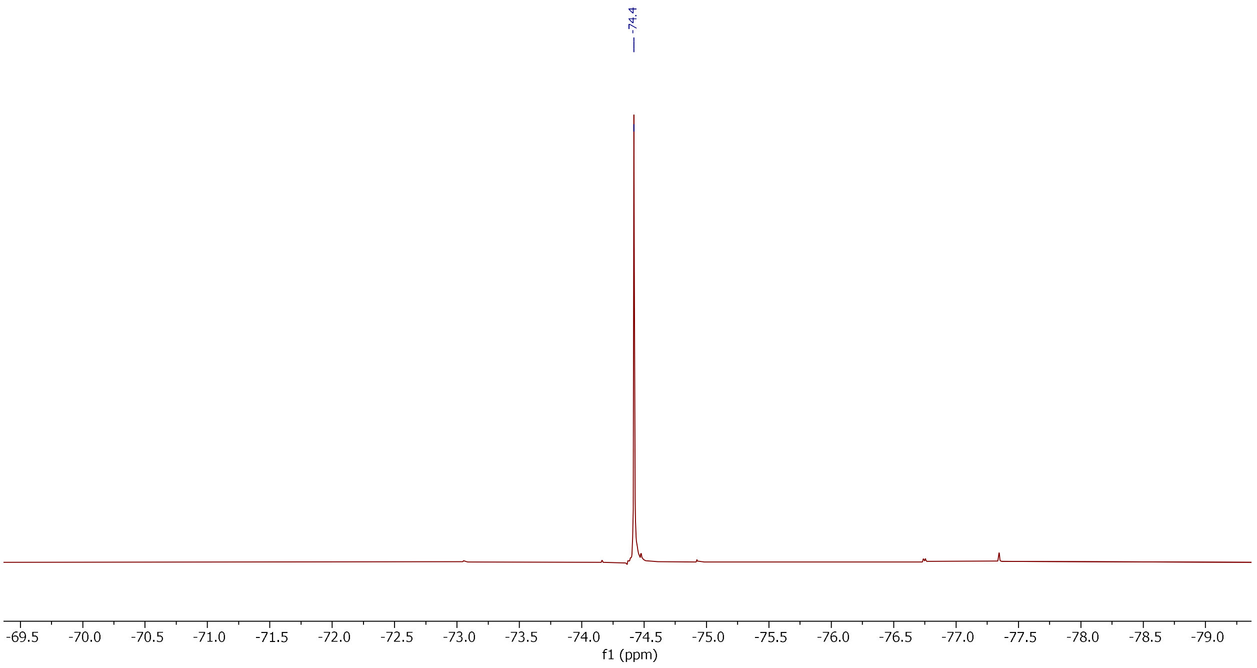
-74.4
-69.5
-70.0
-70.5
-71.0
-71.5
-72.0
-72.5
-73.0
-73.5
-74.0
-74.5
-75.0
-75.5
-76.0
-76.5
-77.0
-77.5
-78.0
-78.5
-79.0
f1 (ppm)

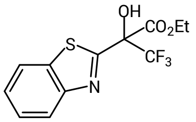

13

ethyl 2-(benzo[*d*]thiazol-2-yl)-3,3,3-trifluoro-2-hydroxypropanoate (13)

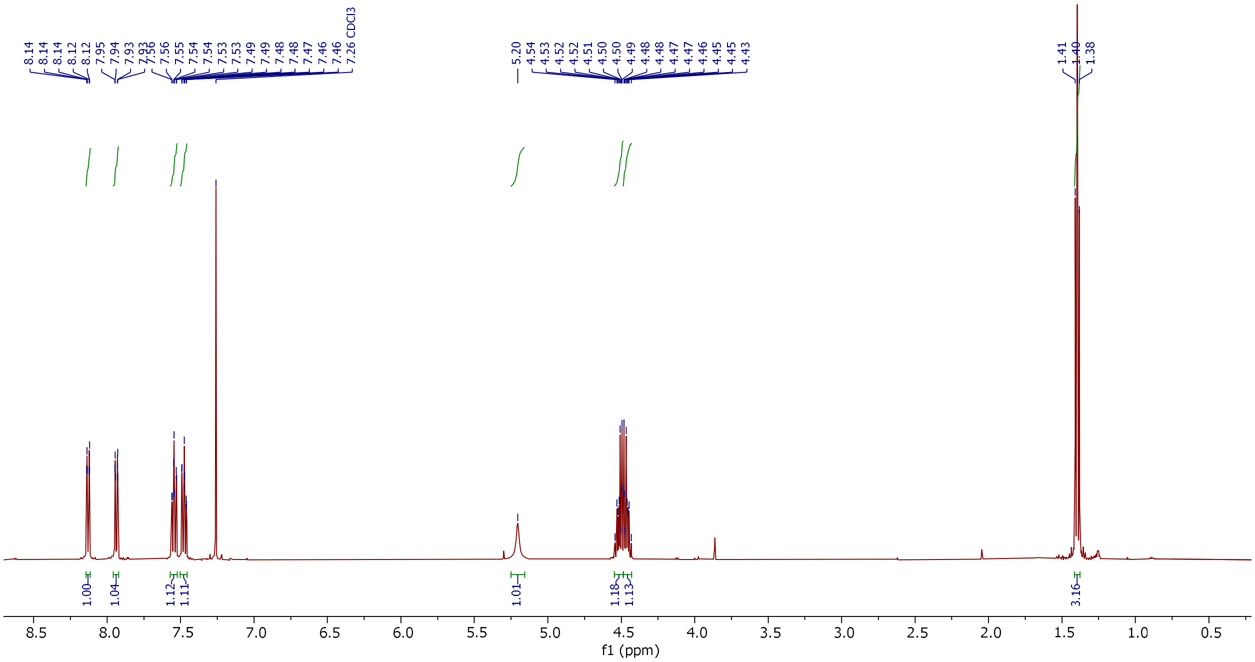

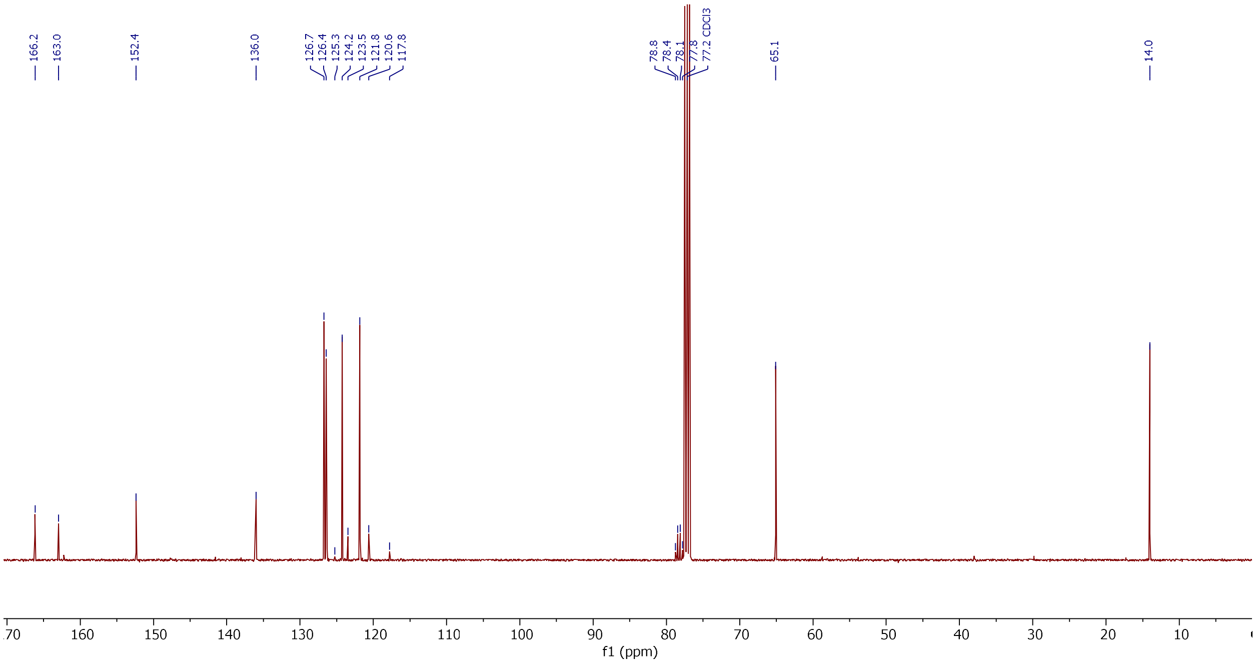

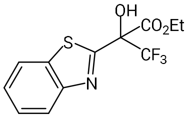

13

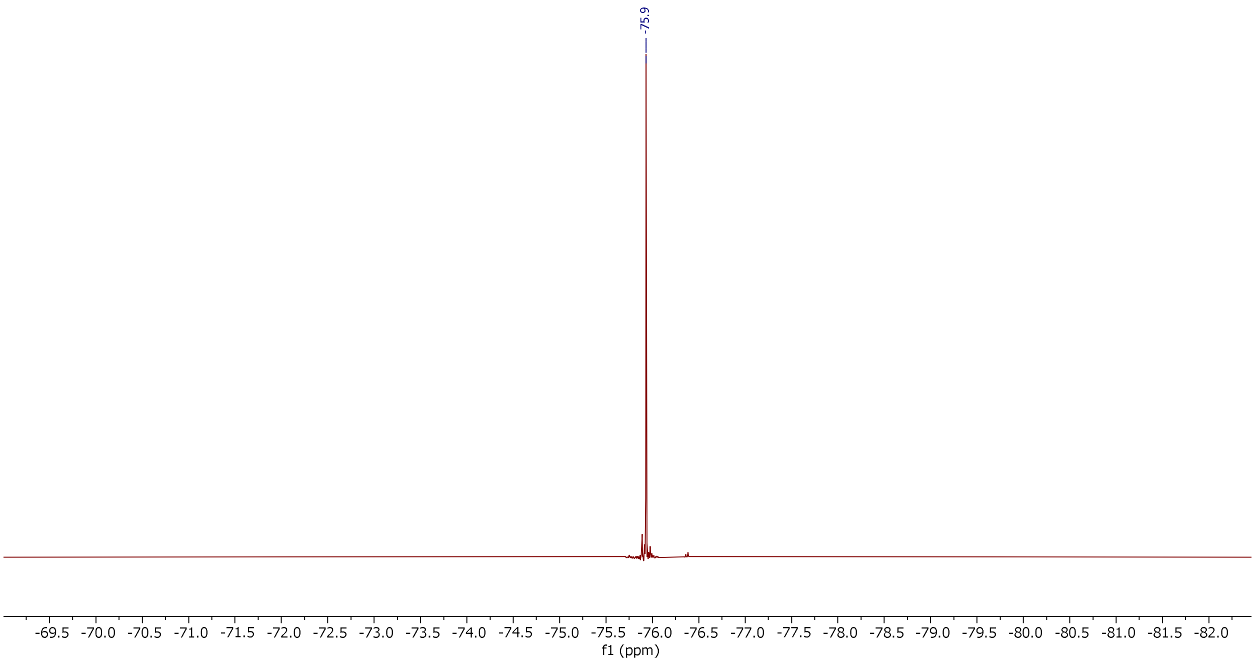
-75.9
-69.5 -70.0 -70.5 -71.0 -71.5 -72.0 -72.5 -73.0 -73.5 -74.0 -74.5 -75.0 -75.5 -76.0 -76.5 -77.0 -77.5 -78.0 -78.5 -79.0 -79.5 -80.0 -80.5 -81.0 -81.5 -82.0
f1 (ppm)

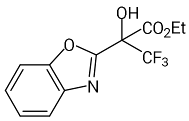

14

ethyl 2-(benzo[*d*]oxazol-2-yl)-3,3,3-trifluoro-2-hydroxypropanoate (14)

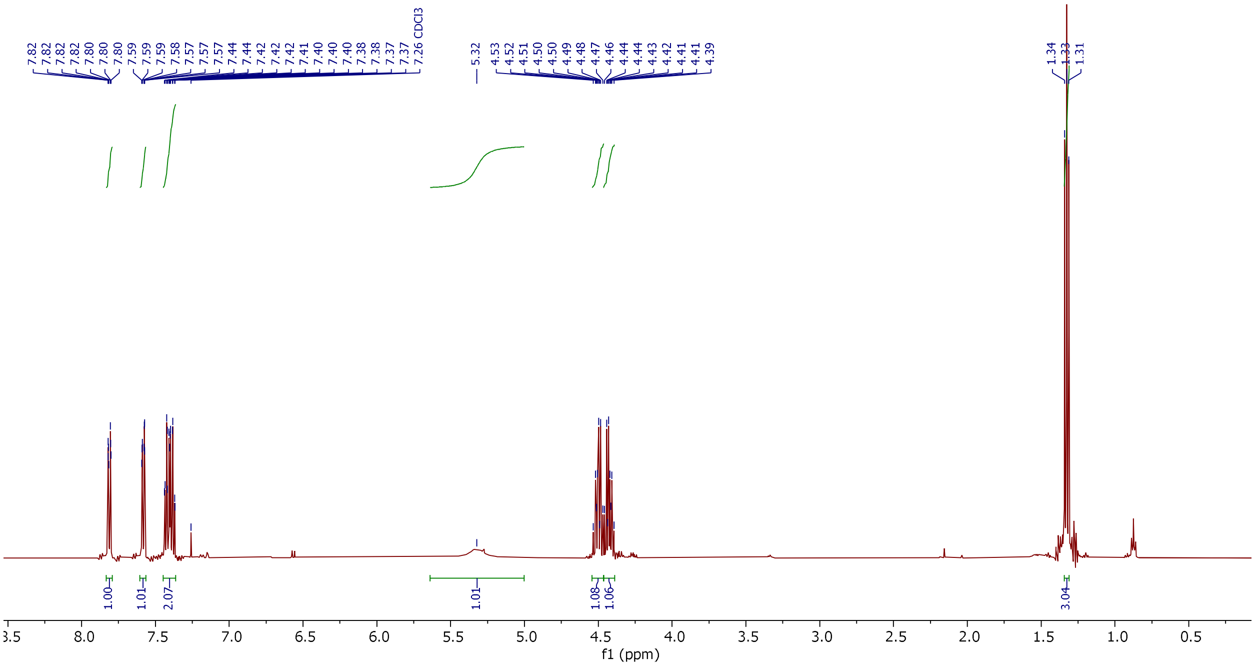

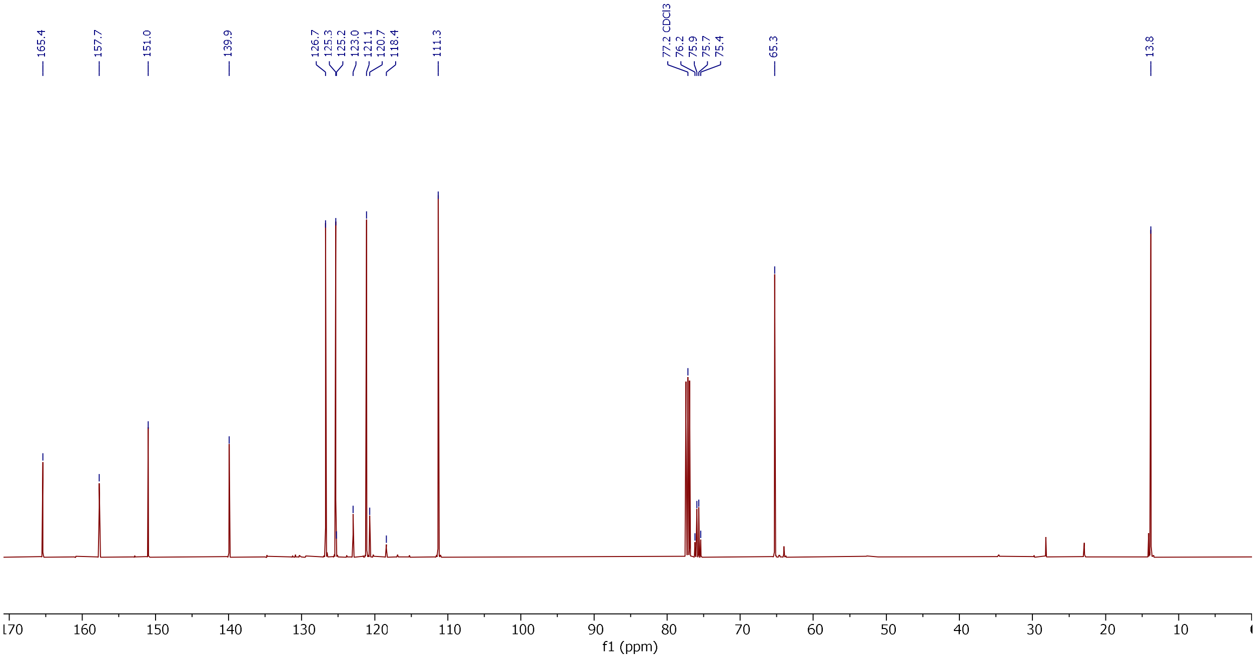

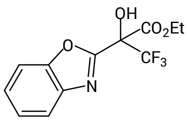
OH
CO2Et
CF3
O
N

14

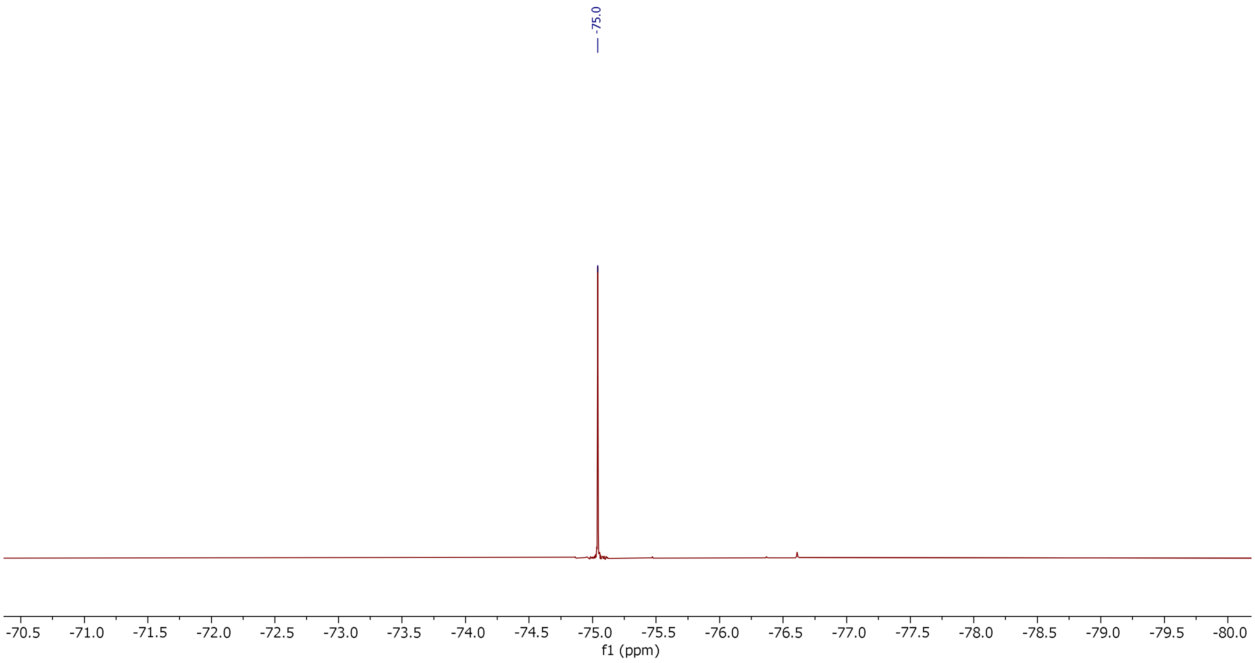
-75.0
-70.5
-71.0
-71.5
-72.0
-72.5
-73.0
-73.5
-74.0
-74.5
-75.0
-75.5
-76.0
-76.5
-77.0
-77.5
-78.0
-78.5
-79.0
-79.5
-80.0
f1 (ppm)

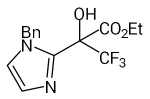

15

ethyl 2-(1-benzyl-1*H*-imidazol-2-yl)-3,3,3-trifluoro-2-hydroxypropanoate (15)

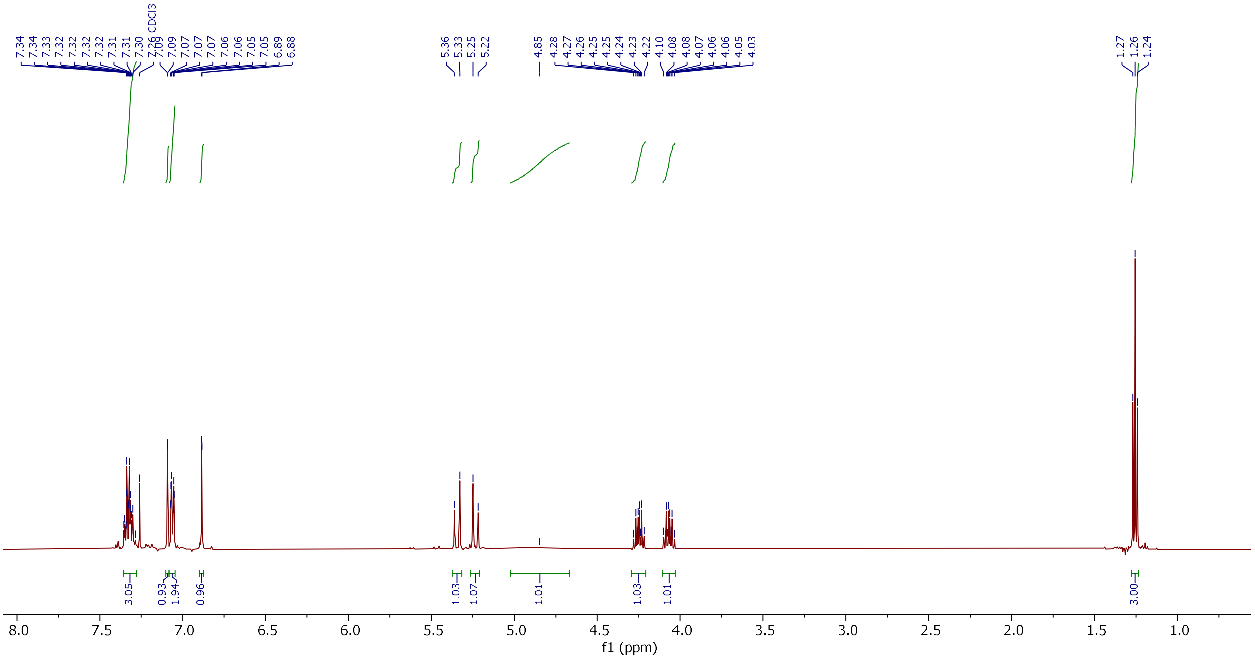

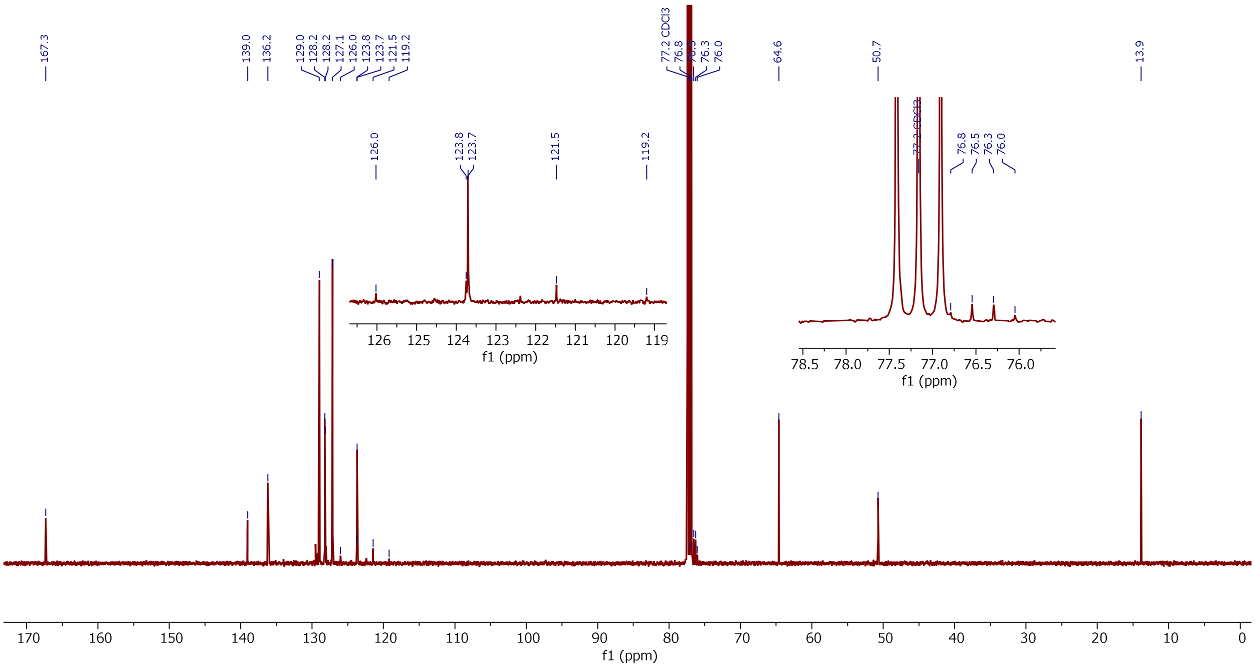

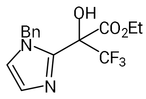

OH
Bn
N
CO2Et
CF3
N

15

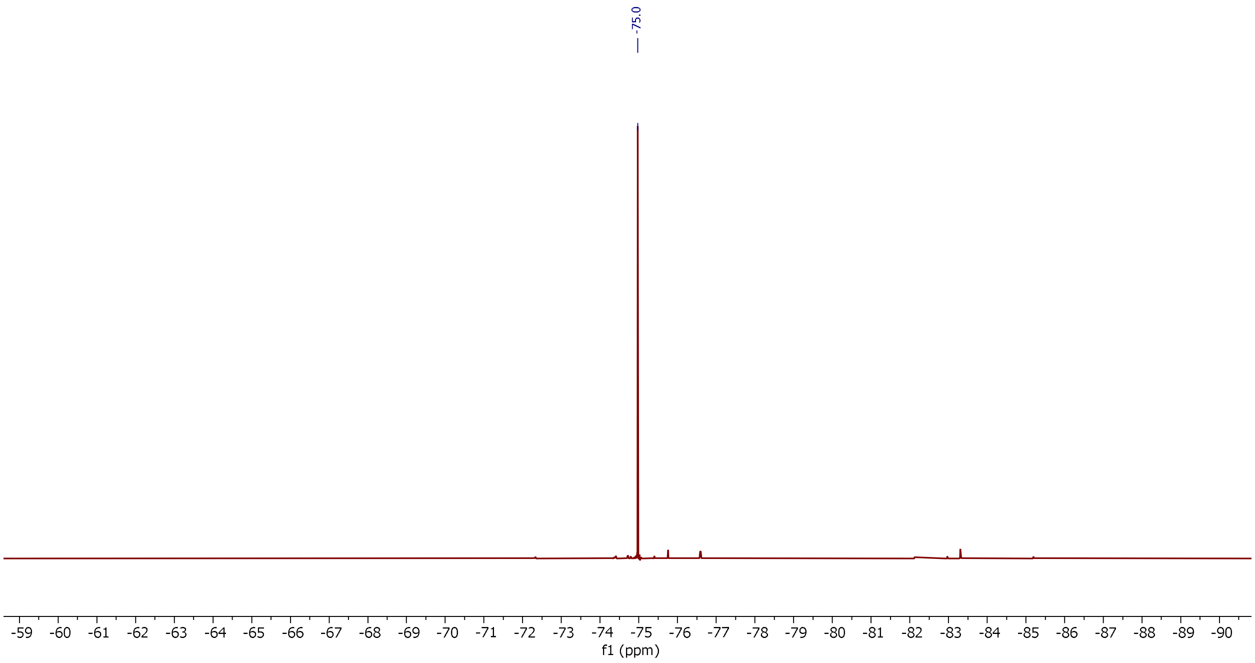

-75.0
-59
-60
-61
-62
-63
-64
-65
-66
-67
-68
-69
-70
-71
-72
-73
-74
-75
-76
-77
-78
-79
-80
-81
-82
-83
-84
-85
-86
-87
-88
-89
-90
f1 (ppm)

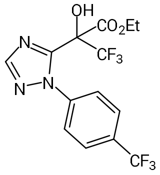

16

ethyl 3,3,3-trifluoro-2-hydroxy-2-(4-(4-(trifluoromethyl)phenyl)-4*H*-1,2,4-triazol-3-yl)propanoate (16)

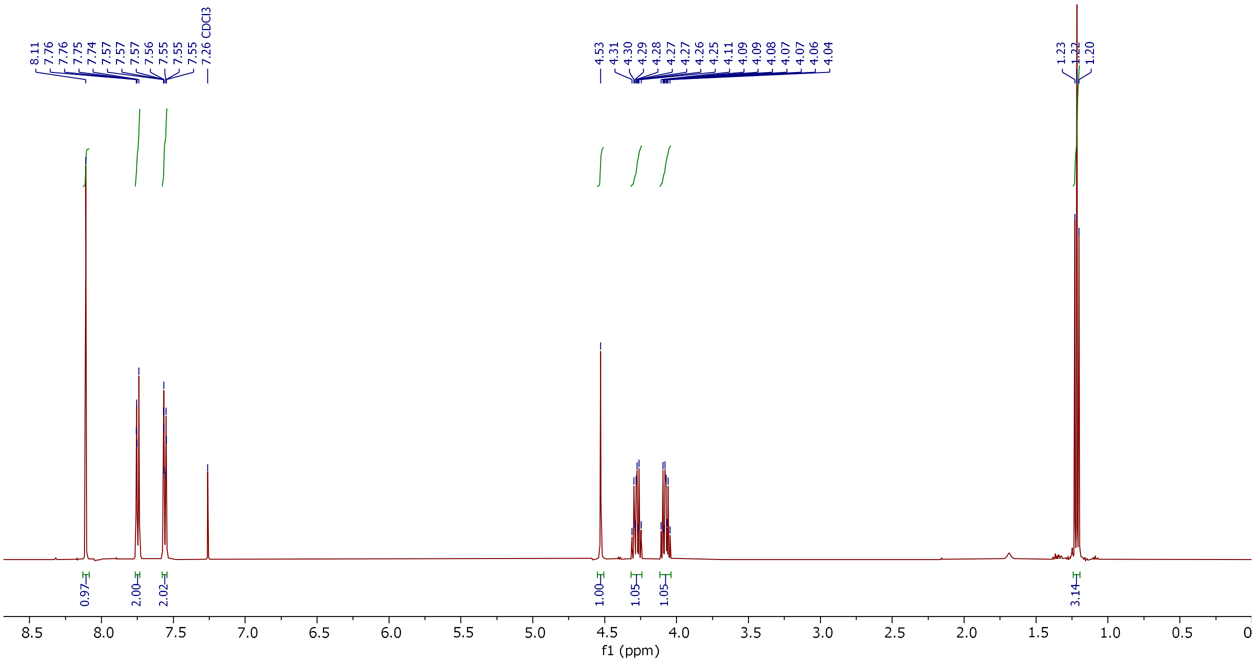

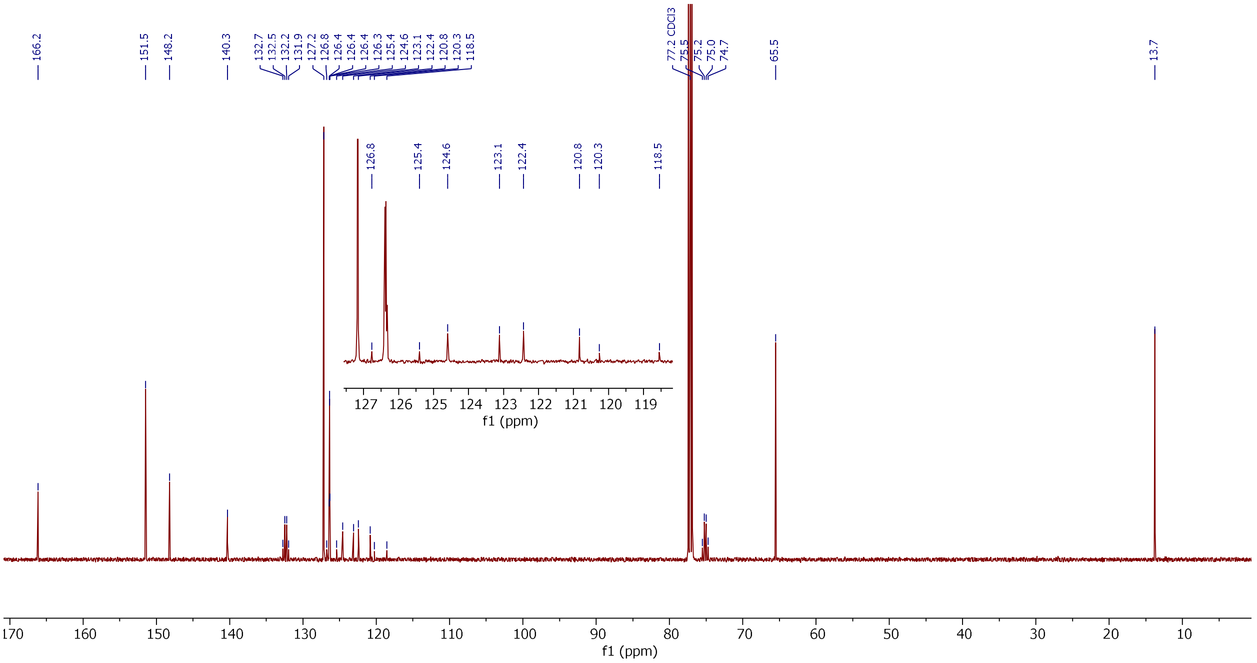

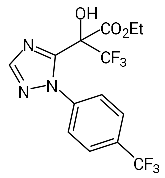

16

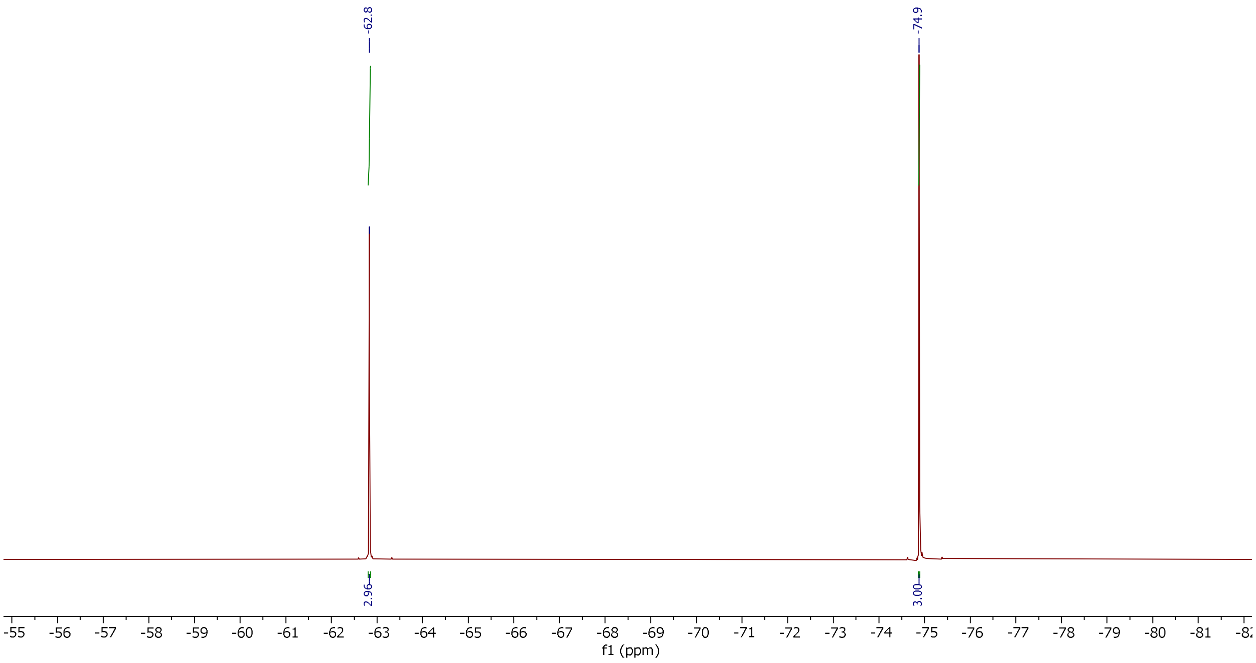
-62.8
-74.9
2.96
3.00
-55 -56 -57 -58 -59 -60 -61 -62 -63 -64 -65 -66 -67 -68 -69 -70 -71 -72 -73 -74 -75 -76 -77 -78 -79 -80 -81
f1 (ppm)

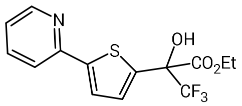

17

ethyl 3,3,3-trifluoro-2-hydroxy-2-(5-(pyridin-2-yl)thiophen-2-yl)propanoate (17)

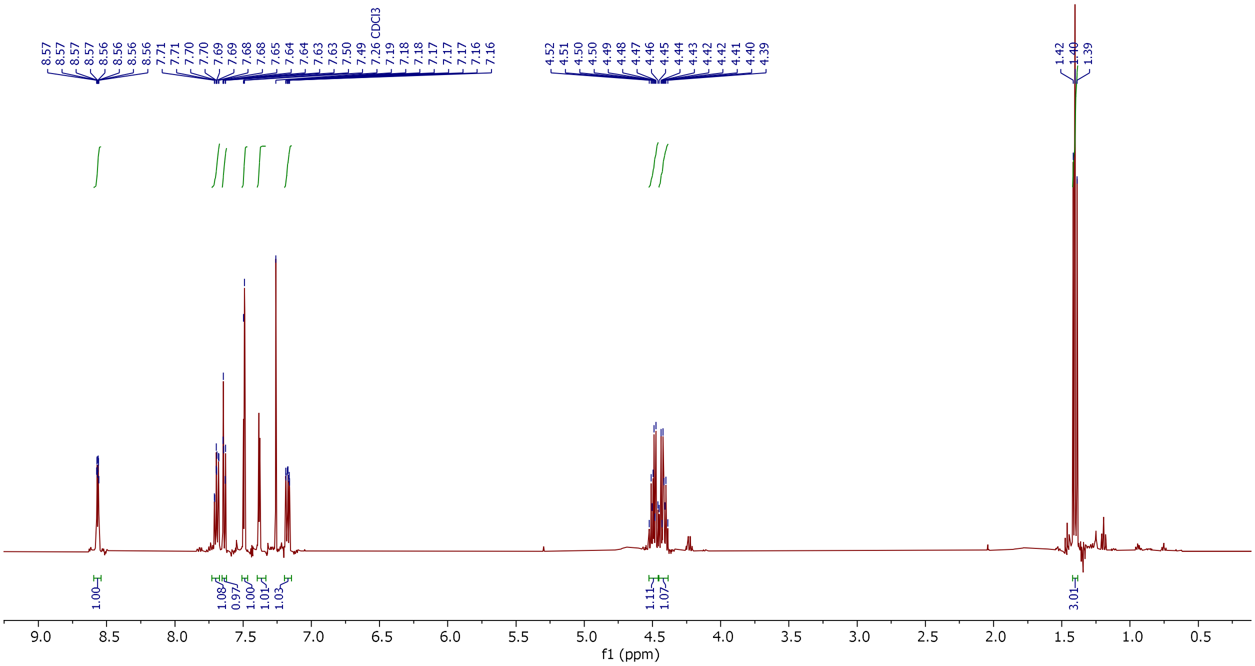

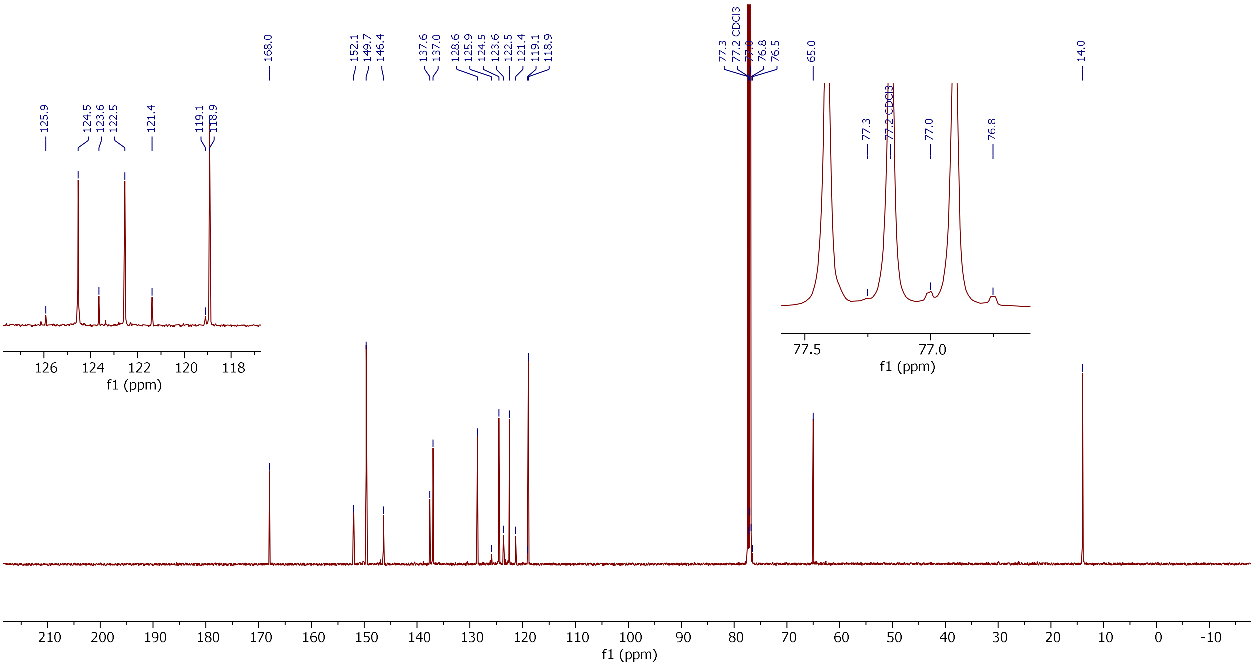

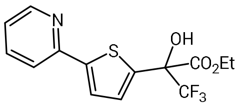

17

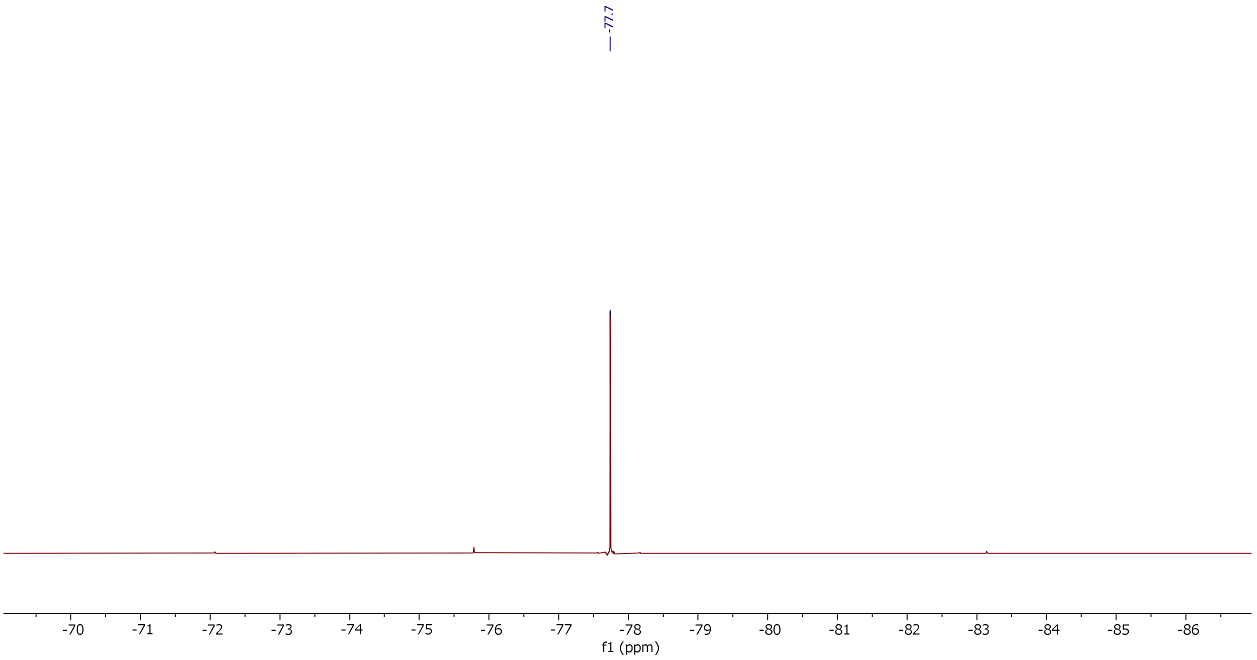

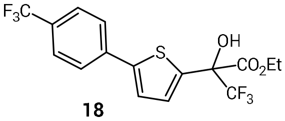

18

ethyl 3,3,3-trifluoro-2-hydroxy-2-(5-(4-(trifluoromethyl)phenyl)thiophen-2-yl)propanoate (18)

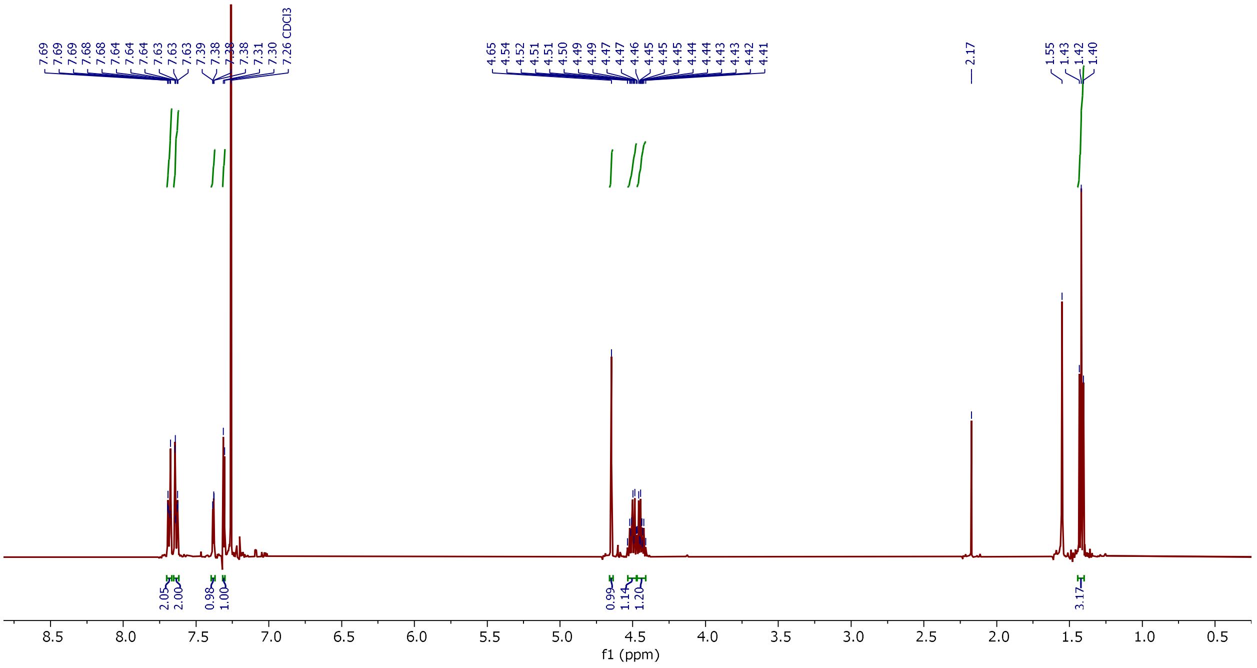

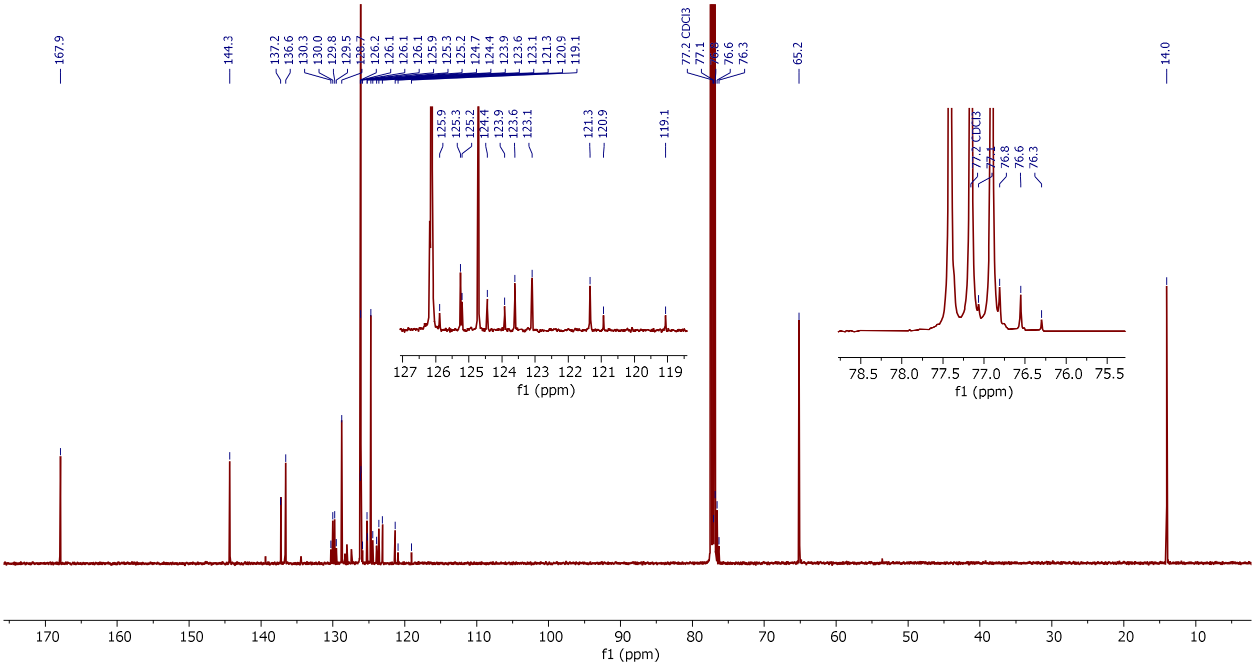

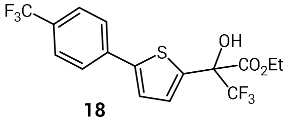

18

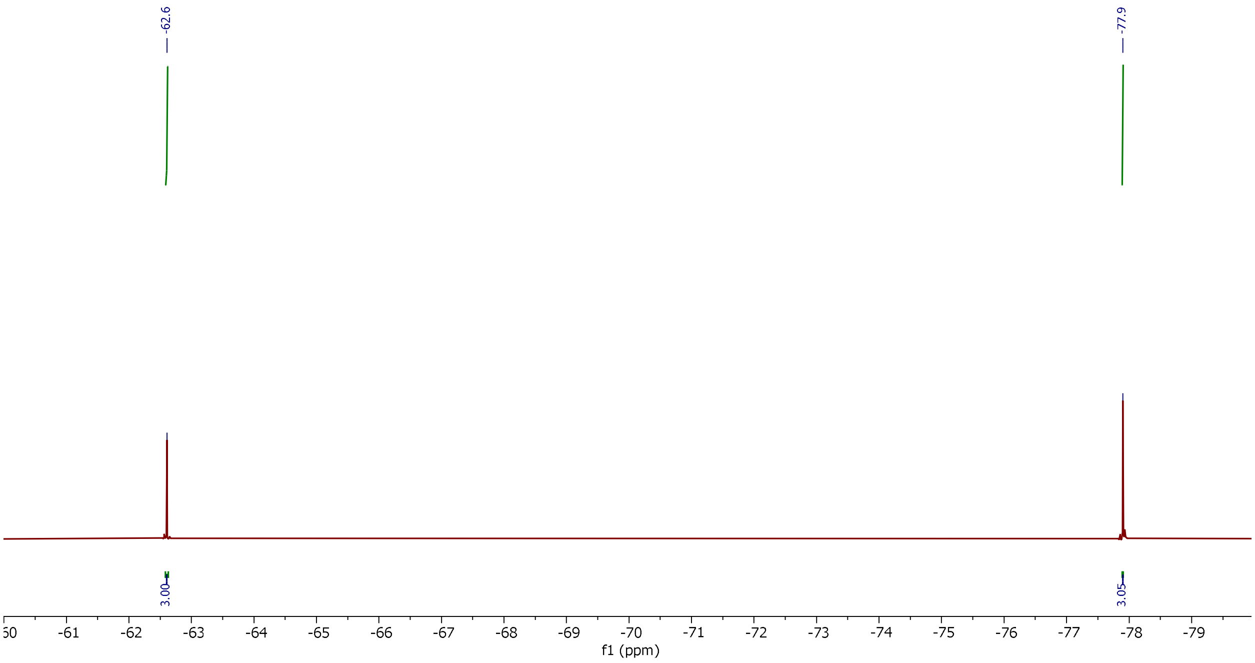
-62.6
-77.9
3.00
3.05
-60
-61
-62
-63
-64
-65
-66
-67
-68
-69
-70
-71
-72
-73
-74
-75
-76
-77
-78
-79
f1 (ppm)

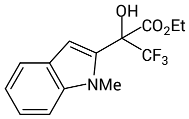

19

ethyl 3,3,3-trifluoro-2-hydroxy-2-(1-methyl-1*H*-indol-2-yl)propanoate (19)

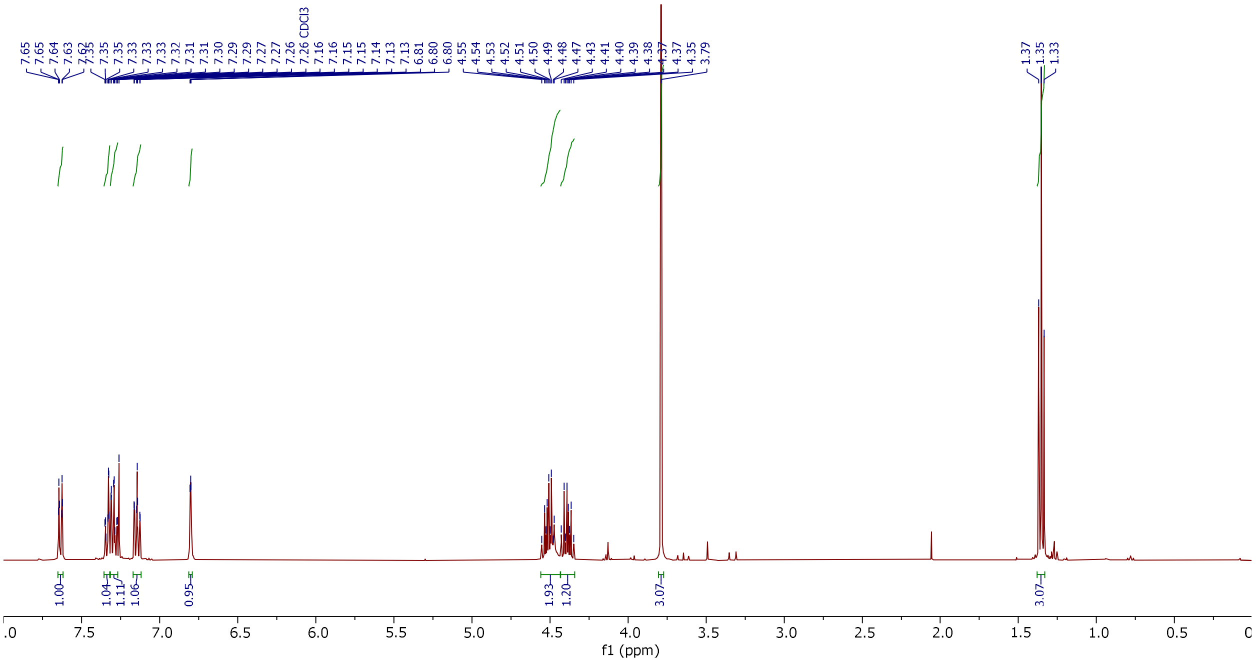

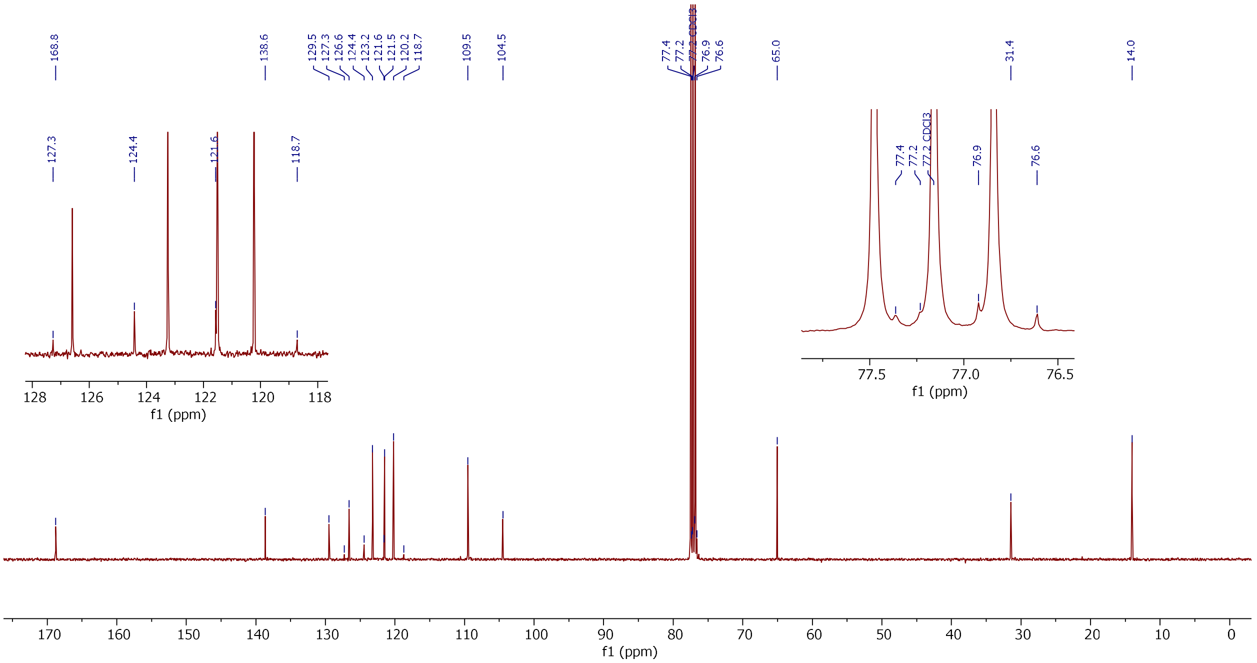

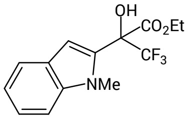

19

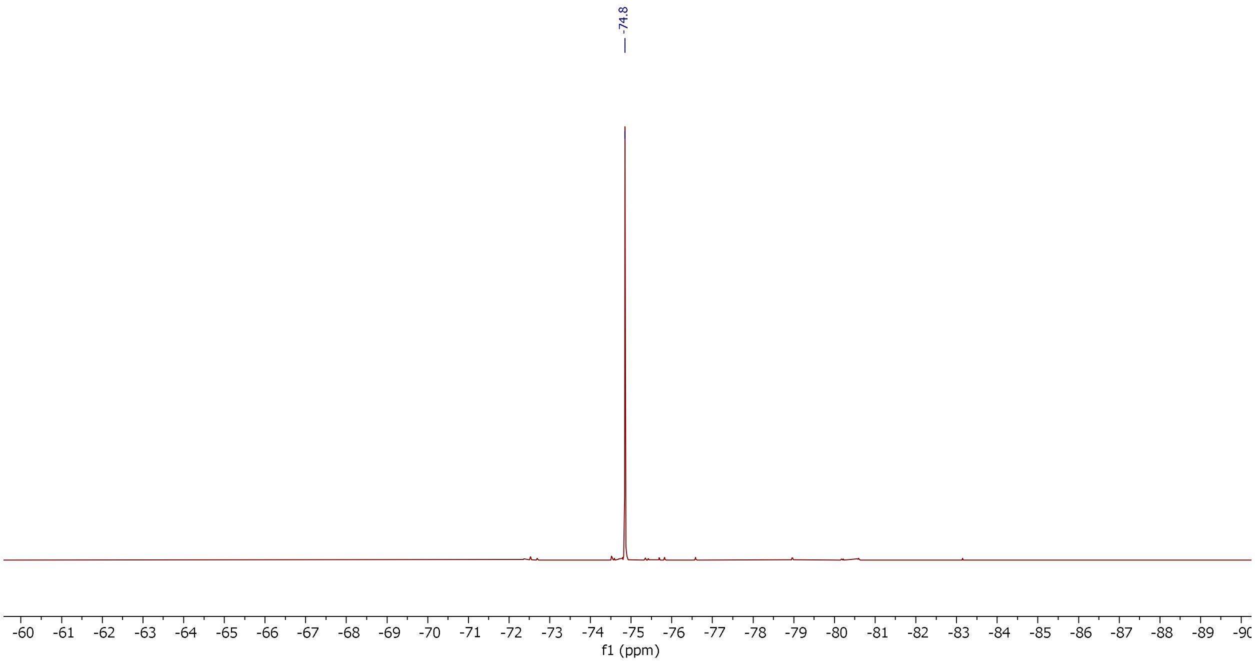

-74.8
-60 -61 -62 -63 -64 -65 -66 -67 -68 -69 -70 -71 -72 -73 -74 -75 -76 -77 -78 -79 -80 -81 -82 -83 -84 -85 -86 -87 -88 -89 -90
f1 (ppm)

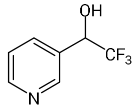

1-TFE

2,2,2-trifluoro-1-(pyridin-3-yl)ethan-1-ol (1-TFE)

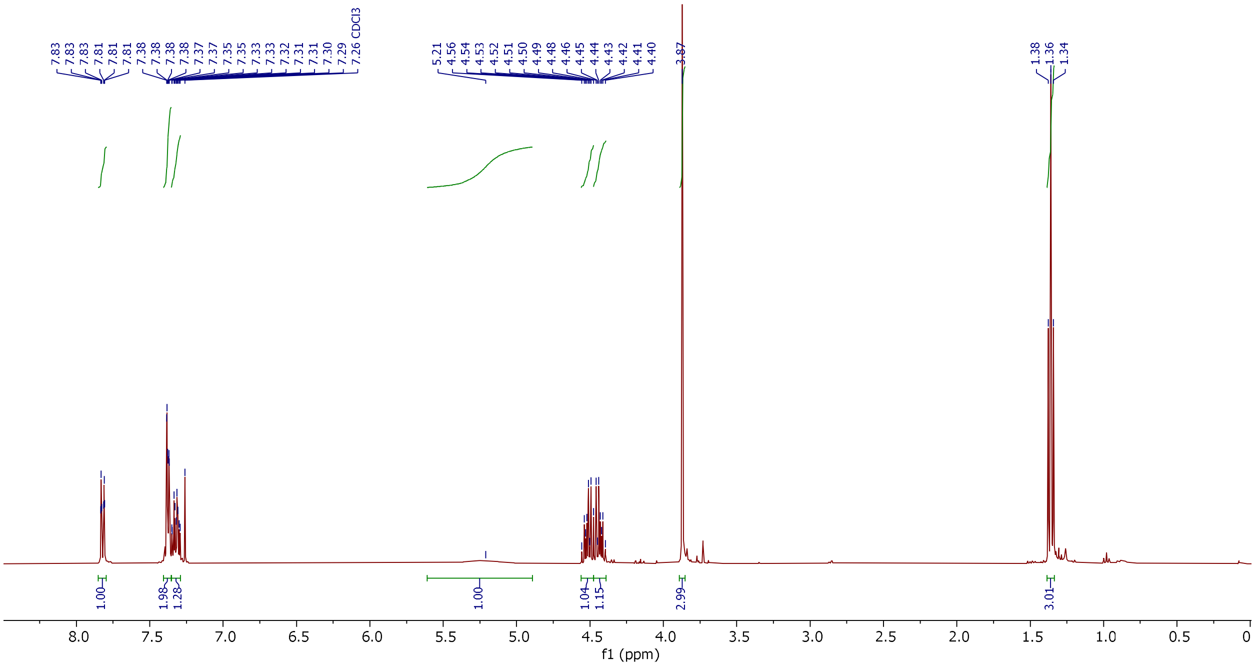

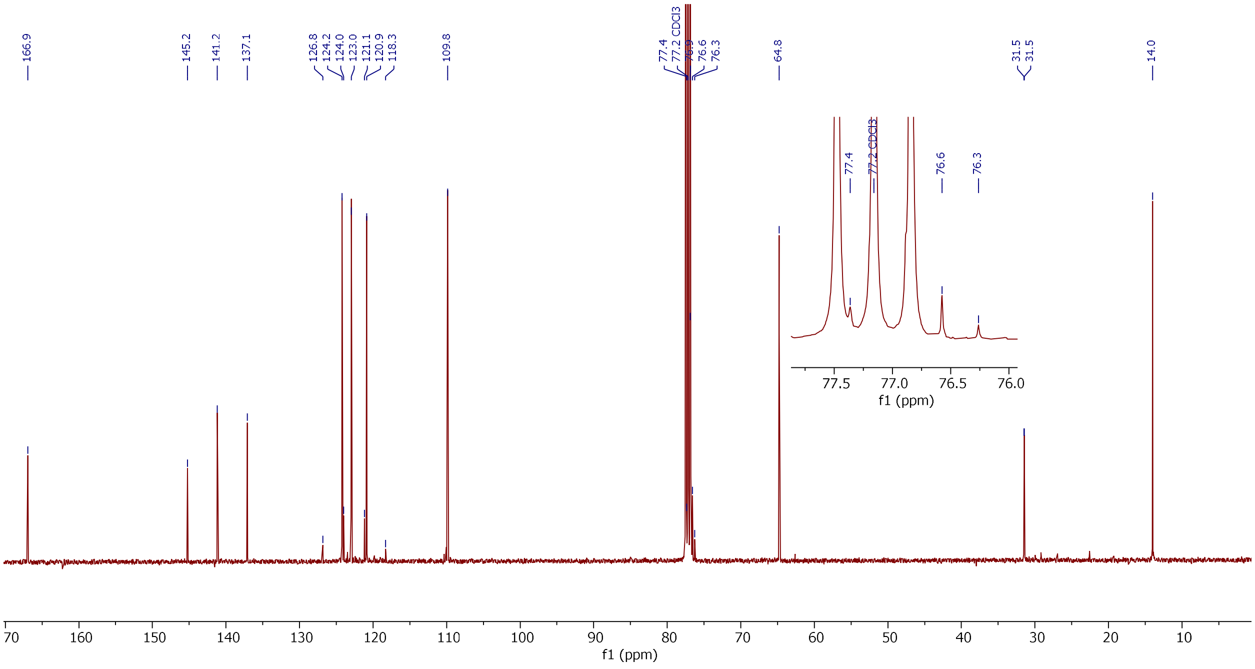

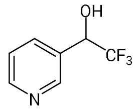

1-TFE

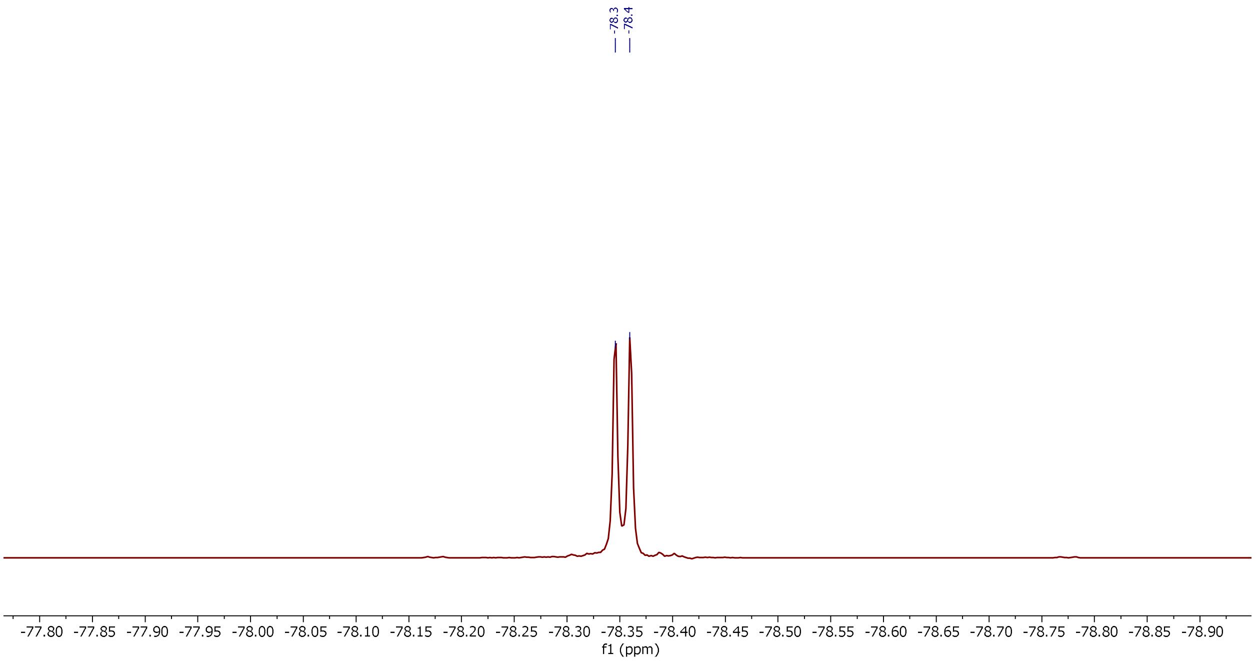
-78.3
-78.4
-77.80 -77.85 -77.90 -77.95 -78.00 -78.05 -78.10 -78.15 -78.20 -78.25 -78.30 -78.35 -78.40 -78.45 -78.50 -78.55 -78.60 -78.65 -78.70 -78.75 -78.80 -78.85 -78.90
f1 (ppm)

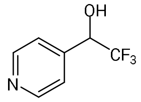

3-TFE

2,2,2-trifluoro-1-(pyridin-4-yl)ethan-1-ol (3-TFE)

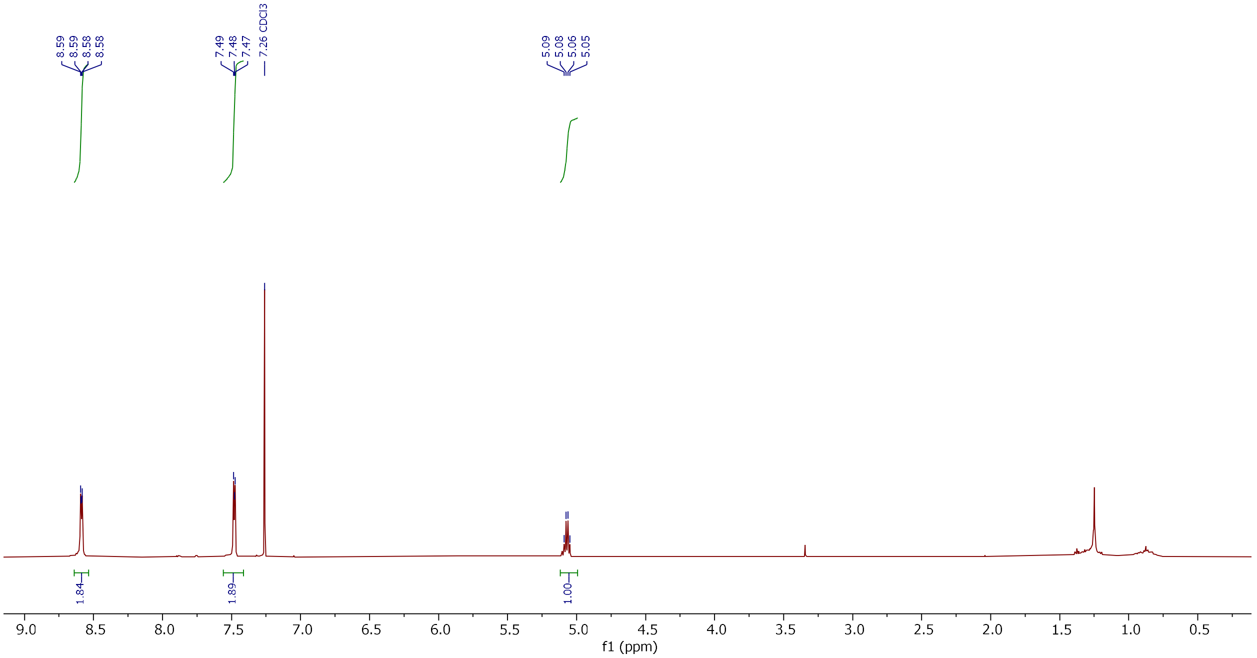

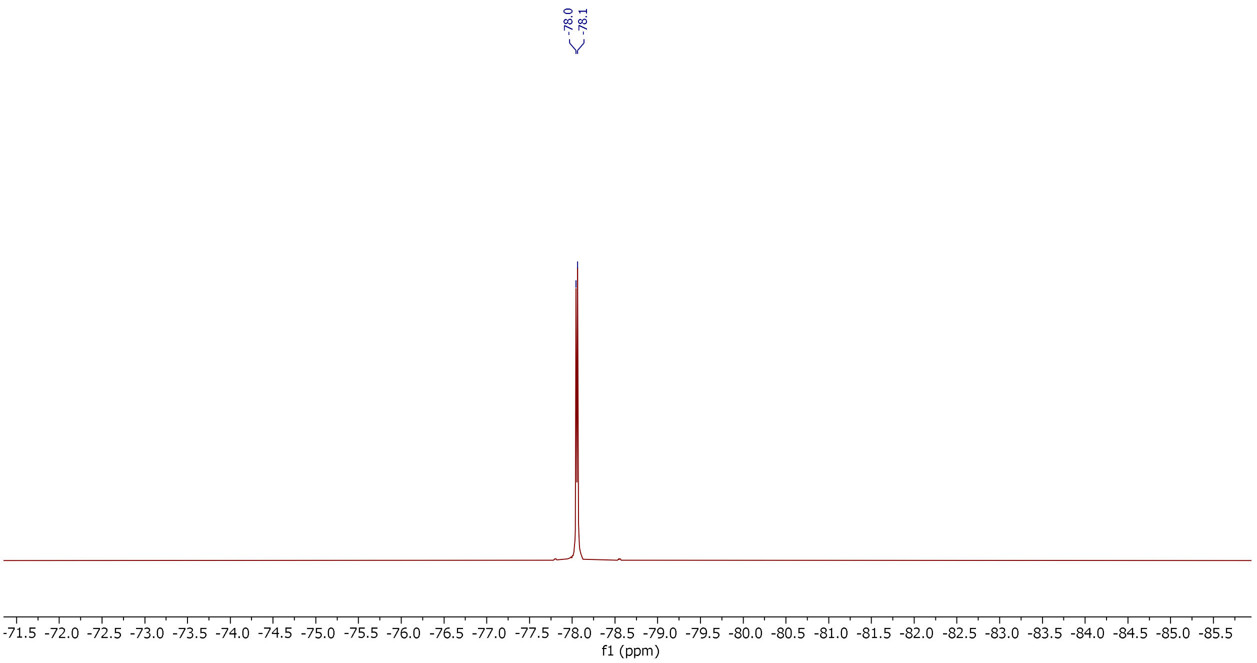

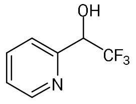

4-TFE

2,2,2-trifluoro-1-(pyridin-2-yl)ethan-1-ol (4-TFE)

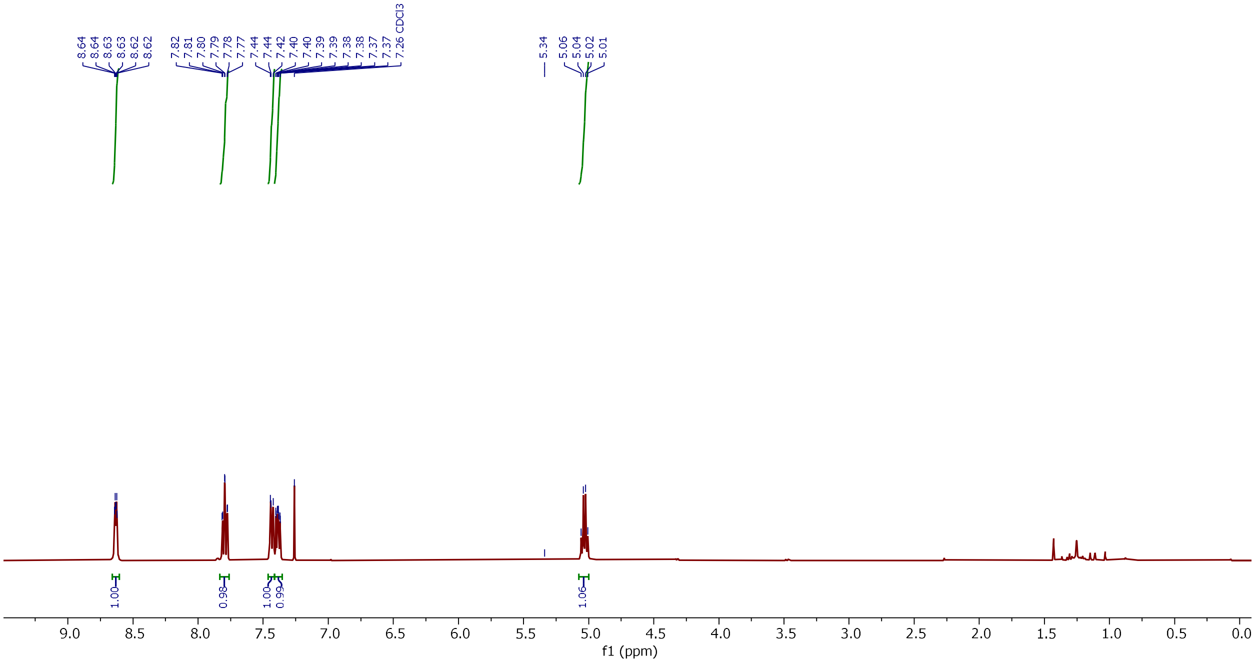

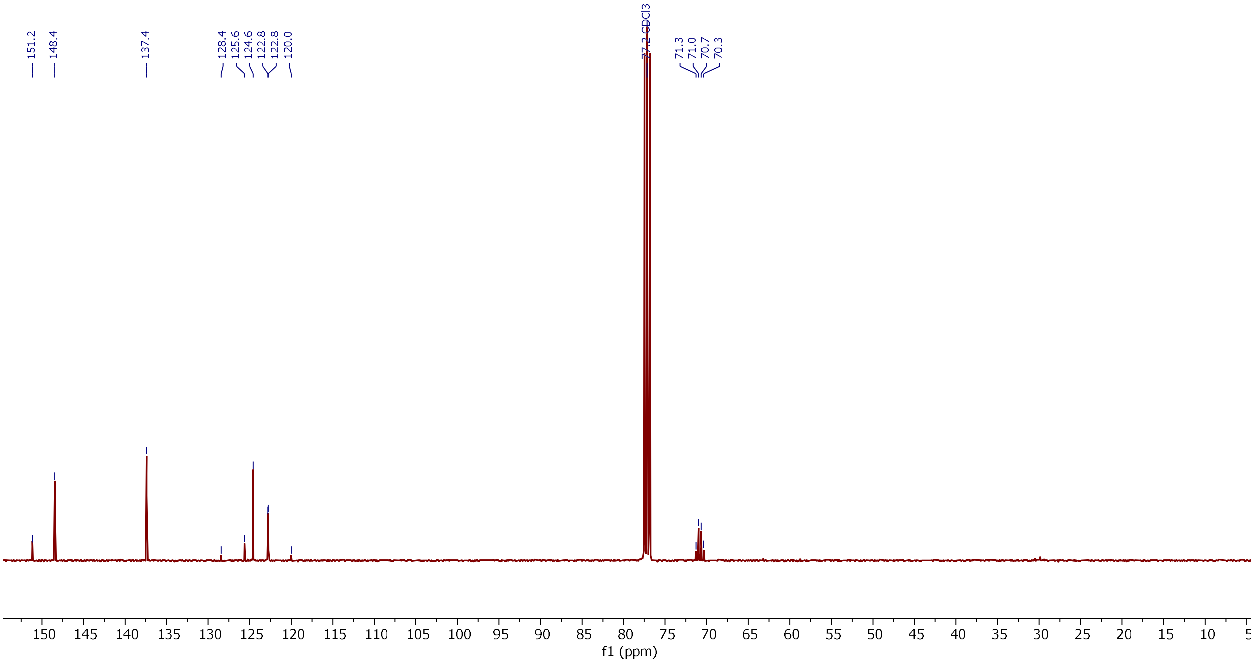

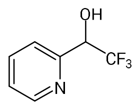
OH
CF3
N

4-TFE

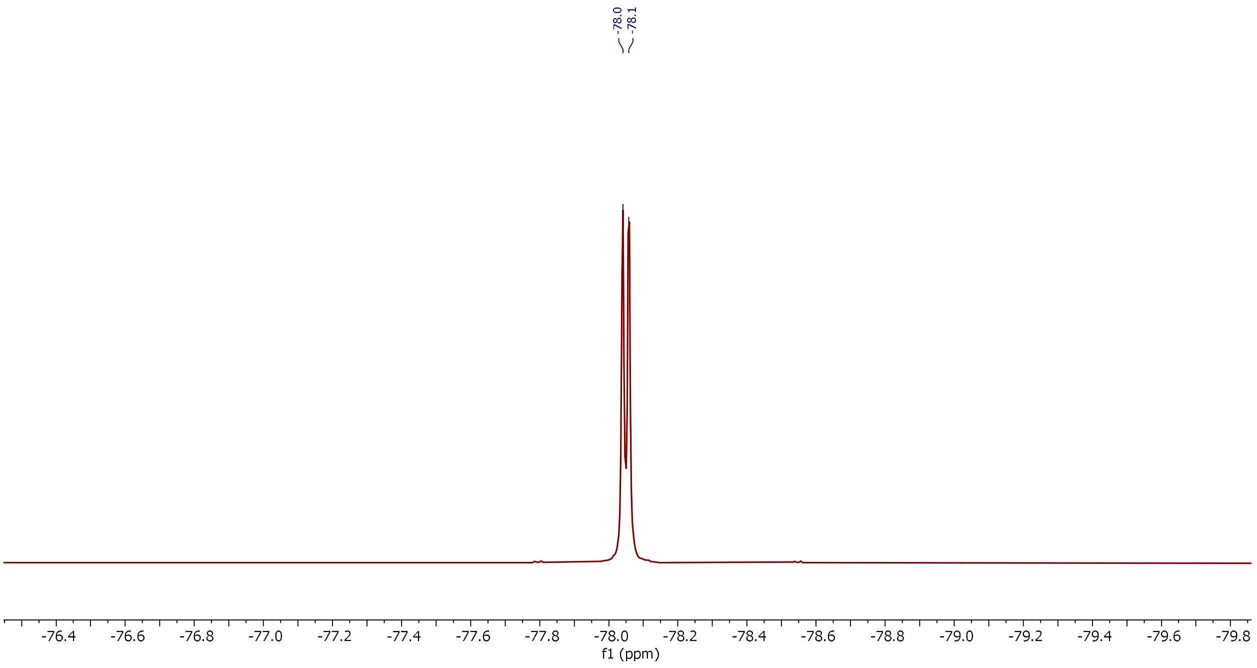
-78.0
-78.1
-76.4
-76.6
-76.8
-77.0
-77.2
-77.4
-77.6
-77.8
-78.0
-78.2
-78.4
-78.6
-78.8
-79.0
-79.2
-79.4
-79.6
-79.8
f1 (ppm)

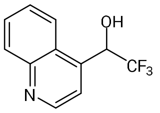

5-TFE

2,2,2-trifluoro-1-(quinolin-4-yl)ethan-1-ol (5-TFE)

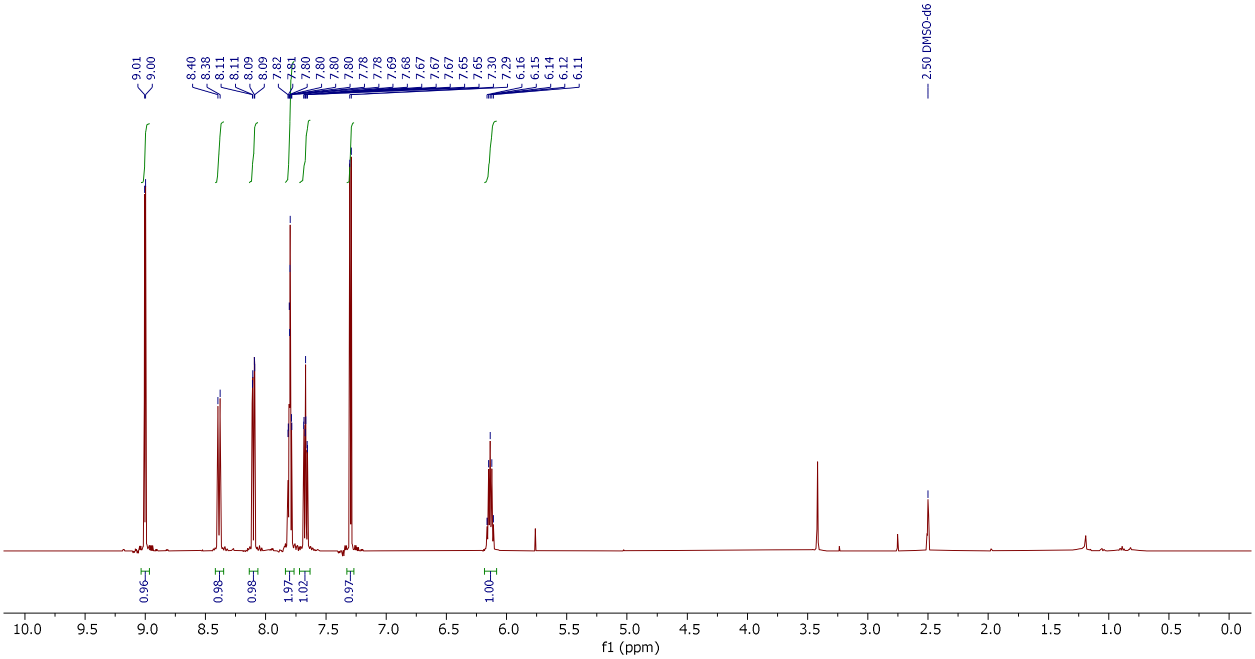

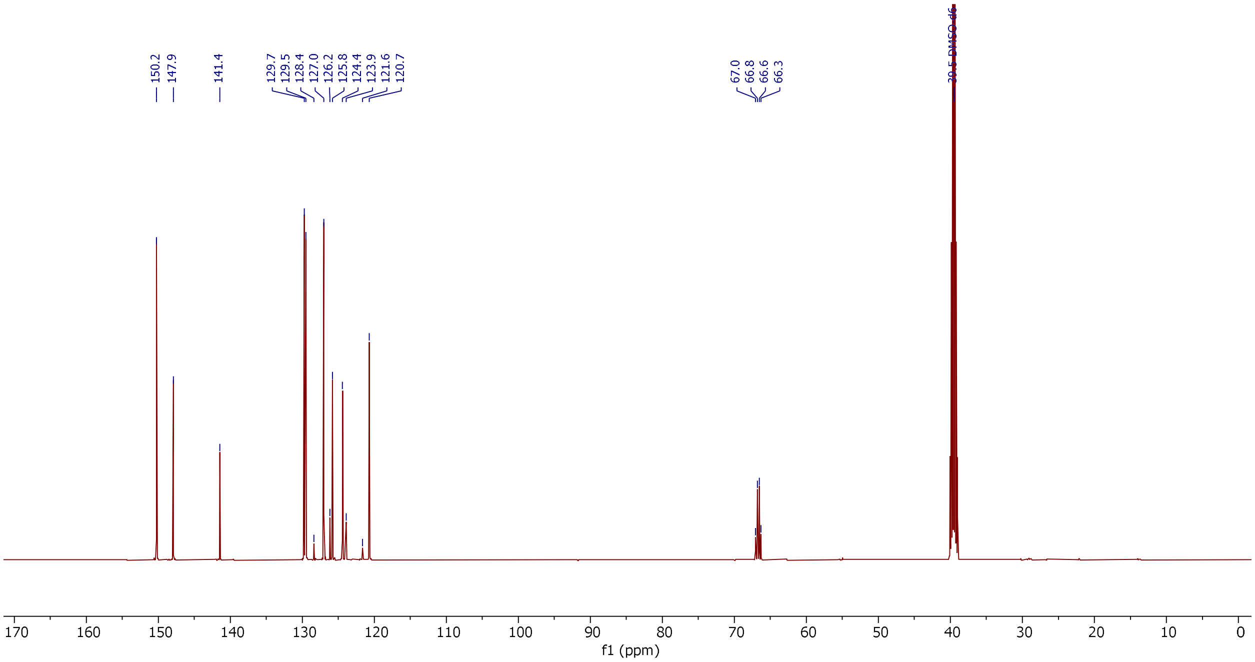

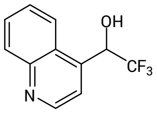

5-TFE

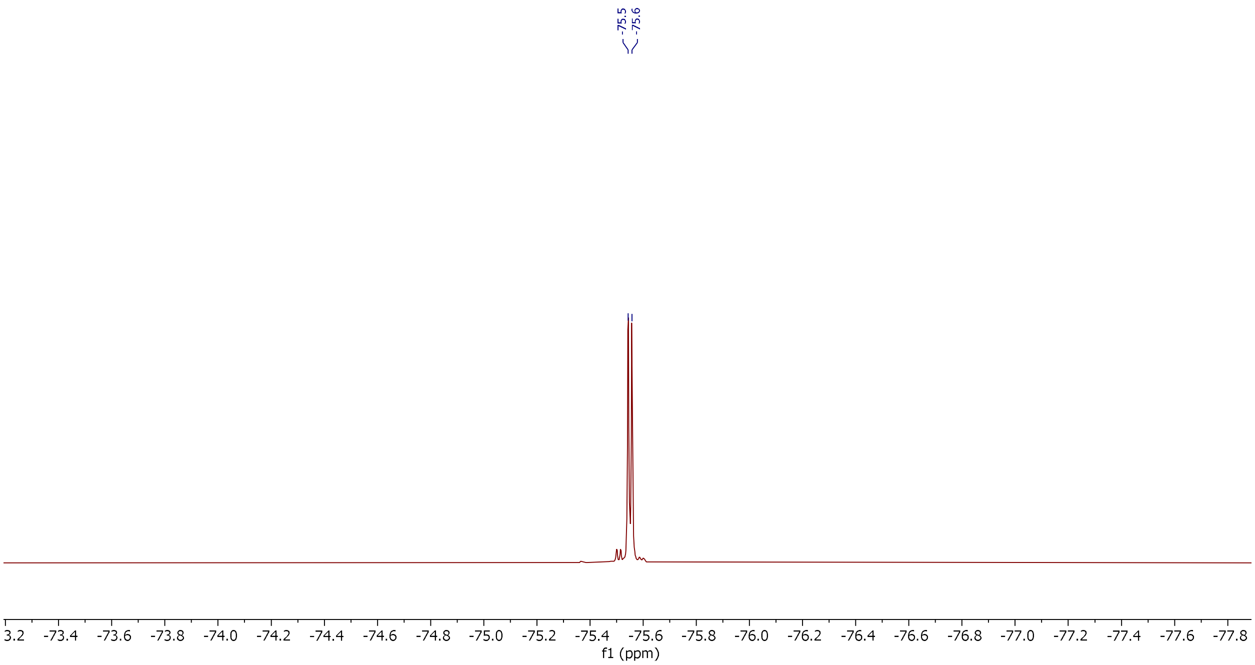

-75.5
-75.6
3.2 -73.4 -73.6 -73.8 -74.0 -74.2 -74.4 -74.6 -74.8 -75.0 -75.2 -75.4 -75.6 -75.8 -76.0 -76.2 -76.4 -76.6 -76.8 -77.0 -77.2 -77.4 -77.6 -77.8
f1 (ppm)

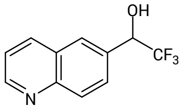

6-TFE

2,2,2-trifluoro-1-(quinolin-6-yl)ethan-1-ol (6-TFE)

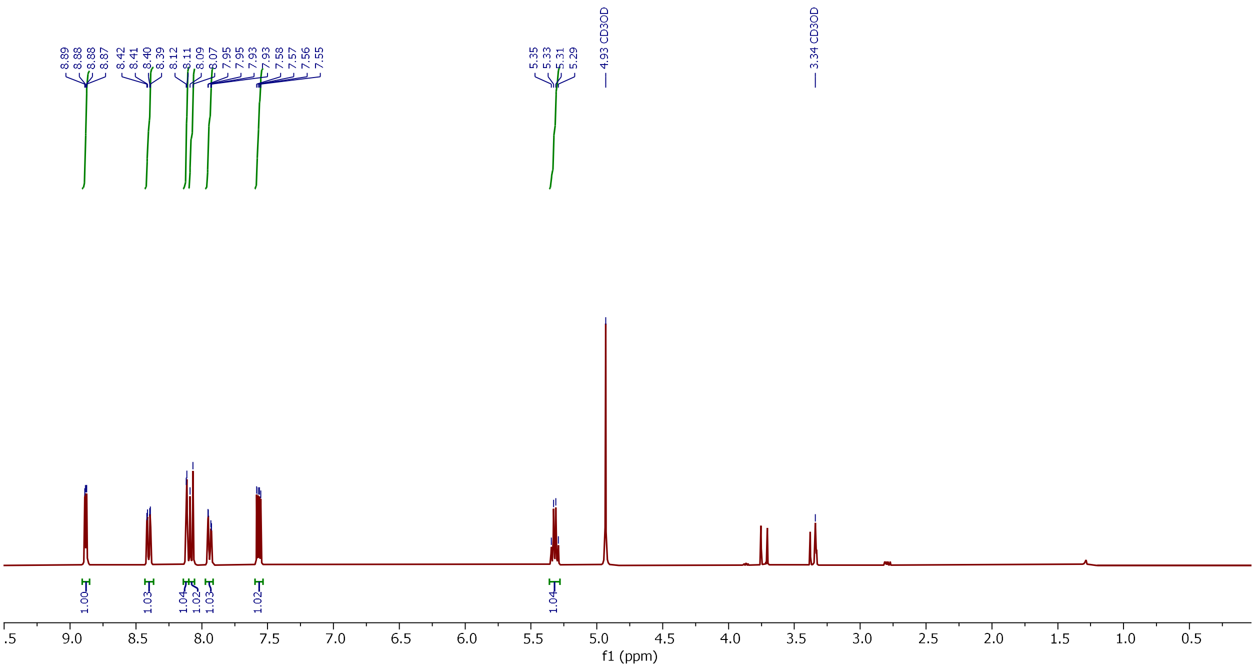

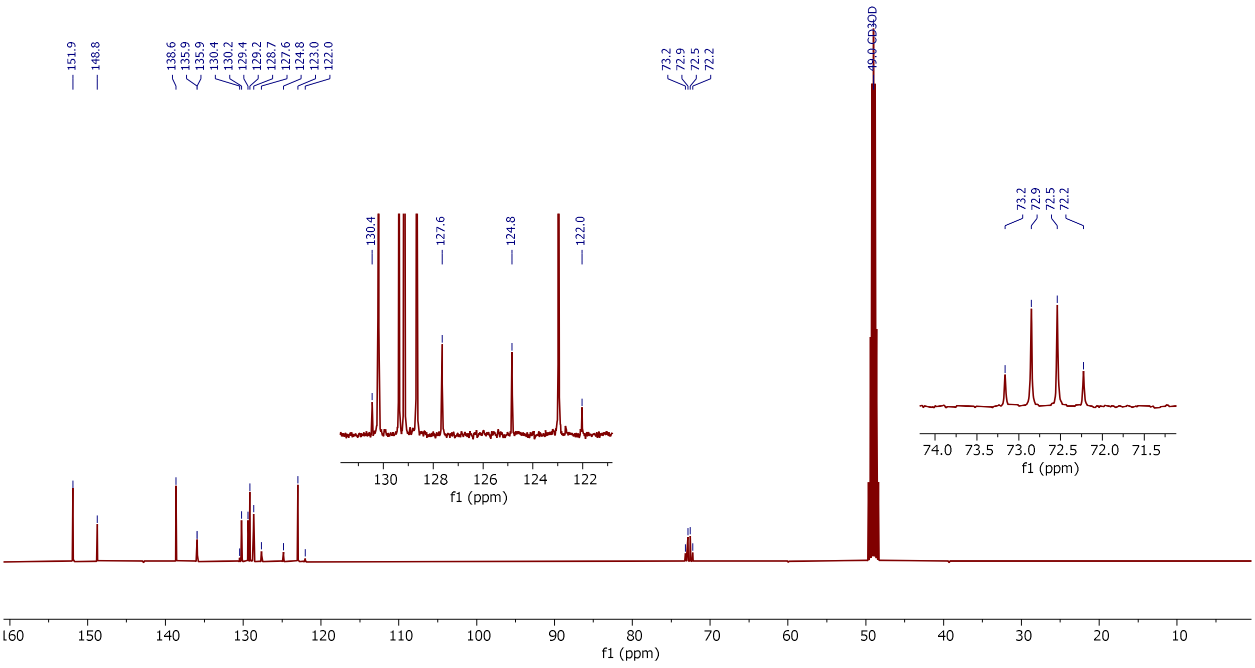

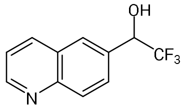

6-TFE

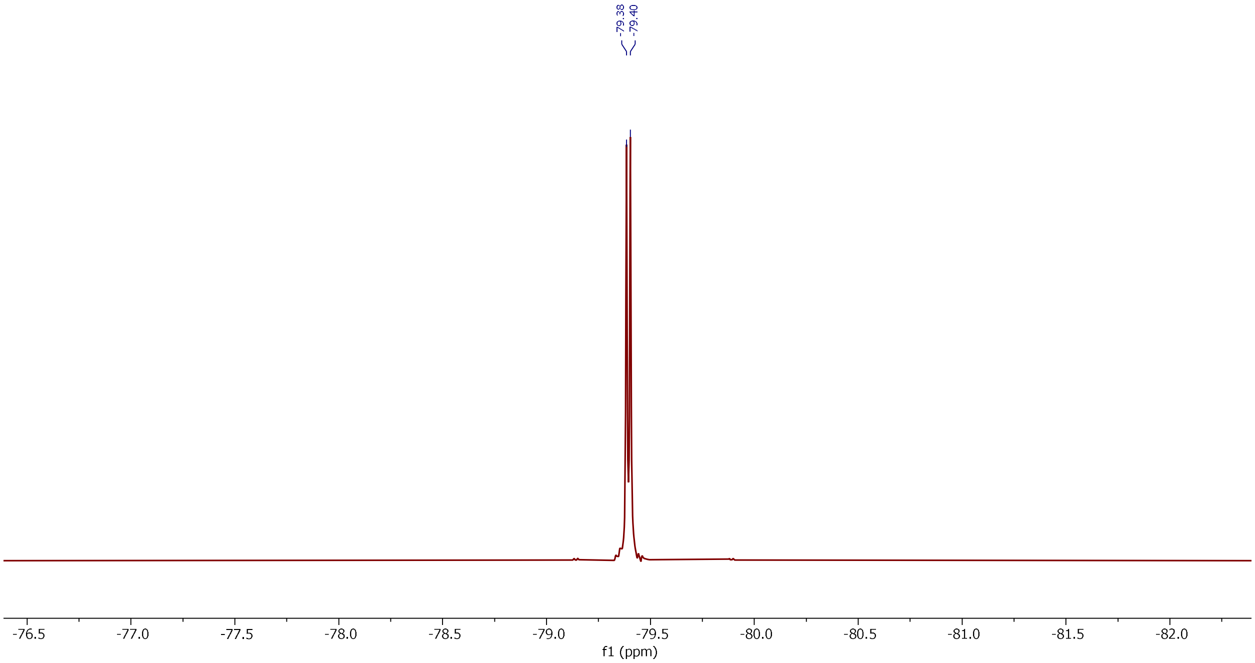

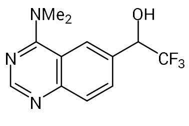

7-TFE

1-(4-(dimethylamino)quinazolin-6-yl)-2,2,2-trifluoroethan-1-ol (7-TFE)

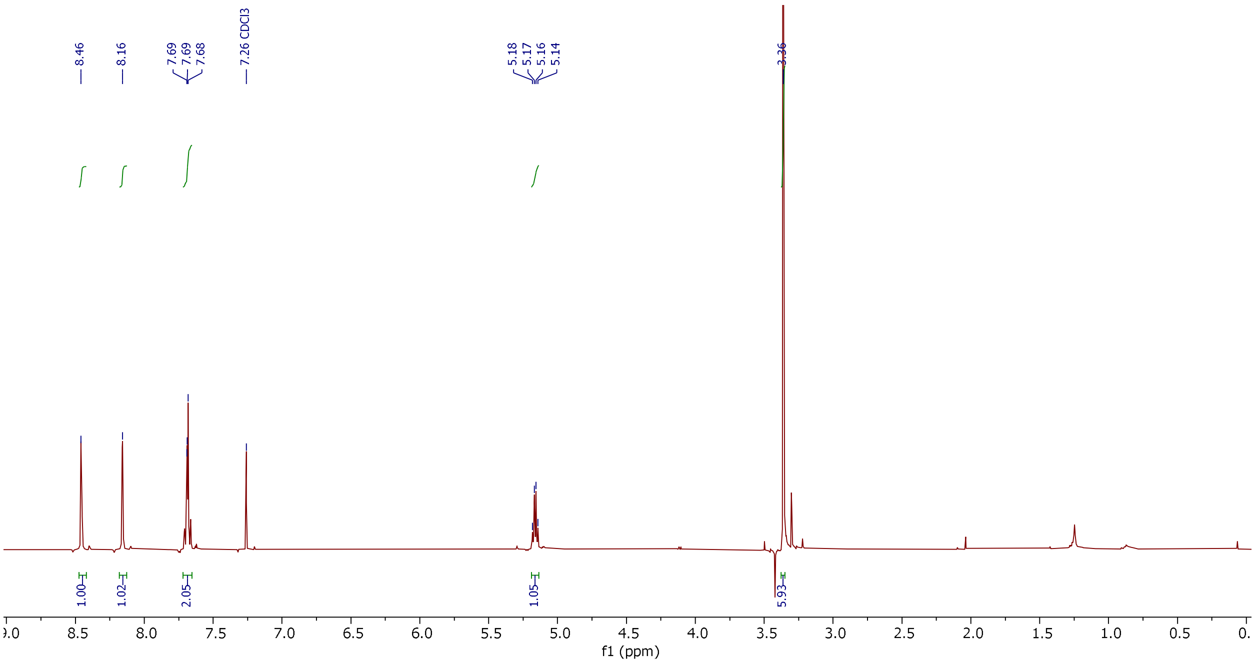

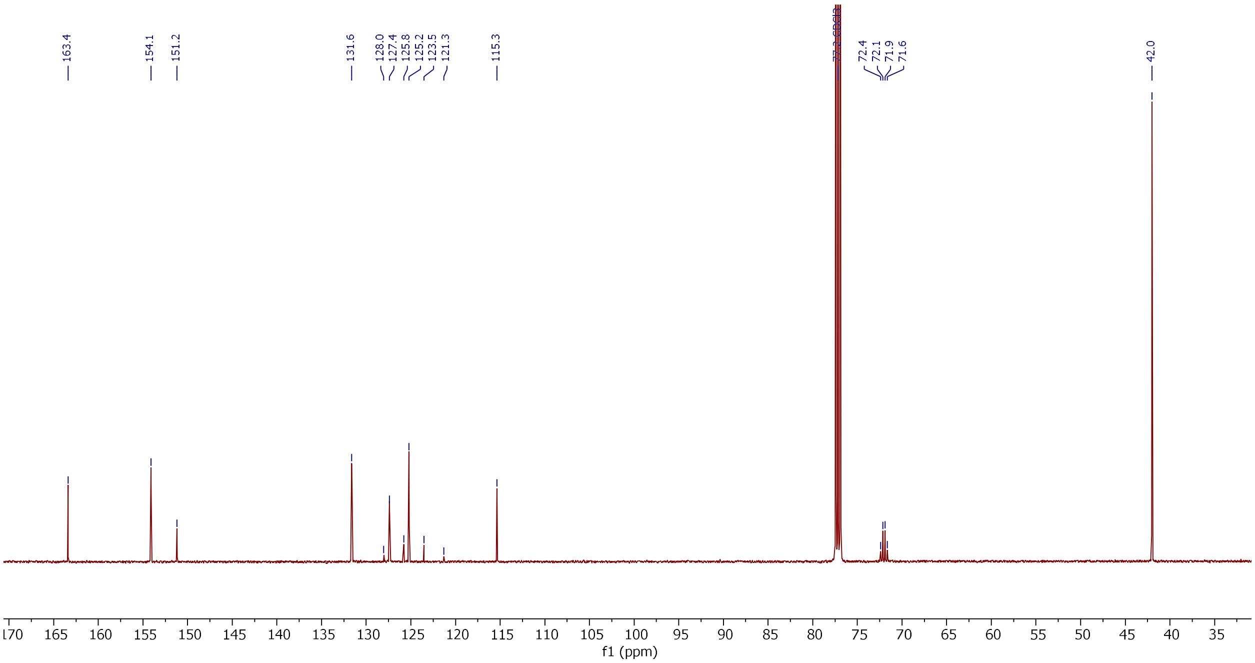

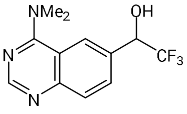
NMe2
OH
N
CF3
N

7-TFE

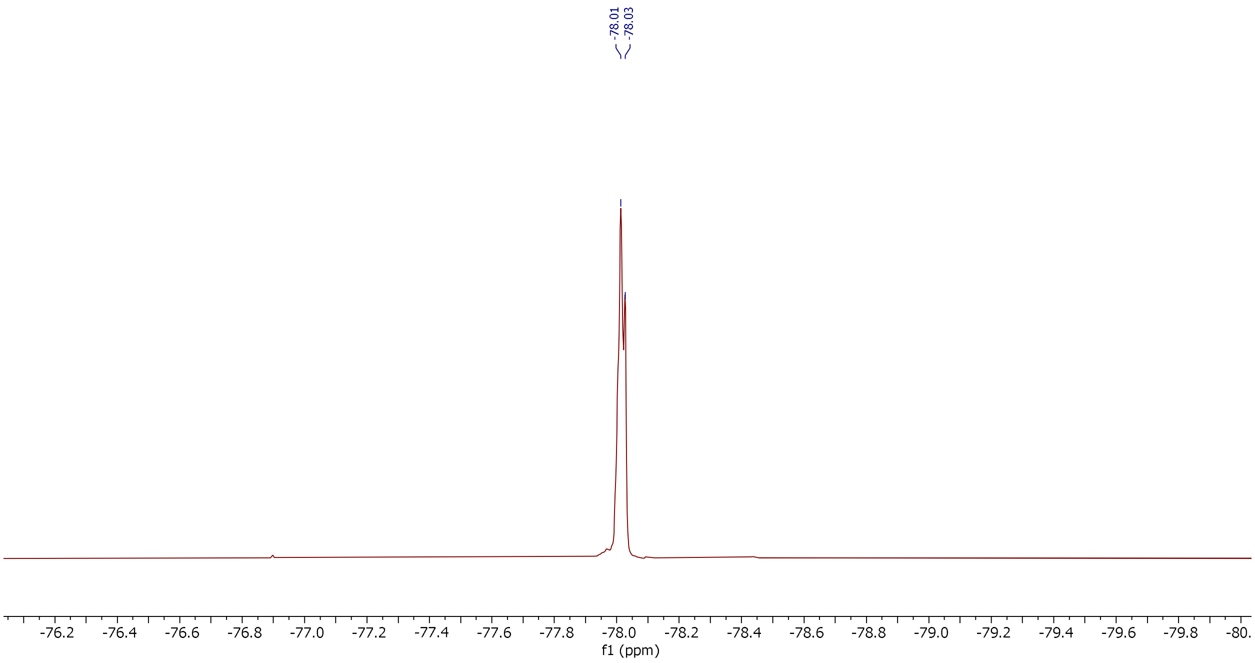
-78.01
-78.03
-76.2
-76.4
-76.6
-76.8
-77.0
-77.2
-77.4
-77.6
-77.8
-78.0
-78.2
-78.4
-78.6
-78.8
-79.0
-79.2
-79.4
-79.6
-79.8
-80.
f1 (ppm)

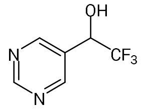

8-TFE

2,2,2-trifluoro-1-(pyrimidin-5-yl)ethan-1-ol (8-TFE)

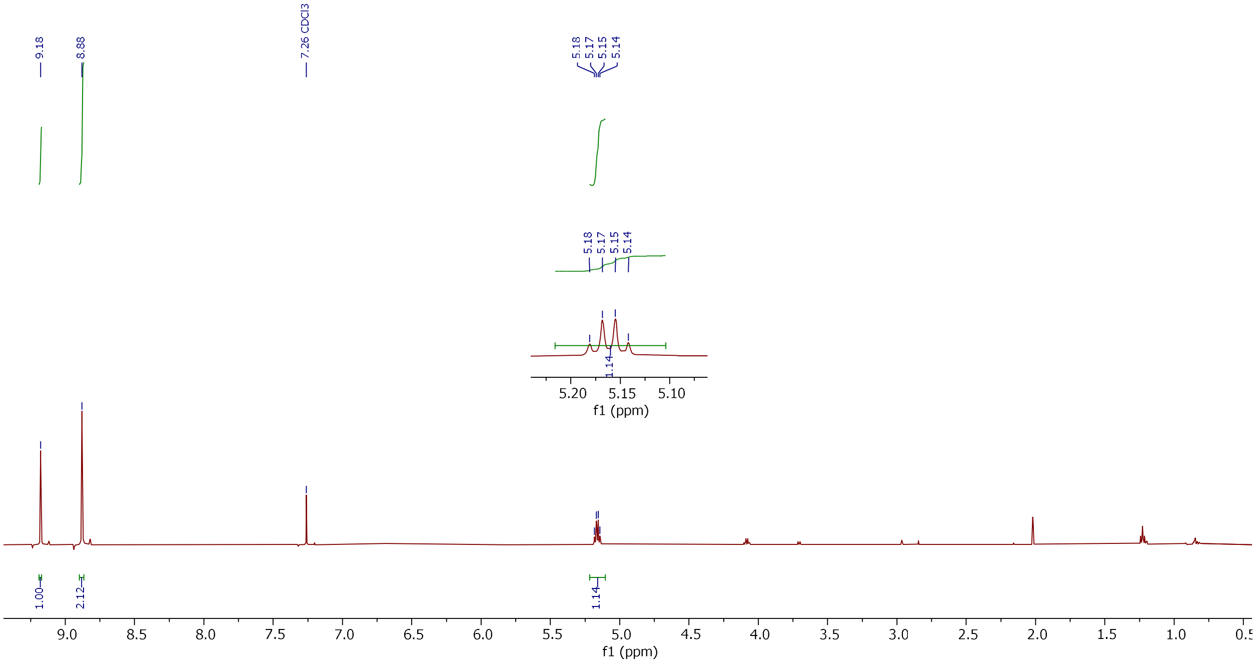

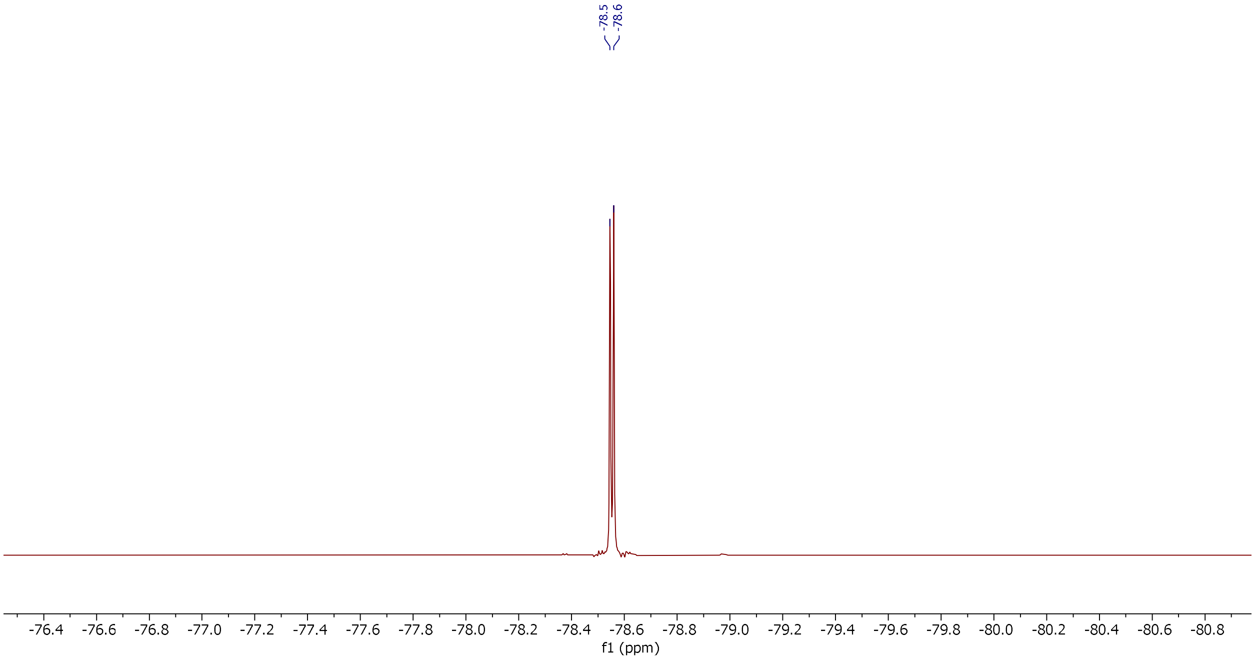

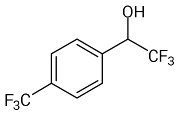

9-TFE

2,2,2-trifluoro-1-(4-(trifluoromethyl)phenyl)ethan-1-ol (9-TFE)

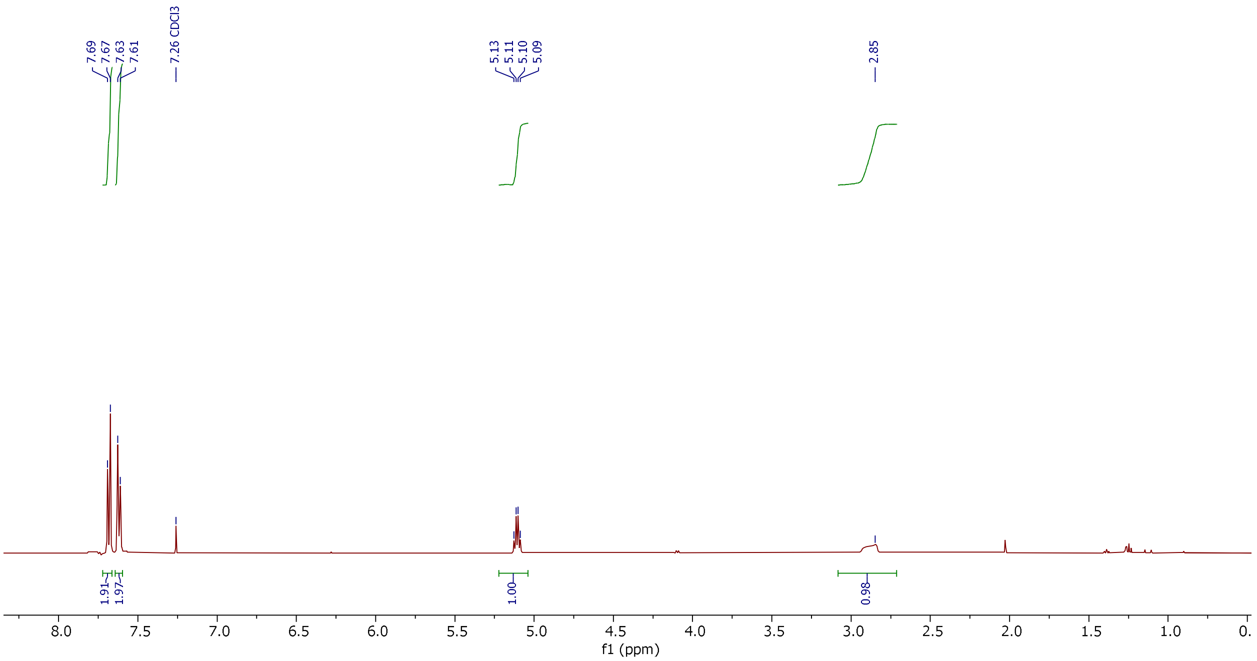

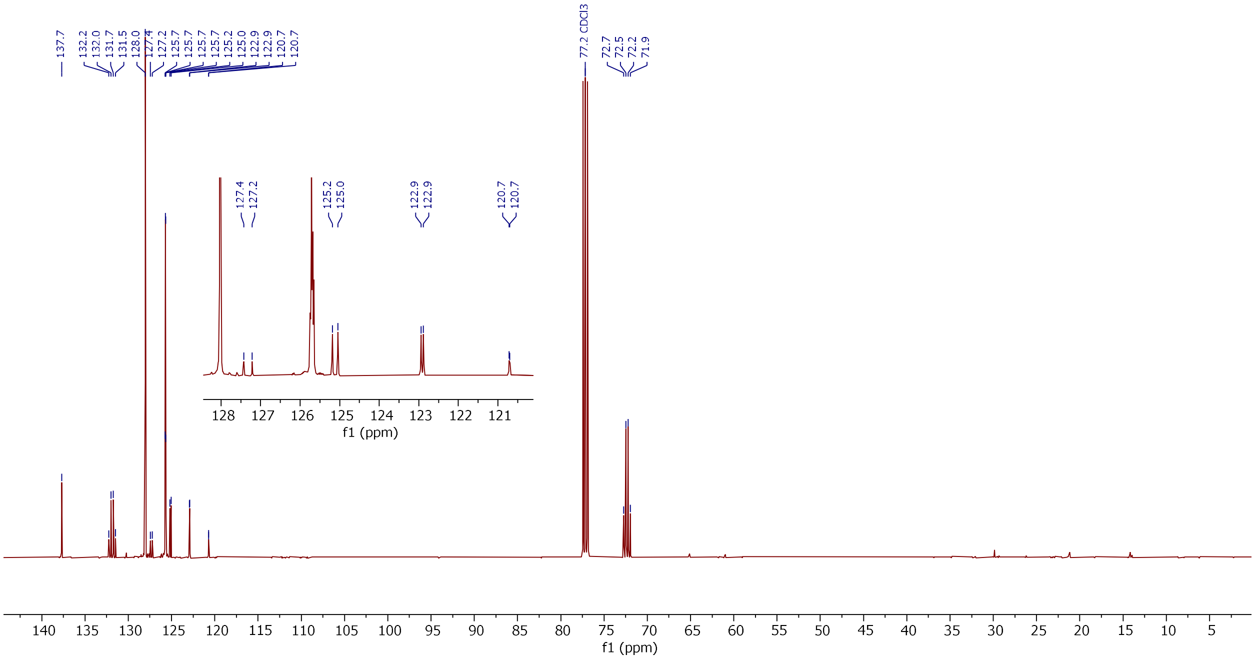

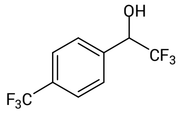

9-TFE

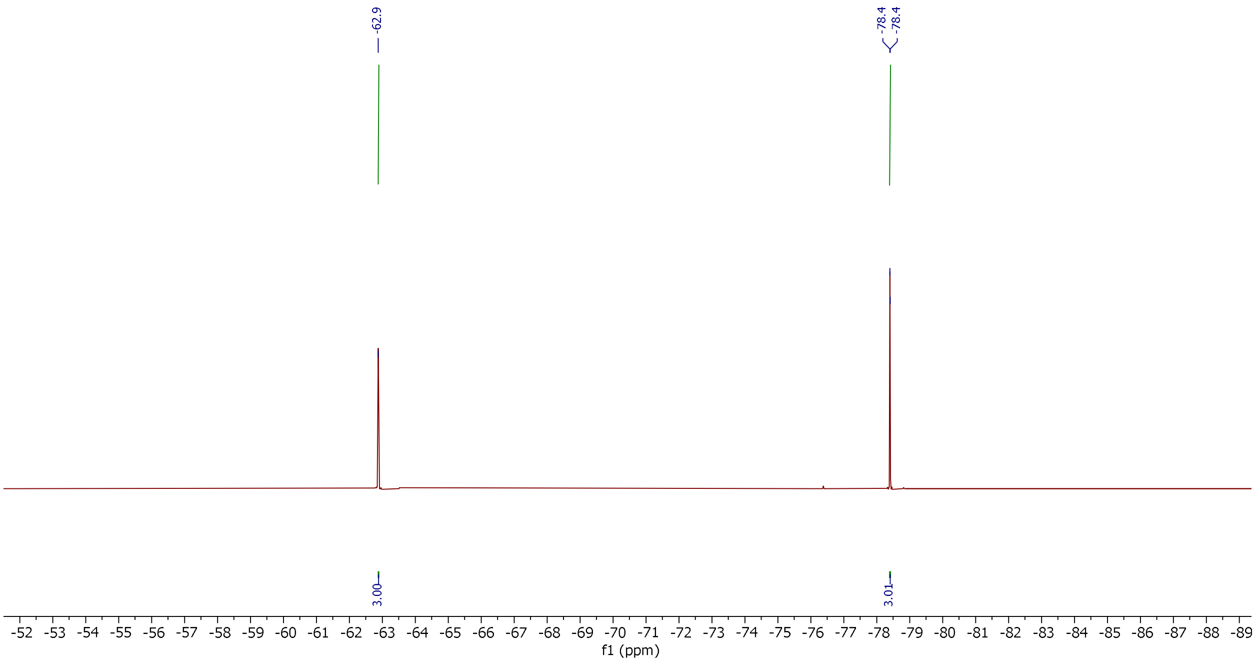
-62.9
-78.4
-78.4
3.00
3.01
-52 -53 -54 -55 -56 -57 -58 -59 -60 -61 -62 -63 -64 -65 -66 -67 -68 -69 -70 -71 -72 -73 -74 -75 -76 -77 -78 -79 -80 -81 -82 -83 -84 -85 -86 -87 -88 -89
f1 (ppm)

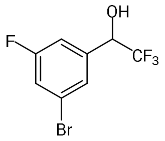

10-TFE

1-(3-bromo-5-fluorophenyl)-2,2,2-trifluoroethan-1-ol (10-TFE)

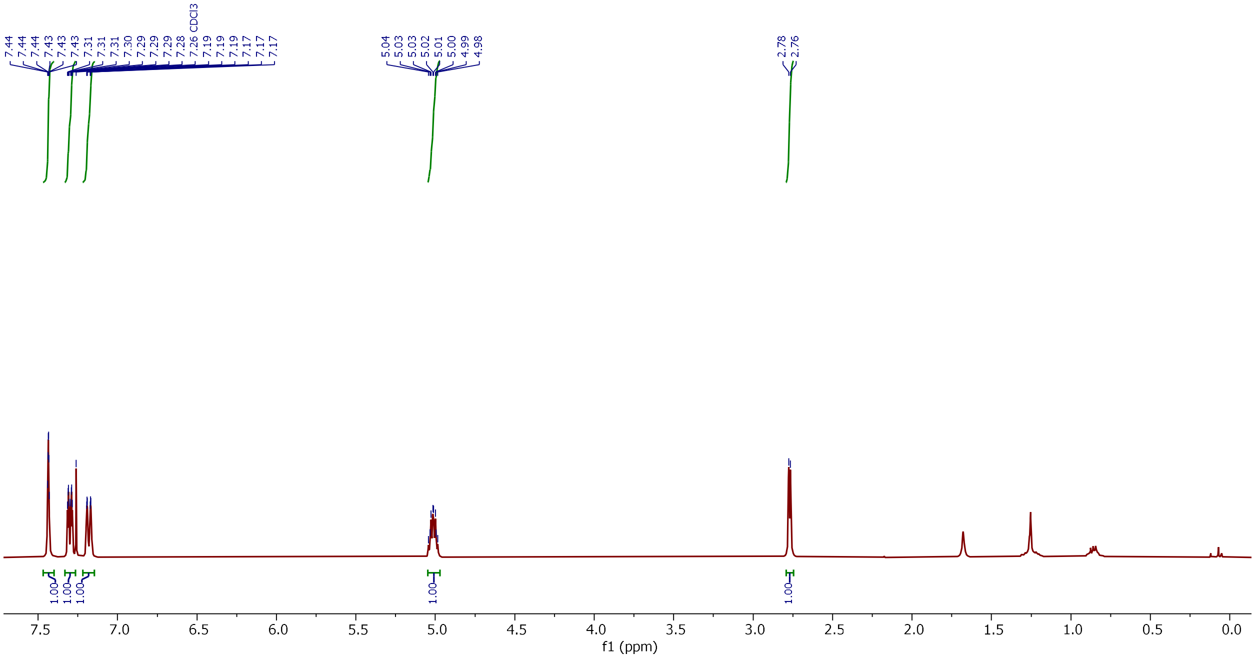

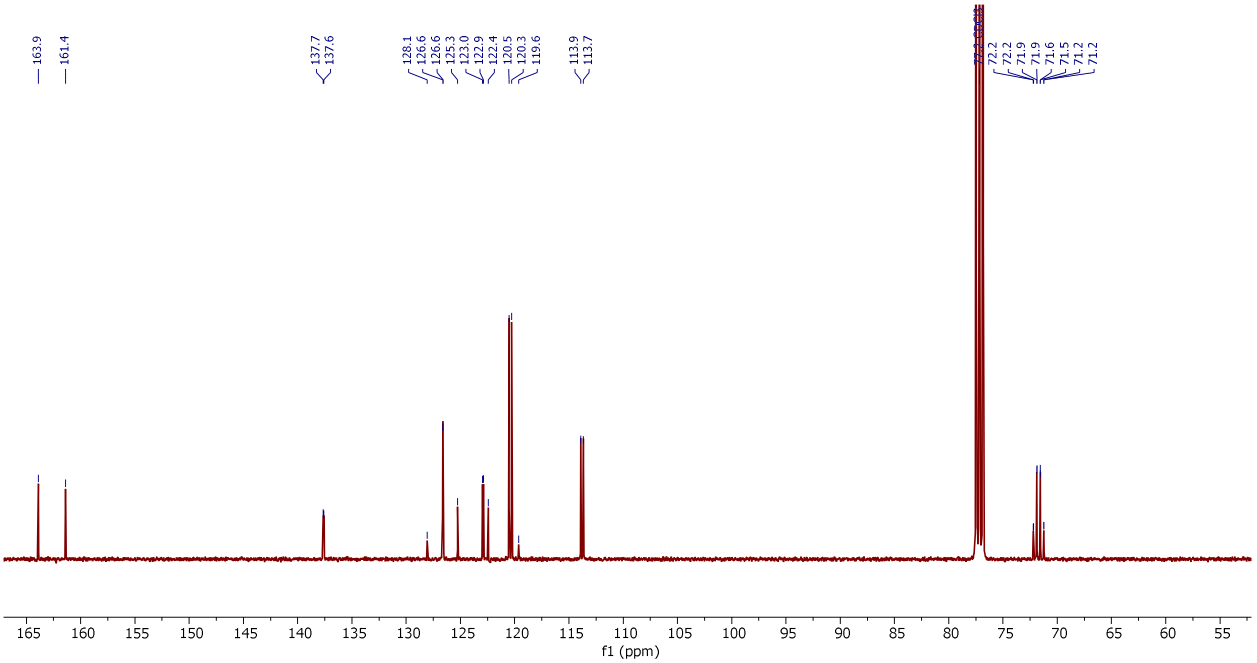

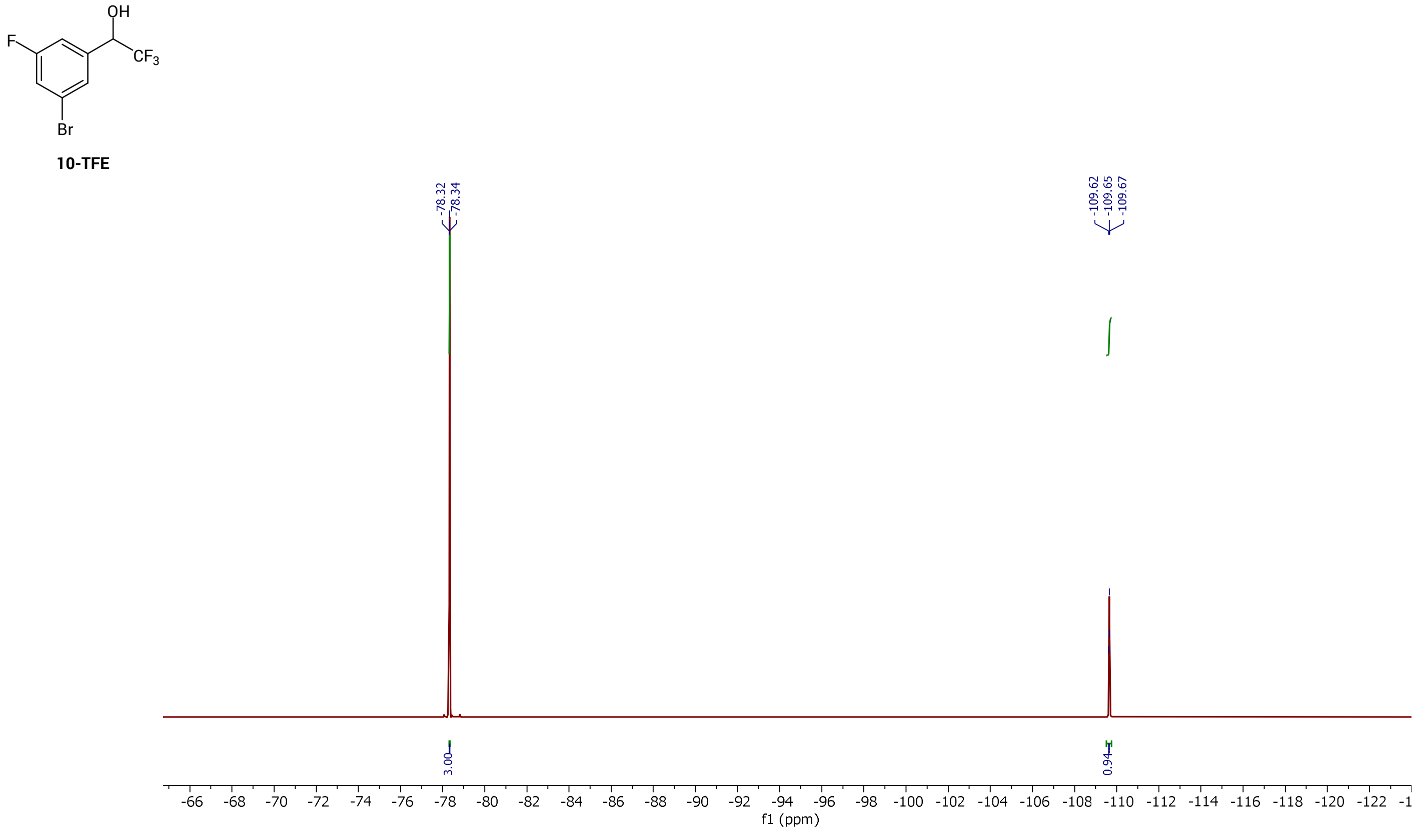

10-TFE

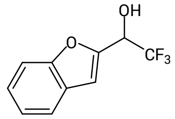

11-TFE

1-(benzofuran-2-yl)-2,2,2-trifluoroethan-1-ol (11-TFE)

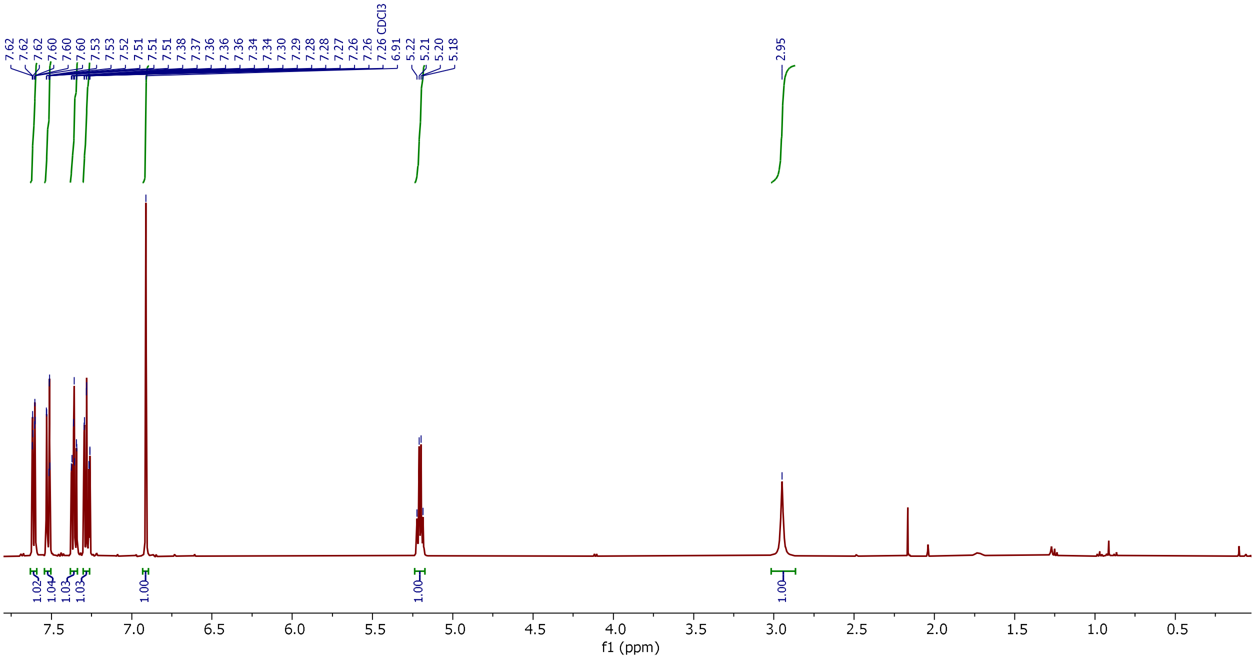

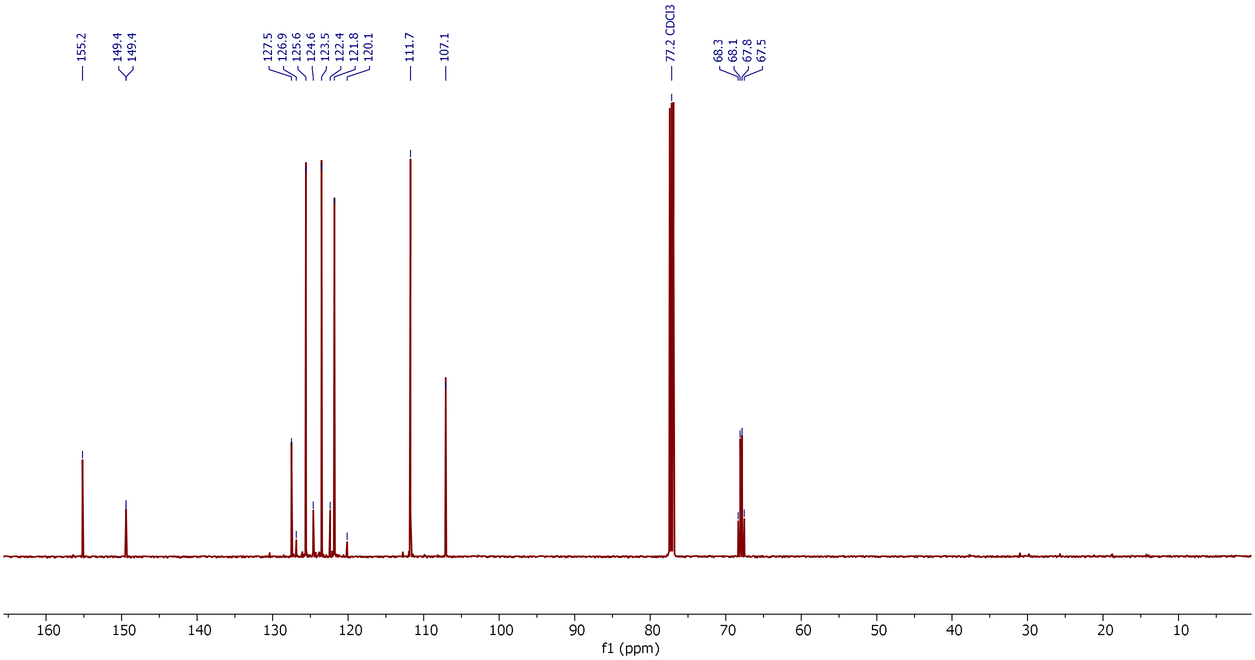

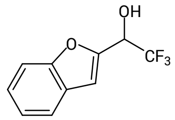
OH
O
CF3

11-TFE

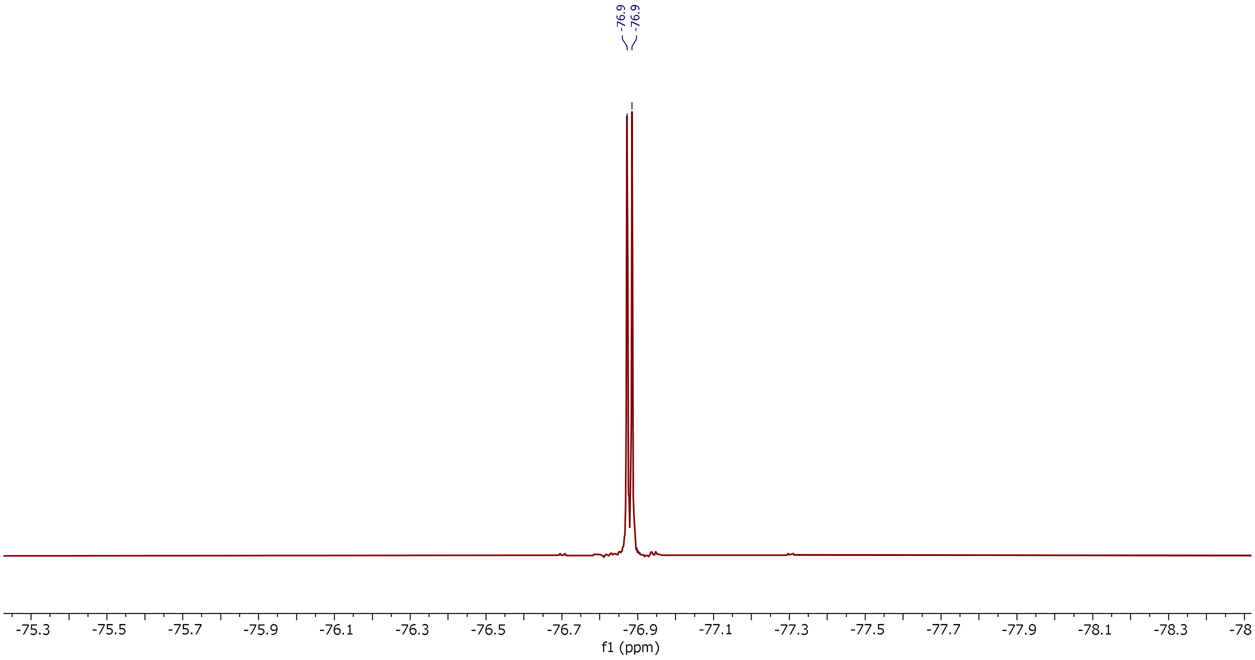
-76.9
-76.9
-75.3
-75.5
-75.7
-75.9
-76.1
-76.3
-76.5
-76.7
-76.9
-77.1
-77.3
-77.5
-77.7
-77.9
-78.1
-78.3
-78
f1 (ppm)

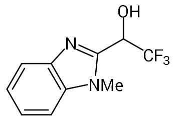

12-TFE

2,2,2-trifluoro-1-(1-methyl-1*H*-benzo[*d*]imidazol-2-yl)ethan-1-ol (12-TFE)

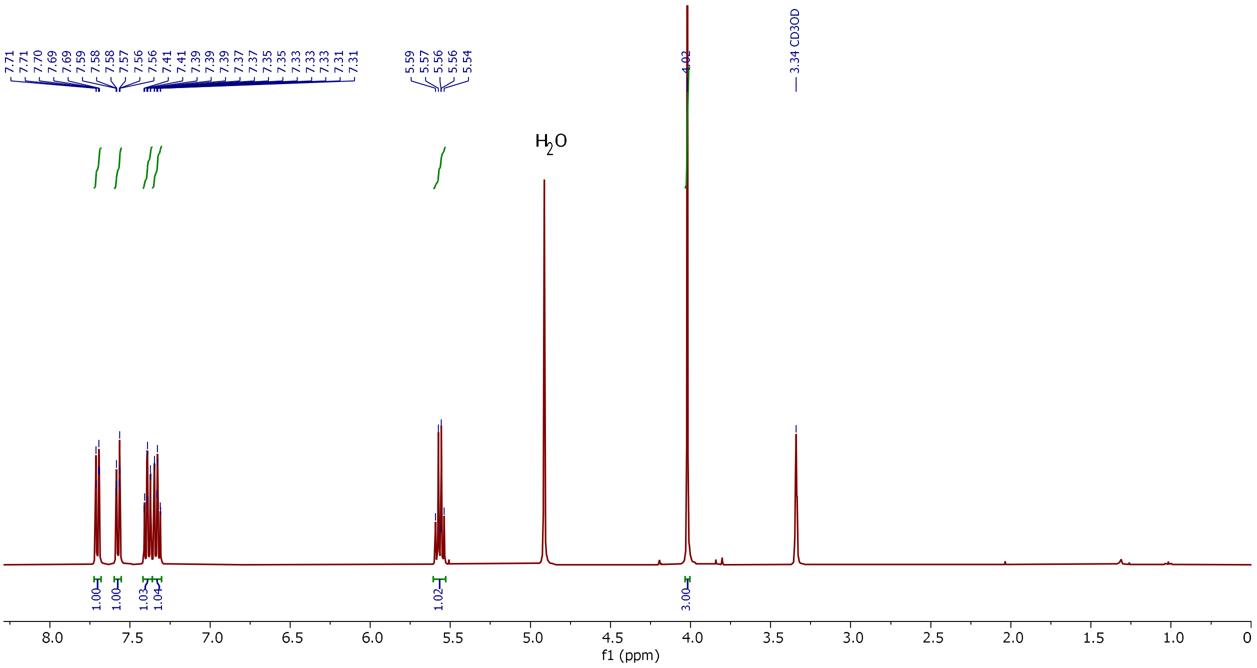

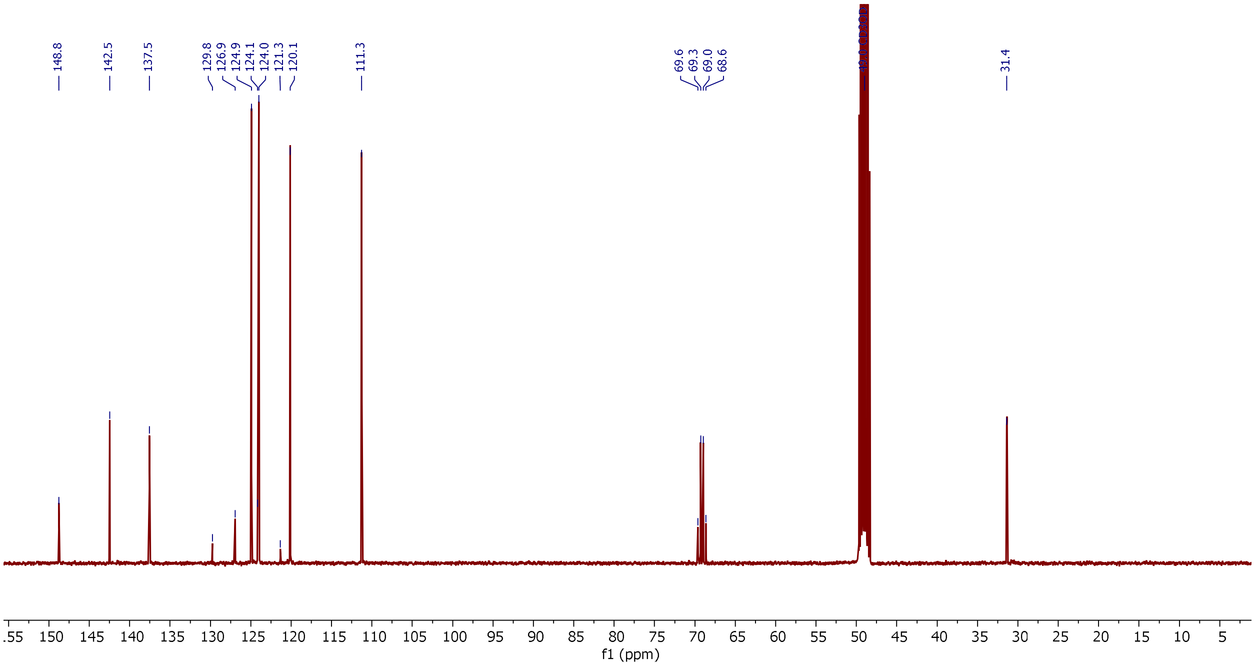

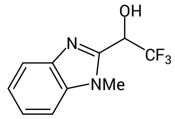

12-TFE

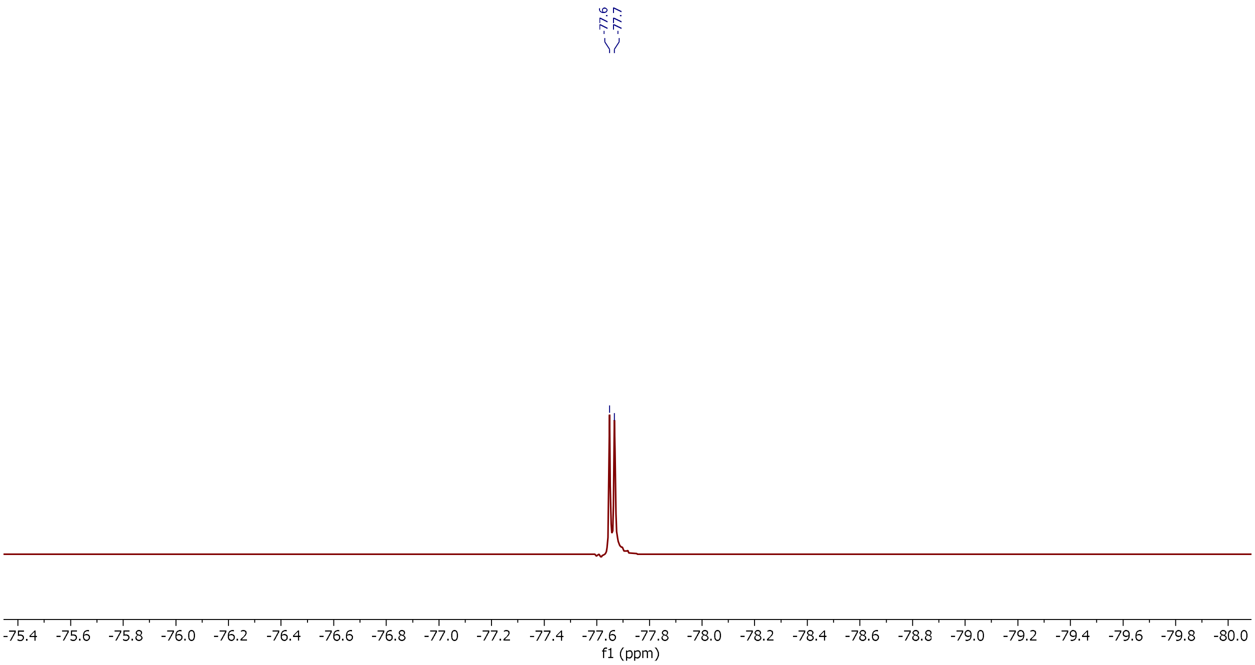
-77.6
-77.7
-75.4
-75.6
-75.8
-76.0
-76.2
-76.4
-76.6
-76.8
-77.0
-77.2
-77.4
-77.6
-77.8
-78.0
-78.2
-78.4
-78.6
-78.8
-79.0
-79.2
-79.4
-79.6
-79.8
-80.0
f1 (ppm)

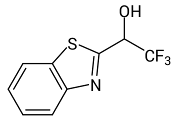

13-TFE

1-(benzo[*d*]thiazol-2-yl)-2,2,2-trifluoroethan-1-ol (13-TFE)

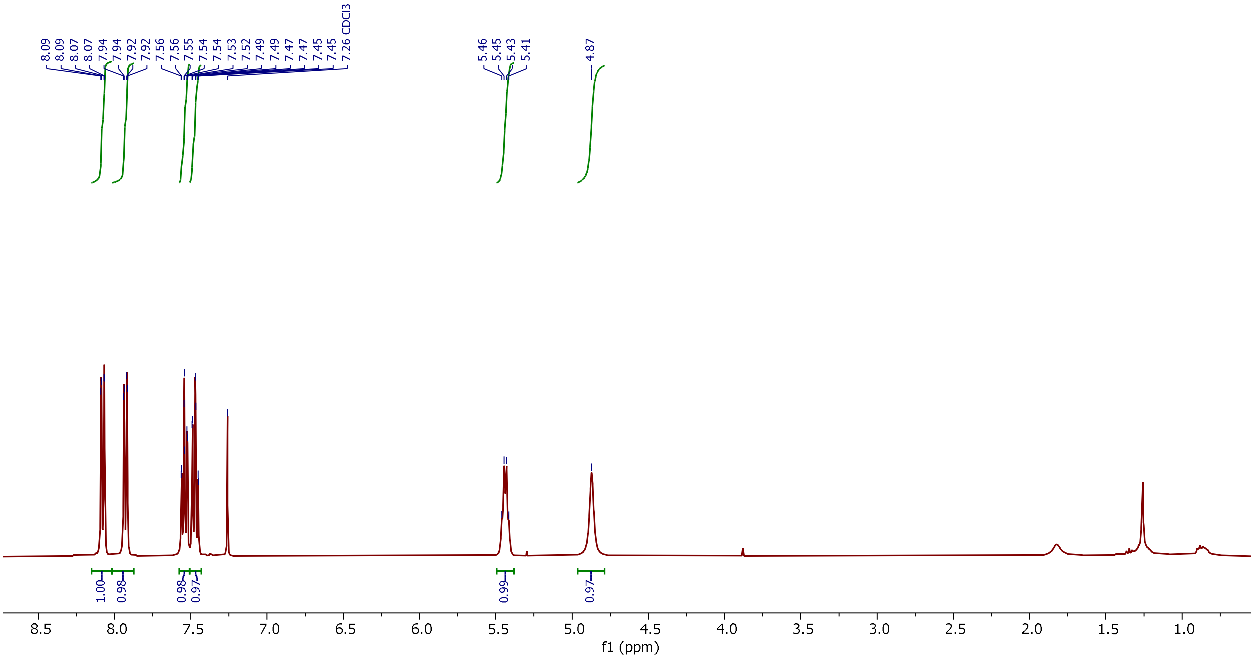

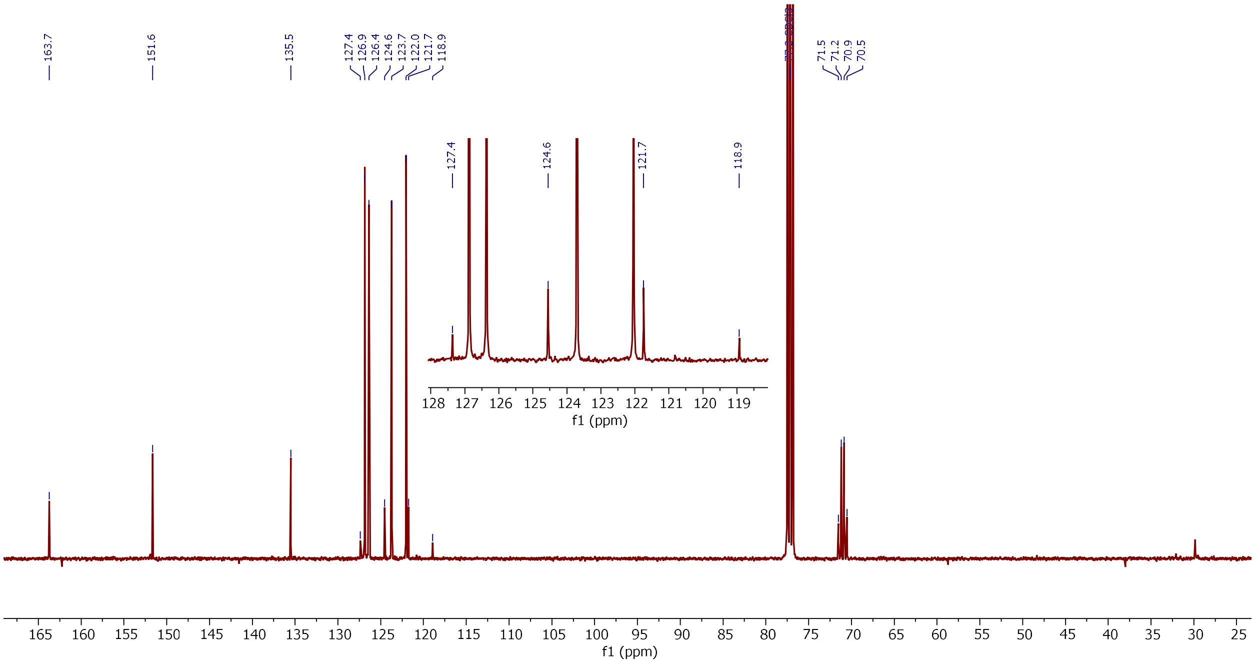

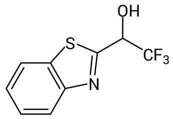

13-TFE

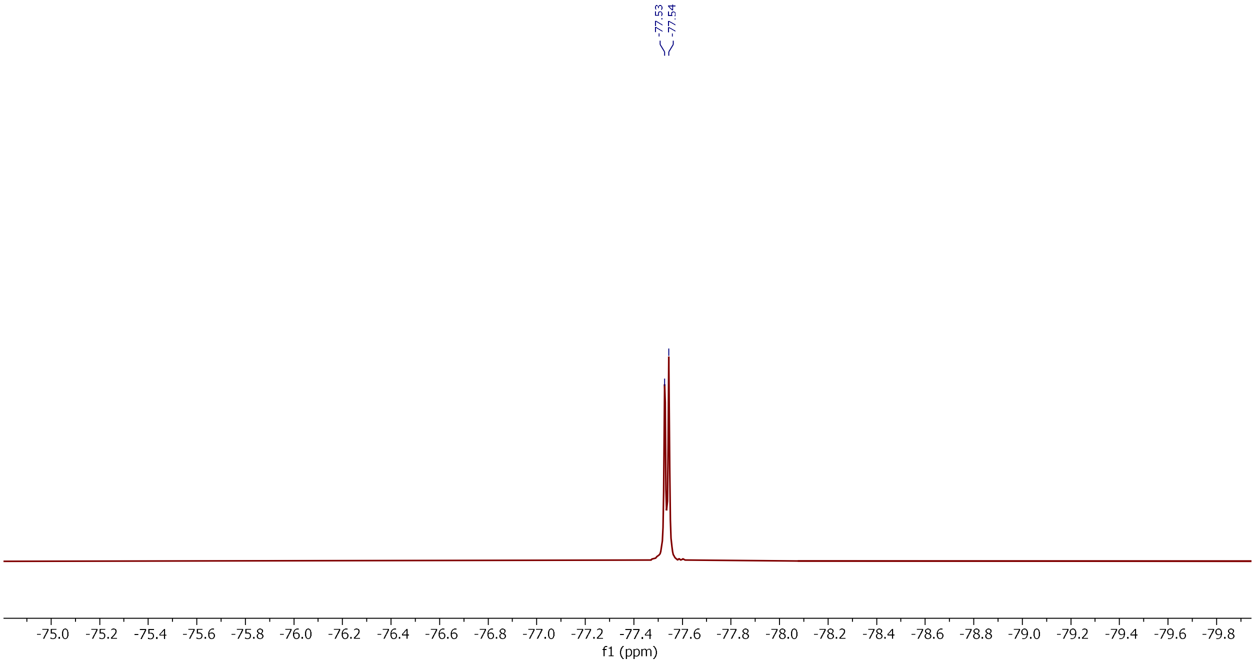

-77.53
-77.54
-75.0
-75.2
-75.4
-75.6
-75.8
-76.0
-76.2
-76.4
-76.6
-76.8
-77.0
-77.2
-77.4
-77.6
-77.8
-78.0
-78.2
-78.4
-78.6
-78.8
-79.0
-79.2
-79.4
-79.6
-79.8
f1 (ppm)

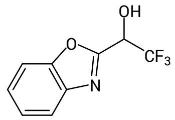

14-TFE

1-(benzo[*d*]oxazol-2-yl)-2,2,2-trifluoroethan-1-ol (14-TFE)

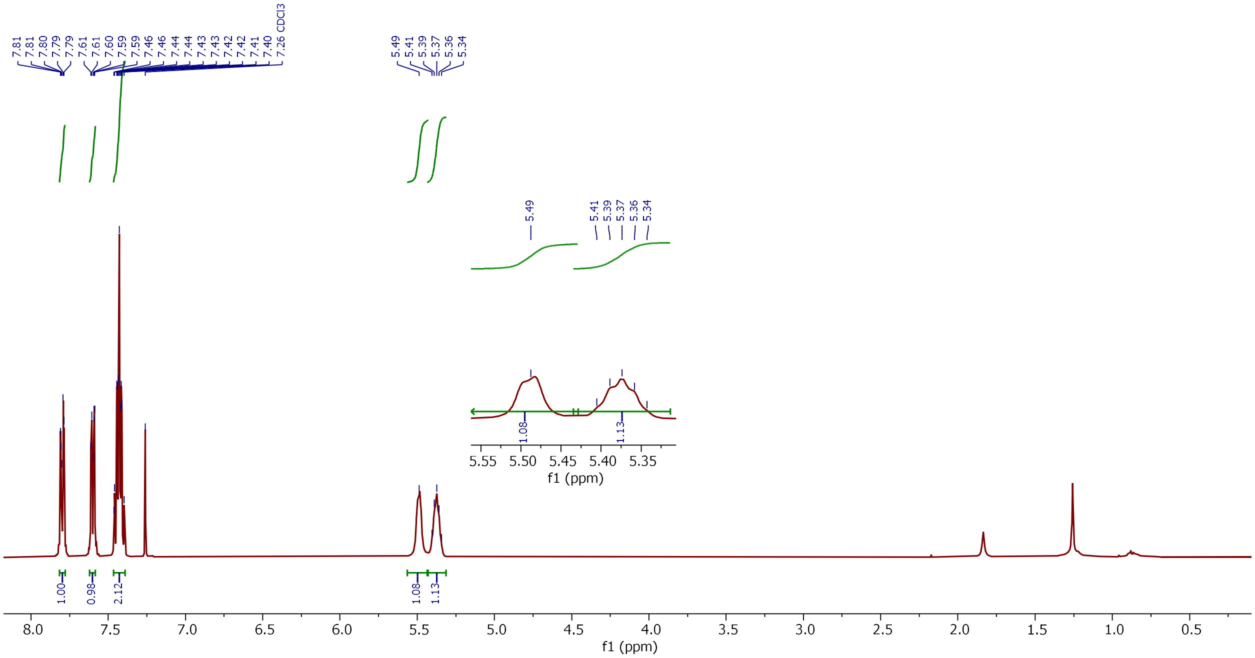

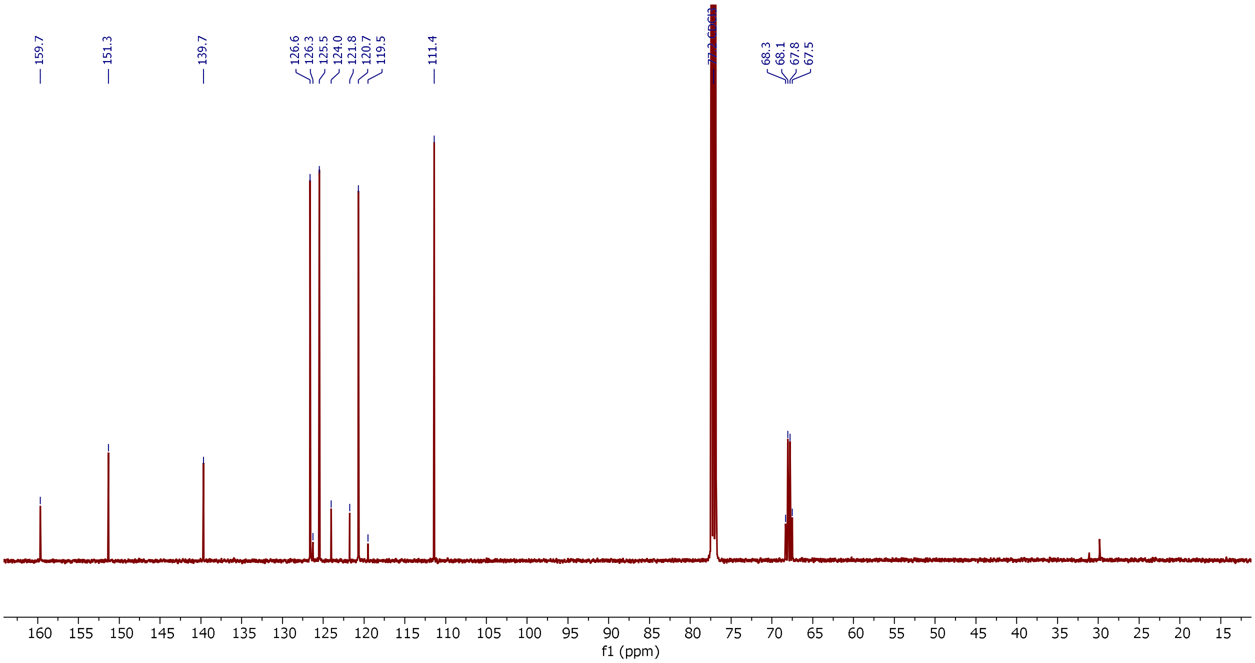

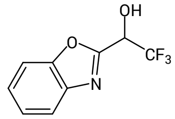

14-TFE

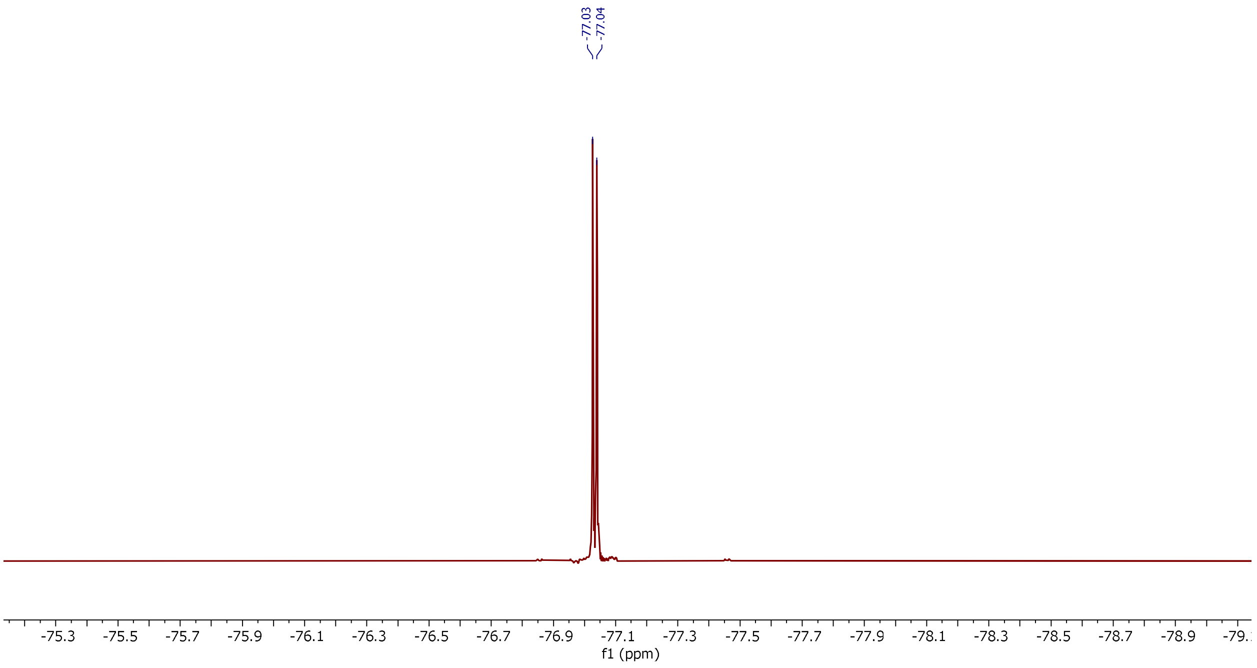

-77.03
-77.04
-75.3
-75.5
-75.7
-75.9
-76.1
-76.3
-76.5
-76.7
-76.9
-77.1
-77.3
-77.5
-77.7
-77.9
-78.1
-78.3
-78.5
-78.7
-78.9
f1 (ppm)

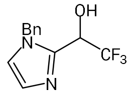

15-TFE

1-(1-benzyl-1*H*-imidazol-2-yl)-2,2,2-trifluoroethan-1-ol (15-TFE)

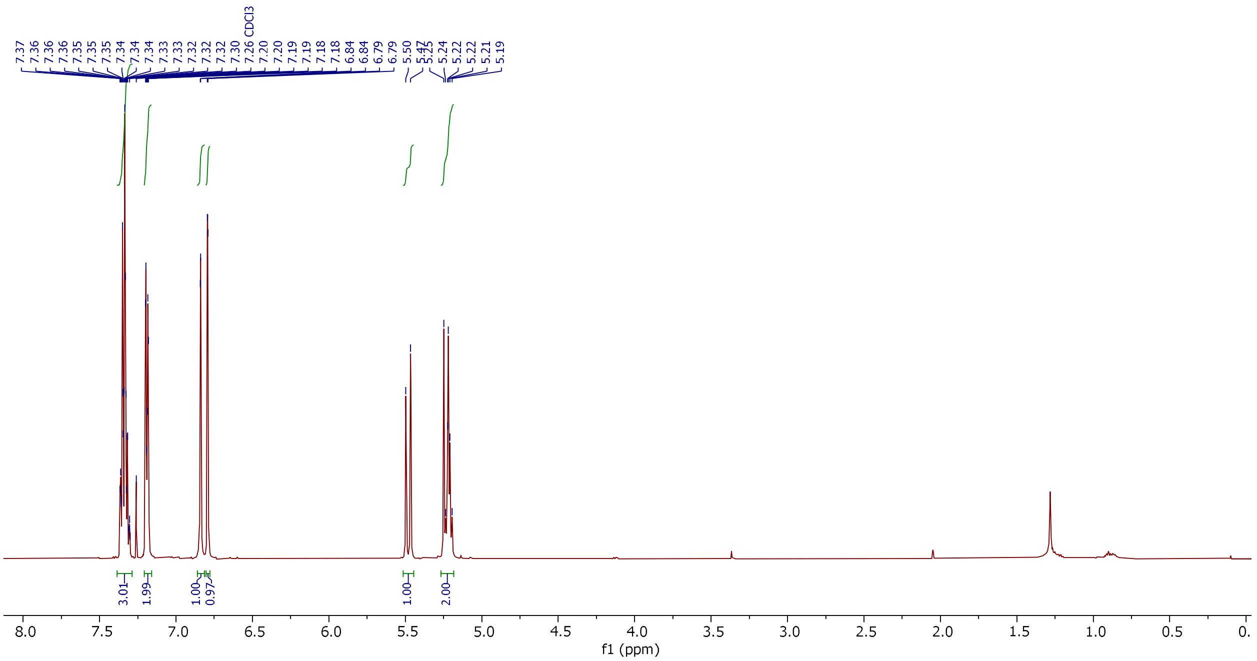

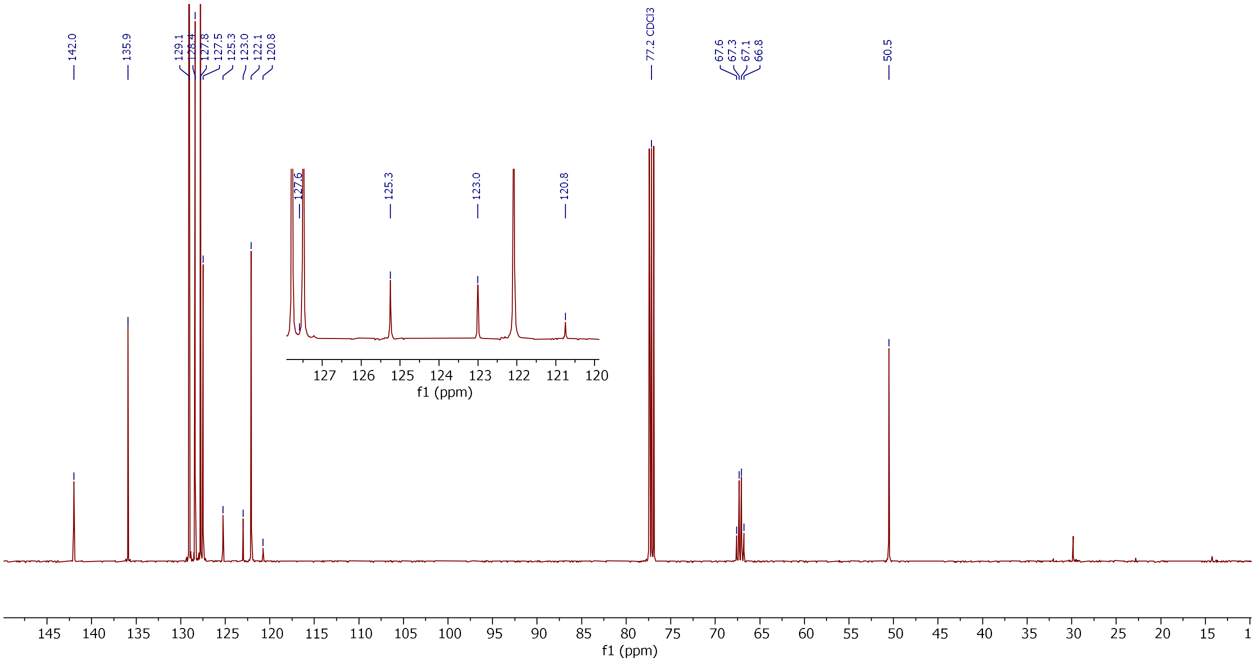

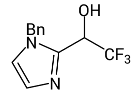
OH
Bn
N
N
CF3

15-TFE

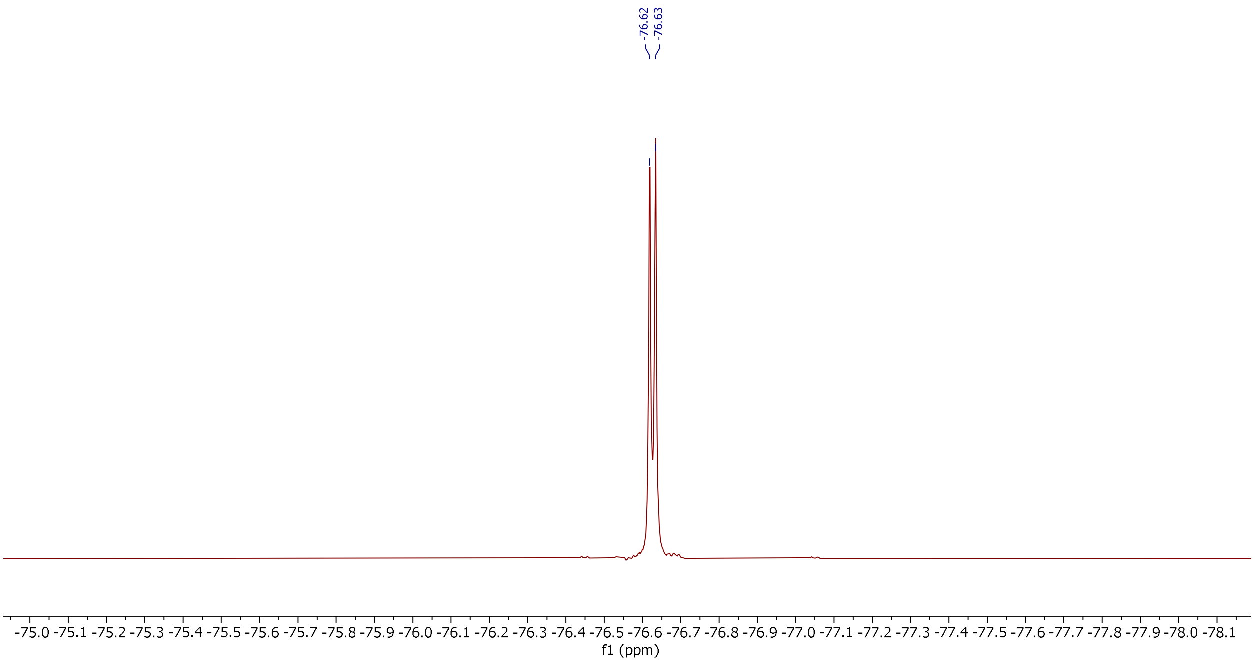
-76.62
-76.63
-75.0 -75.1 -75.2 -75.3 -75.4 -75.5 -75.6 -75.7 -75.8 -75.9 -76.0 -76.1 -76.2 -76.3 -76.4 -76.5 -76.6 -76.7 -76.8 -76.9 -77.0 -77.1 -77.2 -77.3 -77.4 -77.5 -77.6 -77.7 -77.8 -77.9 -78.0 -78.1
f1 (ppm)

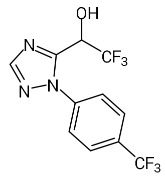

16-TFE

2,2,2-trifluoro-1-(4-(4-(trifluoromethyl)phenyl)-4*H*-1,2,4-triazol-3-yl)ethan-1-ol (16-TFE)

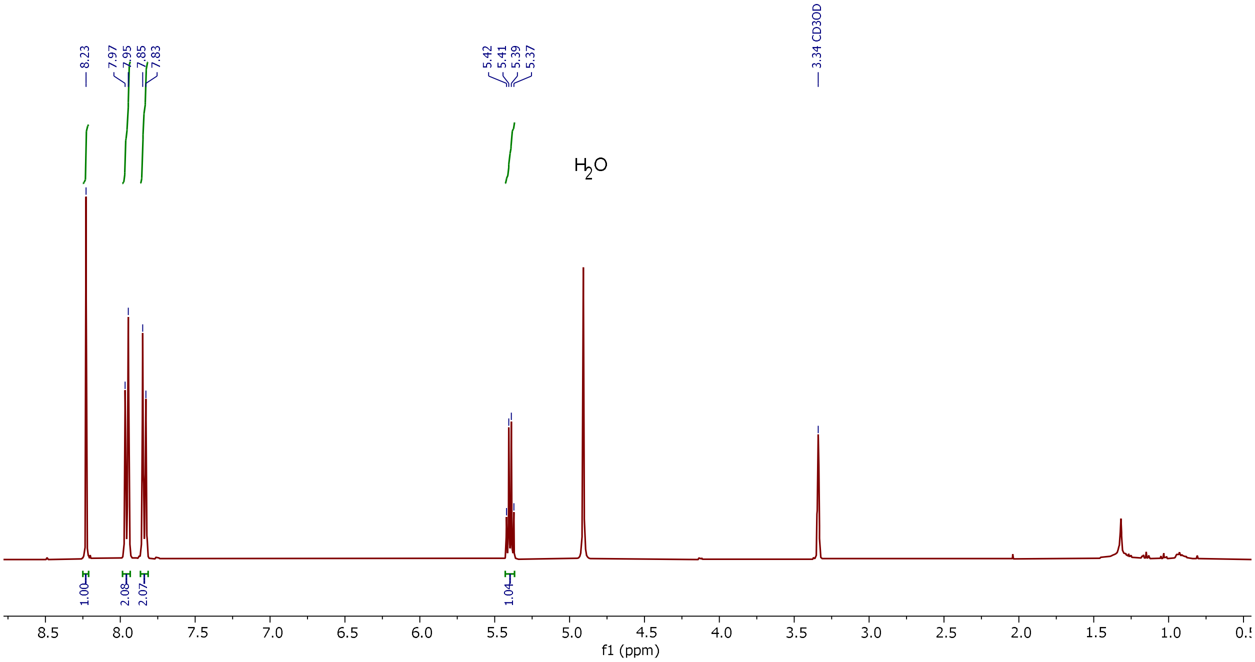

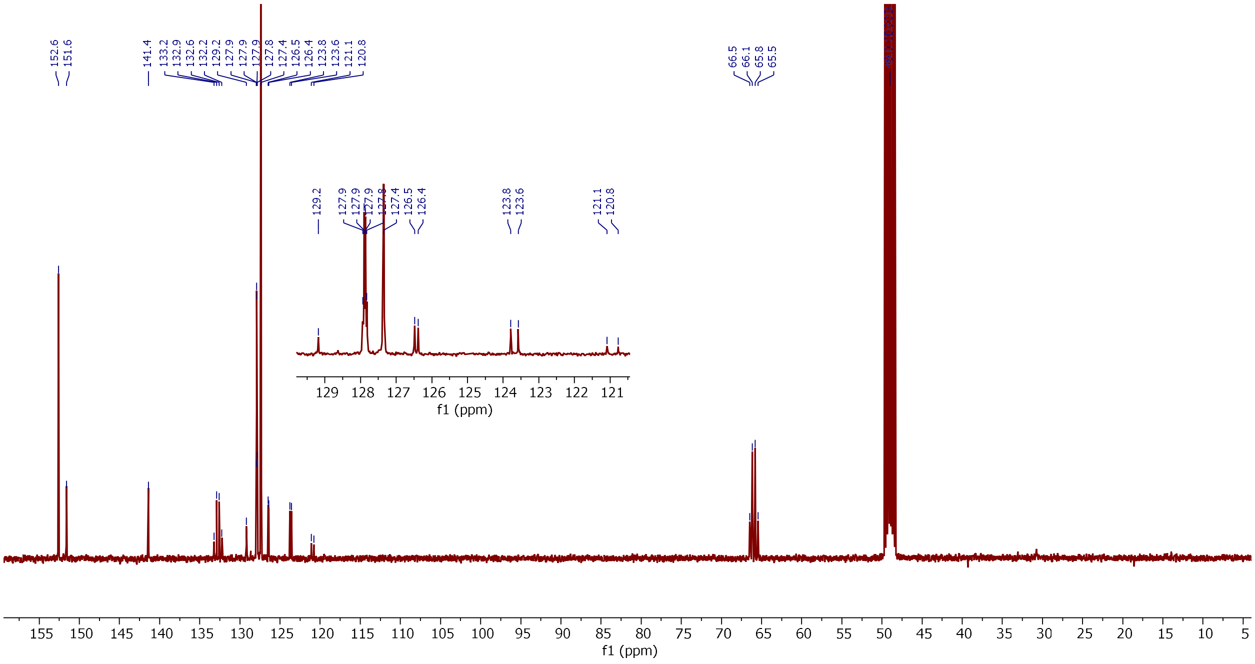

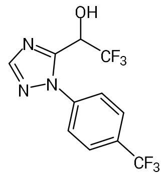

16-TFE

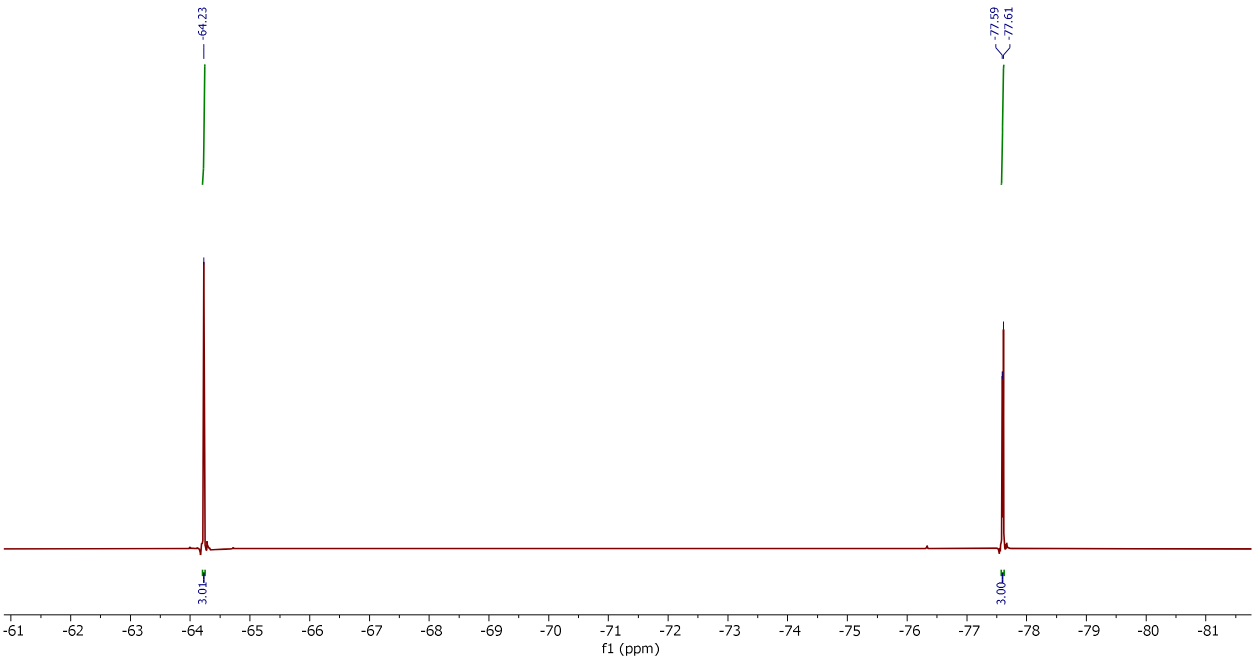

-64.23
-77.59
-77.61
3.01
3.00
-61 -62 -63 -64 -65 -66 -67 -68 -69 -70 -71 -72 -73 -74 -75 -76 -77 -78 -79 -80 -81
f1 (ppm)

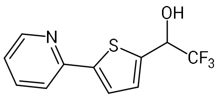

17-TFE

2,2,2-trifluoro-1-(5-(pyridin-2-yl)thiophen-2-yl)ethan-1-ol (17-TFE)

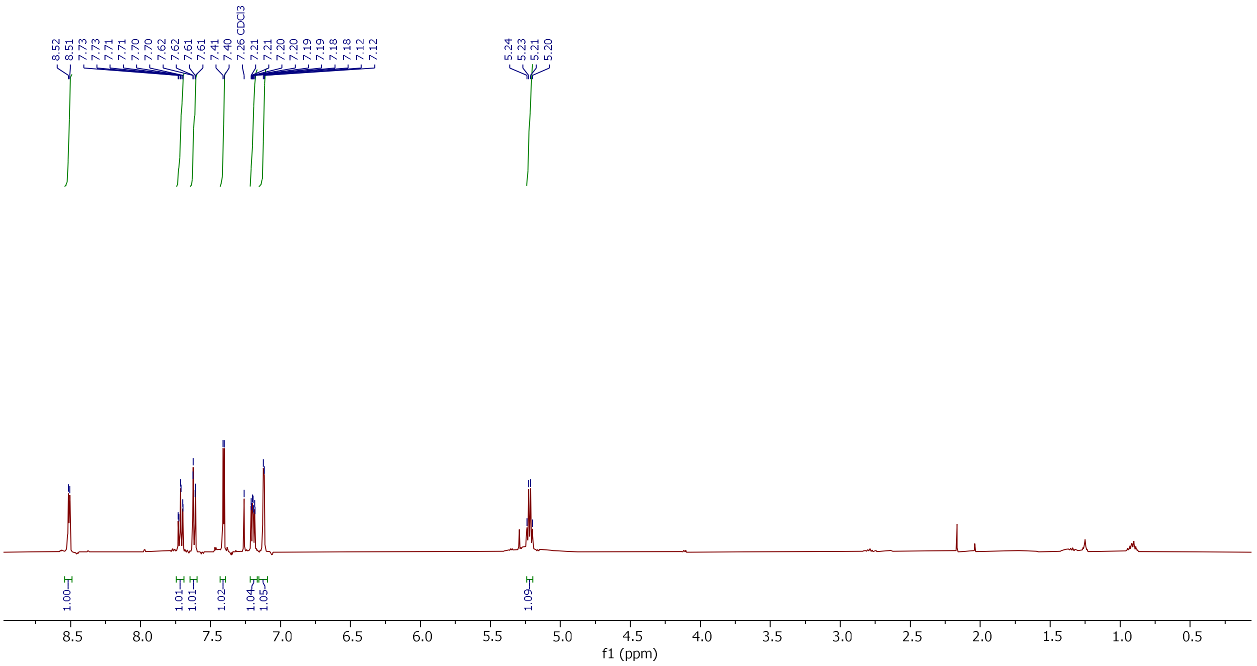

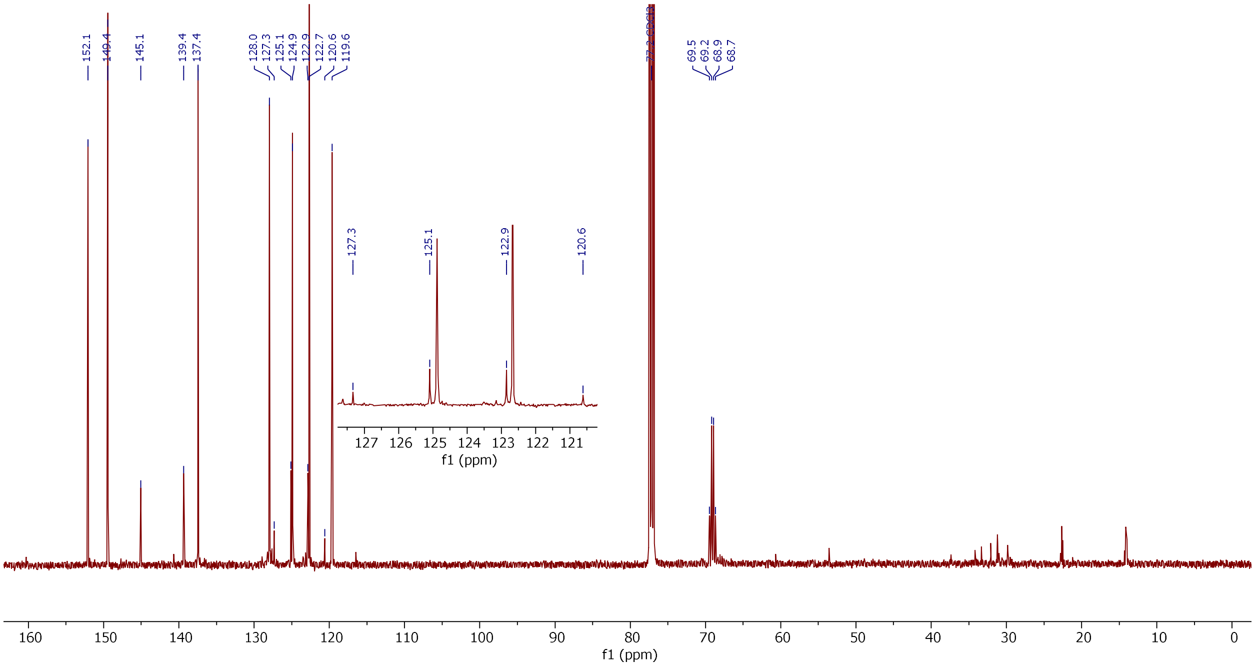

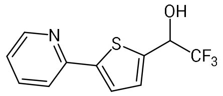

17-TFE

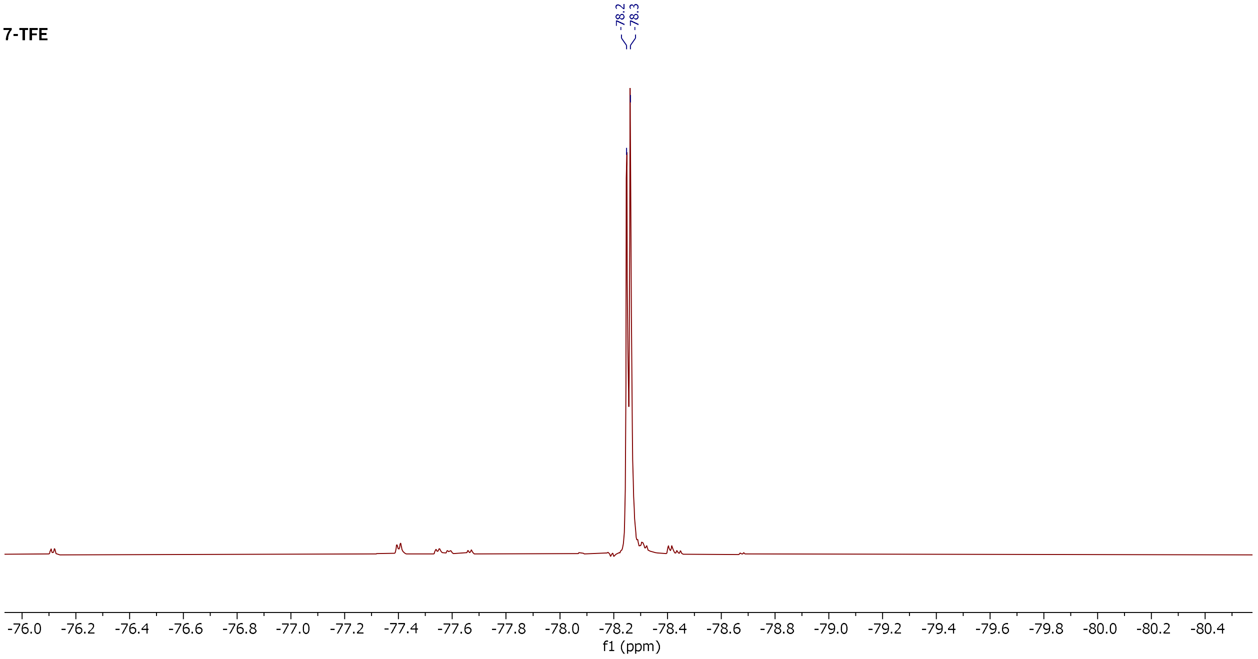

-78.2
-78.3
-76.0 -76.2 -76.4 -76.6 -76.8 -77.0 -77.2 -77.4 -77.6 -77.8 -78.0 -78.2 -78.4 -78.6 -78.8 -79.0 -79.2 -79.4 -79.6 -79.8 -80.0 -80.2 -80.4
f1 (ppm)

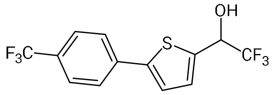

18-TFE

2,2,2-trifluoro-1-(5-(4-(trifluoromethyl)phenyl)thiophen-2-yl)ethan-1-ol (18-TFE)

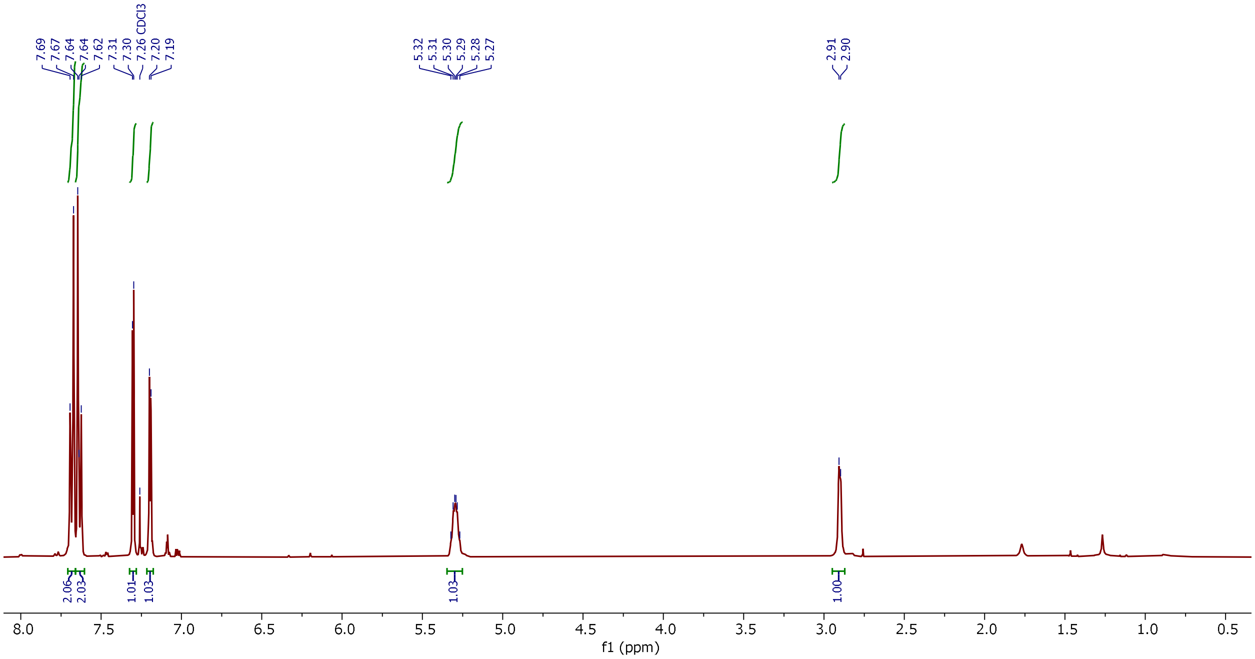

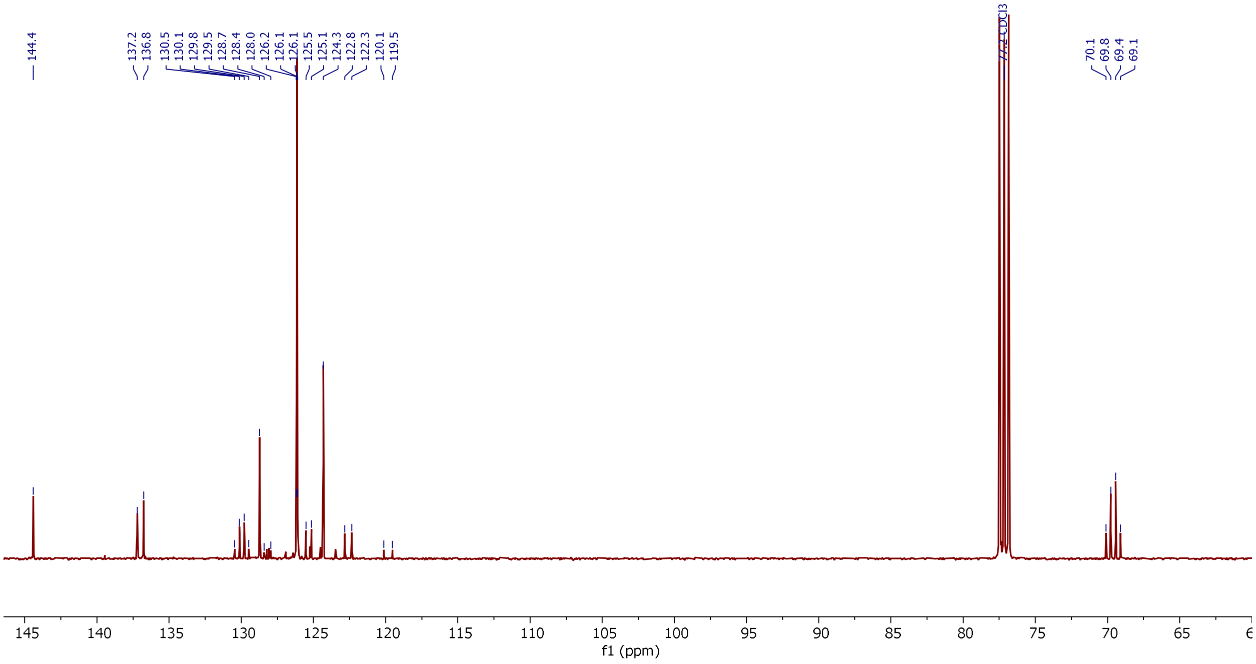

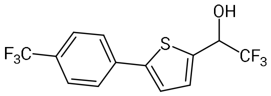

18-TFE

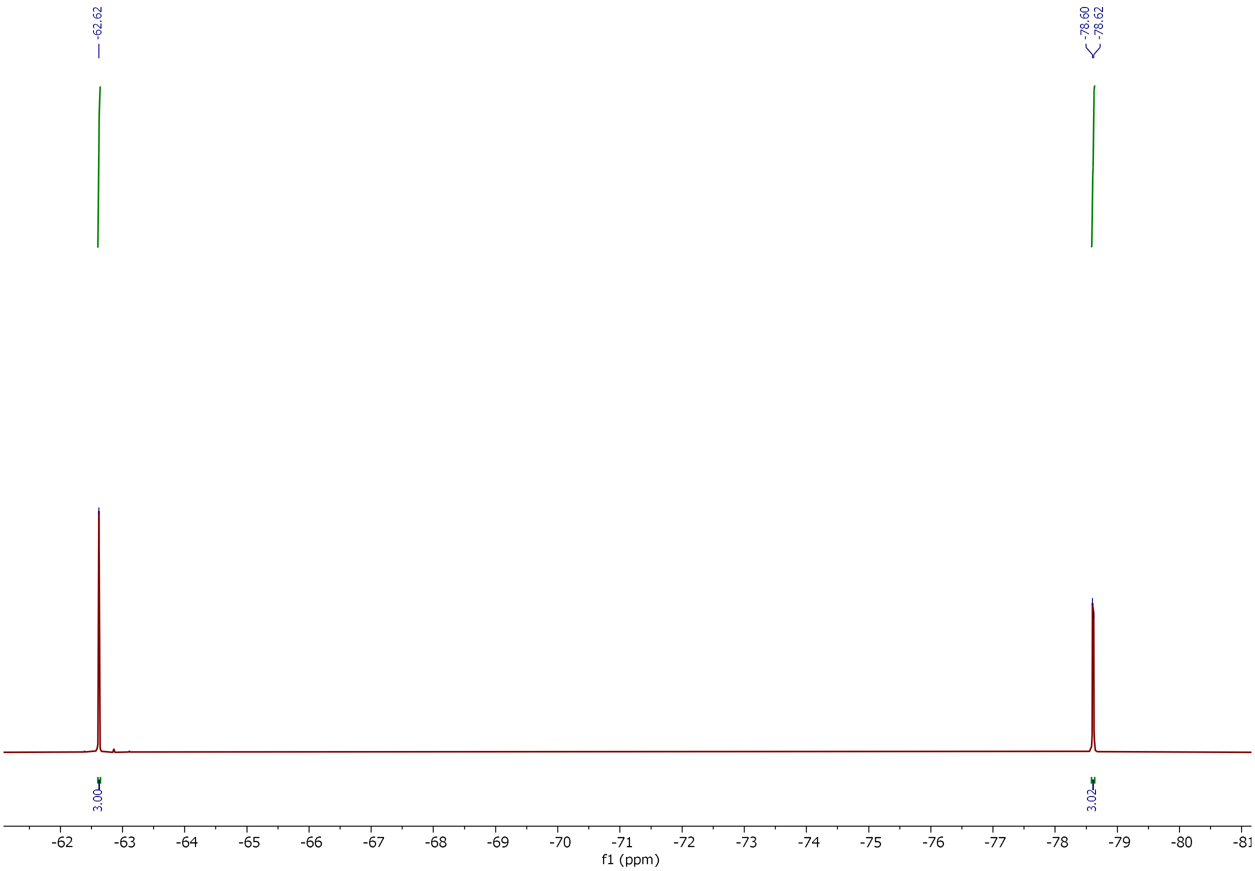
-62.62
-78.60
-78.62
3.00
3.02
-62
-63
-64
-65
-66
-67
-68
-69
-70
-71
-72
-73
-74
-75
-76
-77
-78
-79
-80
-81
f1 (ppm)

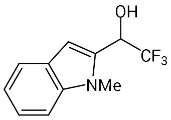

19-TFE

2,2,2-trifluoro-1-(1-methyl-1*H*-indol-2-yl)ethan-1-ol (19-TFE)

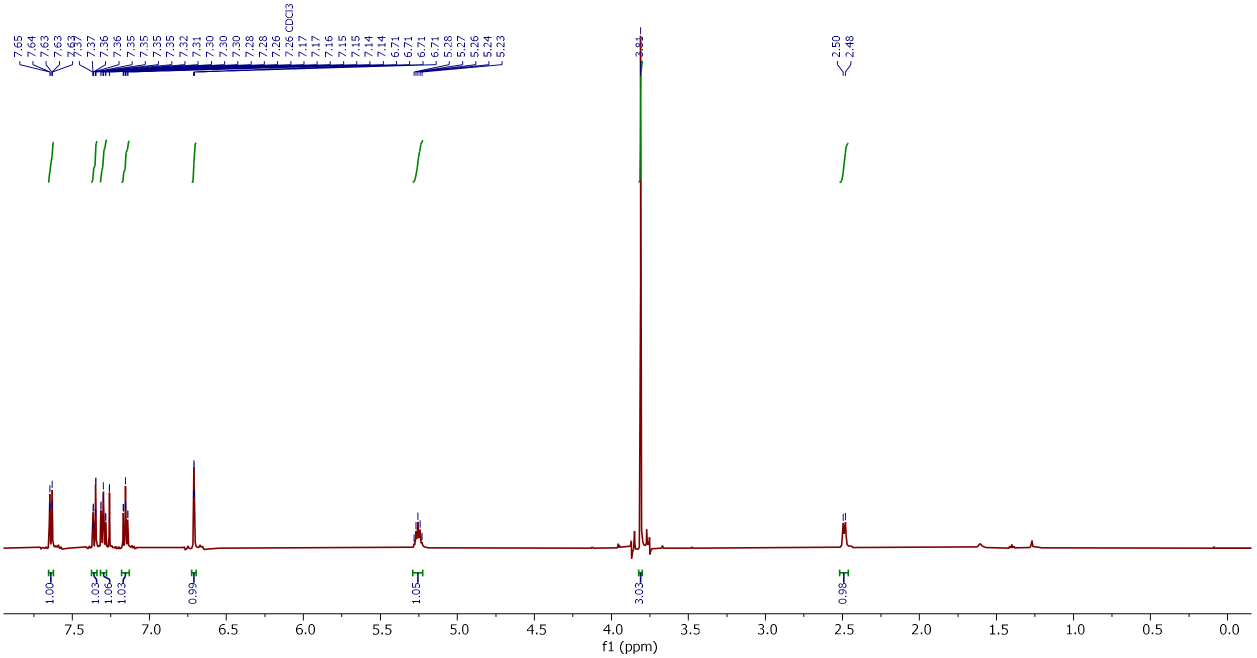

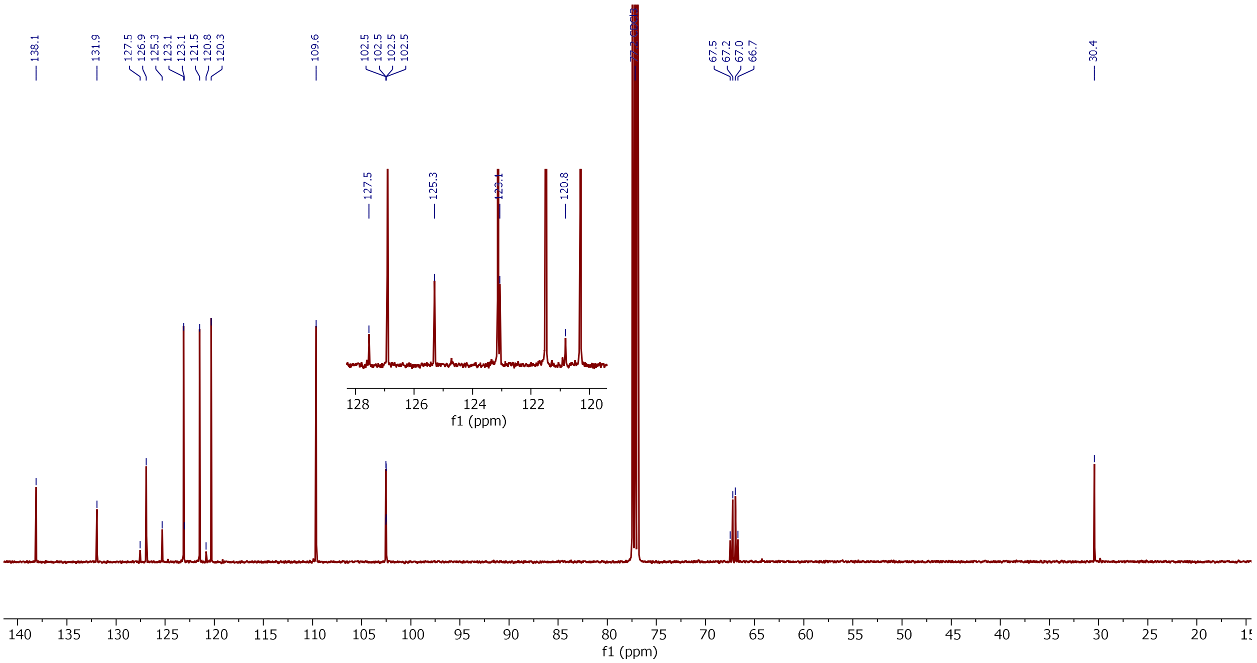

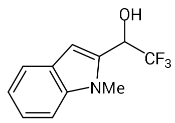
OH
CF3
NMe

19-TFE

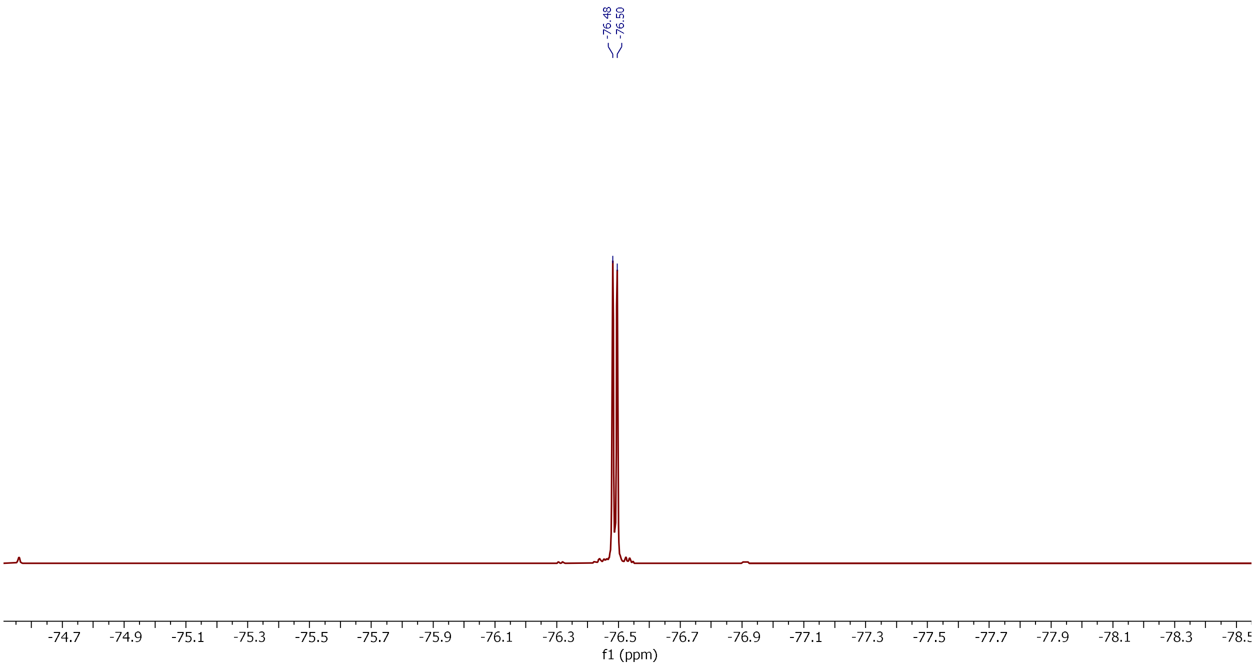
-76.48
-76.50
-74.7
-74.9
-75.1
-75.3
-75.5
-75.7
-75.9
-76.1
-76.3
-76.5
-76.7
-76.9
-77.1
-77.3
-77.5
-77.7
-77.9
-78.1
-78.3
f1 (ppm)

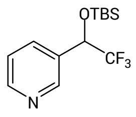

1-TBS

3-(1-((*tert*-butyldimethylsilyl)oxy)-2,2,2-trifluoroethyl)pyridine (1-TBS)

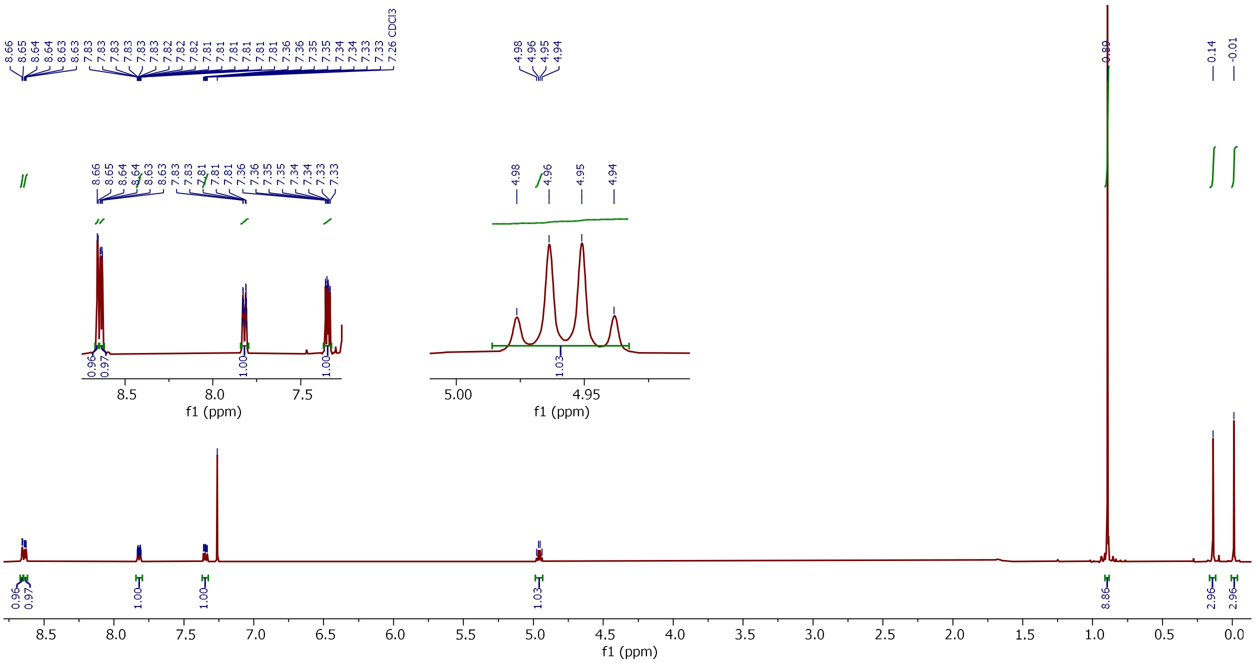

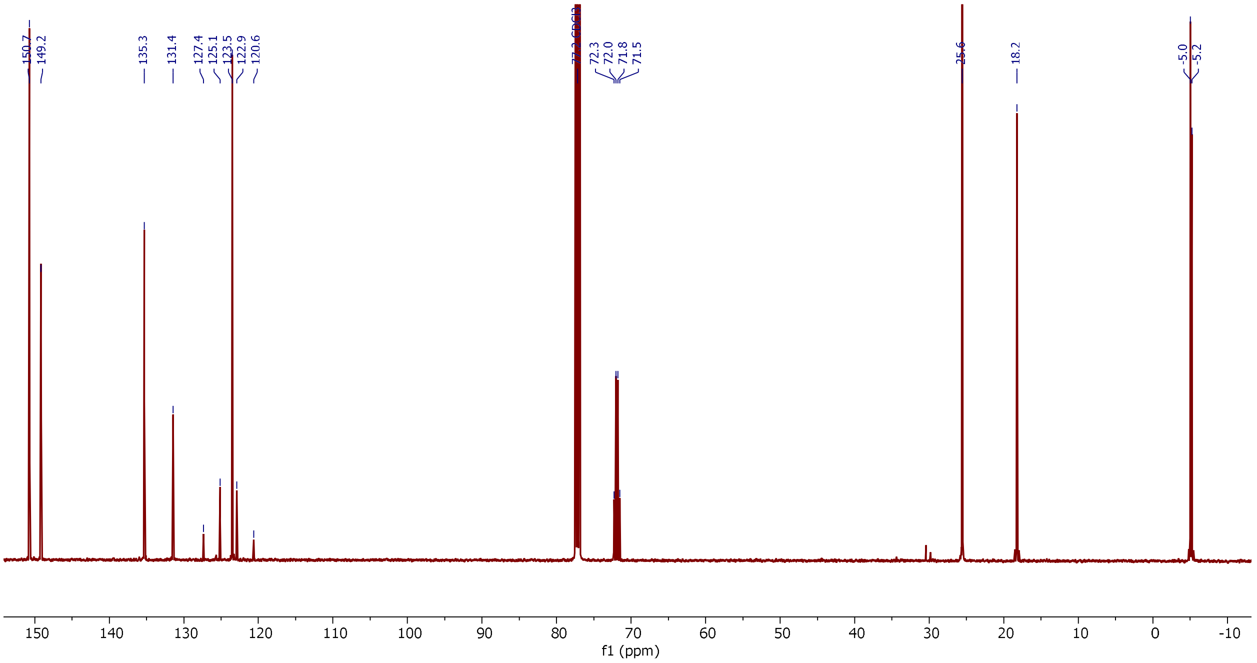

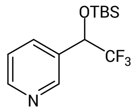
OTBS
CF3
N

1-TBS

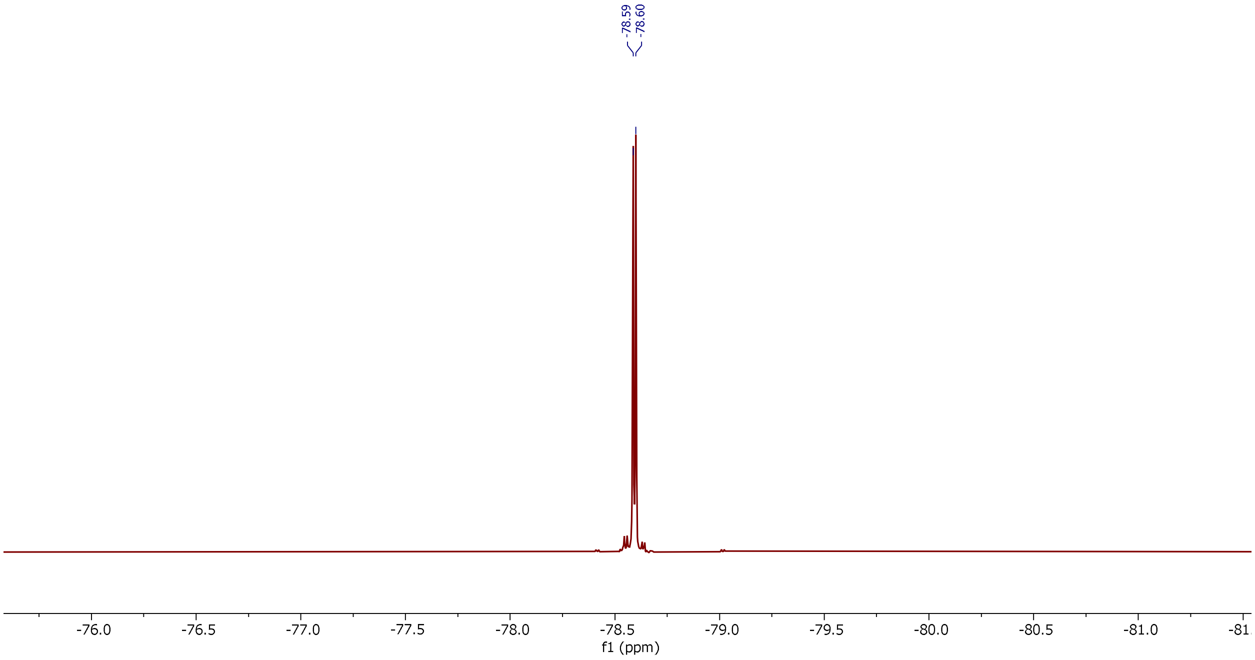
-78.59
-78.60
-76.0
-76.5
-77.0
-77.5
-78.0
-78.5
-79.0
-79.5
-80.0
-80.5
-81.0
f1 (ppm)

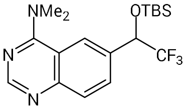

7-TBS

6-(1-((*tert*-butyldimethylsilyl)oxy)-2,2,2-trifluoroethyl)-*N*,*N*-dimethylquinazolin-4-amine (7-TBS)

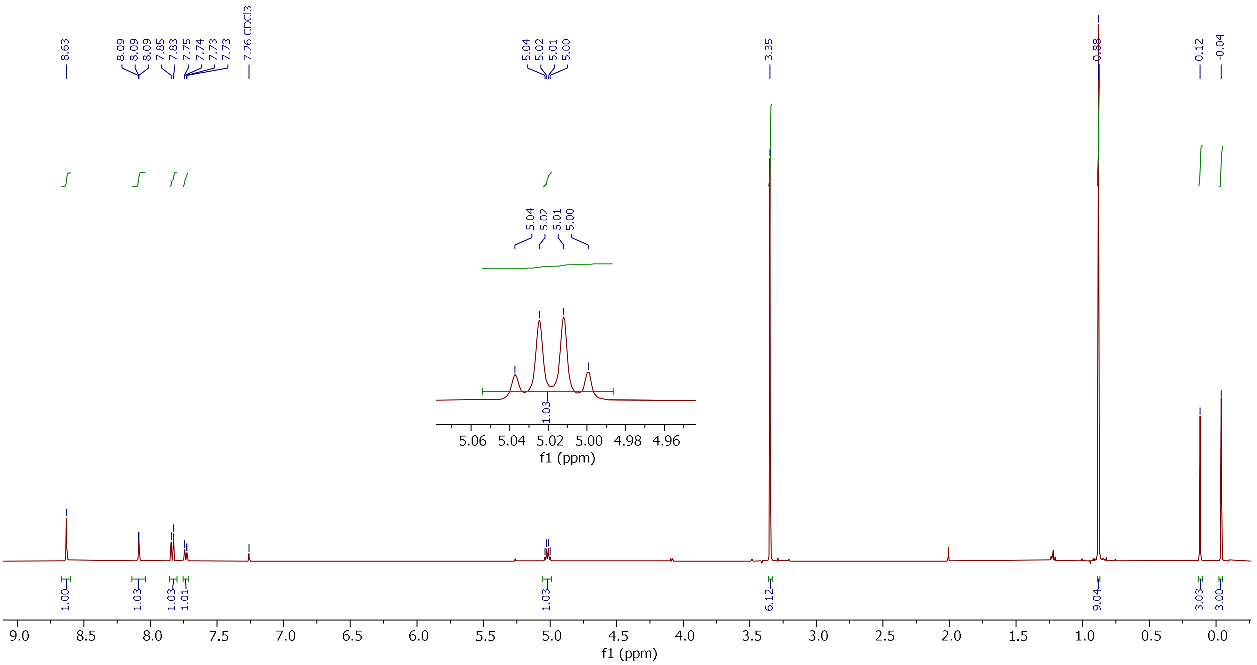

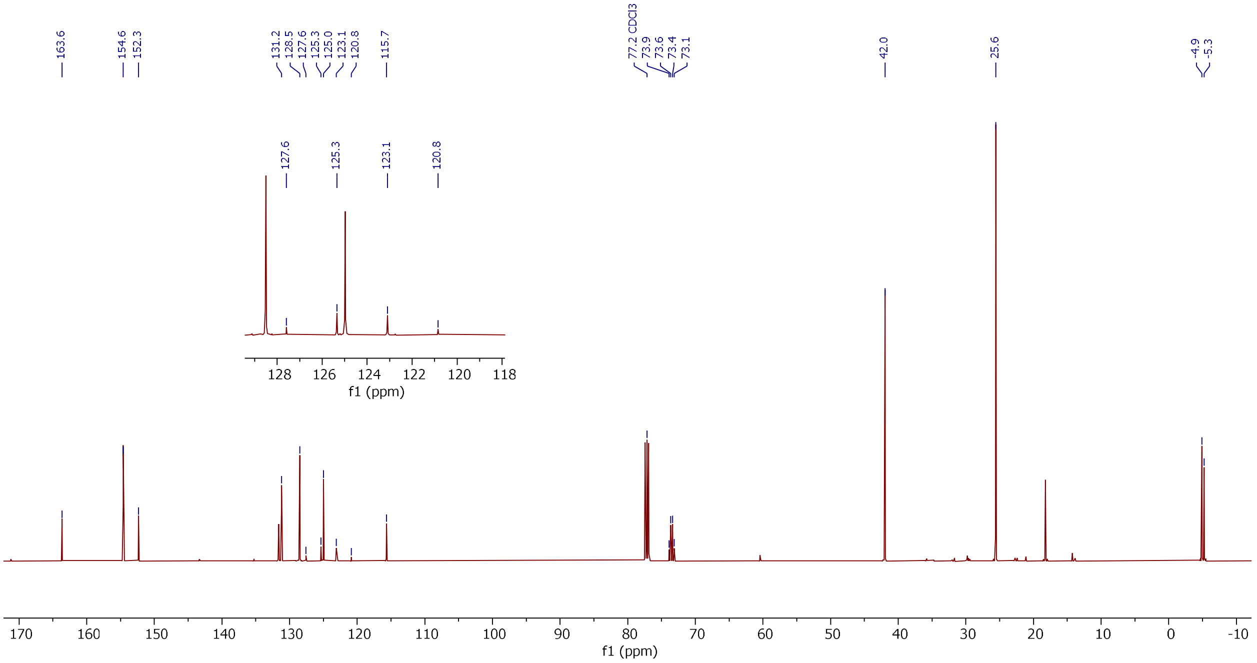

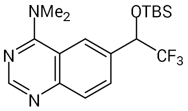
NMe2
OTBS
N
N
CF3

7-TBS

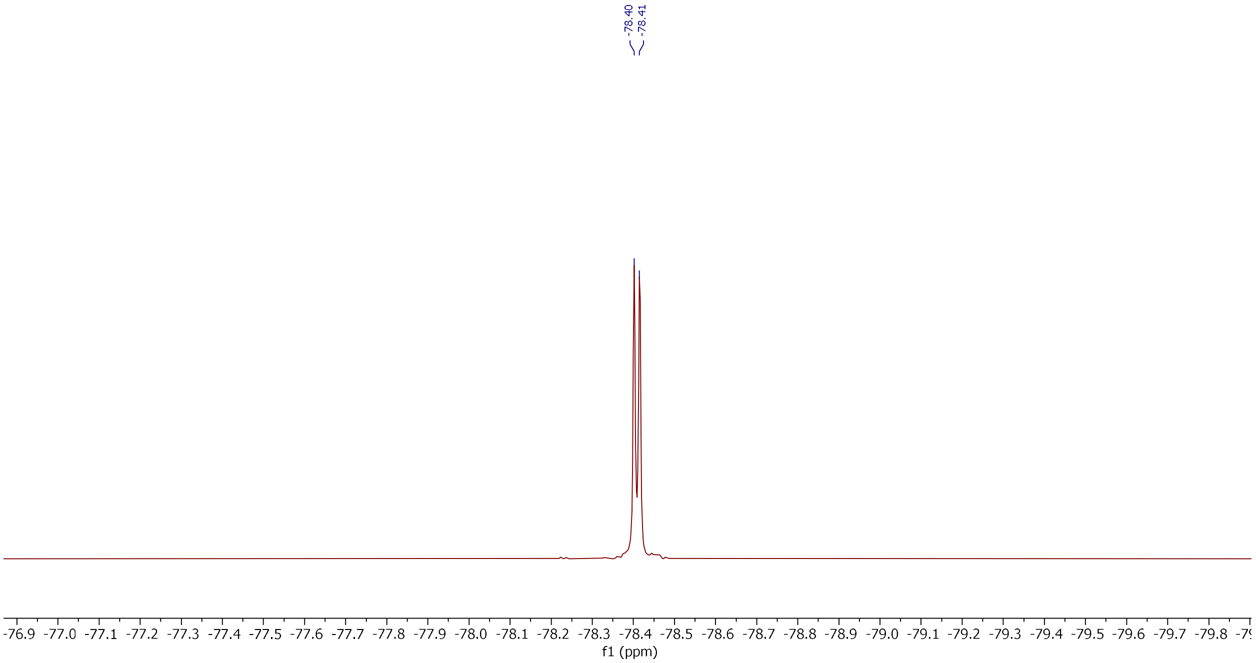
-78.40
-78.41
-76.9 -77.0 -77.1 -77.2 -77.3 -77.4 -77.5 -77.6 -77.7 -77.8 -77.9 -78.0 -78.1 -78.2 -78.3 -78.4 -78.5 -78.6 -78.7 -78.8 -78.9 -79.0 -79.1 -79.2 -79.3 -79.4 -79.5 -79.6 -79.7 -79.8
f1 (ppm)

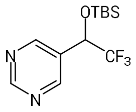

8-TBS

5-(1-((*tert*-butyldimethylsilyl)oxy)-2,2,2-trifluoroethyl)pyrimidine (8-TBS)

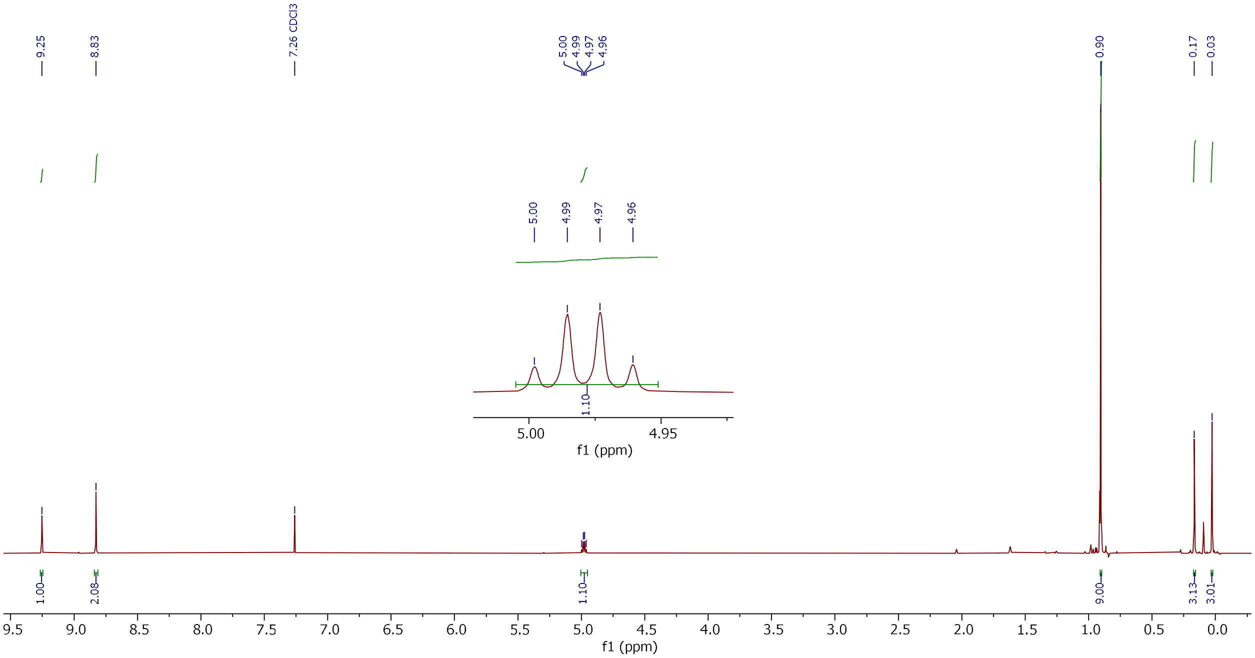

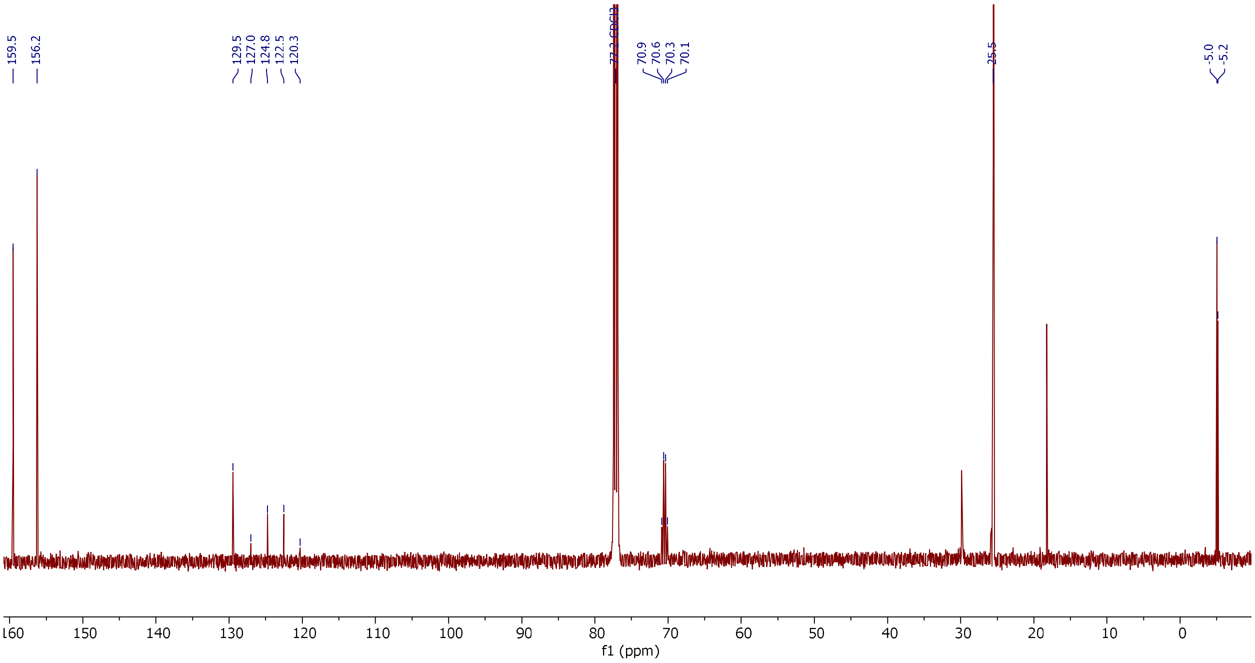

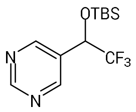
OTBS
N
N
CF3

8-TBS

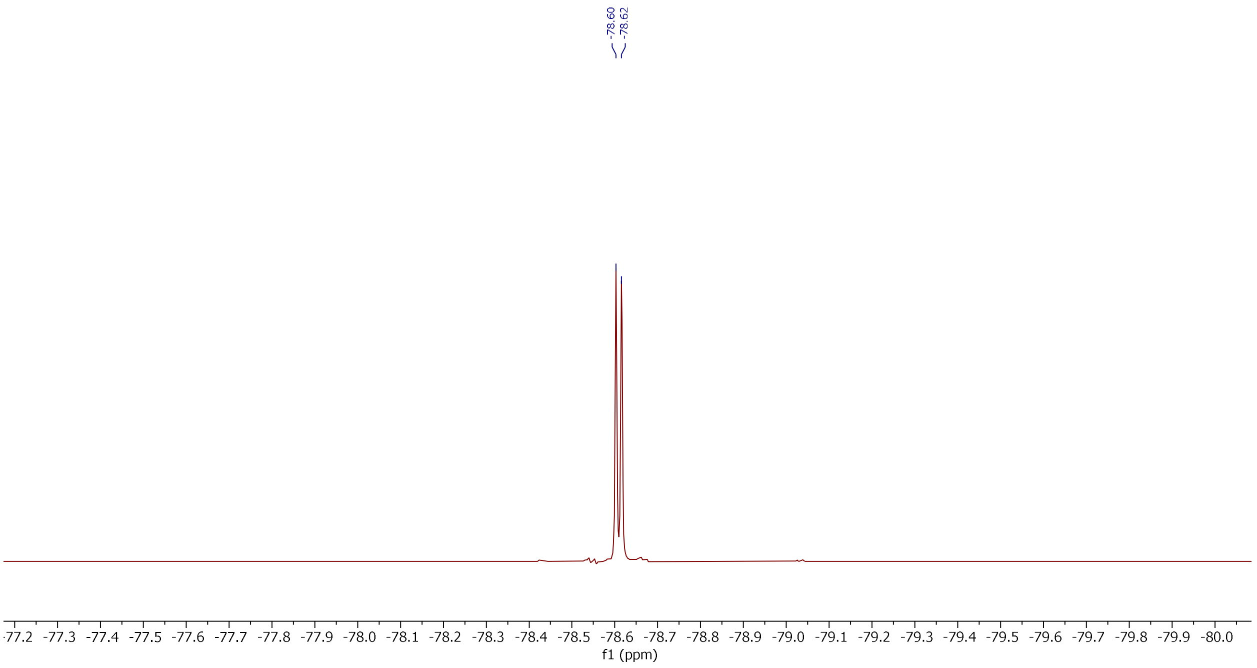
-78.60
-78.62
-77.2 -77.3 -77.4 -77.5 -77.6 -77.7 -77.8 -77.9 -78.0 -78.1 -78.2 -78.3 -78.4 -78.5 -78.6 -78.7 -78.8 -78.9 -79.0 -79.1 -79.2 -79.3 -79.4 -79.5 -79.6 -79.7 -79.8 -79.9 -80.0
f1 (ppm)

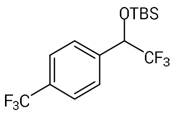

9-TBS

tert-butyldimethyl(2,2,2-trifluoro-1-(4-(trifluoromethyl)phenyl)ethoxy)silane (9-TBS)

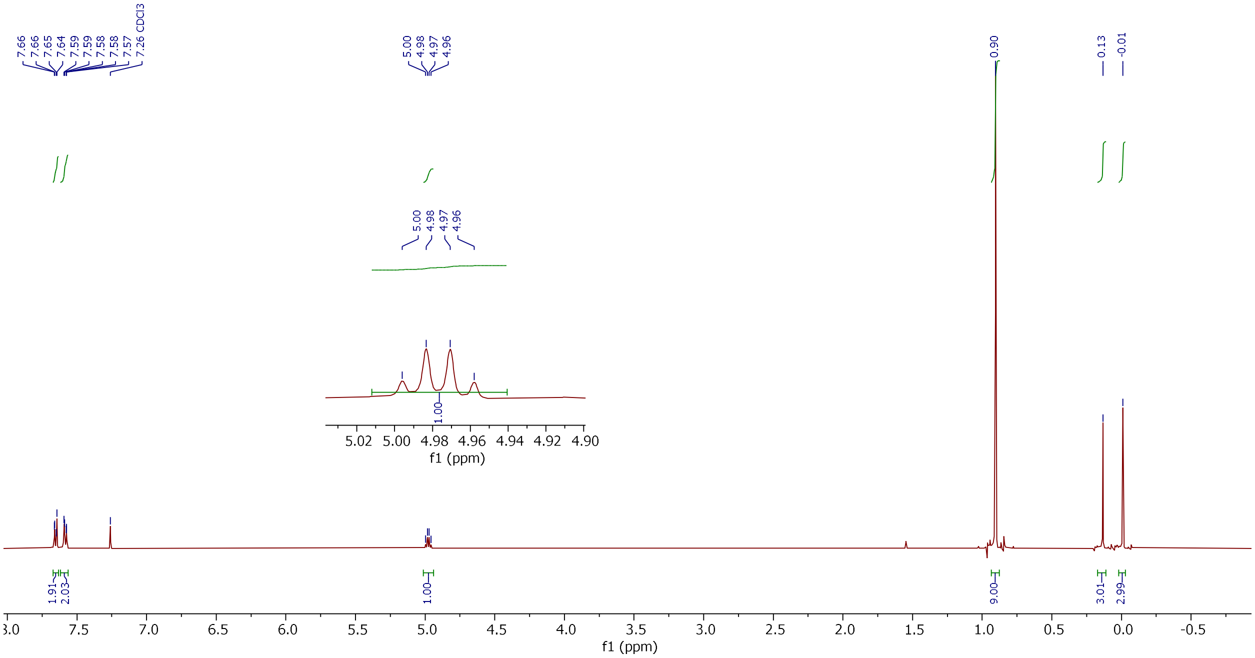

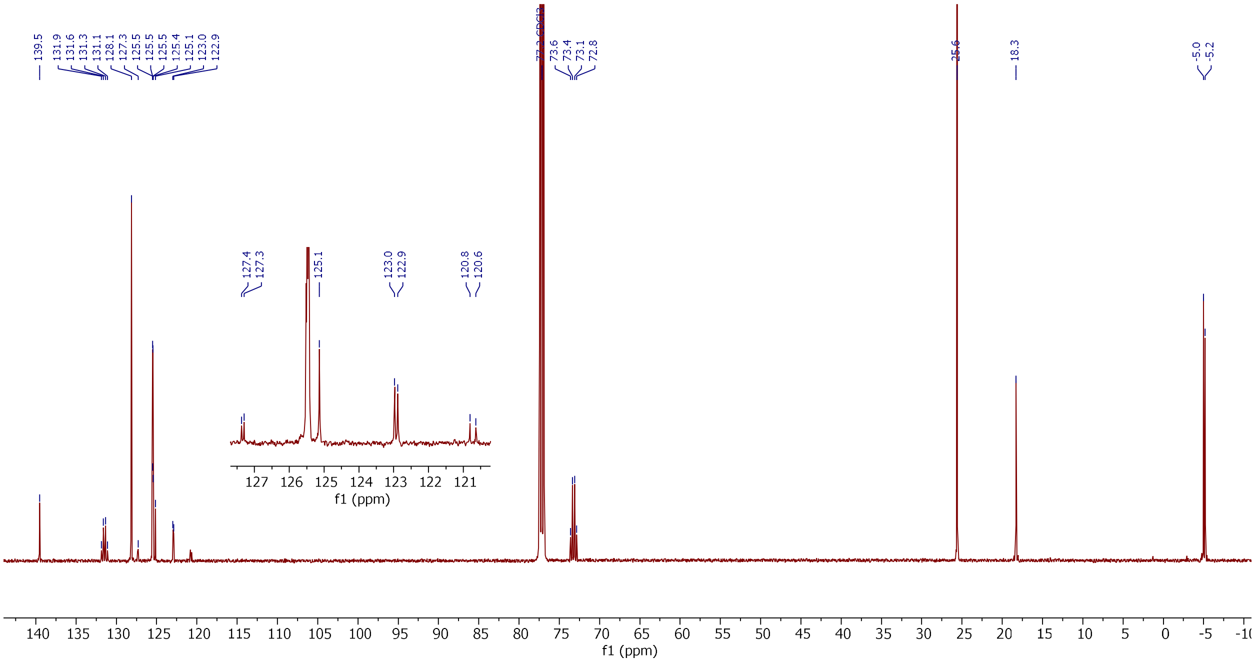

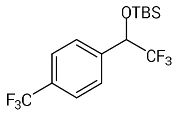

9-TBS

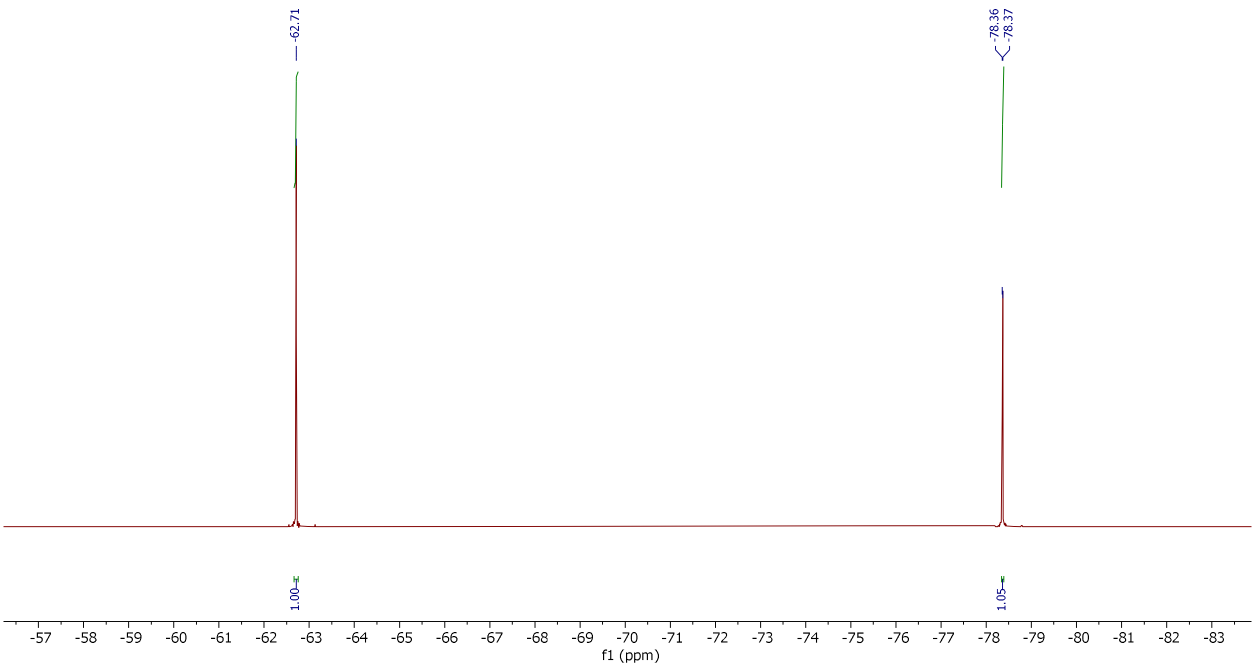

-62.71
-78.36
-78.37
1.00
1.05
-57 -58 -59 -60 -61 -62 -63 -64 -65 -66 -67 -68 -69 -70 -71 -72 -73 -74 -75 -76 -77 -78 -79 -80 -81 -82 -83
f1 (ppm)

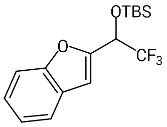

11-TBS

(1-(benzofuran-2-yl)-2,2,2-trifluoroethoxy)(*tert*-butyl)dimethylsilane (11-TBS)

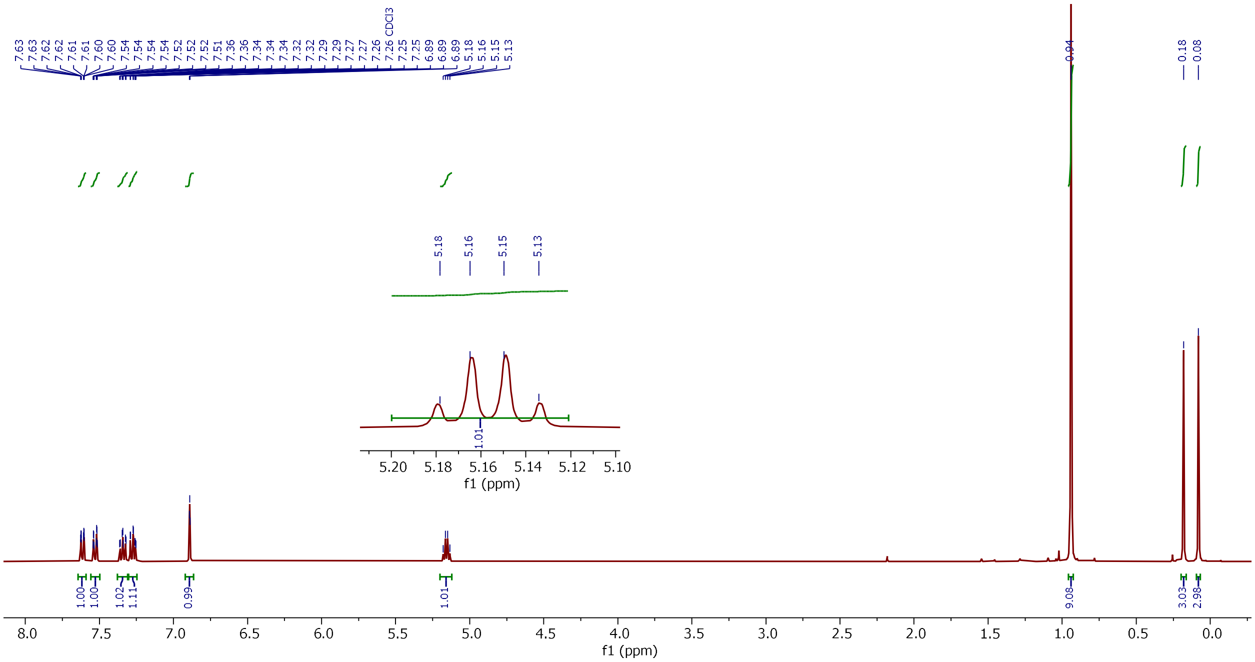

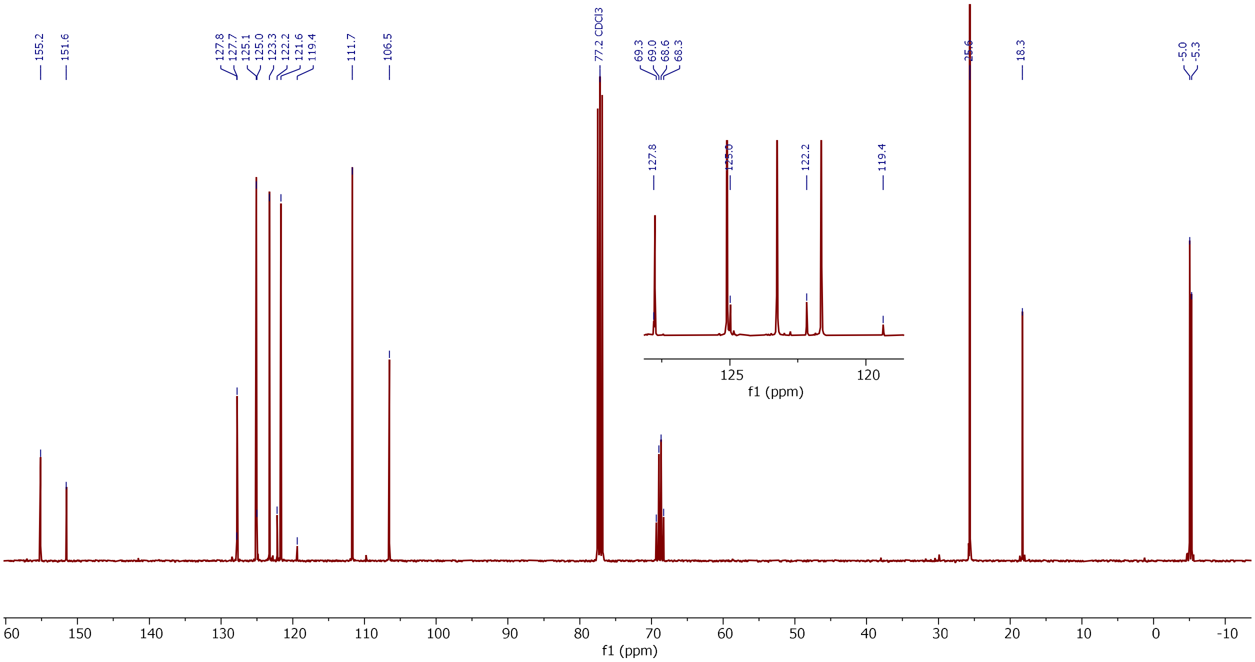

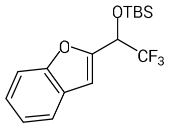

11-TBS

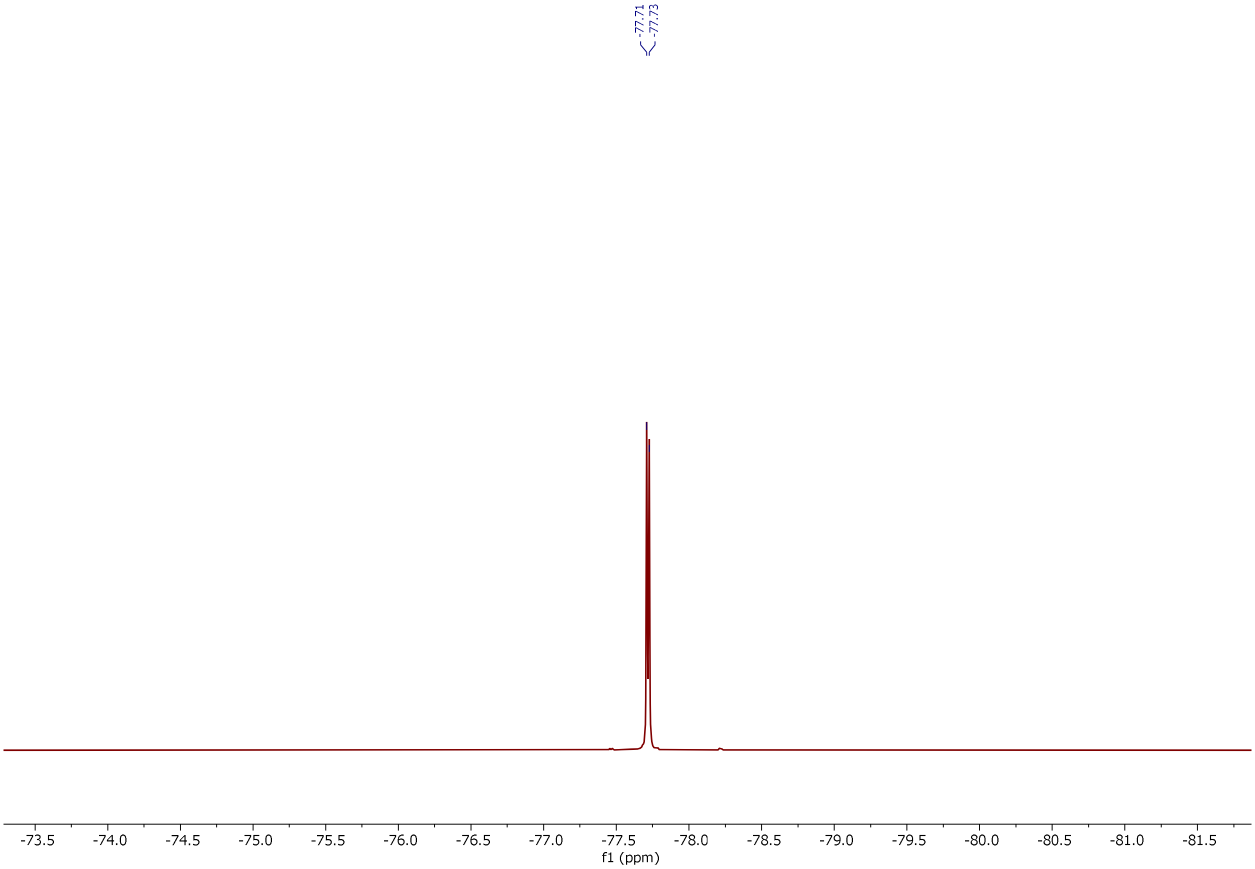
-77.71
-77.73
-73.5
-74.0
-74.5
-75.0
-75.5
-76.0
-76.5
-77.0
-77.5
-78.0
-78.5
-79.0
-79.5
-80.0
-80.5
-81.0
-81.5
f1 (ppm)

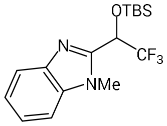

12-TBS

2-(1-((*tert*-butyldimethylsilyl)oxy)-2,2,2-trifluoroethyl)-1-methyl-1*H*-benzo[*d*]imidazole (12-TBS)

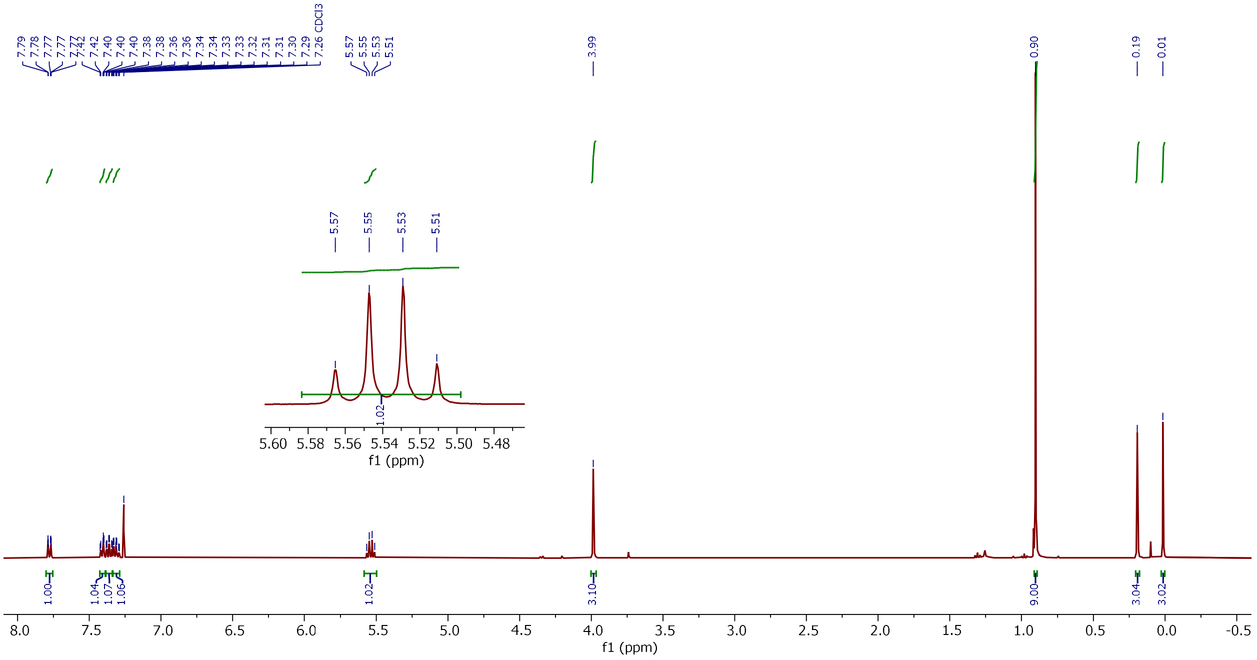

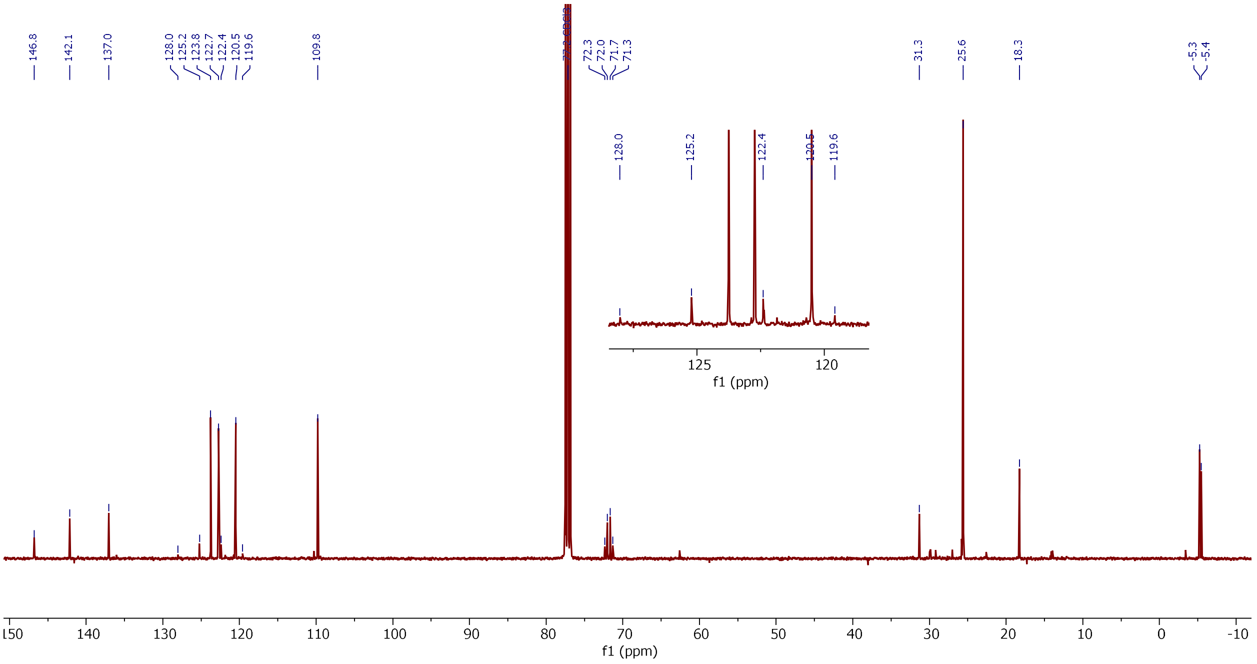

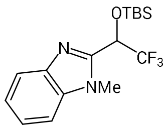
OTBS
N
CF3
NMe

12-TBS

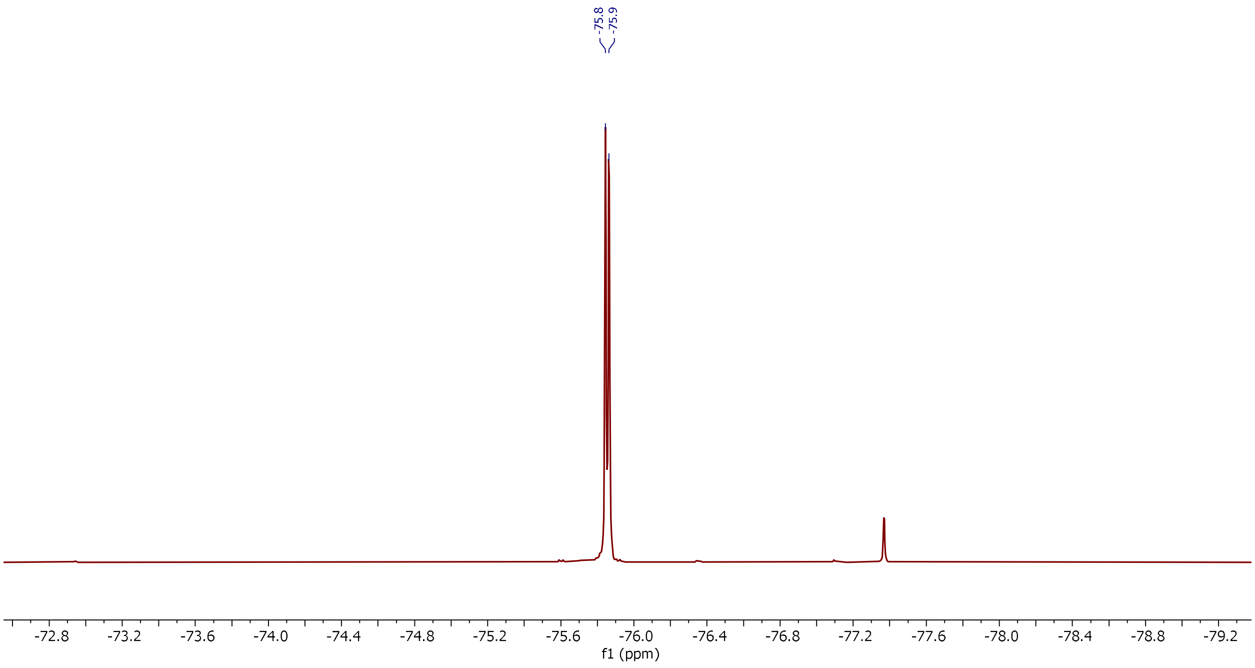
-75.8
-75.9
-72.8
-73.2
-73.6
-74.0
-74.4
-74.8
-75.2
-75.6
-76.0
-76.4
-76.8
-77.2
-77.6
-78.0
-78.4
-78.8
-79.2
f1 (ppm)

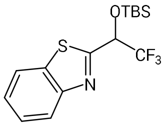

13-TBS

2-(1-((*tert*-butyldimethylsilyl)oxy)-2,2,2-trifluoroethyl)benzo[*d*]thiazole (13-TBS)

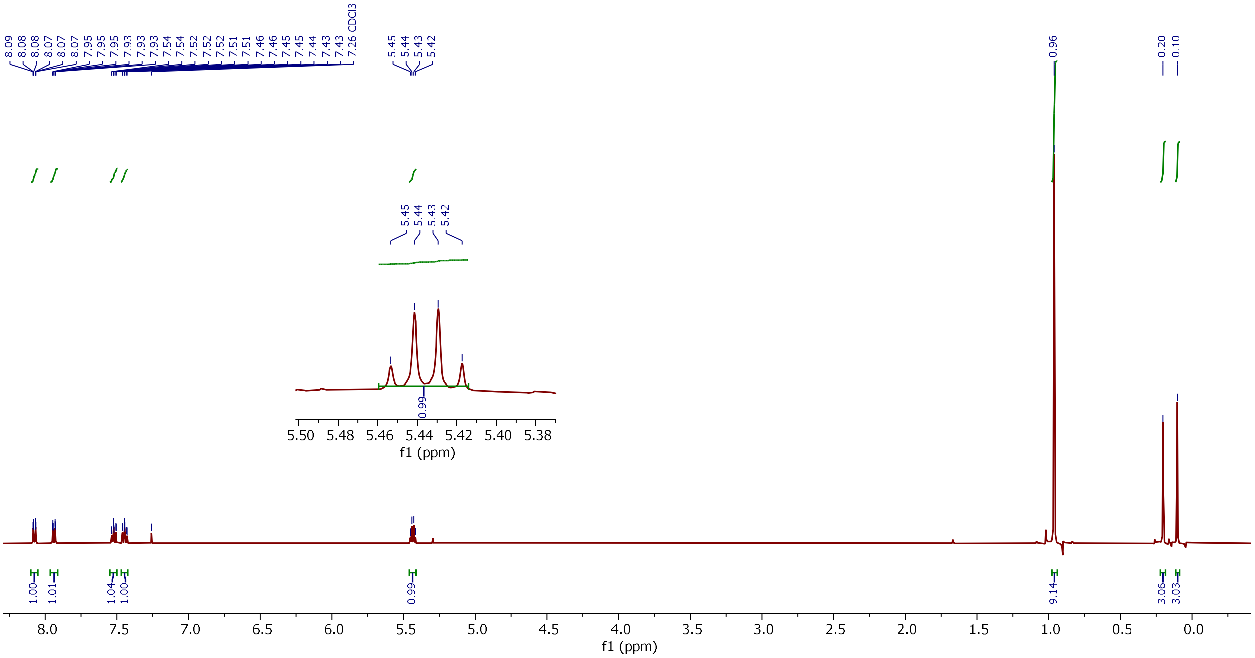

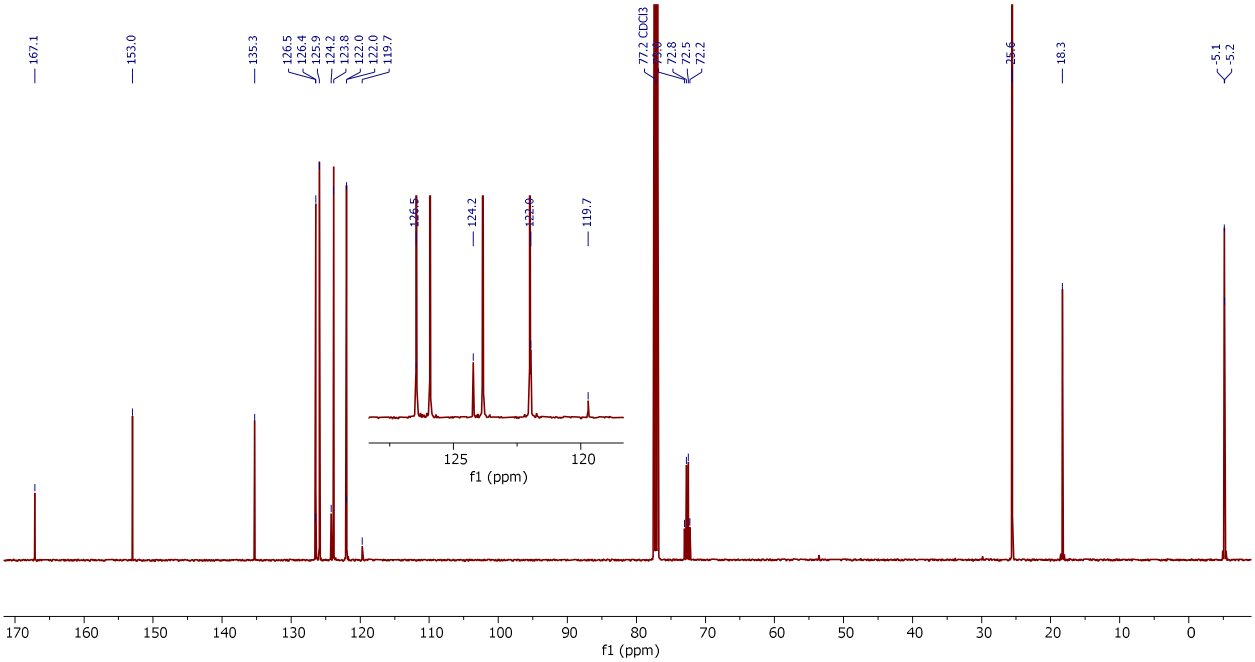

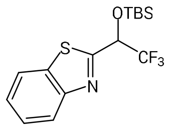

13-TBS

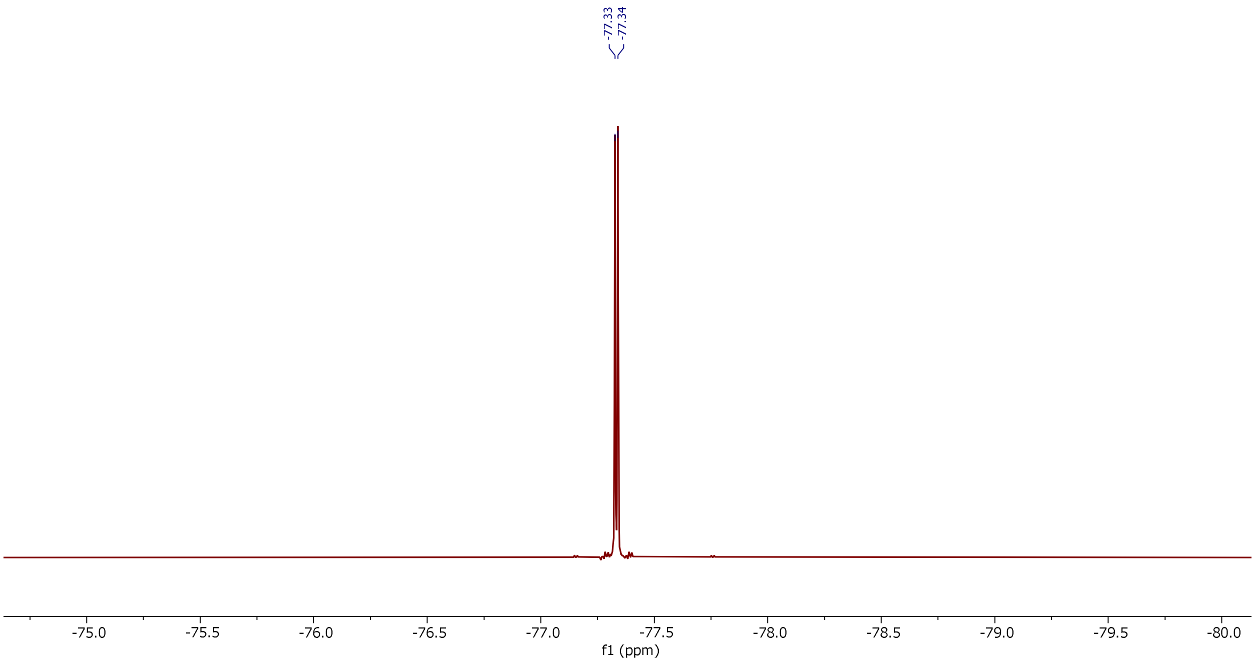

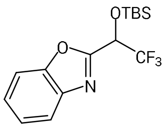

14-TBS

2-(1-((*tert*-butyldimethylsilyl)oxy)-2,2,2-trifluoroethyl)benzo[*d*]oxazole (14-TBS)

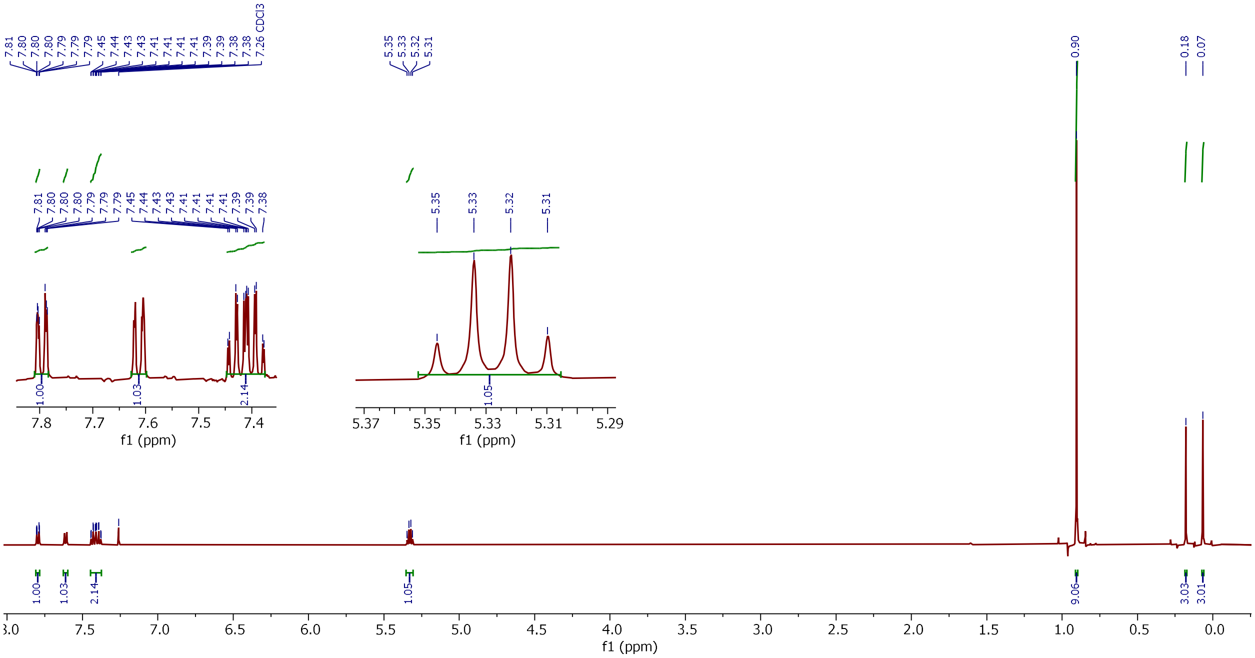

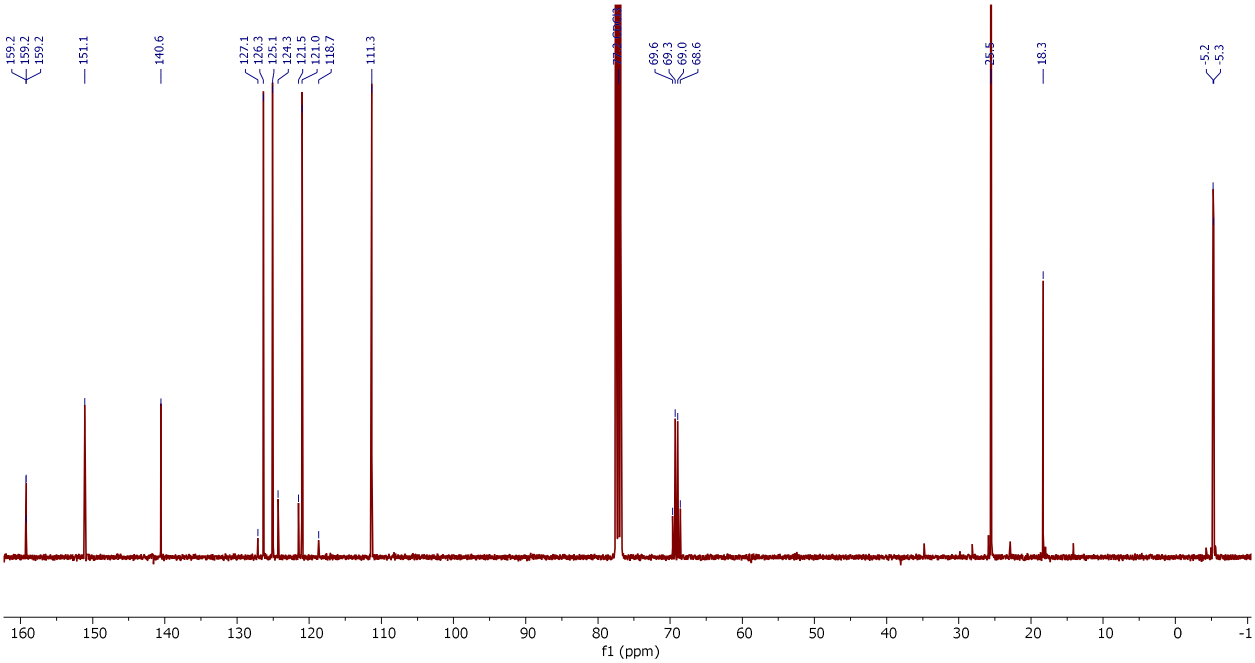

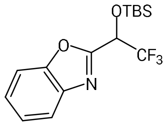
OTBS
O
N
CF3

14-TBS

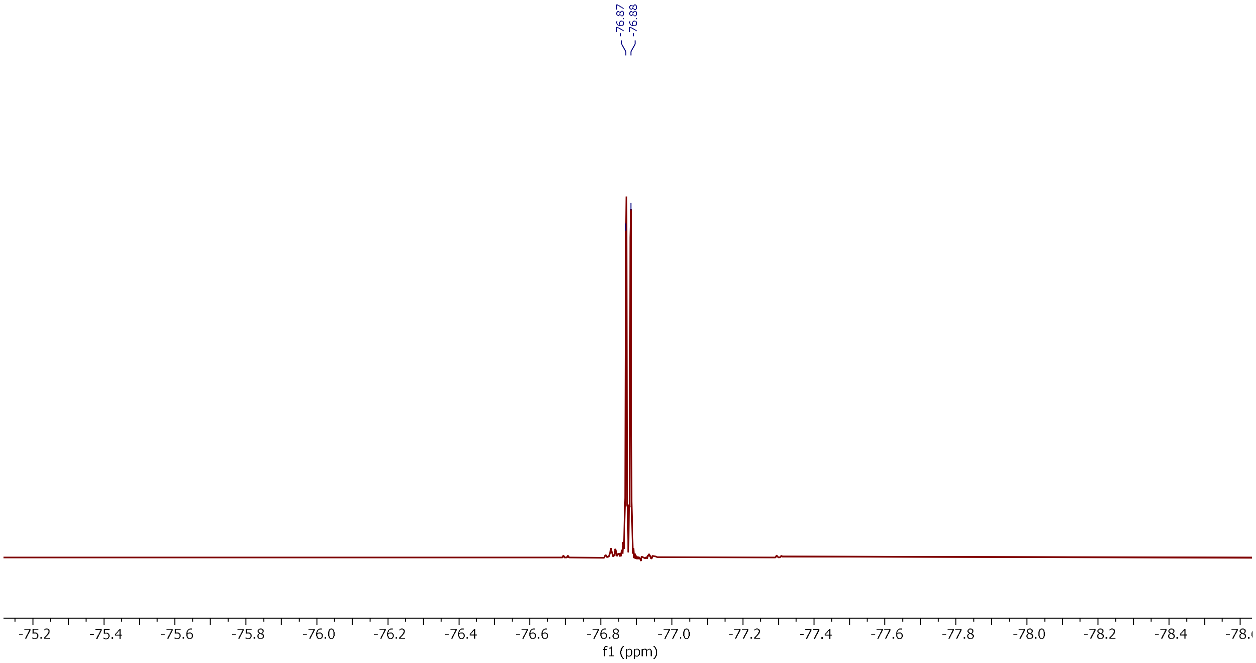
-76.87
-76.88
-75.2
-75.4
-75.6
-75.8
-76.0
-76.2
-76.4
-76.6
-76.8
-77.0
-77.2
-77.4
-77.6
-77.8
-78.0
-78.2
-78.4
f1 (ppm)

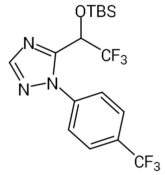

16-TBS

5-(1-((*tert*-butyldimethylsilyl)oxy)-2,2,2-trifluoroethyl)-1-(4-(trifluoromethyl)phenyl)-1*H*-1,2,4-triazole (16-TBS)

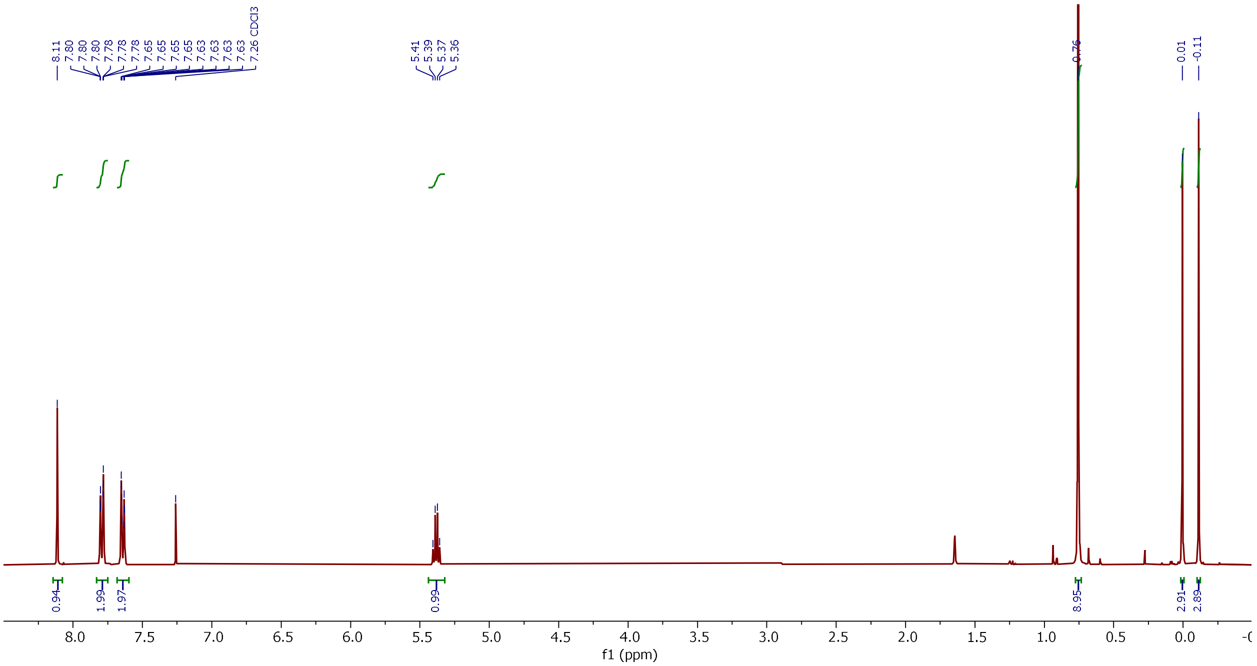

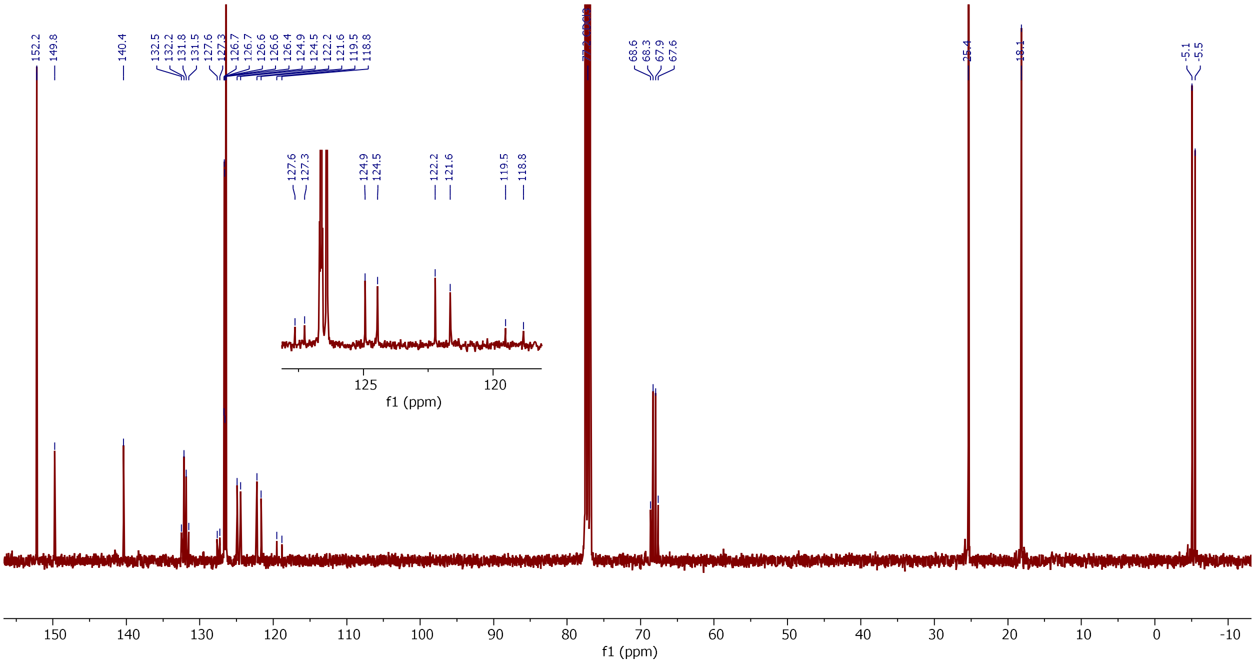

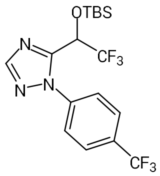
OTBS
N
N
N
CF3
CF3

16-TBS

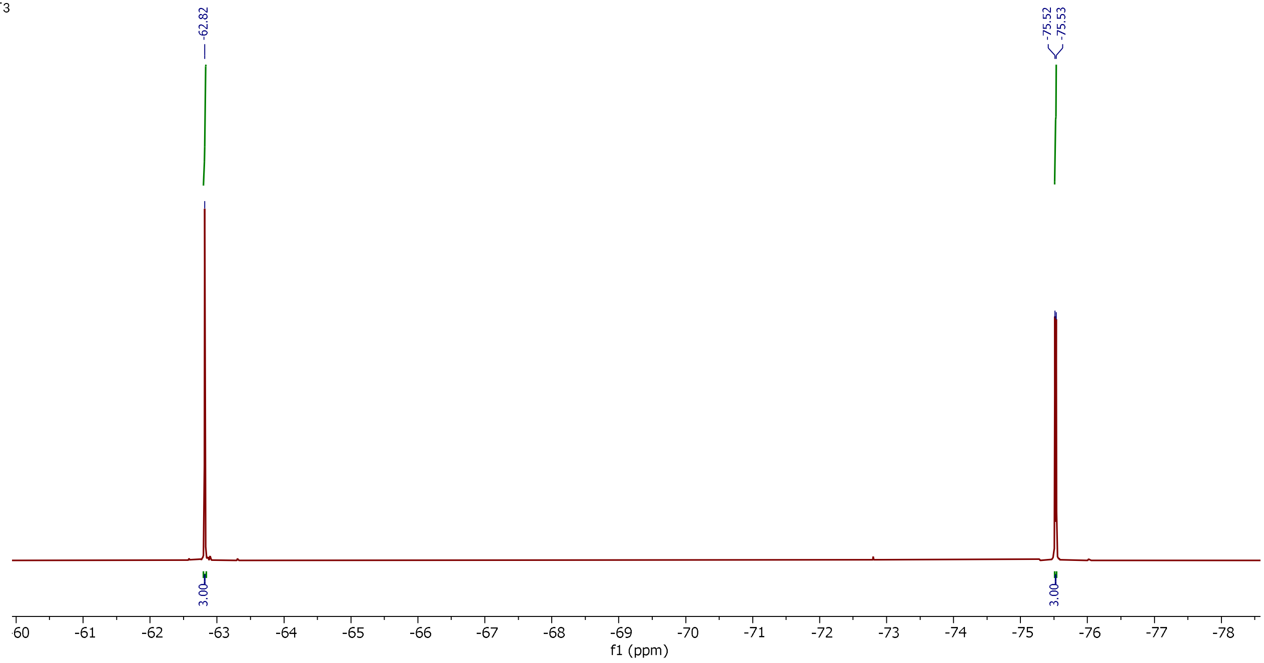
-62.82
-75.52
-75.53
3.00
3.00
60
-61
-62
-63
-64
-65
-66
-67
-68
-69
-70
-71
-72
-73
-74
-75
-76
-77
-78
f1 (ppm)

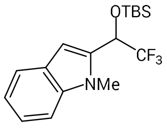

19-TBS

2-(1-((*tert*-butyldimethylsilyl)oxy)-2,2,2-trifluoroethyl)-1-methyl-1*H*-indole (19-TBS)

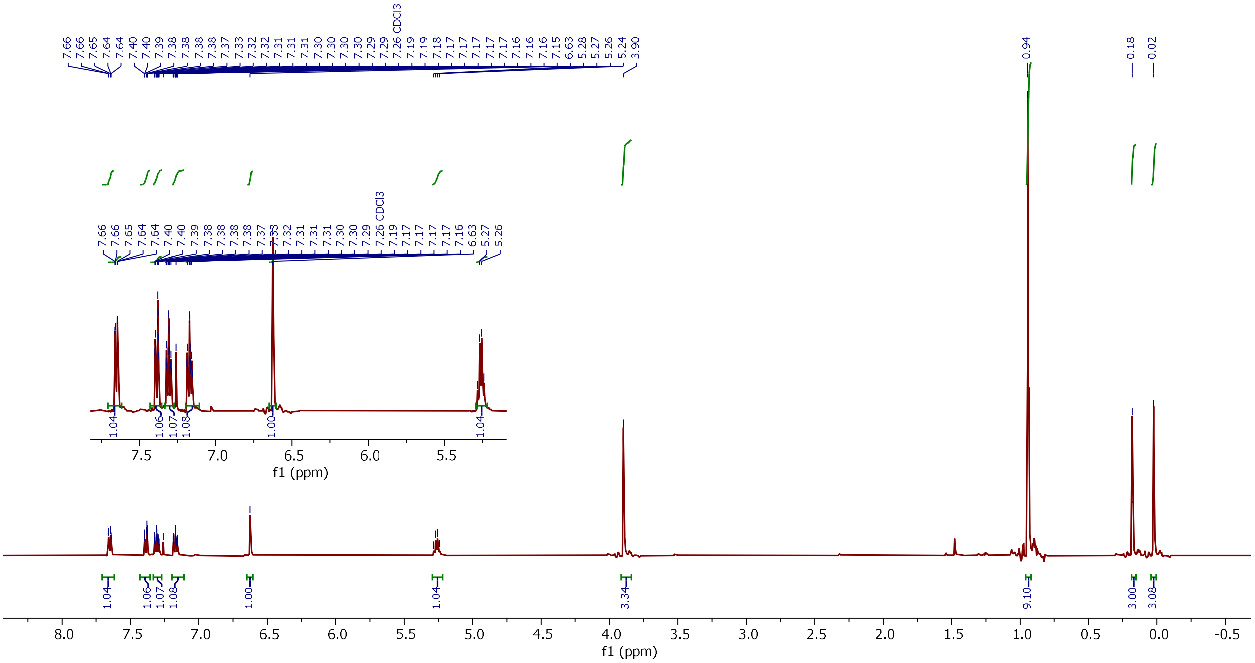

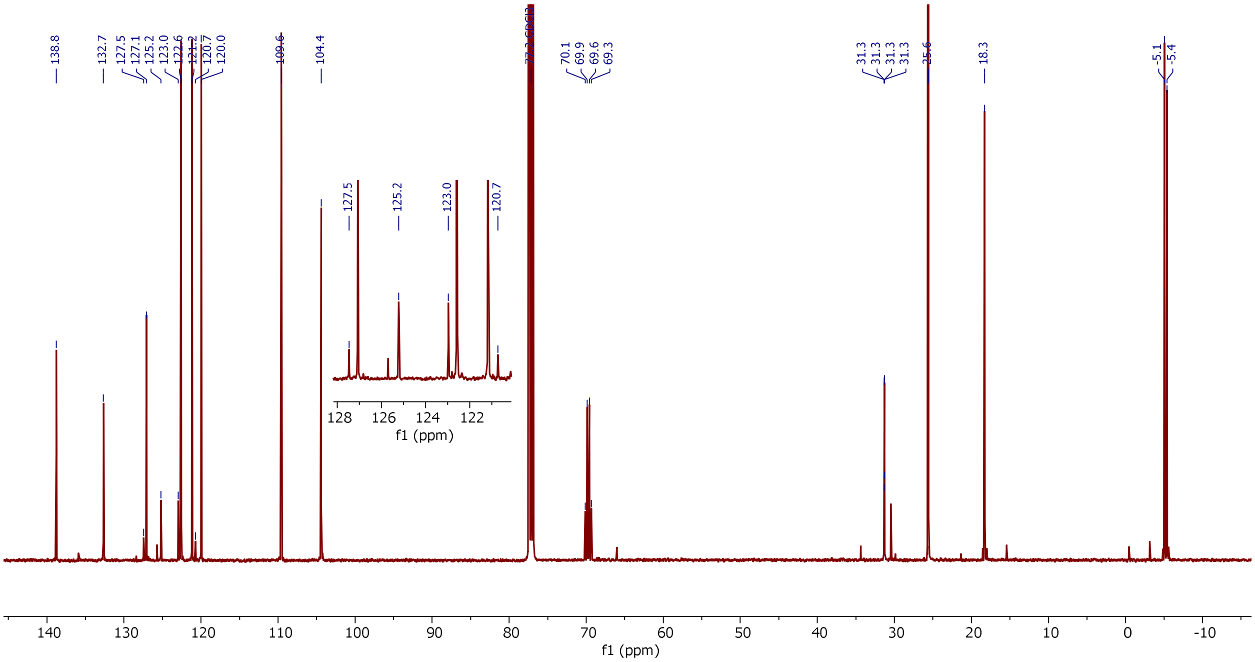

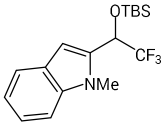
OTBS
CF3
NMe

19-TBS

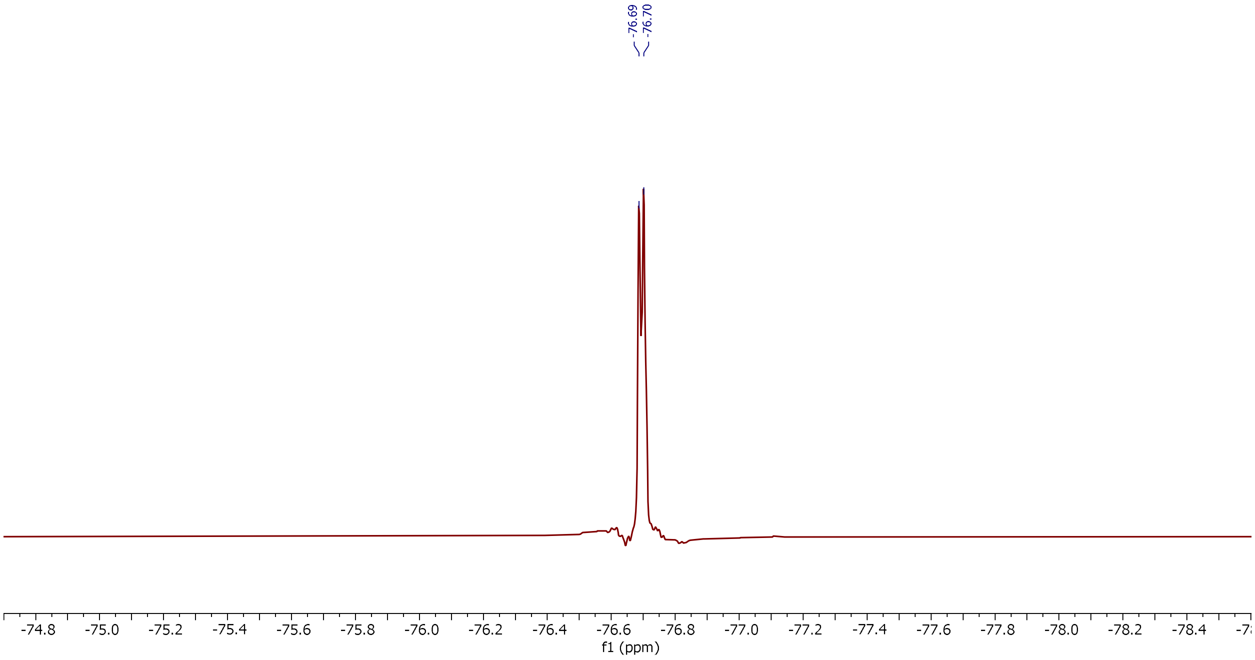
-76.69
-76.70
-74.8 -75.0 -75.2 -75.4 -75.6 -75.8 -76.0 -76.2 -76.4 -76.6 -76.8 -77.0 -77.2 -77.4 -77.6 -77.8 -78.0 -78.2 -78.4
f1 (ppm)

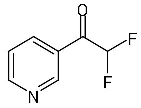

1-DFMK

2,2-difluoro-1-(pyridin-3-yl)ethan-1-one (1-DFMK)

Note: due to volatility, isolated as HCl salt in hydrate form (>20:1 hydrate:ketone)

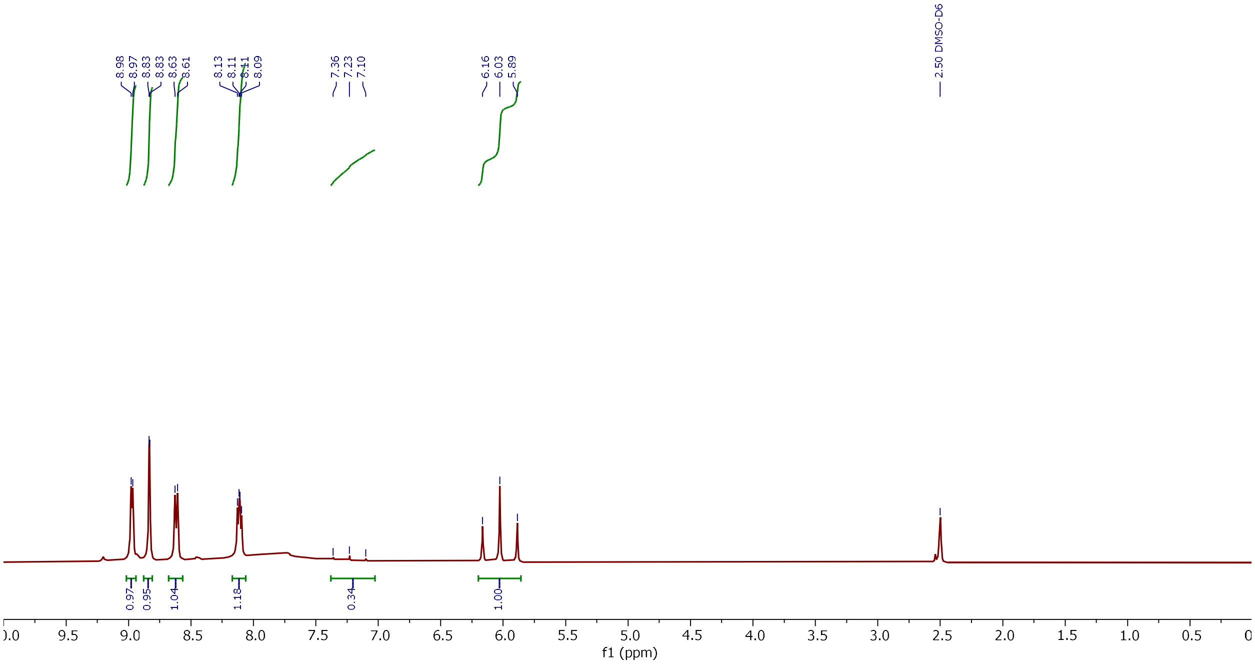

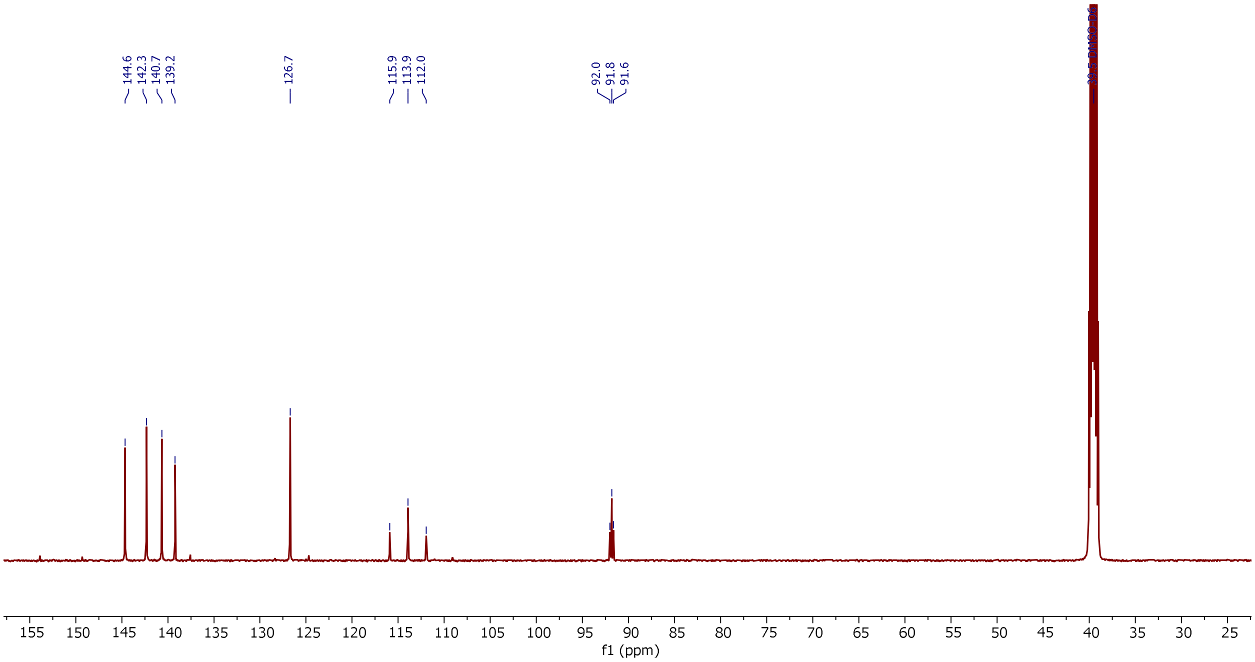

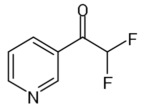

1-DFMK

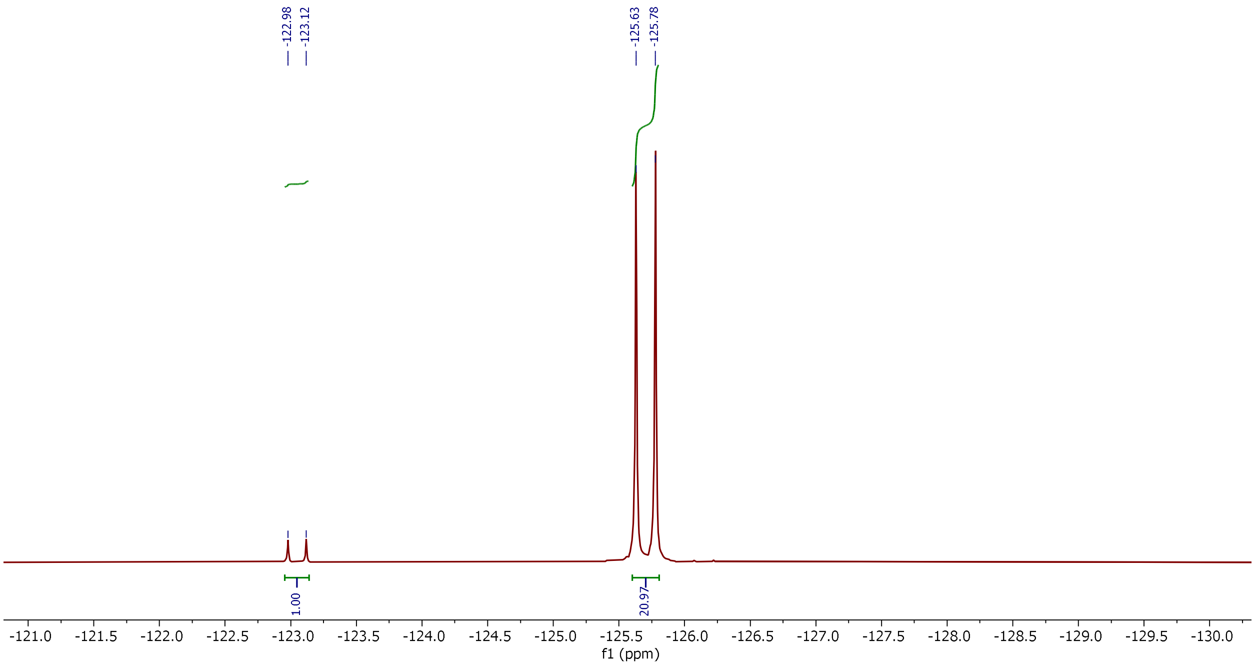
-122.98
-123.12
-125.63
-125.78
1.00
20.97
-121.0 -121.5 -122.0 -122.5 -123.0 -123.5 -124.0 -124.5 -125.0 -125.5 -126.0 -126.5 -127.0 -127.5 -128.0 -128.5 -129.0 -129.5 -130.0
f1 (ppm)

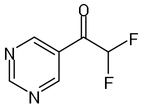

8-DFMK

2,2-difluoro-1-(pyrimidin-5-yl)ethan-1-one (8-DFMK)

Note: isolated as mixture of ketone:hydrate (1.2:1)

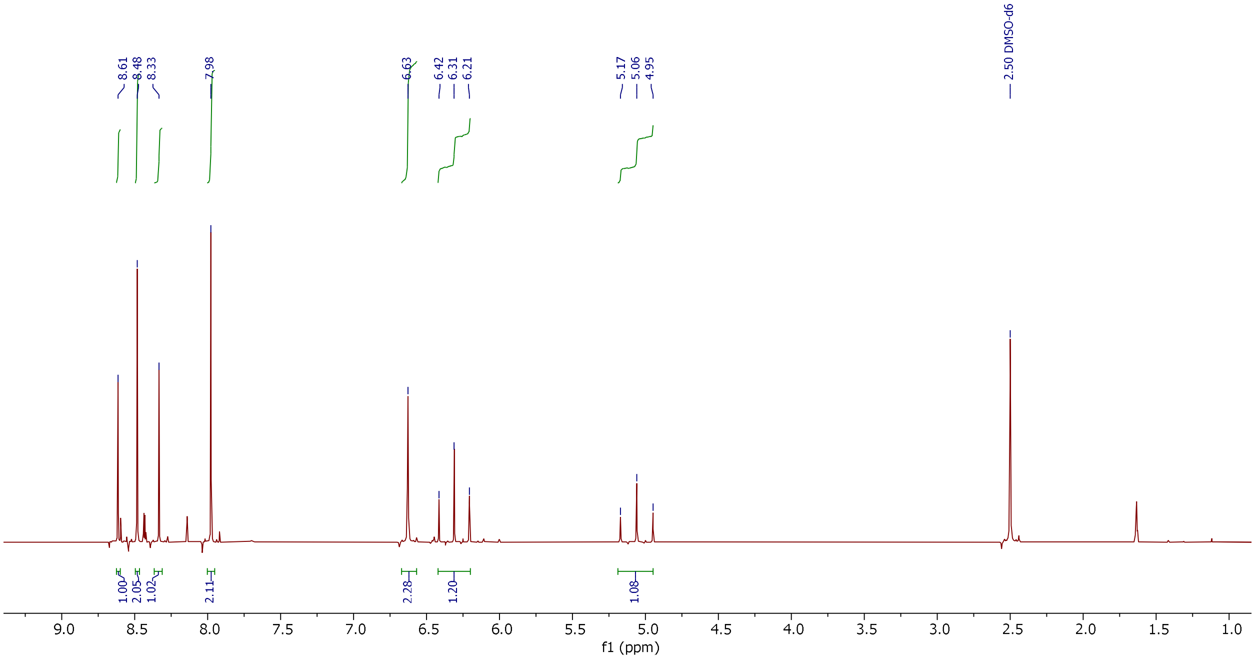

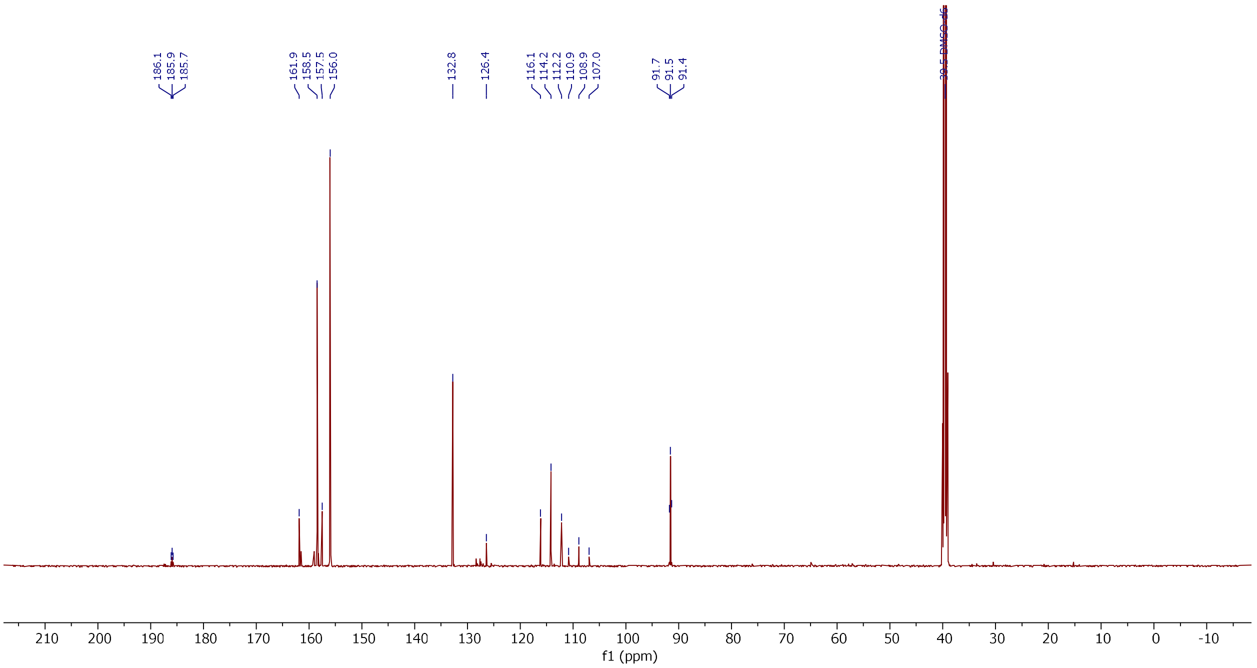

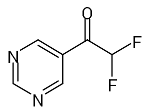

8-DFMK

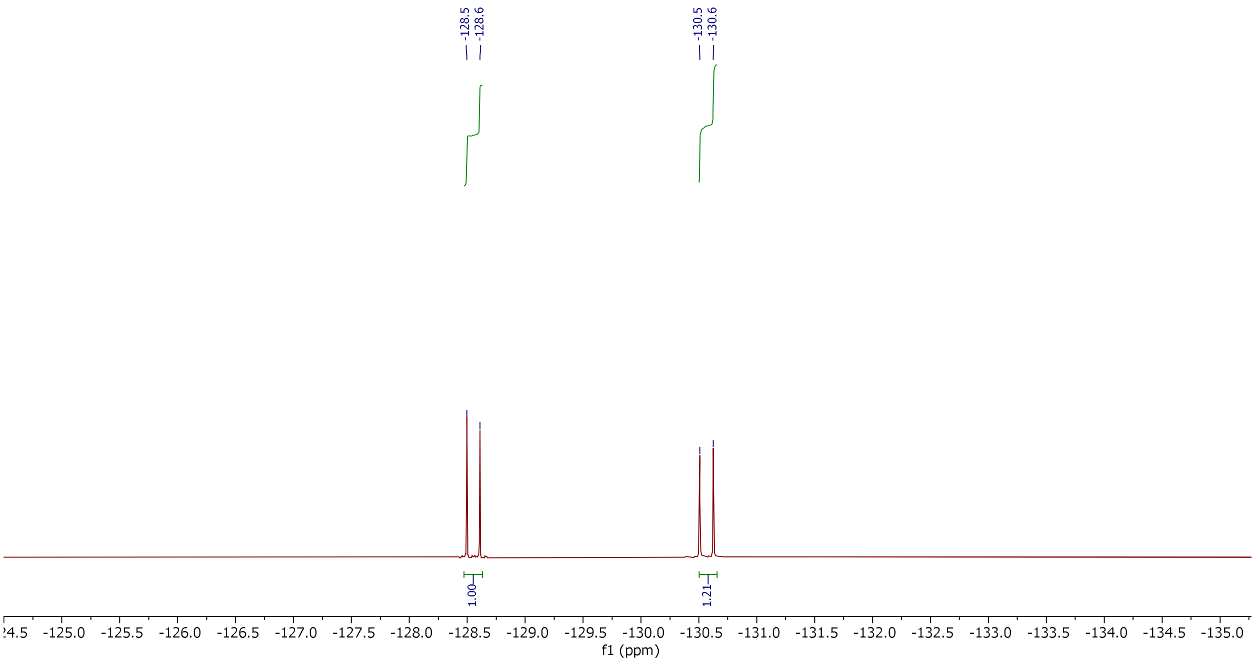

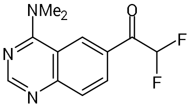

7-DFMK

1-(4-(dimethylamino)quinazolin-6-yl)-2,2-difluoroethan-1-one (7-DFMK)

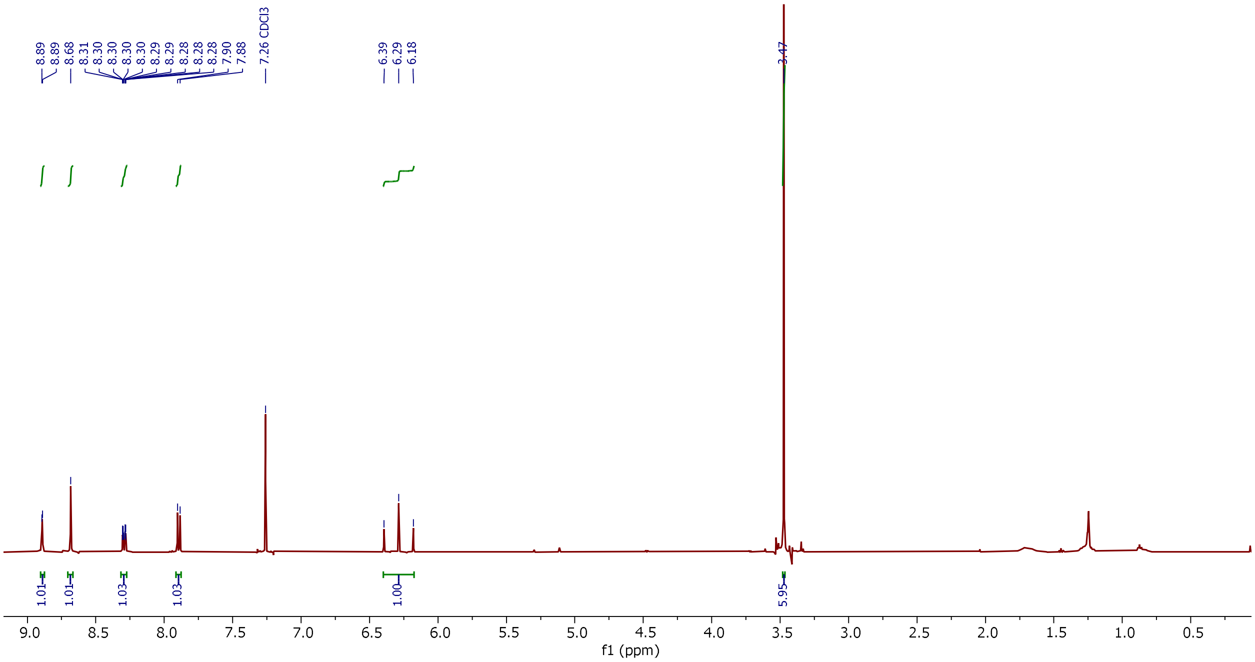

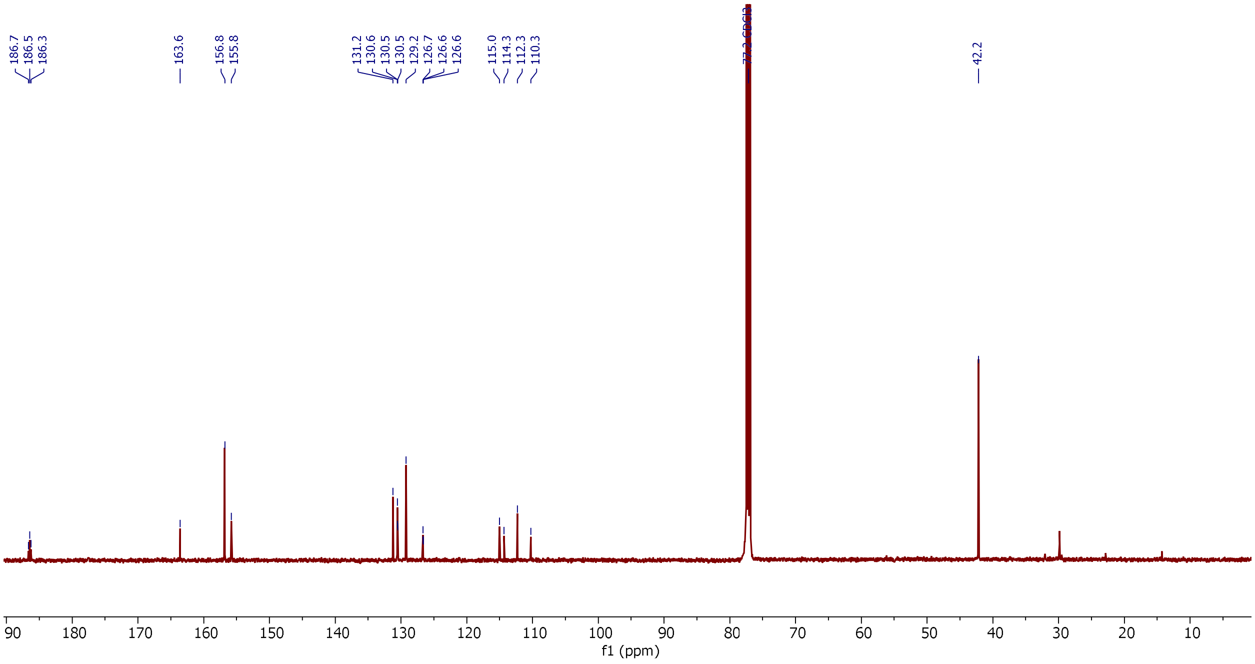

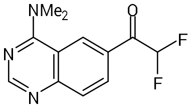
NMe2
N
N
O
F
F

7-DFMK

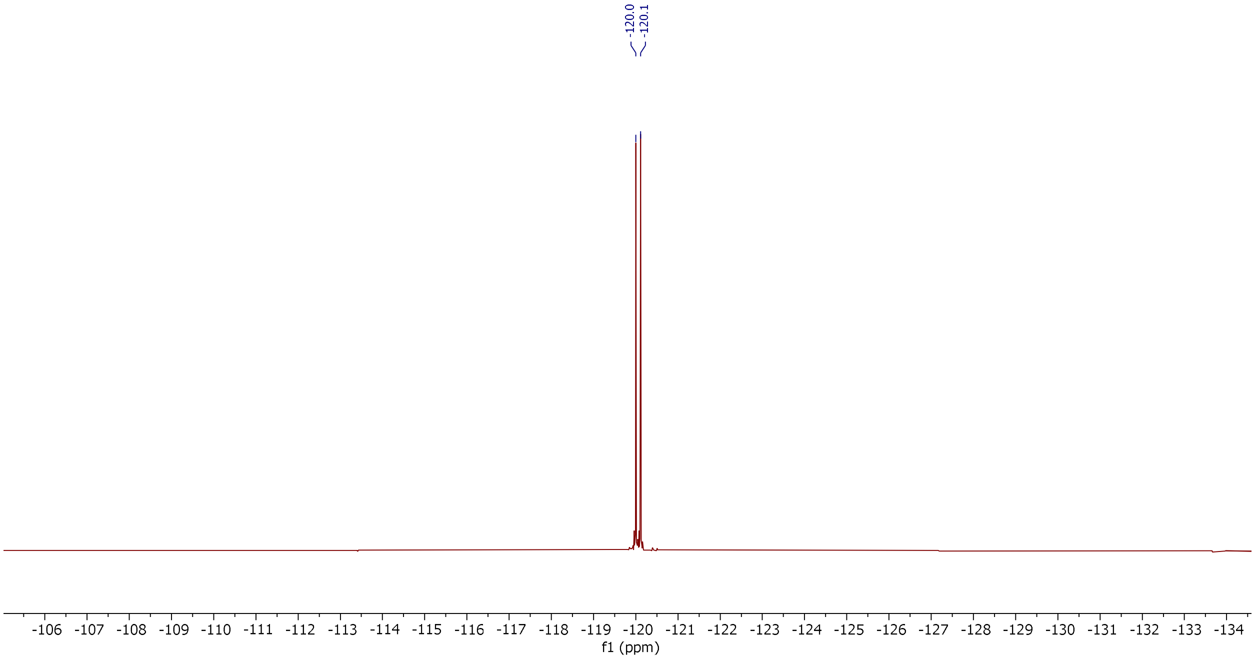
-120.0
-120.1
-106 -107 -108 -109 -110 -111 -112 -113 -114 -115 -116 -117 -118 -119 -120 -121 -122 -123 -124 -125 -126 -127 -128 -129 -130 -131 -132 -133 -134
f1 (ppm)

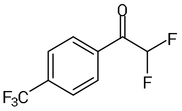

9-DFMK

2,2-difluoro-1-(4-(trifluoromethyl)phenyl)ethan-1-one (9-DFMK)

Note: isolated as mixture of ketone:hydrate (4.3:1)

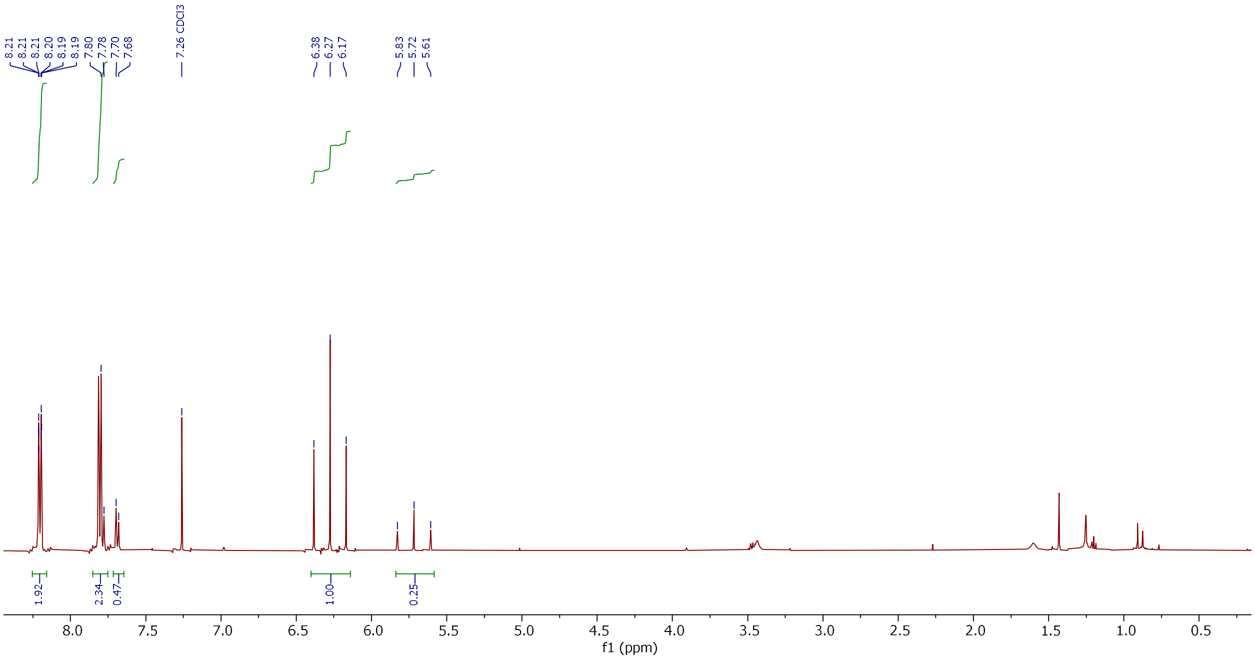

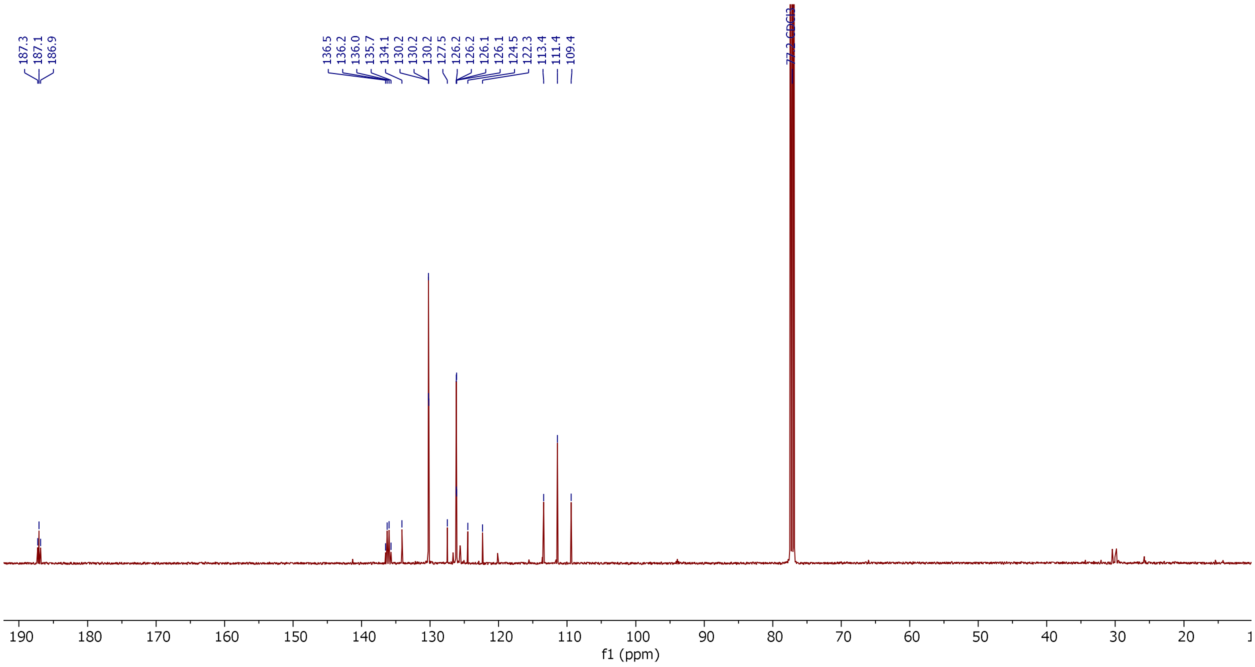

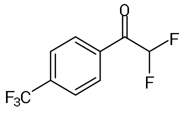

9-DFMK

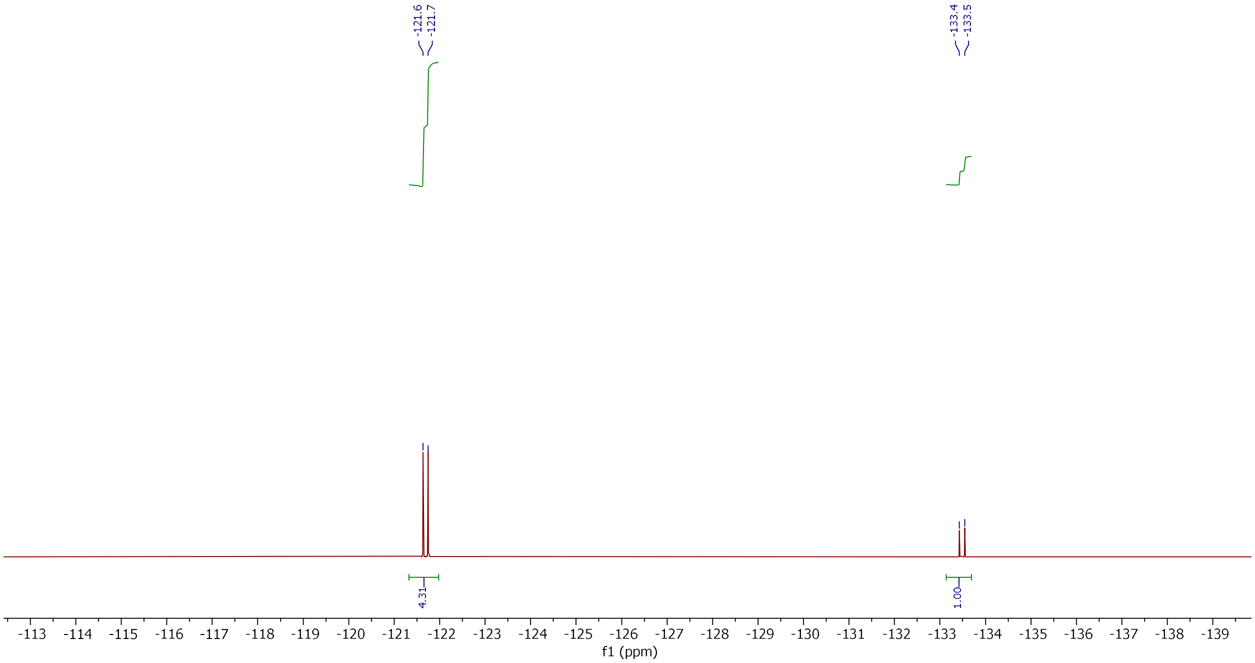
-121.6
-121.7
-133.4
-133.5
4.31
1.00
-113 -114 -115 -116 -117 -118 -119 -120 -121 -122 -123 -124 -125 -126 -127 -128 -129 -130 -131 -132 -133 -134 -135 -136 -137 -138 -139
f1 (ppm)

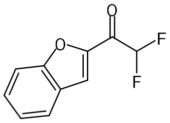

11-DFMK

1-(benzofuran-2-yl)-2,2-difluoroethan-1-one (11-DFMK)

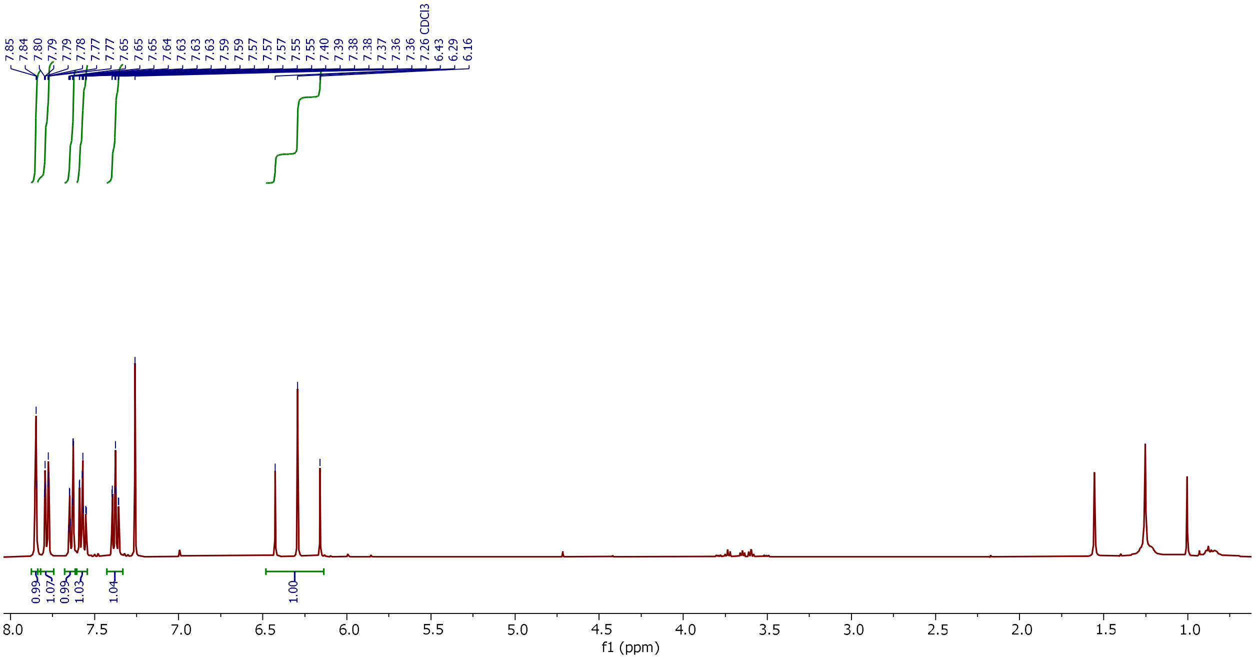

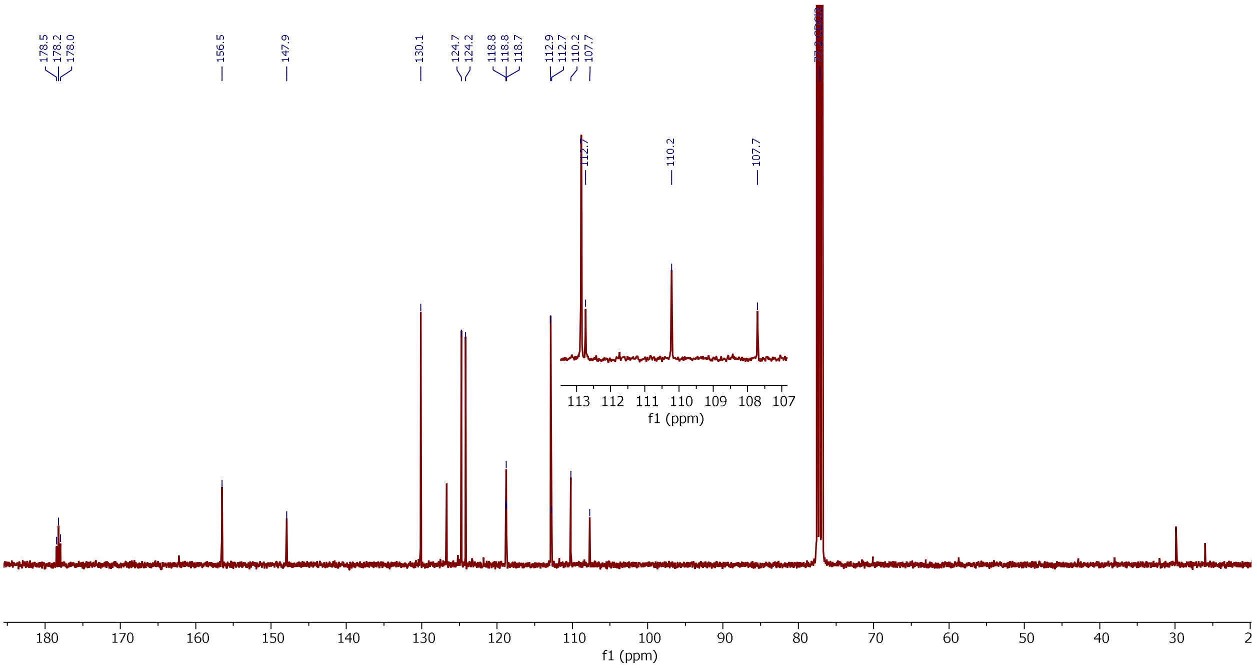

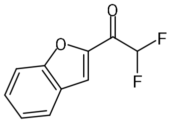

11-DFMK

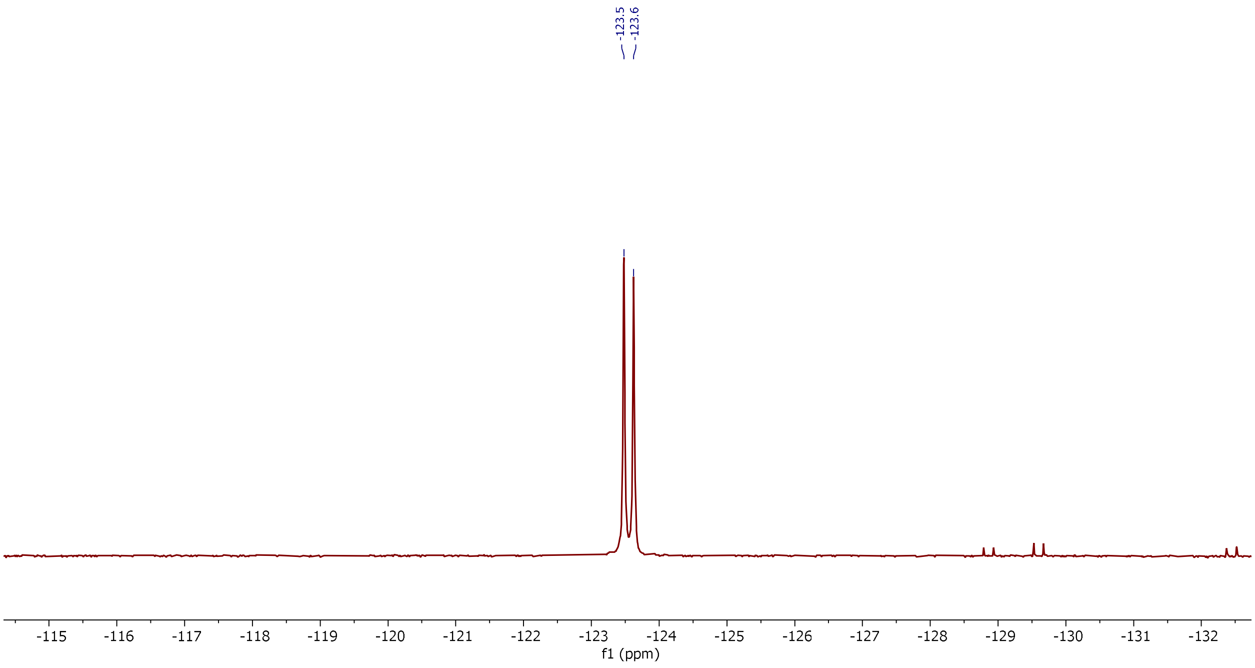

-123.5
-123.6
-115
-116
-117
-118
-119
-120
-121
-122
-123
-124
-125
-126
-127
-128
-129
-130
-131
-132
f1 (ppm)

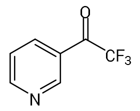

1-TFMK

2,2,2-trifluoro-1-(pyridin-3-yl)ethan-1-one (1-TFMK)

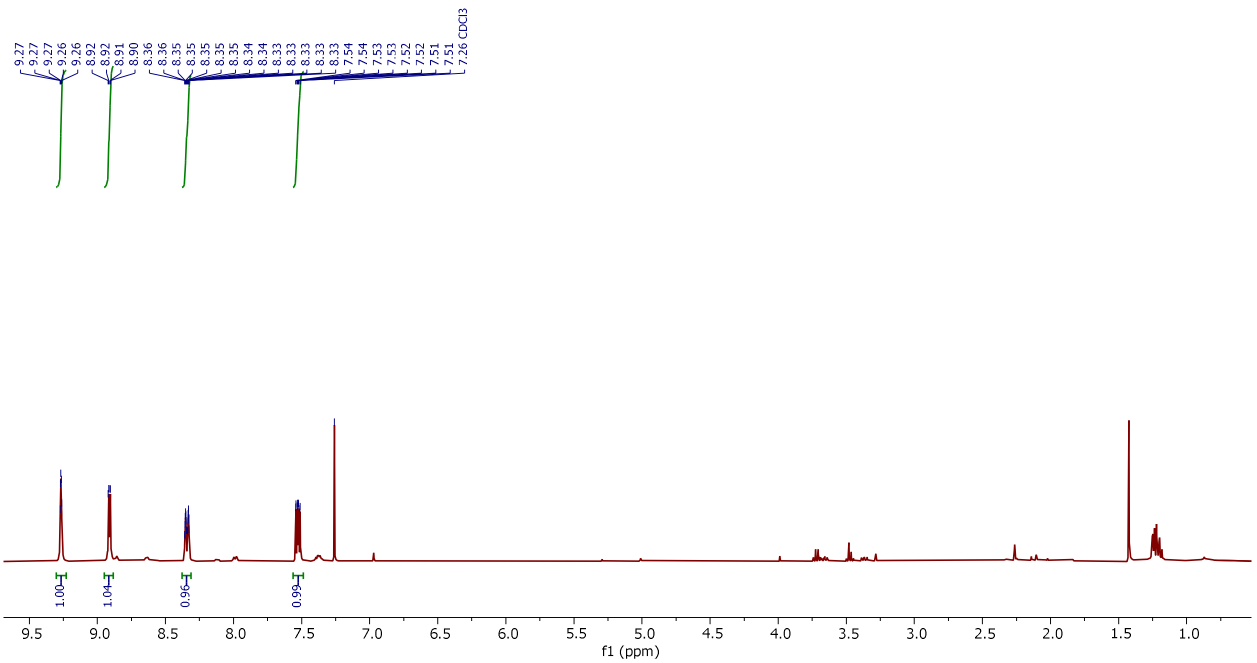

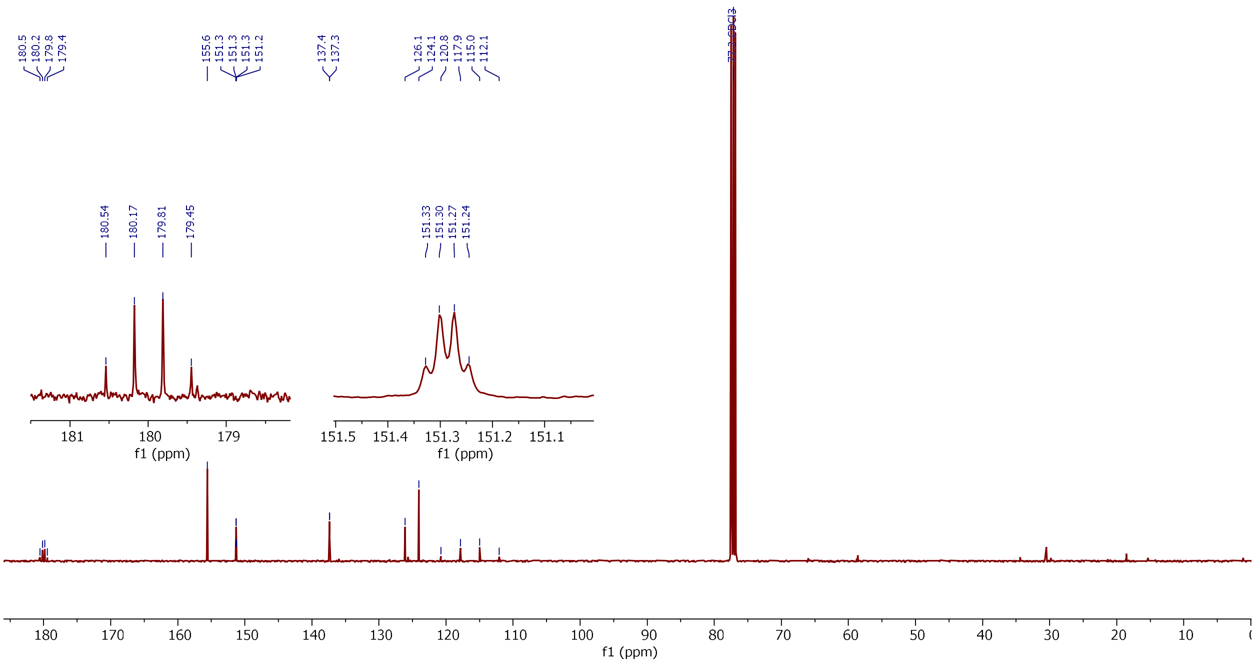

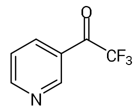

1-TFMK

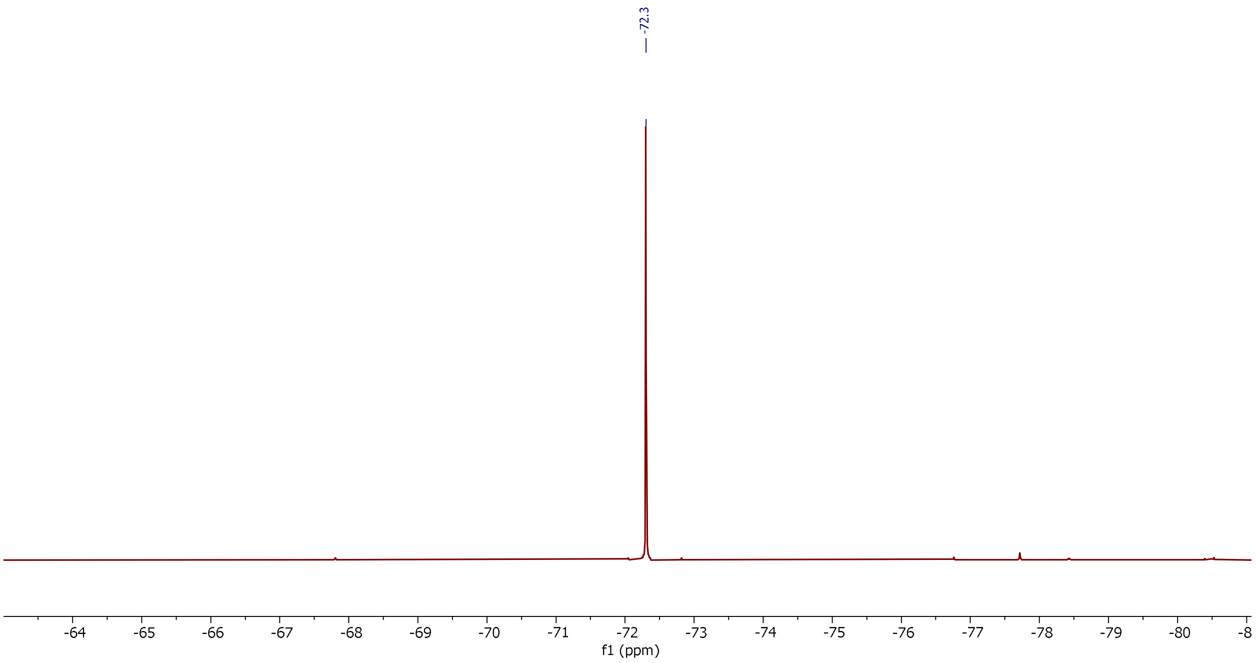

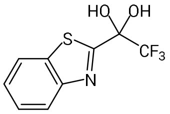

13-TFMK

1-(benzo[*d*]thiazol-2-yl)-2,2,2-trifluoroethan-1-one (13-TFMK)

Note: isolated in hydrate form.

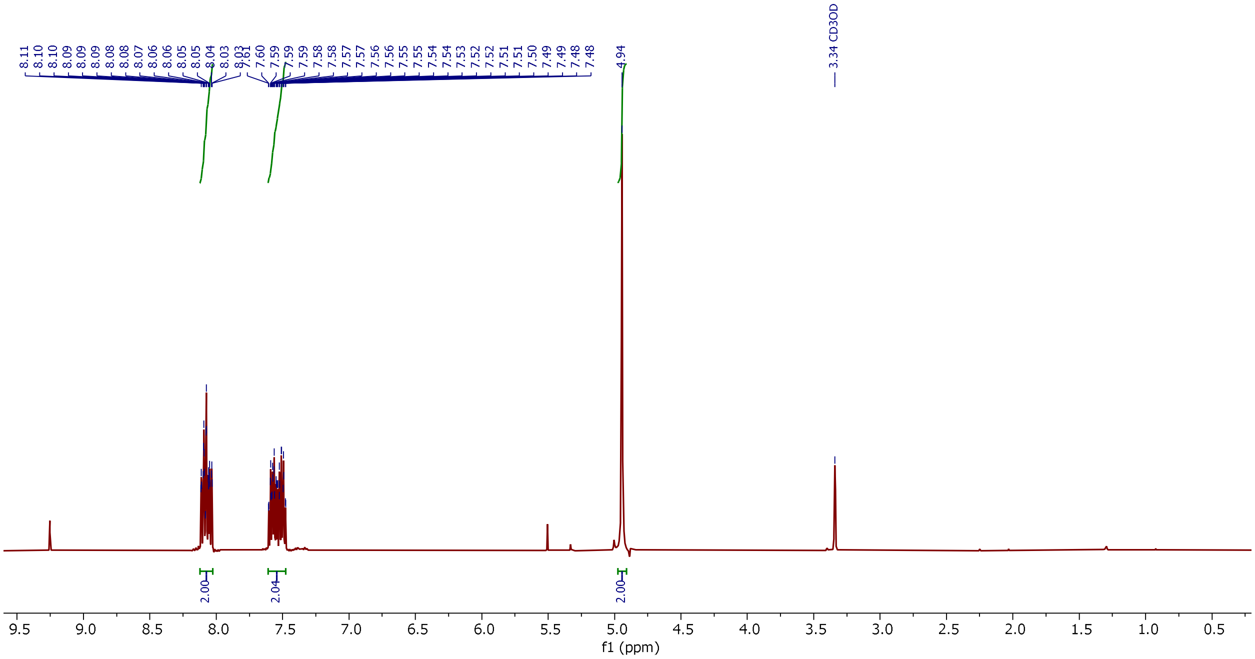

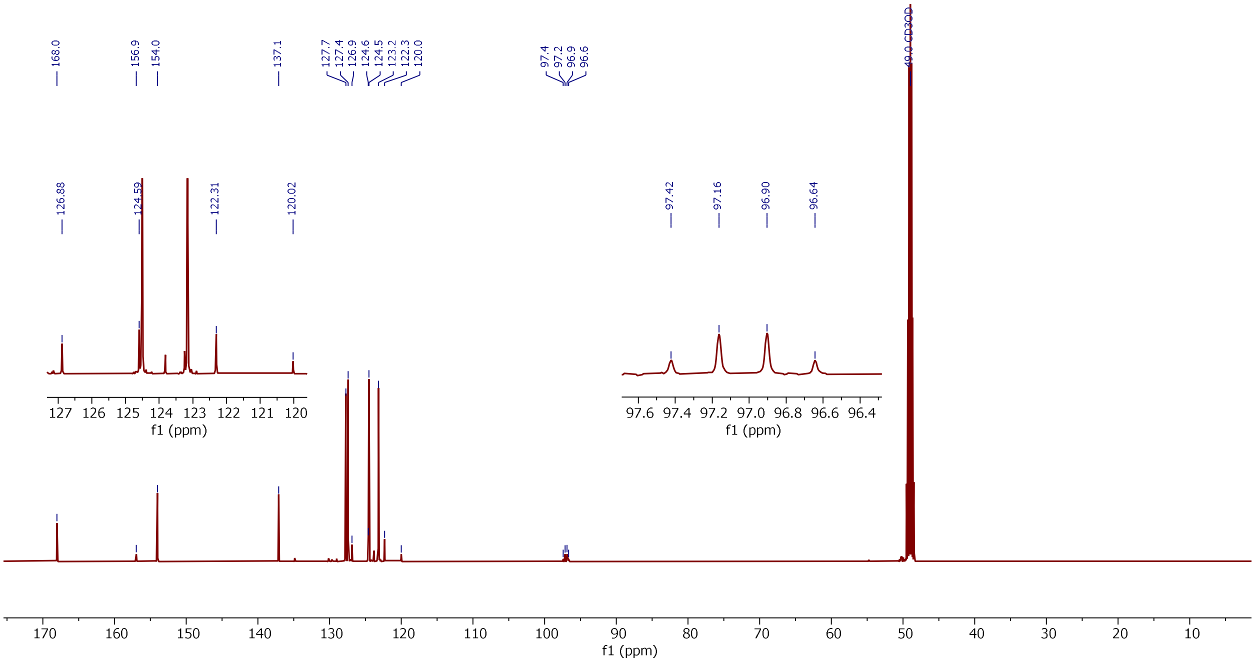

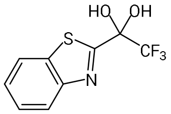

13-TFMK

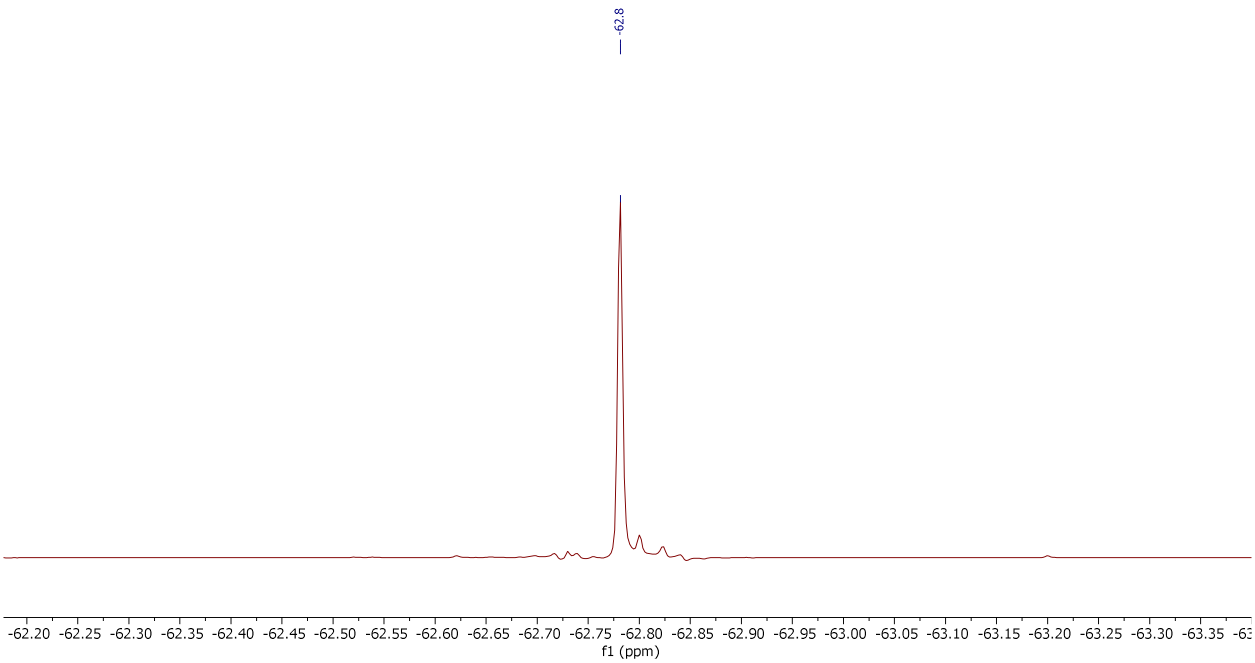
-62.8
-62.20 -62.25 -62.30 -62.35 -62.40 -62.45 -62.50 -62.55 -62.60 -62.65 -62.70 -62.75 -62.80 -62.85 -62.90 -62.95 -63.00 -63.05 -63.10 -63.15 -63.20 -63.25 -63.30 -63.35
f1 (ppm)

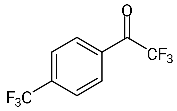

9-TFMK

2,2,2-trifluoro-1-(4-(trifluoromethyl)phenyl)ethan-1-one (9-TFMK)

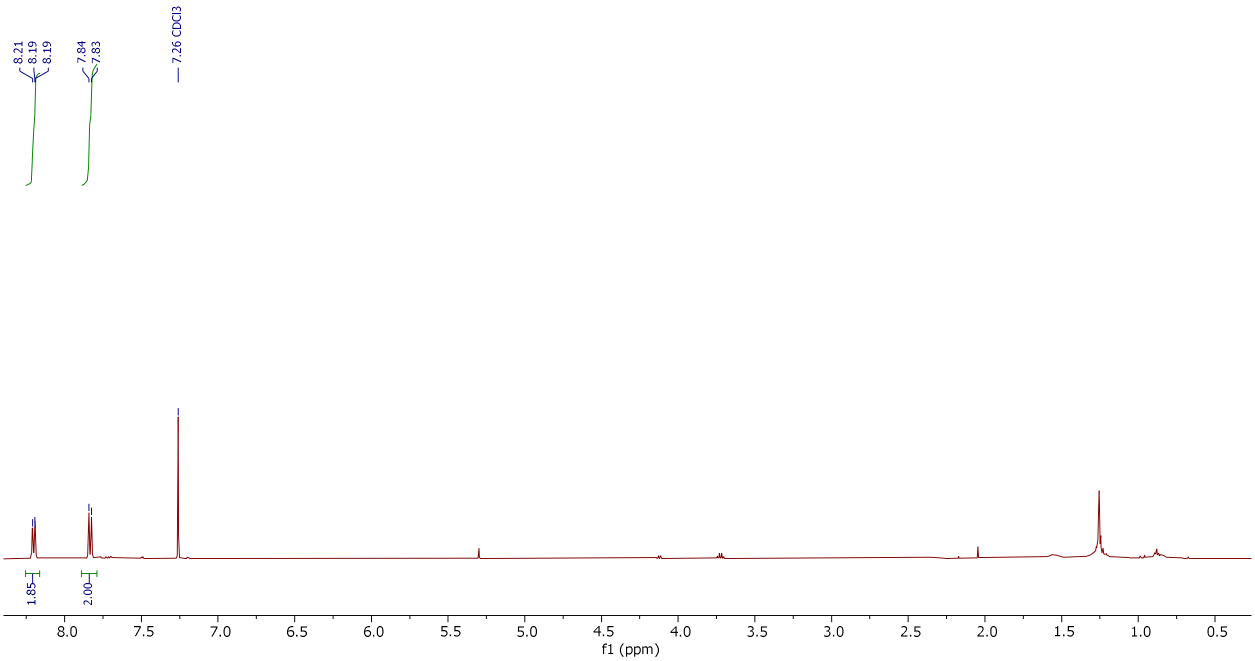

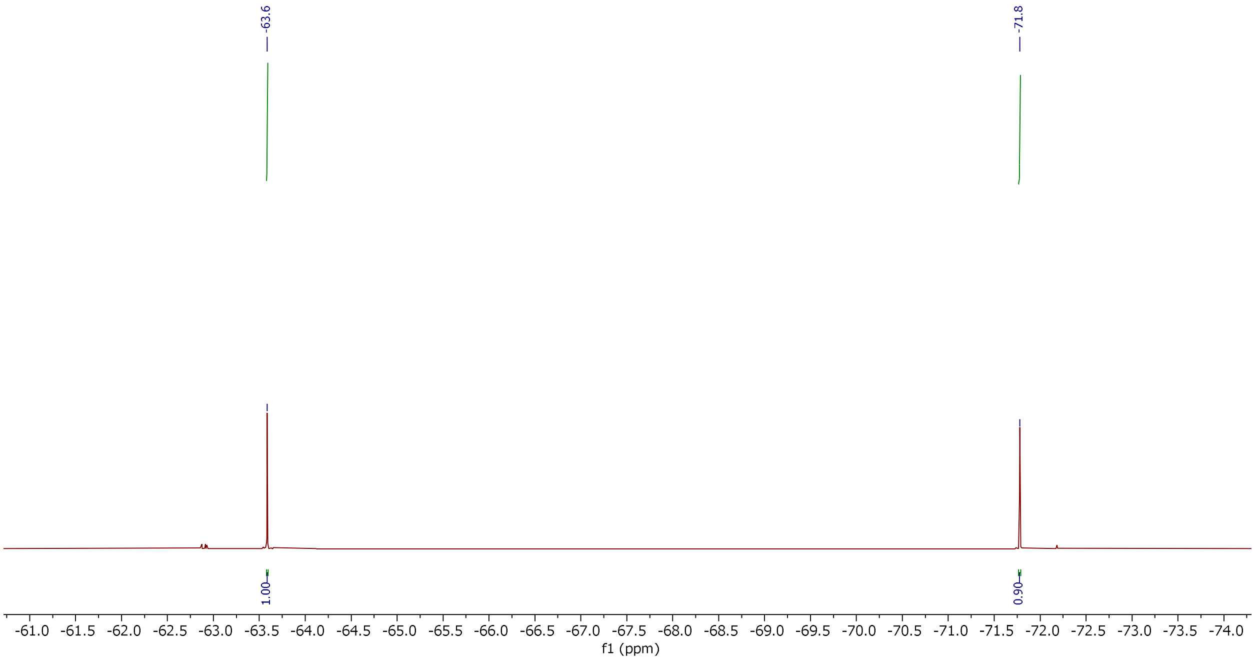

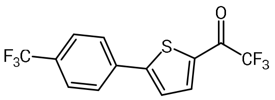

18-TFMK

2,2,2-trifluoro-1-(5-(4-(trifluoromethyl)phenyl)thiophen-2-yl)ethan-1-one (18-TFMK)

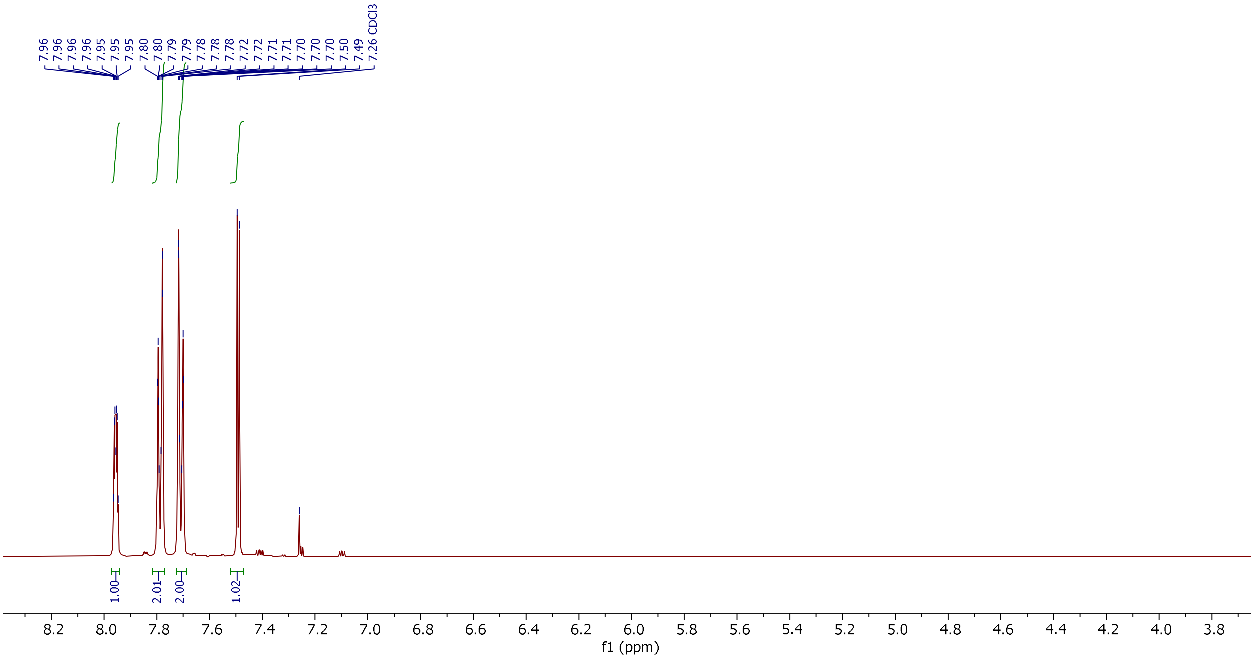

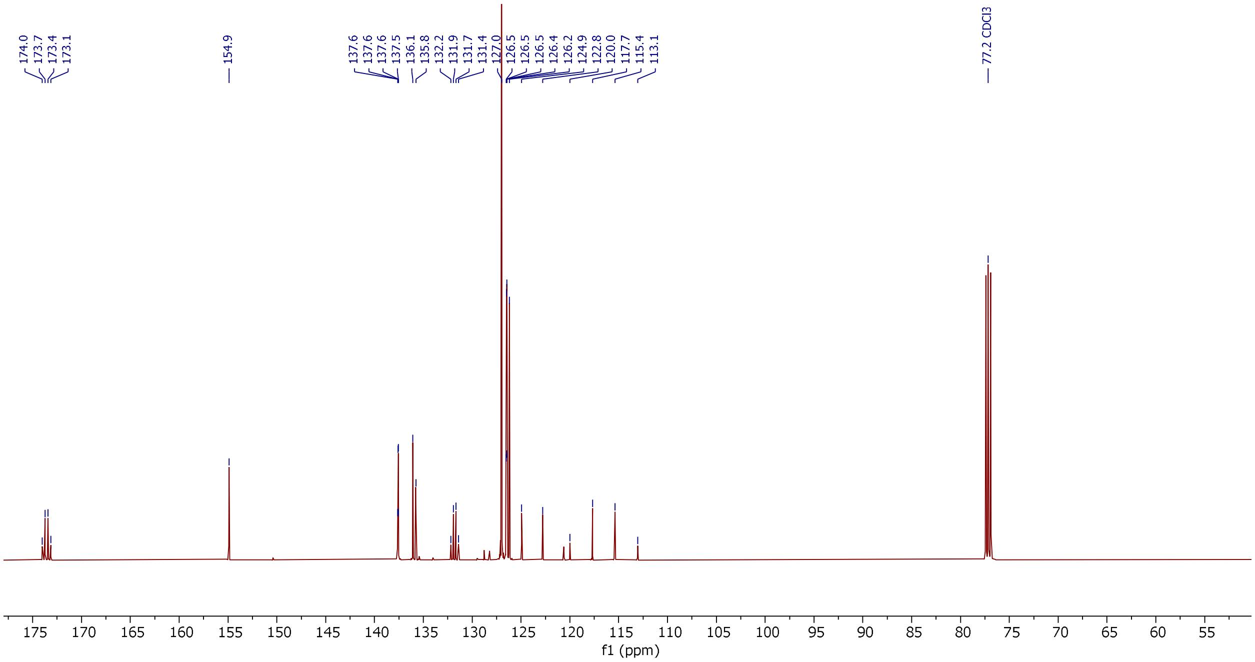

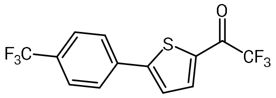

18-TFMK

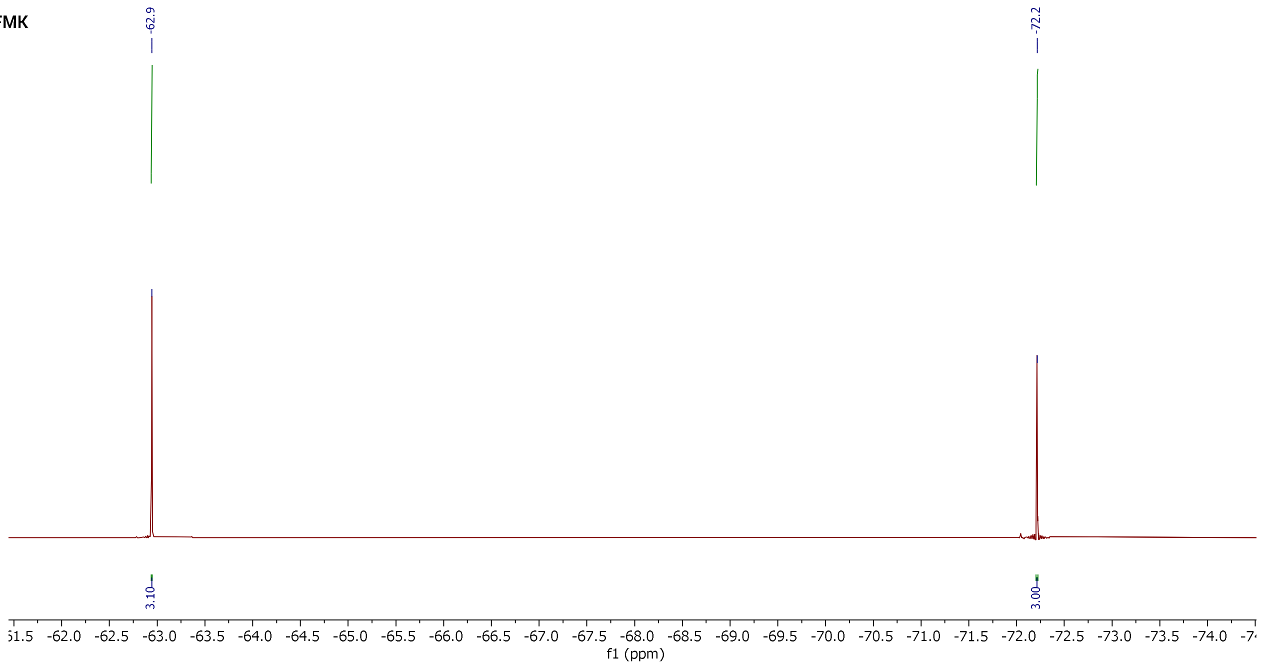

-62.9
-72.2
3.10
3.00
-61.5 -62.0 -62.5 -63.0 -63.5 -64.0 -64.5 -65.0 -65.5 -66.0 -66.5 -67.0 -67.5 -68.0 -68.5 -69.0 -69.5 -70.0 -70.5 -71.0 -71.5 -72.0 -72.5 -73.0 -73.5 -74.0
f1 (ppm)

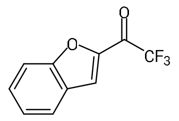

11-TFMK

1-(benzofuran-2-yl)-2,2,2-trifluoroethan-1-one (11-TFMK)

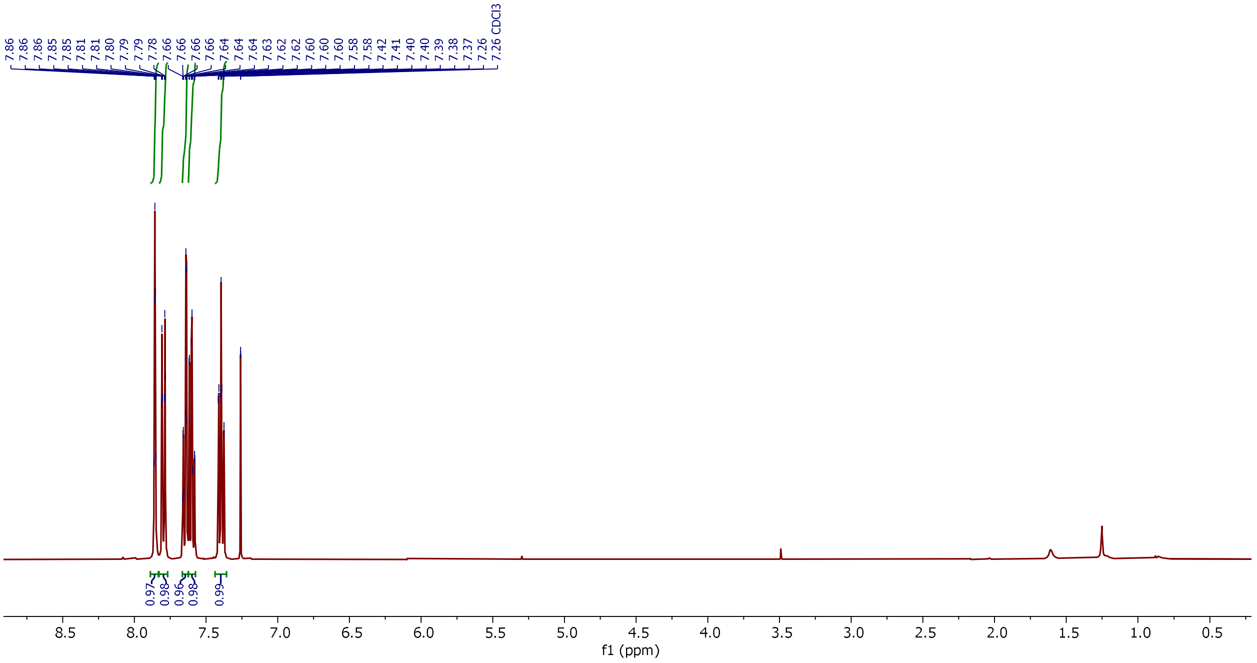

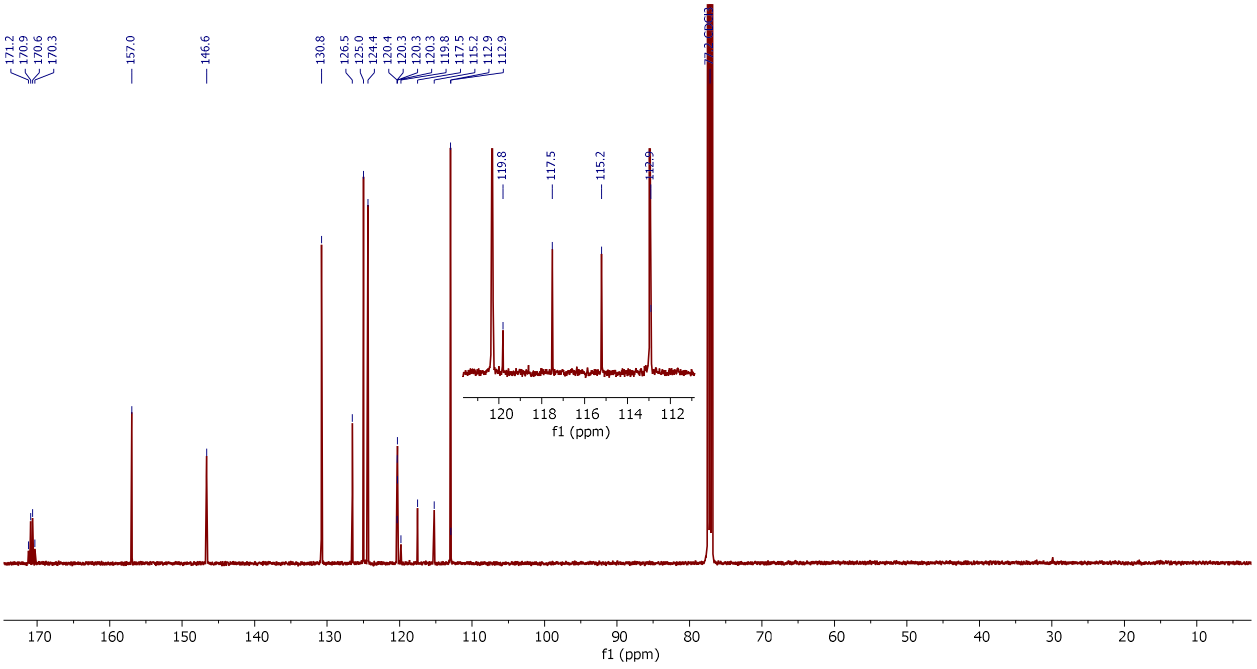

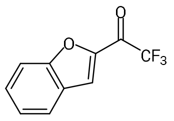

11-TFMK

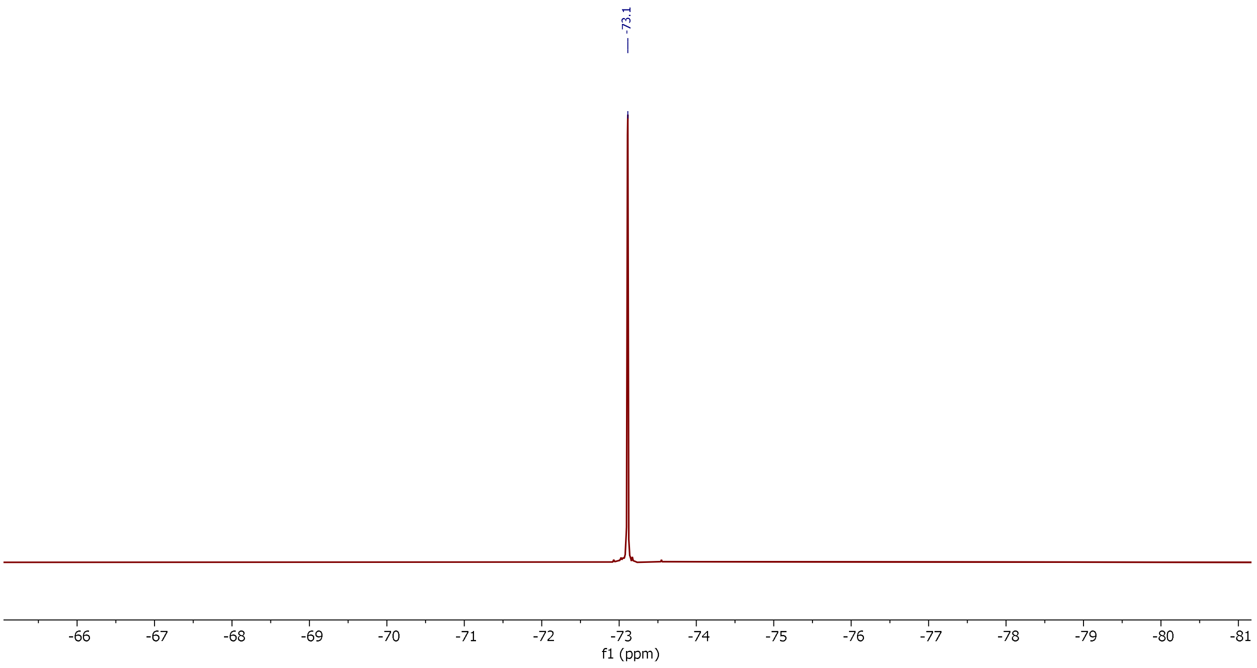

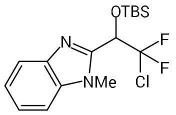

12-CDFE

2-(1-((*tert*-butyldimethylsilyl)oxy)-2-chloro-2,2-difluoroethyl)-1-methyl-1*H*-benzo[*d*]imidazole (12-CDFE)

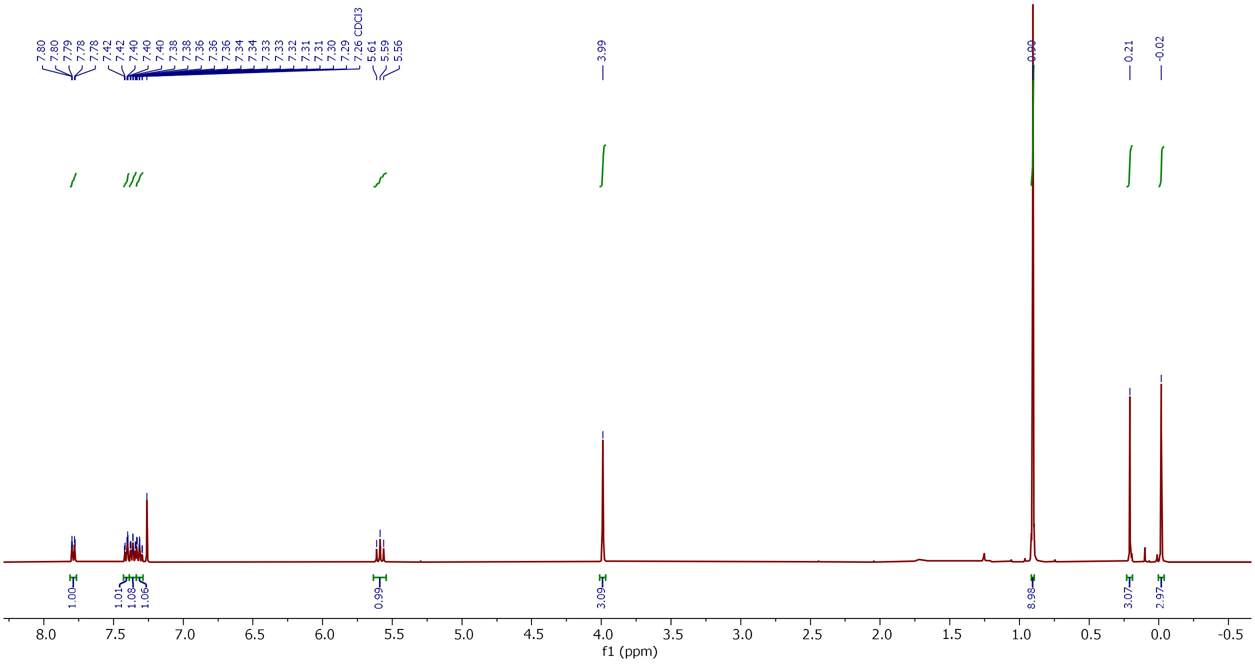

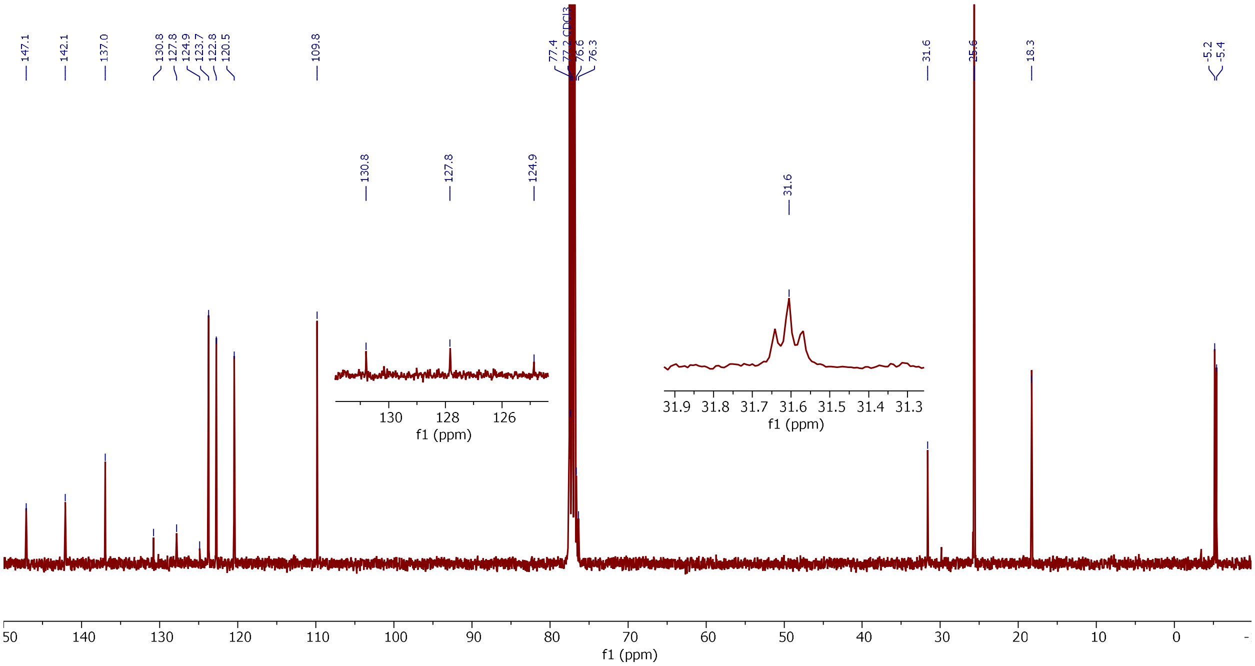

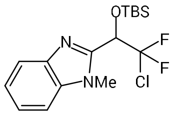
OTBS
N
NMe
F
F
Cl

12-CDFE

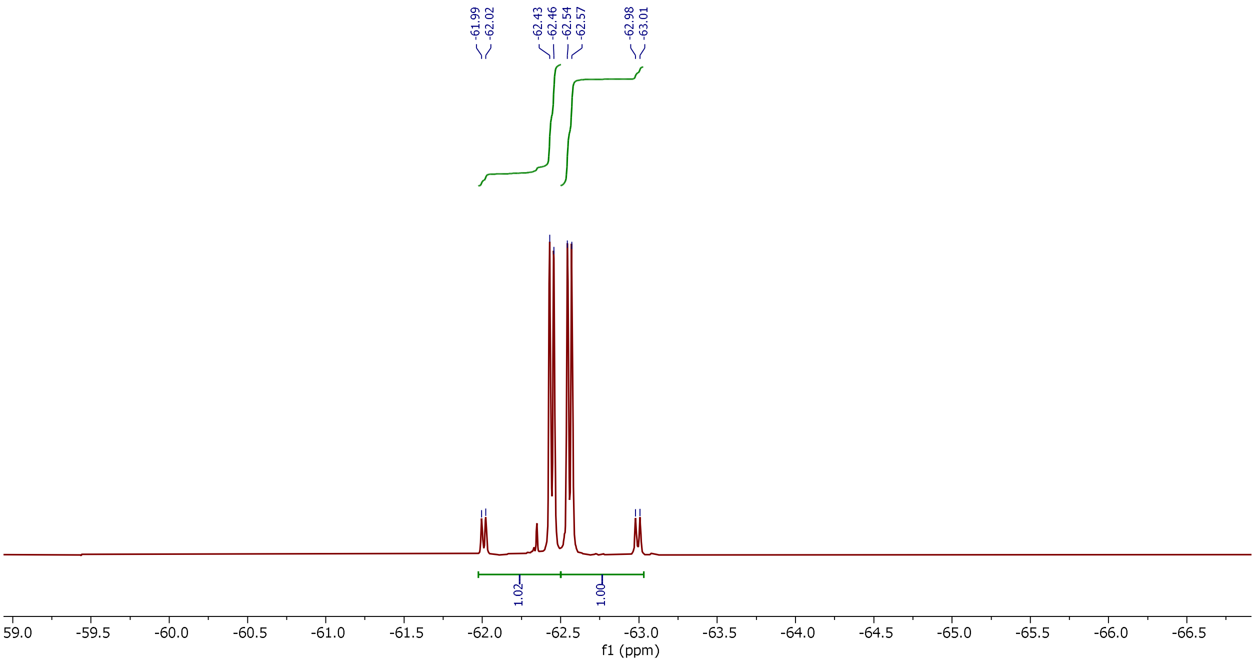
-61.99
-62.02
-62.43
-62.46
-62.54
-62.57
-62.98
-63.01
1.02
1.00
59.0
-59.5
-60.0
-60.5
-61.0
-61.5
-62.0
-62.5
-63.0
-63.5
-64.0
-64.5
-65.0
-65.5
-66.0
-66.5
f1 (ppm)

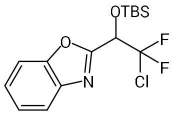

14-CDFE

2-(1-((*tert*-butyldimethylsilyl)oxy)-2-chloro-2,2-difluoroethyl)benzo[*d*]oxazole (14-CDFE)

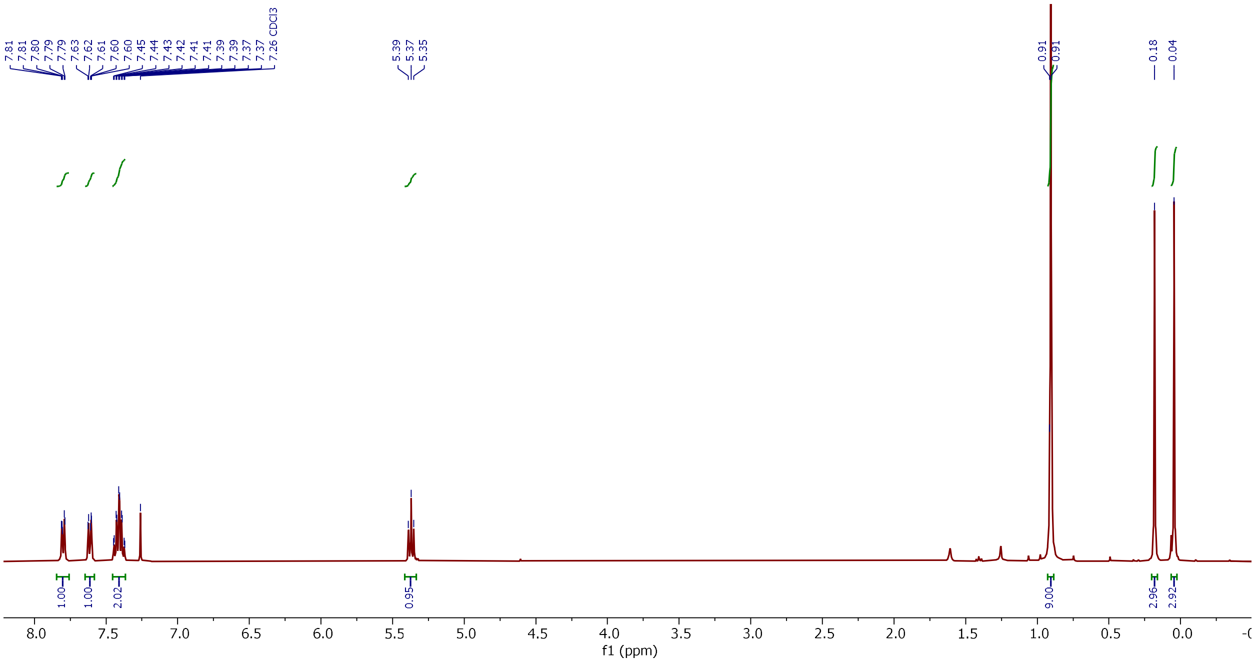

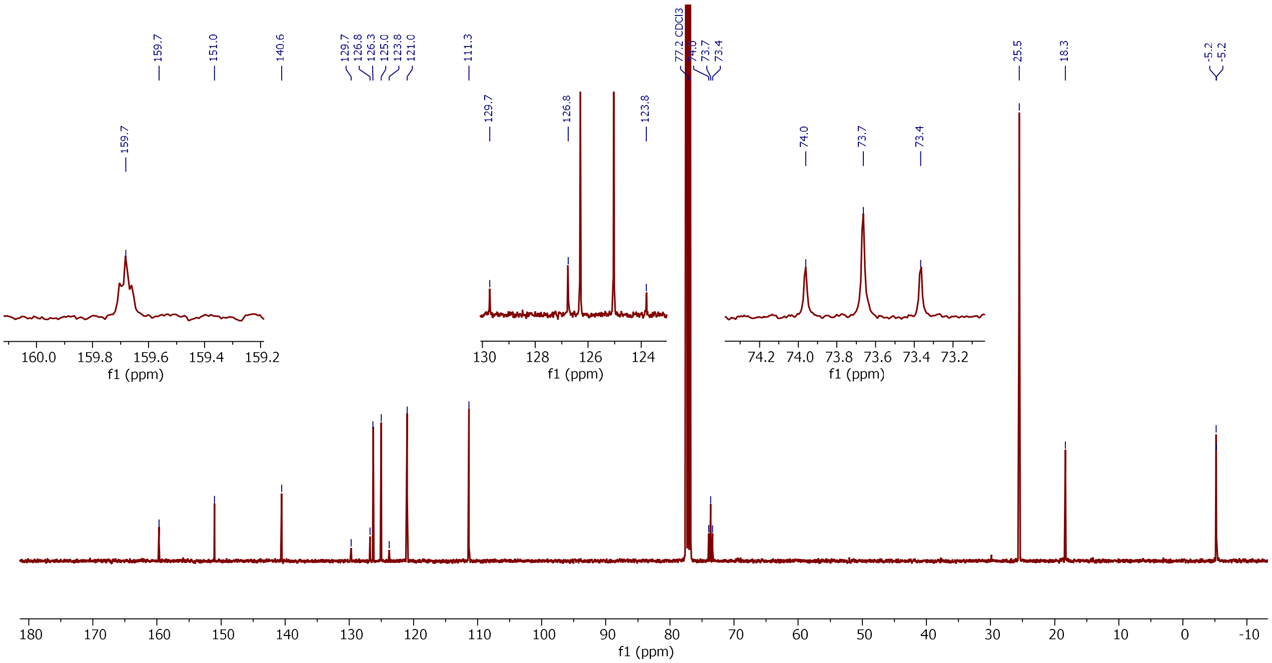

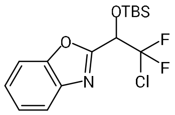

14-CDFE

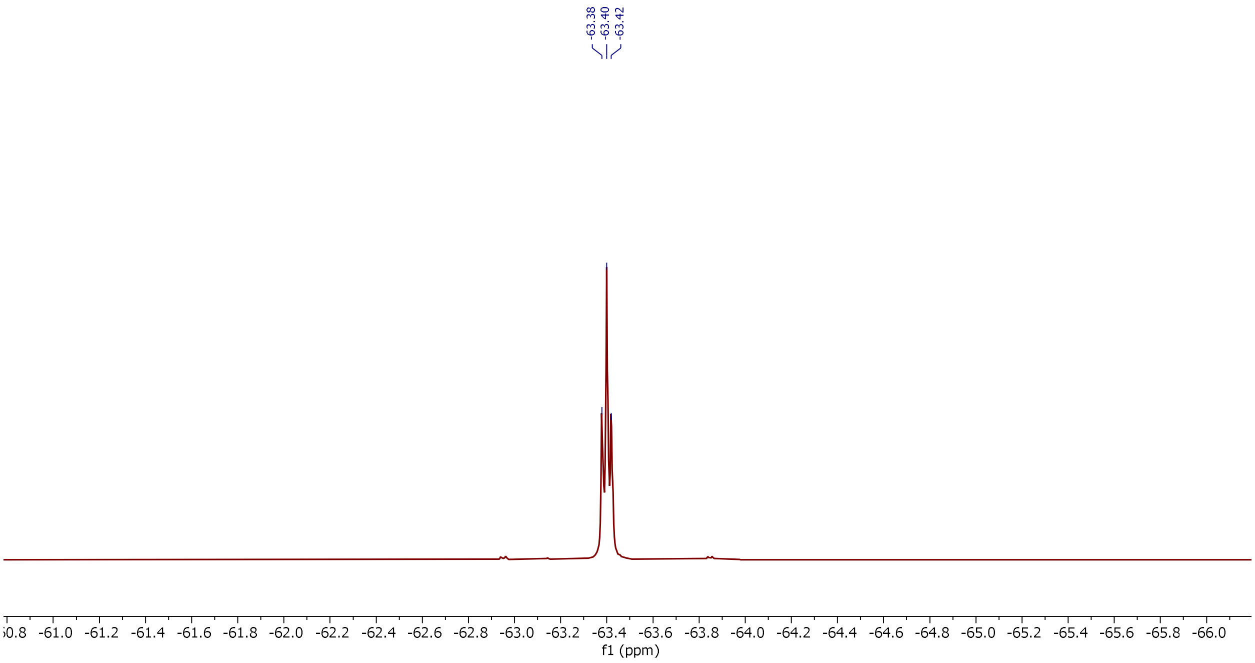

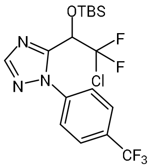

16-CDFE

5-(1-((*tert*-butyldimethylsilyl)oxy)-2-chloro-2,2-difluoroethyl)-1-(4-(trifluoromethyl)phenyl)-1*H*-1,2,4-triazole (16-CDFE)

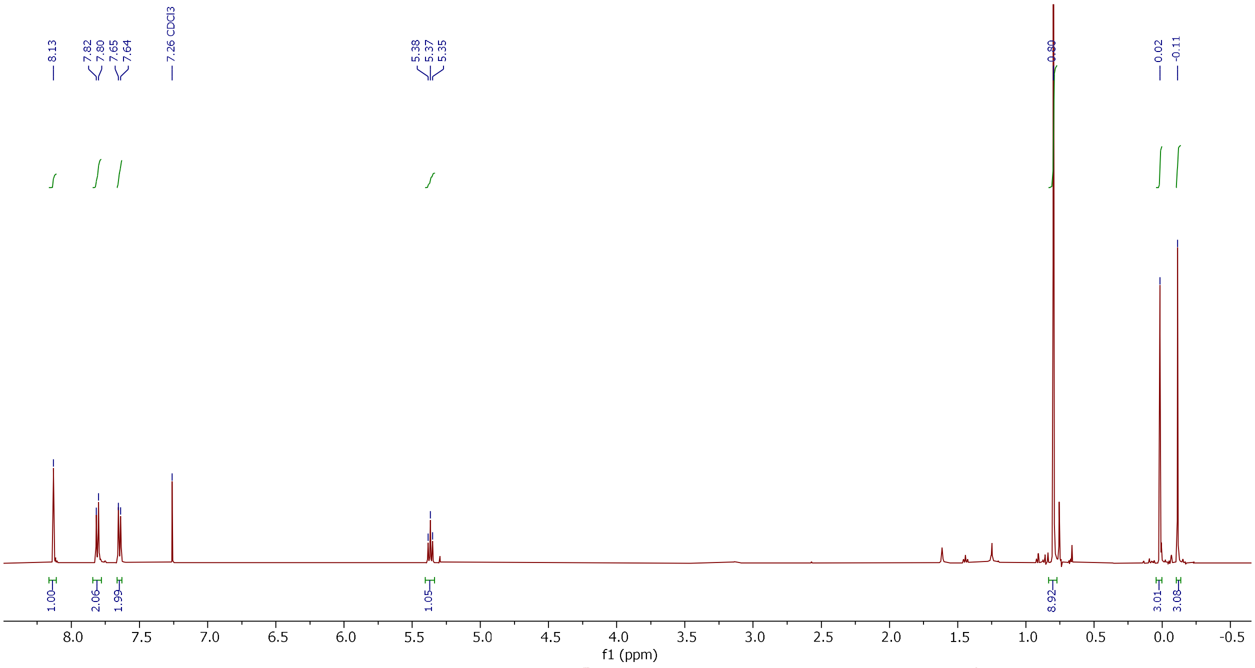

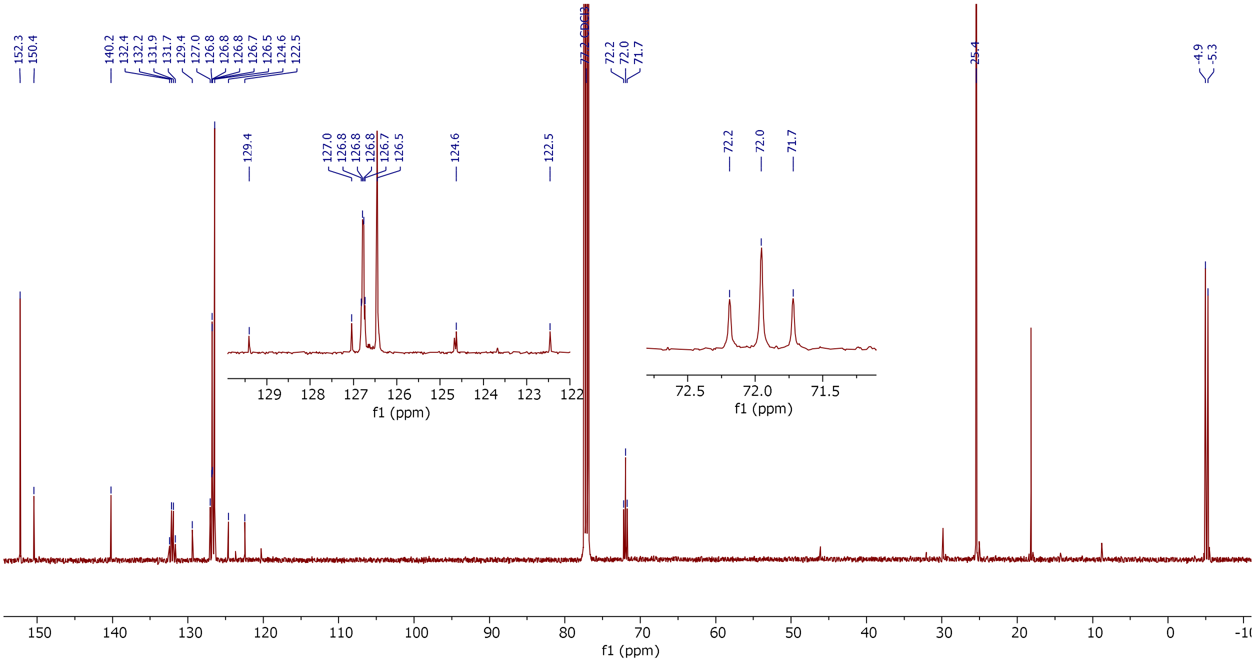

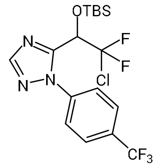
OTBS
N
F
F
N
N
Cl
CF3

16-CDFE

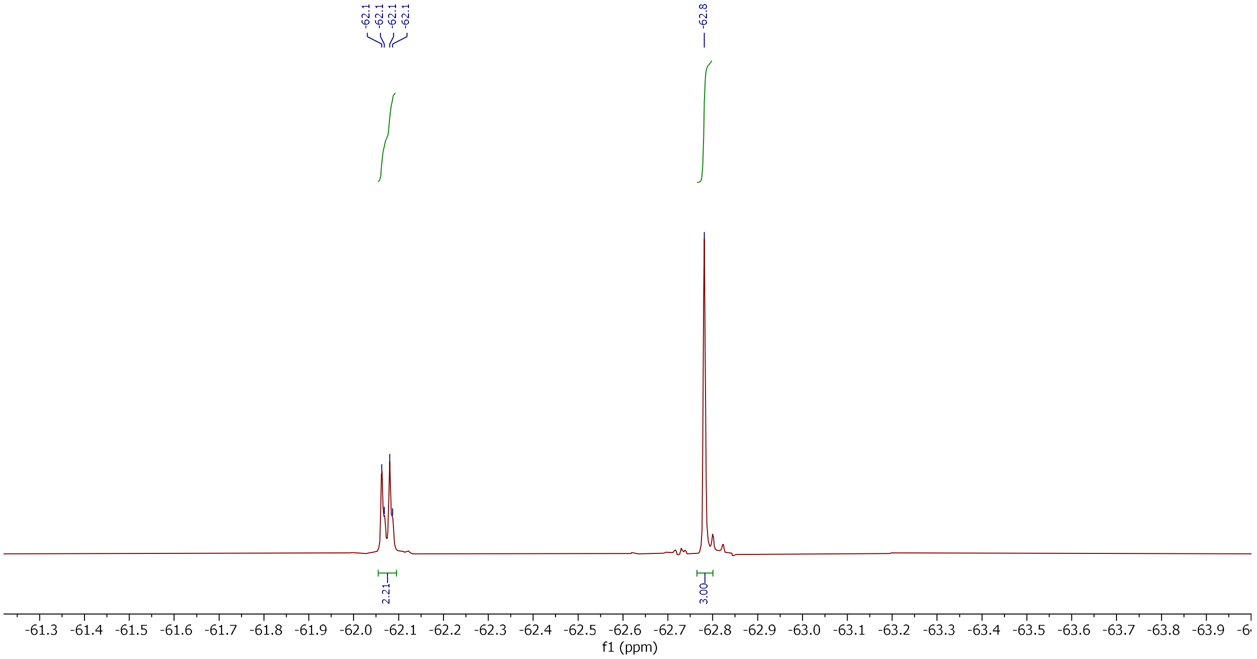
-62.1
-62.1
-62.1
-62.1
-62.8
2.21
3.00
-61.3 -61.4 -61.5 -61.6 -61.7 -61.8 -61.9 -62.0 -62.1 -62.2 -62.3 -62.4 -62.5 -62.6 -62.7 -62.8 -62.9 -63.0 -63.1 -63.2 -63.3 -63.4 -63.5 -63.6 -63.7 -63.8 -63.9
f1 (ppm)

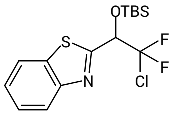

13-CDFE

2-(1-((*tert*-butyldimethylsilyl)oxy)-2-chloro-2,2-difluoroethyl)benzo[*d*]thiazole (13-CDFE)

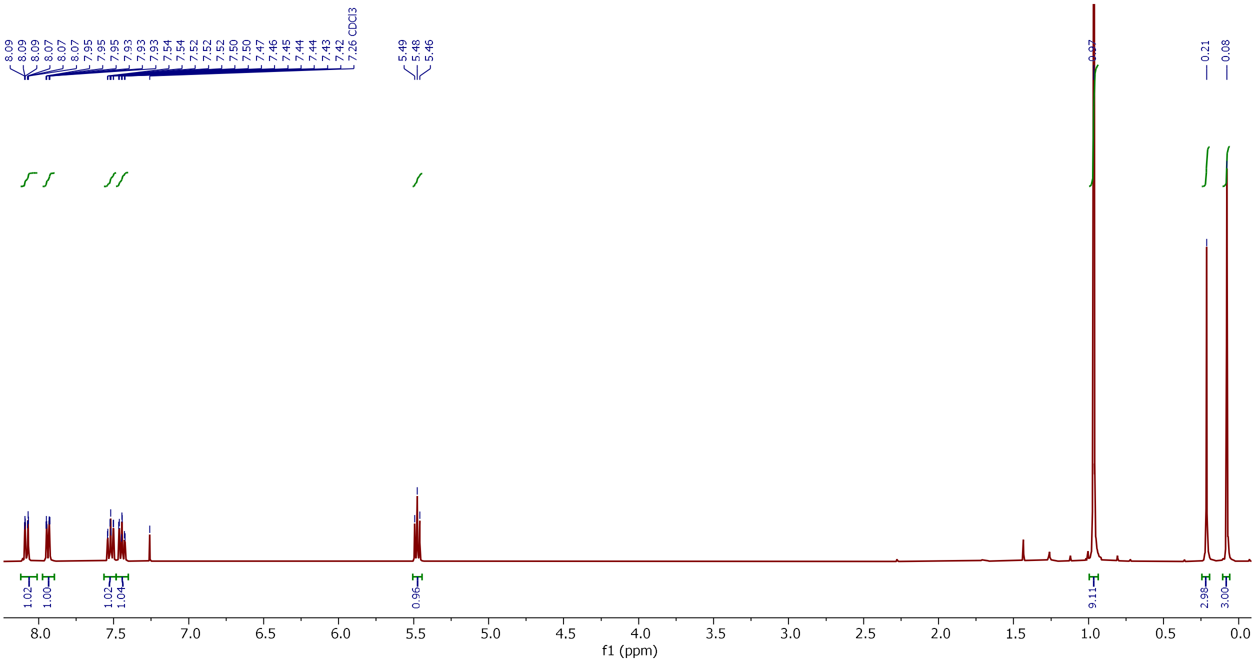

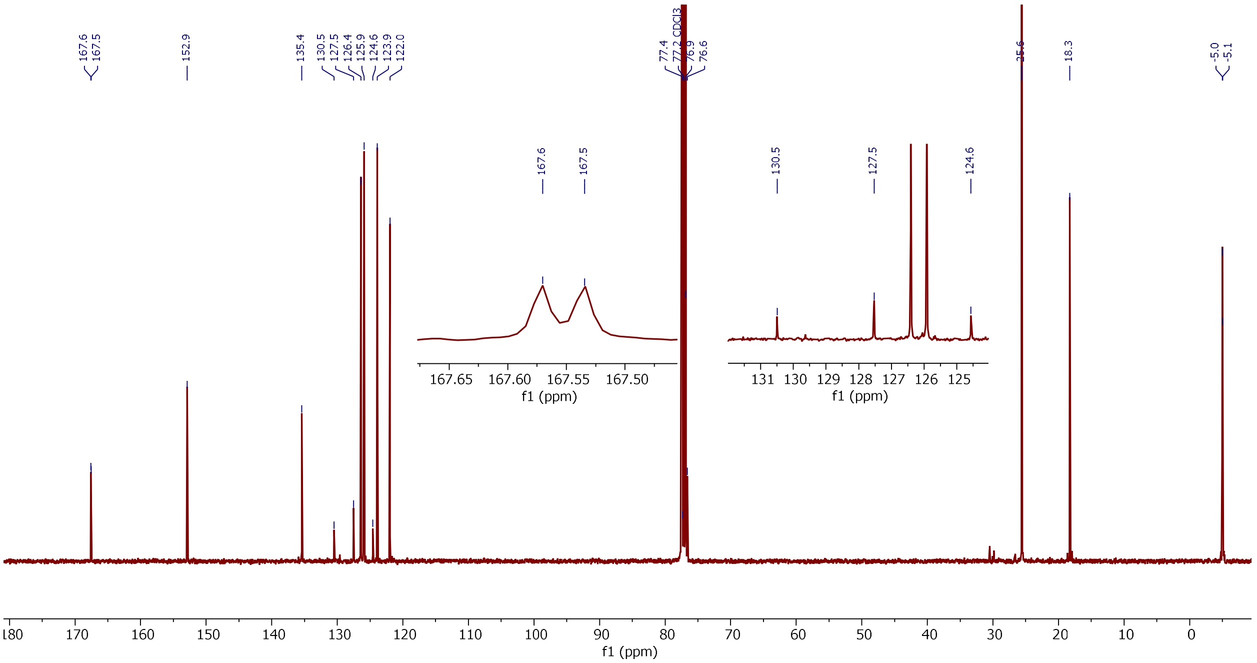

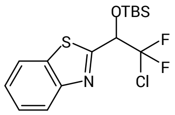

13-CDFE

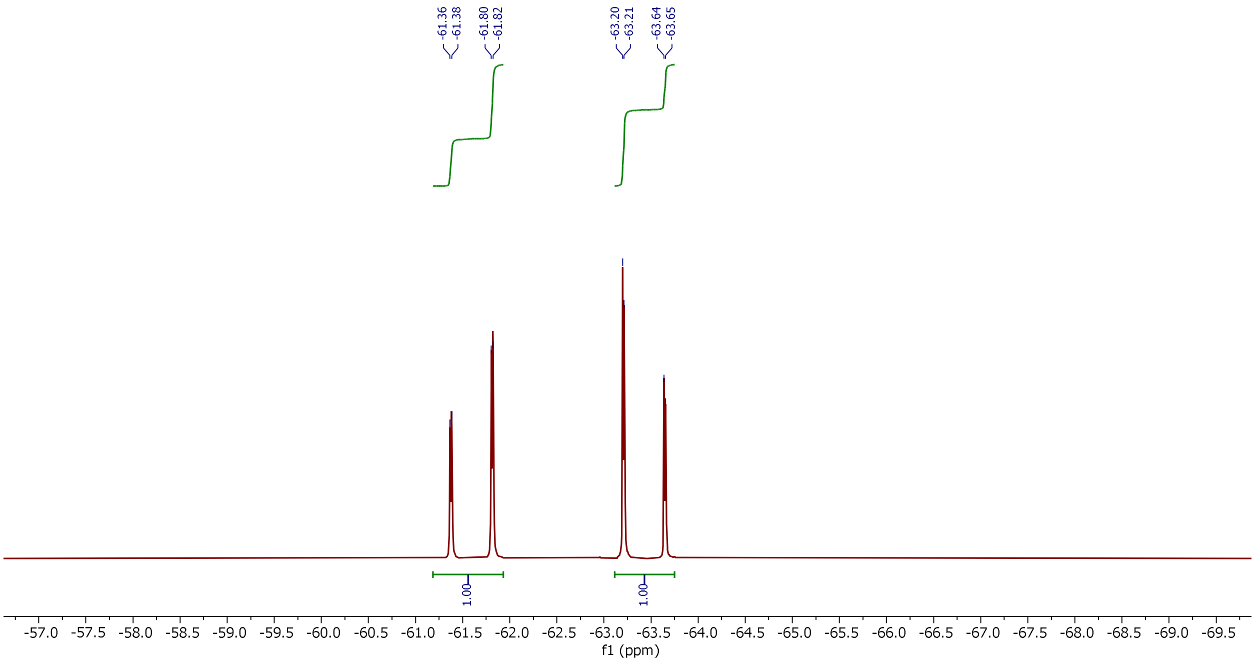

-61.36
-61.38
-61.80
-61.82
-63.20
-63.21
-63.64
-63.65
1.00
1.00
-57.0 -57.5 -58.0 -58.5 -59.0 -59.5 -60.0 -60.5 -61.0 -61.5 -62.0 -62.5 -63.0 -63.5 -64.0 -64.5 -65.0 -65.5 -66.0 -66.5 -67.0 -67.5 -68.0 -68.5 -69.0 -69.5
f1 (ppm)

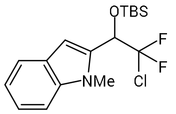

19-CDFE

2-(1-((*tert*-butyldimethylsilyl)oxy)-2-chloro-2,2-difluoroethyl)-1-methyl-1*H*-indole (19-CDFE)

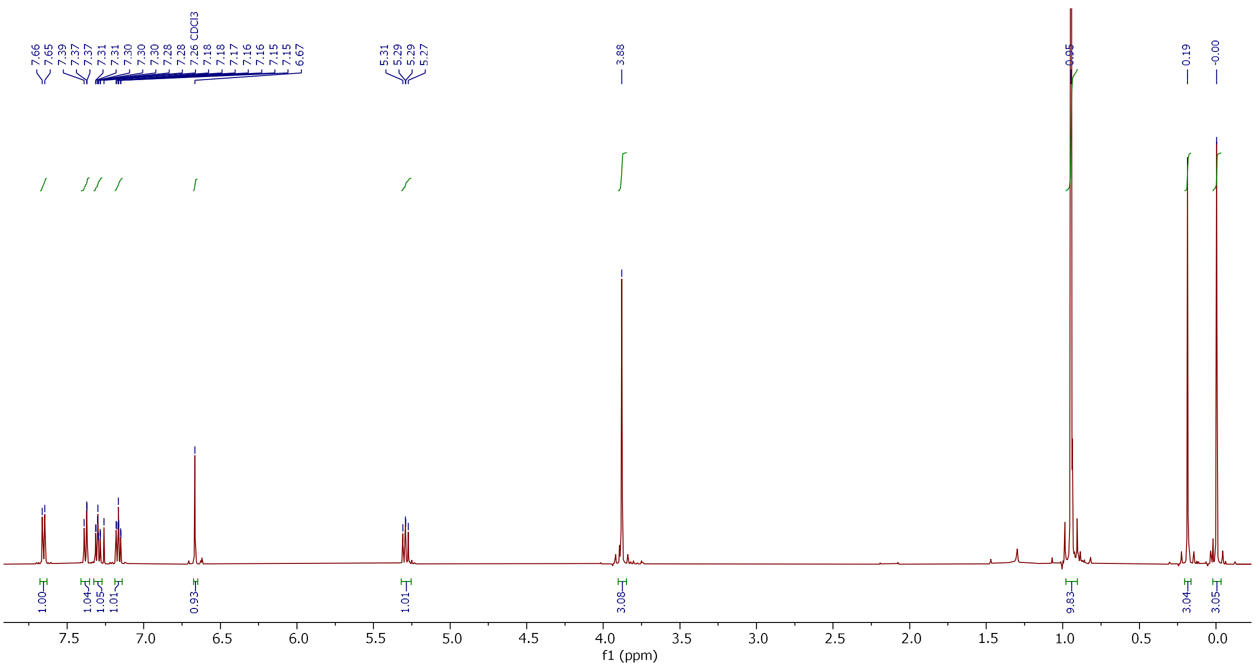

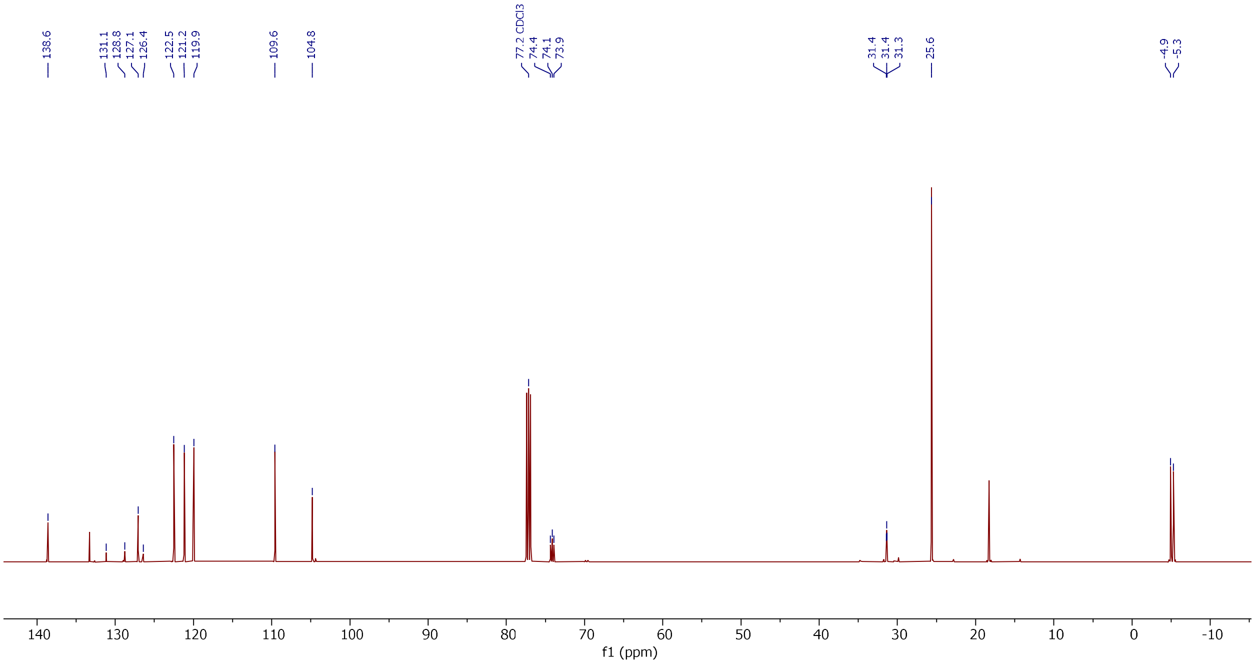

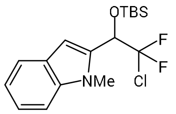

19-CDFE

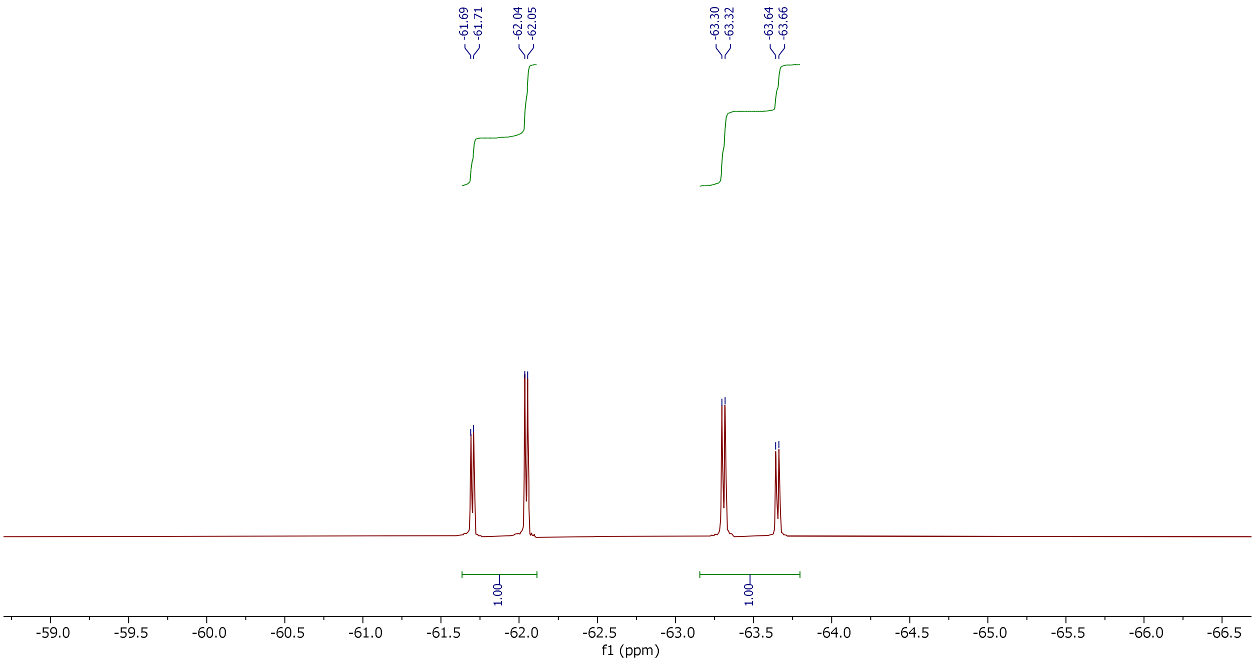

-61.69
-61.71
-62.04
-62.05
-63.30
-63.32
-63.64
-63.66
1.00
1.00
-59.0
-59.5
-60.0
-60.5
-61.0
-61.5
-62.0
-62.5
-63.0
-63.5
-64.0
-64.5
-65.0
-65.5
-66.0
-66.5
f1 (ppm)

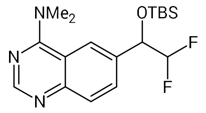

7-DFTBS

6-(1-((*tert*-butyldimethylsilyl)oxy)-2,2-difluoroethyl)-*N*,*N*-dimethylquinazolin-4-amine (7-DFTBS)

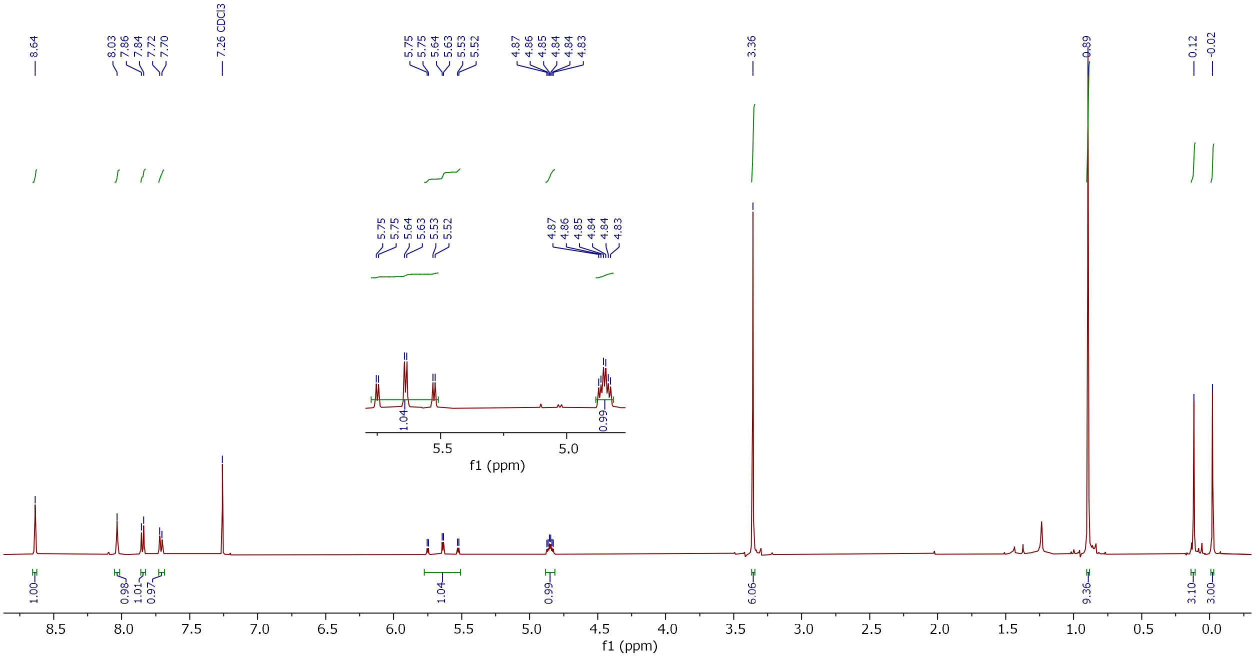

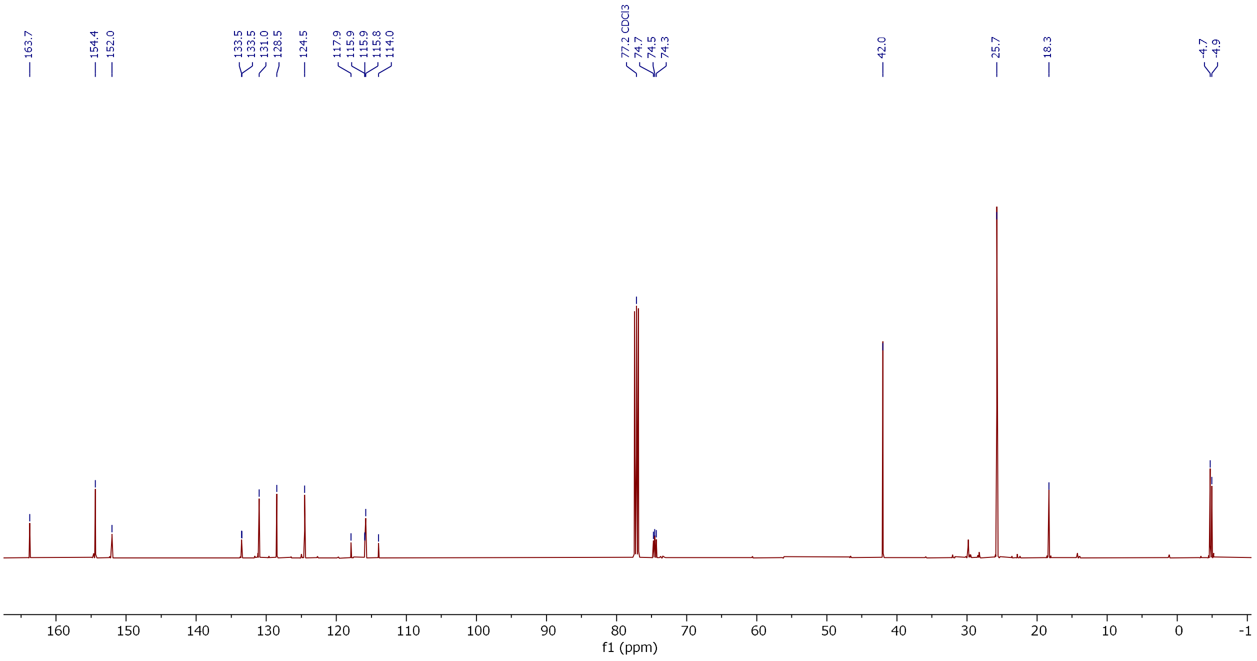

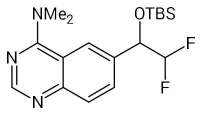
NMe2
OTBS
N
N
F
F

7-DFTBS

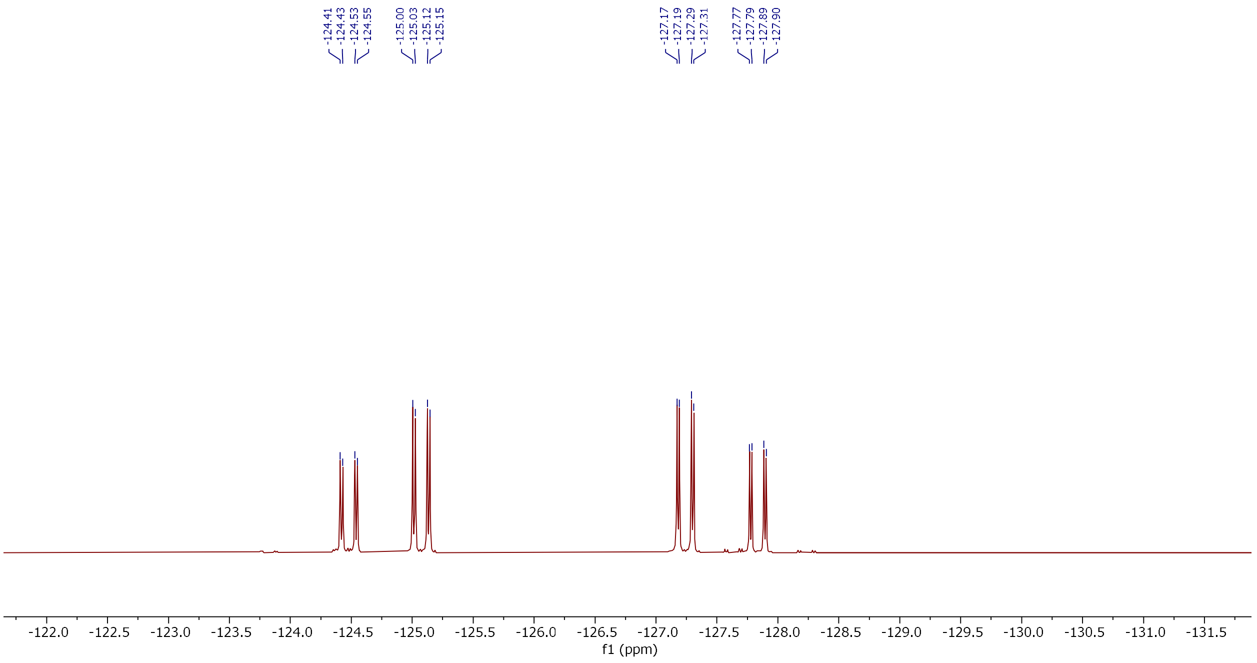
-124.41
-124.43
-124.53
-124.55
-125.00
-125.03
-125.12
-125.15
-127.17
-127.19
-127.29
-127.31
-127.77
-127.79
-127.89
-127.90
-122.0
-122.5
-123.0
-123.5
-124.0
-124.5
-125.0
-125.5
-126.0
-126.5
-127.0
-127.5
-128.0
-128.5
-129.0
-129.5
-130.0
-130.5
-131.0
-131.5
f1 (ppm)

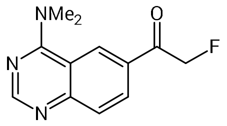

7-MFMK

1-(4-(dimethylamino)quinazolin-6-yl)-2-fluoroethan-1-one (7-MFMK)

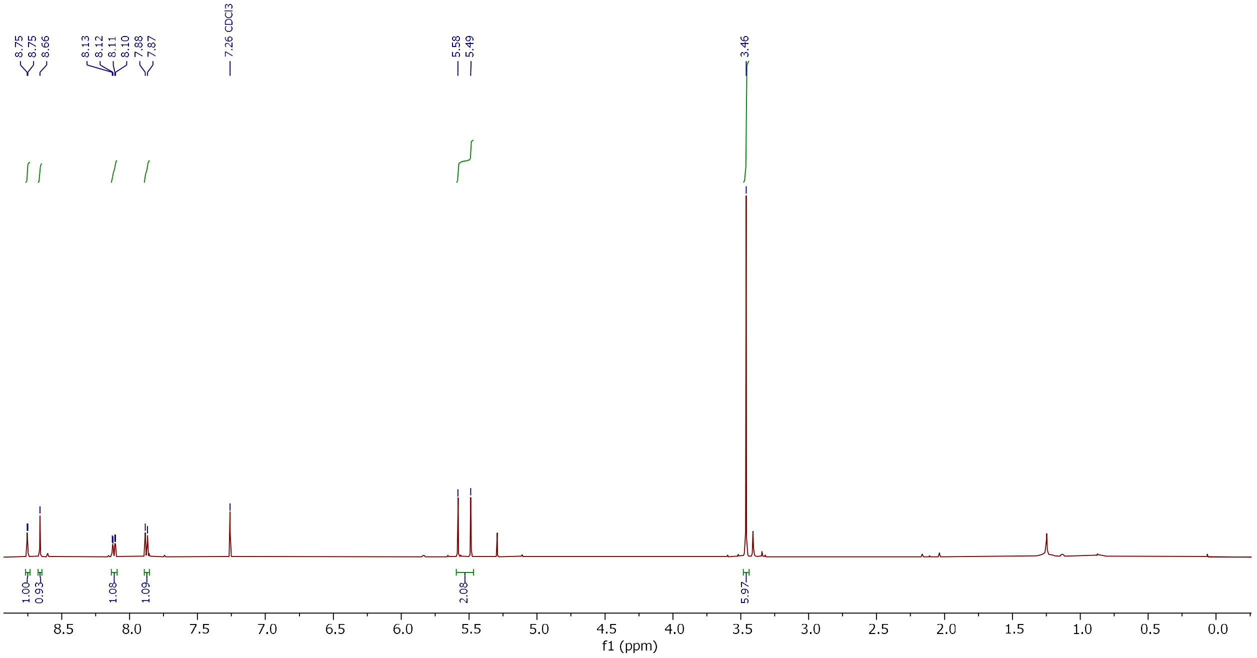

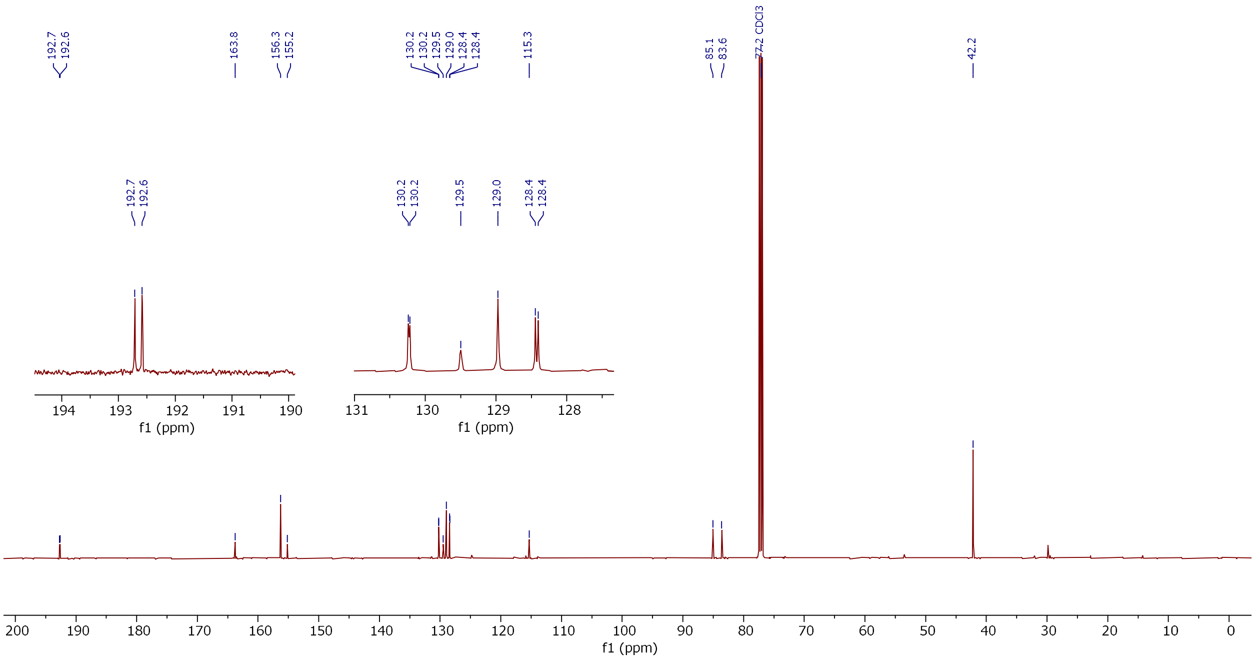

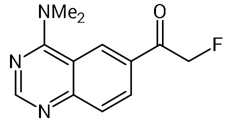
NMe2
O
F
N
N

7-MFMK

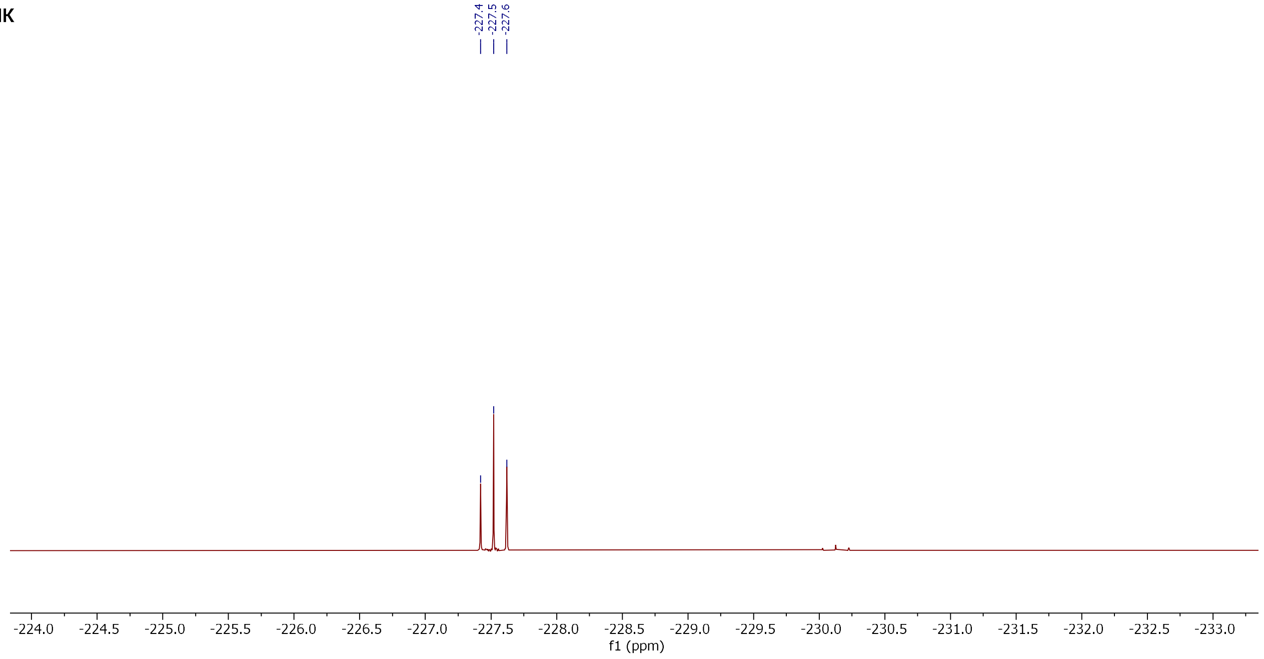
-227.4
-227.5
-227.6
-224.0
-224.5
-225.0
-225.5
-226.0
-226.5
-227.0
-227.5
-228.0
-228.5
-229.0
-229.5
-230.0
-230.5
-231.0
-231.5
-232.0
-232.5
-233.0
f1 (ppm)
